# Tandem RCM–Claisen Rearrangement–[2+2] Cycloaddition of *O,O'*-(But-2-en-1,4-diyl)-bridged Binaphthols

**DOI:** 10.3390/molecules171214531

**Published:** 2012-12-07

**Authors:** Michael Abraham, Wolfgang Reischl, Karl A. Kirchner, Alexander Roller, Luis F. Veiros, Michael Widhalm

**Affiliations:** 1 Faculty of Chemistry, Institute of Organic Chemistry, University of Vienna, Währinger Straße 38, A-1090 Wien, Austria; E-Mails: abraham350@gmail.com (M.A.); wolfgang.reischl@univie.ac.at (W.R.); 2 Institute of Applied Synthetic Chemistry, Vienna University of Technology, Getreidemarkt 9/163/AC, A-1060 Wien, Austria; E-Mail: karl.kirchner@tuwien.ac.at; 3 Faculty of Chemistry, Institute of Inorganic Chemistry, University of Vienna, Währinger Straße 42, A-1090 Wien, Austria; E-Mail: alexander.roller@univie.ac.at; 4 Centro de Química Estrutural, Instituto Superior Técnico, Universidade Técnica de Lisboa, 1049-001 Lisboa, Portugal; E-Mail: veiros@ist.utl.pt

**Keywords:** DFT calculations, Grubbs II catalyst, cage compounds, spiro-compounds, chiral macrocycle

## Abstract

Attempted RCM of 2,2'-bis(allyloxy)-1,1'-binaphthyl resulted in a Claisen-type rearrangement of a postulated labile dioxacyclodecine proceeding at room temperature and followed by [2+2] cycloaddition. Structures of products were confirmed by X-ray crystallography. A mechanistic rationalisation based on relative stabilities of proposed intermediates and transition states is provided.

## 1. Introduction

A sequence of reactions is designated as a tandem process if the reacting functional group(s) of each step are formed or activated only in the preceding one and with no need of adding reagents for individual steps. A further requirement is that all reactions proceed under the same conditions with no mutual interference of by-products. Since advantages over conventional multistep synthesis by avoiding work-up and purification of intermediates are obvious, such processes have found widespread application [1,2,3,4,5,6,7,8,9,10,11]. With increasing complexity of target structures functional group tolerance and stereoselectivity of transformations become crucial and are particularly challenging in the synthesis of natural products and biologically active compounds. Consequently, the usefulness of tandem reactions will also rely on a sufficient high degree of stereocontrol in each step. This requirement is often fulfilled with rearrangements proceeding via cyclic transition states and with sigmatropic rearrangements which represent stereospecific transformations. Typical examples are Cope and Claisen rearrangements and variations like Claisen-Ireland, Claisen-Johnsen, Meerwein-Eschenmoser-Claisen, thio- and aza-Claisen, and Carrol rearrangement [12,13,14,15,16,17,18,19,20,21]. A special situation arises when atropisomeric biaryls are involved translating axial-chirality of the substrates into centro-chirality of the products provided the reaction proceeds at a temperature where no racemisation of the biaryl takes place. Particularly *O,O'*-disubstituted binaphthol derivatives have shown various rearrangements and cyclisations ending up with configurationally stable polycyclic structures formed on cost of the aromaticity of one of the benzene rings [22,23]. While most of published procedures require elevated temperature to proceed, the present process takes place below 60 °C and affords a single rearranged product in excellent yield. Such transformations offer a unique access to otherwise difficult to synthesize centrochiral compounds, eventually useful as chiral building blocks [24]. The present paper reports on a tandem sequence where up to three intramolecular transformations are involved producing stereoselectively spiro- and finally chiral cage-compounds from simple binaphthyl precursors.

## 2. Results and Discussion

### 2.1. Synthesis and Rearrangements

**Scheme 1:**
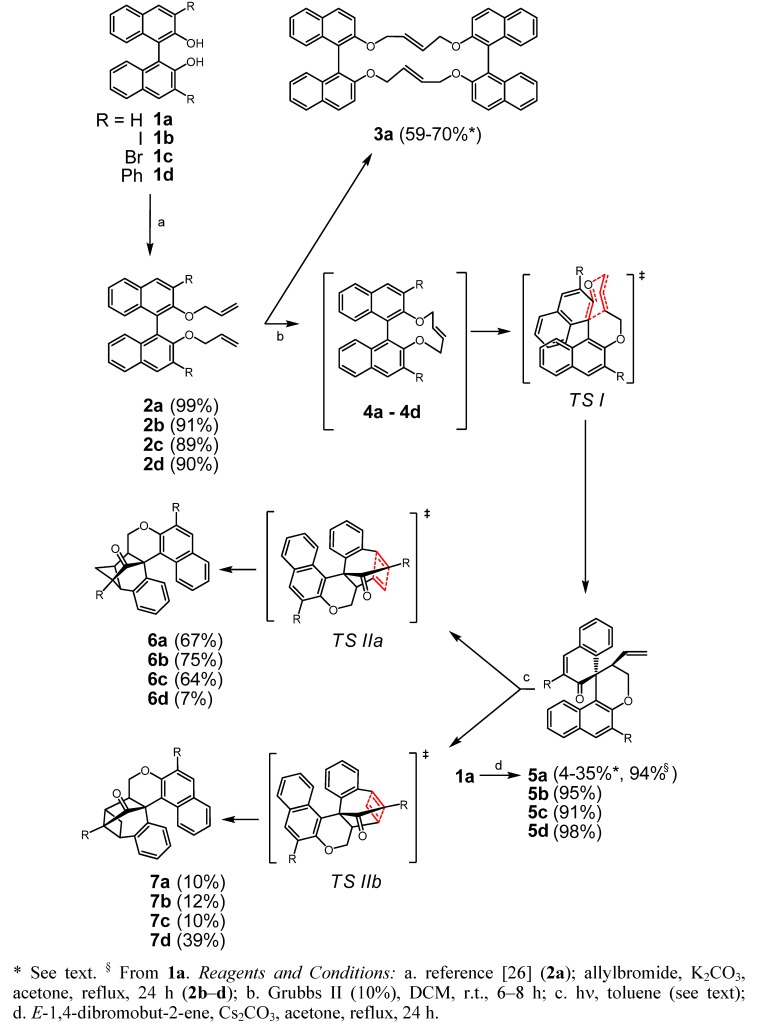
Rearrangement of 1,1'-binaphthyl derivatives with 2,2'-*O*-allyl fragments.

**Scheme 2:**
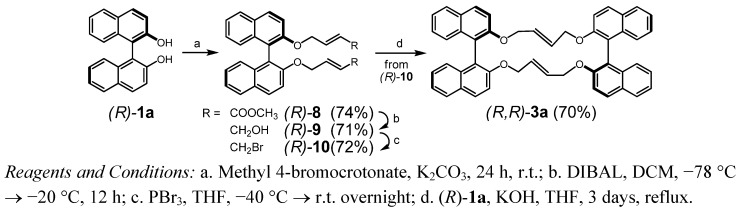
Synthesis of *(R,R)*-**3a** through macrocyclisation.

In course of our attempts to synthesize macrocyclic chiral (di)olefins with incorporation of biaryl units, eventually useful as chiral ligand in asymmetric catalysis, we investigated the RCM of 2,2'-bis(allyloxy)-1,1'-binaphthyl (**2a**), a versatile precursor previously applied in the synthesis of ring systems [25,26,27]. While RCM of a homologue of **2a** (4-butenyloxy instead allyloxy in positions 2 and 2′) proceeded as expected, bridging positions 2 and 2′ of the binaphthol and yielding the dioxacyclododecine product as a *cis/trans* mixture [28], compound **2a** behaved completely different (Scheme 1). Treatment with Grubbs II catalyst (DCM, reflux) resulted in the formation of a *C_1_* symmetrical species (35%) and a product with higher symmetry (59%). While in the latter case ESI-MS gave a molecular peak of *m/z* 699.3 and correct HRMS for a dimeric structure **3a** with *D_2_* symmetry, the former one gave the correct mass of *m/z* 338.2 for the desired product, but disagreeing NMR spectra for structure **4a**, with the spectroscopic data pointing rather to a rearranged product **5a** [29,30]. In order to increase the yield of the macrocycle we repeated the cyclisation at r.t. with enantiopure precursor (*R*)-**2a**. Under these conditions 15% of starting material was recovered after 10 h and only small amounts (4%) of **5a** were formed. As the main product 70% of dimer **3a** ([α]_D_^20^ +110) was isolated (82% rel. to recovered starting material) thus confirming the relative biaryl configurations in the racemic product to be (*R*)_ax_(*R*)_ax_/(*S*)_ax_(*S*)_ax_ and absence of “*meso*-**3a**” with (*R*)_ax_(*S*)_ax_ configuration. The *trans*-geometry of double bonds was confirmed by macrocyclisation of dibromide (*R*)-**10** with (*R*)-**1a** yielding the same product obtained by RCM (Scheme 2). A crystal structure of **3a** was published [24].

It is interesting to note that the temperature plays a decisive role in the outcome of this reaction. According to Piedra *et al.* the formation of dimers can be suppressed at elevated temperature (3 h, 120 °C, MW) and at higher dilution, while variation of the catalyst showed little influence. This behaviour seems nearly unaffected from substituents in positions 6 or 7 of the naphthalene rings [24]. In contrast, when using substrates **2b**–**d** with substituents in position 3 no dimeric species could be detected (see below).

The rearrangement of an aryl-allylether undergoing a Claisen-type rearrangement is usually formulated via a concerted or radical mechanism generating an aryloxy- and allyl radical in the latter case (photo-Claisen rearrangement) [31]. As the most likely precursor for **5a** we postulated labile (*E*)-dioxacyclodecine **4a**, which might smoothly traverse a chair-like transition state *TS I* to give spiro compound **5a**. The involvement of (labile) **4a** with an *E*-double bond was supported since **5a** was obtained as the exclusive product when reacting **1a** with *E-*1,4-dibromobut-2-ene (Cs_2_CO_3_, acetone, 20 h reflux, 94%) [32]. Mechanistic implications will be discussed below.

If **5a** was irradiated with long wave-length UV light (>300 nm) in toluene for 3 h at ambient temperature, a mixture of two rearranged products **6a** (67%) and **7a** (10%) was formed through intramolecular [2+2] cycloaddition (6% of starting material was recovered). Inspection of a wire model revealed that particularly for the formation of the predominating isomer **6a** the required conformation for a transition state like *TS IIa* can be easily adopted. While intramolecular [2+2] cycloadditions of enones with olefins have been frequently observed [33,34], an analogous cyclisation of 1-allyl-naphthalen-2(1*H*)-one was only reported in 1980 [35,36].

**Figure 1:**
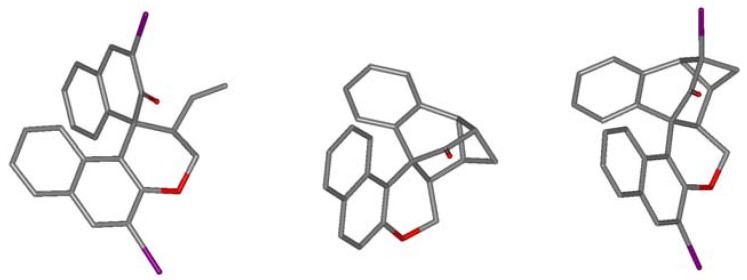
Crystal structures of **5b** (left), **6a** (middle) and **6b **(right). H-atoms and solvent molecules omitted for clarity.

Similar treatment of 3,3′-diiodo substituted substrate **2b** showed similar conversions and corresponding products obtained, but displaying significantly different stability/reactivity. Thus, RCM of **2b** at r.t. in the dark yielded exclusively **5b** (95%) without detectible amounts of dimerisation product. If light was not rigorously excluded some rearranged product **6b** (10%) was also detected. Its structure was confirmed by crystal structure analysis (Figure 1). When a solution of **5b** in toluene was irradiated with visible light (60 W light bulb) for several hours, complete photoaddition took place yielding a mixture of **6b** and **7b** (approx. 9:1). The isomers were separable by chromatography and fully characterized by NMR and MS. The ease of accessing cyclobutanes **6b** and **7b** prompted us to perform the sequence **2b**→ **4b** **→** **5b** **→** **6b/7b** in one pot. Conducting the RCM in toluene under irradiation with visible light resulted in exclusive formation of **6b/7b** (85:15, 89% total yield). For comparative studies also bromo- as well as phenyl-substituted *O*-allyl precursors **2c** and **2d**, respectively were investigated. Their behavior on treatment with Grubbs II catalyst was virtually the same, yielding **5c** and **5d** in excellent yield (91% and 98%, respectively) and subsequent photoisomerisation under UV irradiation gave **6c**/**7c**(64%/10%) and **6d**/**7d**(7%/39%), respectively. 

**Scheme 3:**
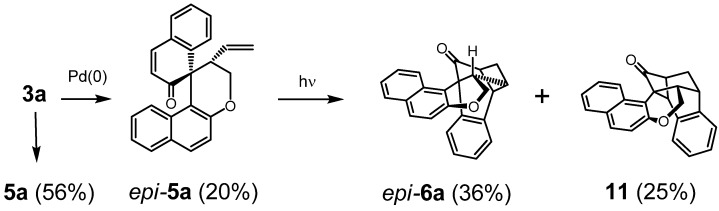
Pd(0) mediated cleavage of **3a** and subsequent photoisomerisation of *epi*-**5a**.

In view of intended use in asymmetric catalysis macrocyclic diolefin **3a** was treated with [RhCl(C_2_H_4_)_2_]_2_, [C_3_H_5_PdCl]_2_, and Pd_2_(dba)_3_ precursors which did not result in the formation of complexes, instead Pd(PPh_3_)_4_ caused complete cleavage yielding a mixture of **5a** (56%) and *epi*-**5a** (20%). (Scheme 3) A tentative mechanistic interpretation is discussed in the Supporting Information. 

**Figure 2:**
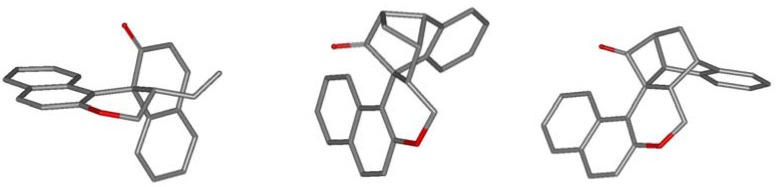
Crystal structures of *epi*-**5a** (left), *epi*-**6a** (middle), and **11 **(right). H-atoms and solvent molecules omitted for clarity.

Subsequent irradiation of *epi*-**5a** for 0.5 h afforded two products. The main product was expectedly the corresponding epimer of **6a**, *epi*-**6a** (36%) while no *epi-***7a** could be detected. Instead cyclobutanone **11** (25%) was formed. Both structures were confirmed by X-ray analysis (Figure 2).

### 2.2. Calculations, Evidence for Existence of ***4a***, Its Geometry and Rearrangement to ***5a***

Geometries and energies of ground state structures as well as transition states were obtained using program packages SPARTAN (B3LYP, MO6 and MP2) and GAUSSIAN09 (B3LYP) [37,38]. For comparability reasons B3LYP data as determined in the absence of solvent and, where appropriate, also in DCM (values in parenthesis) are collected in Table 1 and are discussed below.

**Table 1:** B3LYP (Ru: sdd, all other atoms: 6-31G**), MO6, and MP2 calculated free energies of activation based on ground state and transition state energies ^a^ for conversion of **2a-***pro-cis-* and **2a-***pro-trans***-Ru**, *cis***- **and *trans***-Ru-Cycl**, *E-* and *Z-***4a**, and*E-***12**. Asterisks (*) denote results obtained with the corresponding biphenyl system.

	ΔG^‡ b^		
	B3LYP	MO6	MP2
**2a-***pro-trans*-Ru* **→ ***trans*-Ru-Cycl*	1.0 (1.8)		
**2a-***pro-cis*-Ru* **→ ***cis*-Ru-Cycl*	2.2 (3.2)		
*trans*-Ru-Cycl* → *E***-4a-**Ru*	17.2 (19.4)		
*cis*-Ru-Cycl* → *Z***-4a-**Ru*	7.7 (9.4)		
*E-* **4a ** **→ 5a**	26.4 (26.2)	27.3	21.0
*Z-* **4a ** **→ 5a**	32.3 (33.0)	32.2	26.9
*E-* **12 ** **→ ** *E-* **4a**	3.9 (6.2)	8.3	6.0
*E-* **12 ** **→ 5a**	8.3 (9.7)	10.6	8.2
*E-* **12 ** **→ ** *epi-* **5a**	8.6 (9.8)	10.4	7.8
*E-* **12 ** **→ ** *E-* **13**	39.1	40.0	35.2
*E-* **12 ** **→ 14**	7.3	13.5	13.6
*E-* **12 ** **→ ** *epi-* **14**	7.1	14.4	16.2
*E-* **4a ** **→ ** *epi-* **5a**	39.1	41.4	39.2

^a^ For details see Supporting Information; ^b^ kcal/mol in vacuo and DCM (in parentheses).

**Scheme 4:**
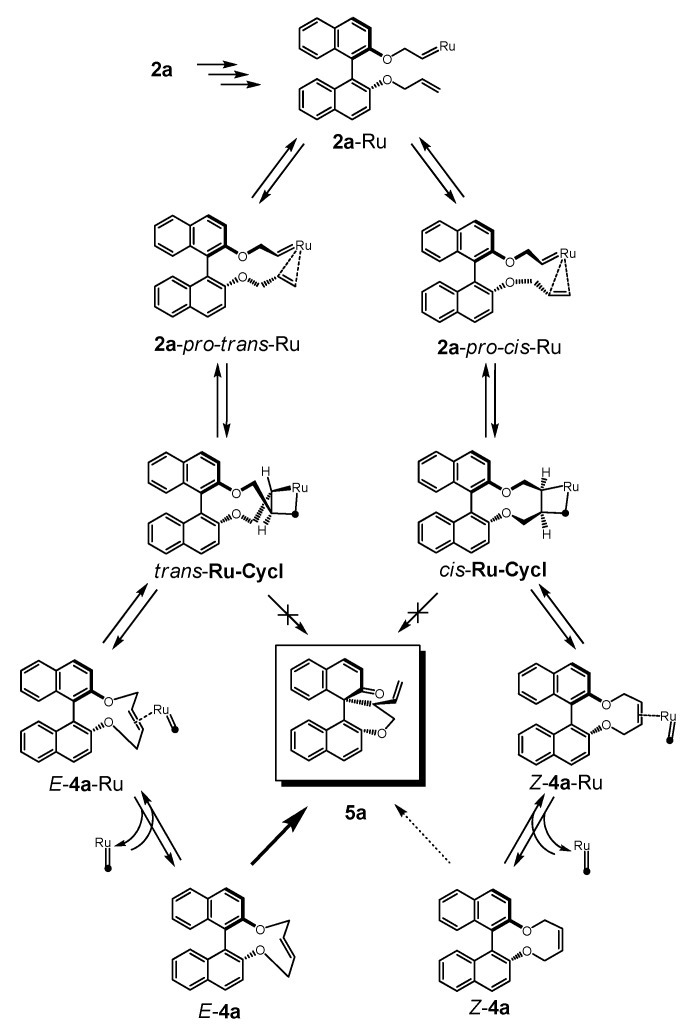
Formation of **5a** from **2a** and postulated intermediates of RCM.

To trace the transformation from **2a** into **5a** under metathesis conditions several possibilities had to be taken into account as outlined in Scheme 4 and energy profiles of alternative reaction paths were determined. Our calculations are based on the established mechanism of the olefin metathesis cyclisation with Grubbs II catalyst [39,40,41] and are carried out on a corresponding biphenyl skeleton which is expected to be a realistic model for the transformation **2a** → **4a**. Calculations on olefin metathesis reaction with ethylene and Grubbs II catalyst supported a dissociative path with *trans*-coordination of the olefin as a barrierless step [42]. Applied to our system an olefin-Ru-carbene, **2a**-Ru, is postulated which coordinates the pending olefin with either *re*- or *si*-side to form (transient) intermediates in the configuration determining step from which 2,3-*cis*- or 2,3-*trans*-substituted metalla-cyclobutanes are obtained. Cycloreversion leads to Ru-olefin complexes of **4a** and after dissociation *Z*- and *E*-**4a** could undergo Claisen rearrangement to **5a**. A direct transformation of a Ru-cycle into **5a** without traversing **4a** would proceed via an *anti*-aromatic transition state (8π electrons) and seems highly unfavourable.

**Figure 3:**
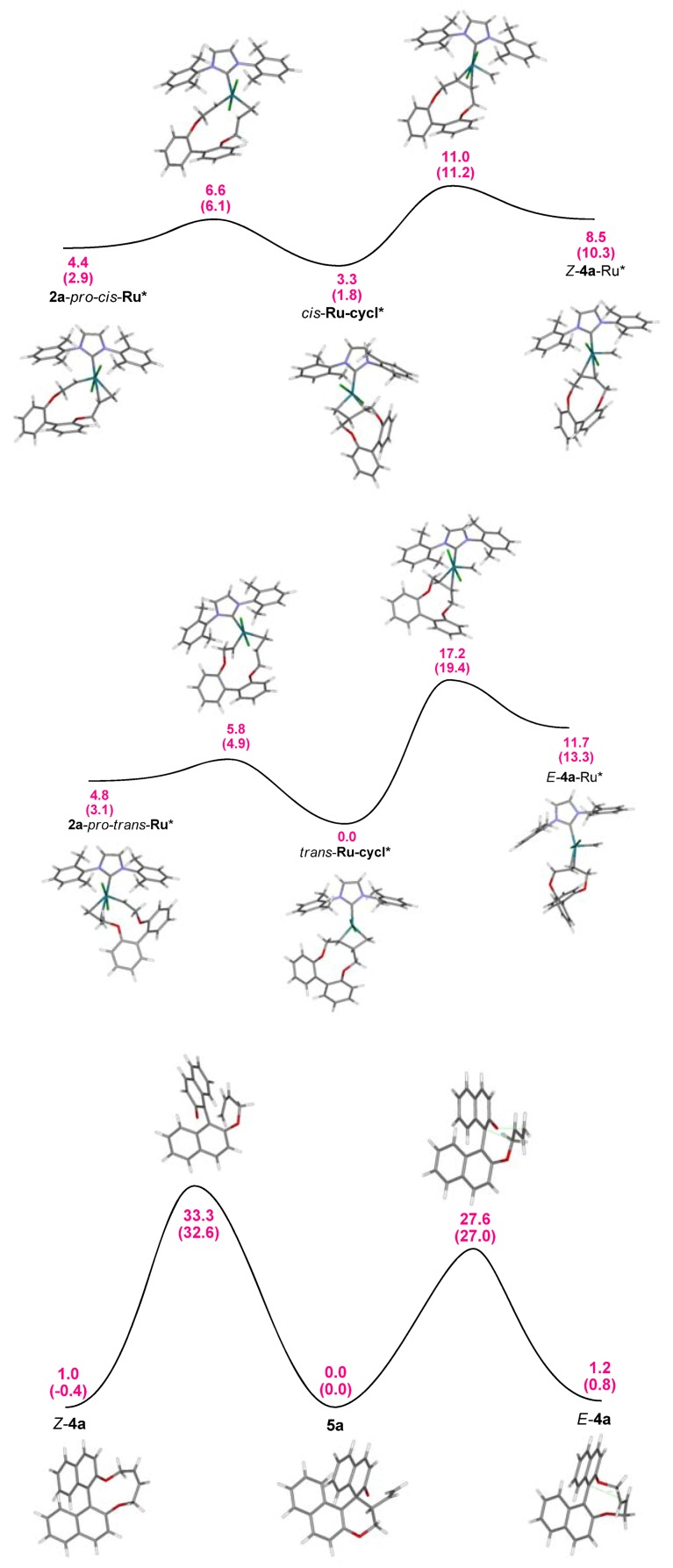
Energy profile (B3LYP, single point energies in DCM in parenthesis) for the formation of *E* and *Z*-olefin-carben-Ru complexes formed in course of a RCM with Grubbs II catalyst. Calculations were performed on a simplified system using the corresponding biphenyl species.

It was found that the metallacycle with *trans* geometry is more stable (ΔG^0^ = 3.3 kcal/mol), but less reactive [ΔG^‡^ = 17.2 kcal/mol *versus* 7.7 kcal/mol, (19.4 kcal/mol *versus* 9.4 kcal/mol)] yielding *E*-**4a**-Ru in a slightly more endergonic reaction (Figure 3, top and middle). 

The subsequent (presumably) barrierless dissociation delivers *E-* and *Z*-olefins. Although ground state energies of *E***-** and *Z***-4a** are very similar (ΔG^0^ < 0.2 kcal/mol), the barriers for the Claisen rearrangement (Figure 3, bottom) differ significantly from each other with 26.2 kcal/mol (from *E*-**4a**) and 33.0 kcal/mol (from *Z*-**4a**) and moreover, both are markedly higher than all barriers between intermediates of the preceding RCM. Accordingly, these intermediates are in equilibrium and the Claisen rearrangement is rate determining proceeding exclusively via *E*-**4a** with the equilibrium on the product side (ΔG^0^ = 1.2 kcal/mol, 0.8 kcal/mol in DCM). It should be pointed out that the overall reaction proceeds under Curtin–Hammett conditions and as a consequence the formation of **5a** via *E*-**4a** and *Z*-**4a** depends only on energy differences of corresponding transition states ΔΔG^‡^ = 5.7 kcal/mol (5.6 kcal).

**Scheme 5:**
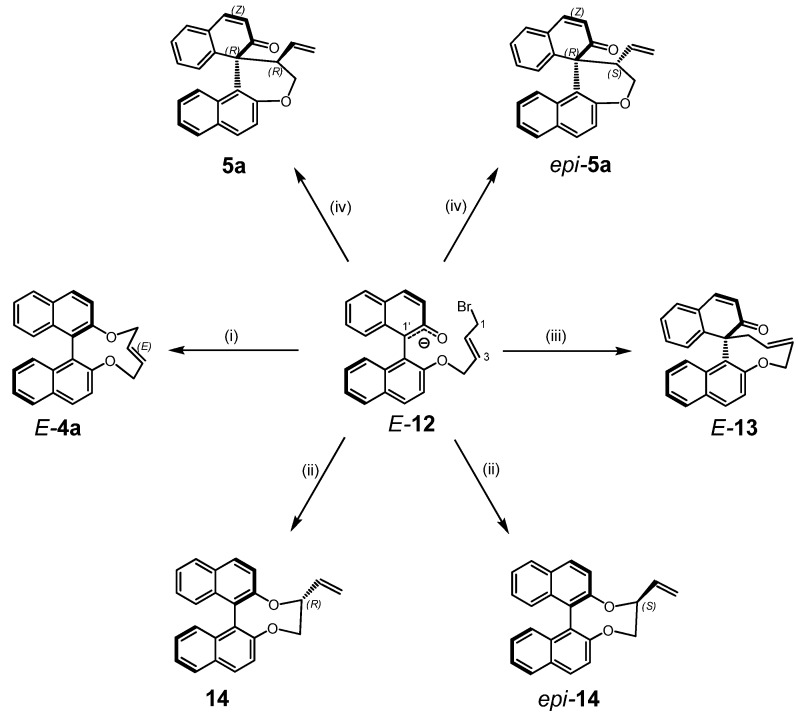
Hypothetic reaction paths (i)–(iv) of anion *E*-**12**.

This is also in agreement with the course of the base catalyzed reaction of **1a** with *E*-1,4-dibromo-but-2-ene at elevated temperature. The first step obviously forms the (mono)anion *E-***12 **through *O*-allylation of **1a** (Scheme 5). Formally this intermediate can undergo four intramolecular nucleophilic substitutions (i)–(iv) with attack of either phenolate or carbanion at either C1 or C3 of the allyl moiety (S_N_ or S_N_' reaction). 

**Figure 4:**
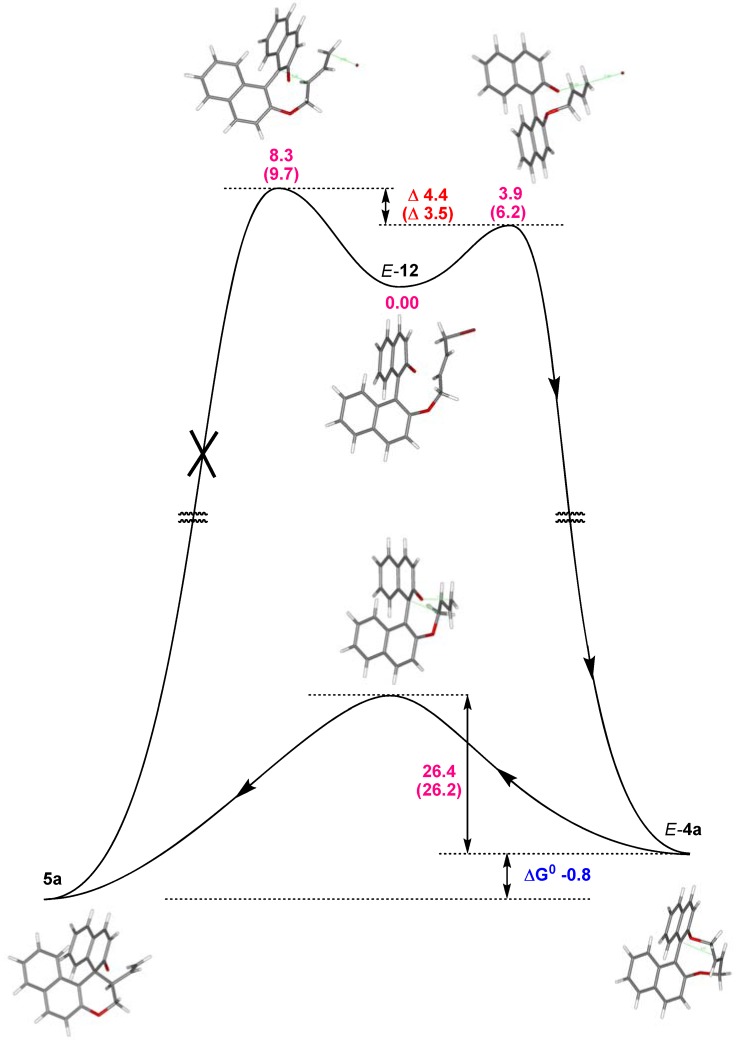
Energy profile (B3LYP) for the formation of **5a** from *E-***12 **under vacuum and in DCM (values in parenthesis).

Since all steps yielding neutral intermediates/products from a charged species *E*-**12** concomitant with precipitation of KBr, these will proceed under kinetic control and are virtually irreversible. Under the applied conditions *E/Z* isomerisation of **12** can be ruled out, but the flexibility of the key intermediate *E*-**12** resulted in a Boltzmann distribution of conformations with several local minima [43]. The lowest transition state with 3.9 kcal/mol (6.2 kcal/mol) was found for path (i) yielding *E-***4a**. For all other processes significantly higher barriers were obtained with 7.3 kcal/mol and 7.1 kcal/mol for (ii), 8.3 kcal/mol (9.7 kcal/mol) and 8.6 kcal/mol (9.8 kcal/mol) for (iv), and even more pronounced for (iii) with 39.1 kcal/mol. This clearly points to a two-step process with *E-***4a** as an intermediate as depicted in Figure 4 [44]. Energies obtained with MP2 and MO6 show similar trends. 

Transformations of **5a**–**d** into **6a**–**d** and **7a**–**d**, as well as the formation of **11** from *epi-***5a**, are triggered by light and exited states are obviously involved. Due to their sufficient energy content various diradical structures may be readily derived therefrom and have to be considered as feasible intermediates [45,46,47]. A detailed investigation for better understanding of these processes is presently under way and will be published in near future.

## 3. Experimental

### 3.1. General

Melting points: Kofler melting point apparatus, uncorrected. NMR: recorded at 400.27 MHz (^1^H) and 100.66 MHz (^13^C), respectively on a Bruker AVIII400 spectrometer. Chemical shifts δ are reported in ppm; for ^1^H rel. to (residuals non-deuterated) solvent signals (chloroform-*d*: 7.24, toluene-*d_8_*: 2.08 ppm), for ^13^C to CDCl_3_ at 77.00 ppm, or CD_3_C_6_D_5_ at 21.40 ppm, respectively. Coupling patterns are designated as s(inglet), d(oublet), t(riplet), q(uartet), m(ultiplet), p(seudo), and br(oad). ^13^C{^1^H}-NMR spectra are recorded in a *J*-modulated mode; signals are assigned as C, CH_2_, and CH_3_; undesignated signals refer to CH-resonances. MS: ESI or EI (ESI-Qq ao TOF mass spectrometer, Bruker, 70 eV). 

For photo rearrangements a mercury medium pressure lamp doped with FeI_2_ (Heraeus TQ718-Z4, 700 W) was used operating at >300 nm in ice cooled Pyrex glass vessels.

Hexane fraction (PE), dichloromethane (DCM), and ethyl acetate (EtOAc) were distilled, absolute THF from sodium benzophenone ketyl, toluene from LiAlH_4_, DCM and acetonitrile from CaH_2_; DIBAL was used as a 1.0 molar solution in toluene. All the other chemicals were analytical grade and used without further purification. Column chromatography was performed on SiO_2_, 40-63 µm. Reported procedures have been followed to obtain 3,3′-disubstituted 2,2′-dihydroxy-1,1′-binaphthyl precursors **1b**–**d** [48,49,50,51,52] and diallyloxy compound **2a** [26].

### 3.2. Syntheses and Rearrangements

#### 3.2.1. Typical Procedure: *2,2'-Bis(allyloxy)-3,3'-diiodo-1,1'-binaphthyl* (**2b**)

To a degassed solution of 2,2′-dihydroxy-3,3′-diiodo-1,1′-binaphthyl (**1b**, 269 mg, 0.5 mM) in acetone (50 mL) was added allylbromide (242 mg, 2.0 mM, 173 µL), pulverised K_2_CO_3_ (208 mg, 1.5 mM) and the mixture was refluxed for 24 h. The solids were filtered off and washed with DCM. After removal of solvents the product was purified by chromatography (DCM/PE, 25:75) to give 273 mg (91%) of **2b**; mp: 143–146 °C (DCM/PE). ^1^H-NMR (CDCl_3_) δ: 8.50 (s, 2H), 7.77 (dm, *J* = 8.3 Hz, 2H), 7.39 (ddd, *J* = 8.1, 6.9, 1.2 Hz, 2H), 7.25 (ddd, *J* = 8.3, 6.7, 1.3 Hz, 2H), 7.08 (dm, *J* = 8.6 Hz, 2H), 5.52 (ddpt, *J* = 17.1, 10.5, 5.6 Hz, 2H), 4.81 (m, 4H), 4.29 (ddpt, *J* = 11.9, 5.7, 1.4 Hz, 2H), 3.93 (ddpt, *J* = 11.9, 5.6, 1.4 Hz, 2H) ppm. ^13^C-NMR (CDCl_3_) δ: 153.90 (C), 139.74, 133.84 (C), 133.08, 132.15 (C), 127.03, 126.94, 125.88, 125.64 (C), 125.62, 117.28 (CH_2_), 92.70 (C), 74.42 (CH_2_) ppm. HRMS (ESI): calcd for C_26_H_20_I_2_O_2 _NH_4_: 635.9896, found: 635.9901.

*2,2'-Bis(allyloxy)-3,3'-dibromo-1,1'-binaphthyl* (**2c**). Yield: 89%; mp: 98–101 °C (DCM/PE). ^1^H-NMR (CDCl_3_) δ: 8.23 (s, 2H), 7.80 (dm, *J* = 8.5 Hz, 2H), 7.40 (ddd, *J* = 8.1, 6.6, 1.1 Hz, 2H), 7.25 (ddd, *J* = 8.4, 6.9, 1.3 Hz, 2H), 7.08 (dm, *J* = 8.6 Hz, 2H), 5.52 (ddpt, *J* = 17.0, 10.6, 5.6 Hz, 2H), 4.82 (m, 4H), 4.36 (ddpt, *J* = 11.9, 5.6, 1.4 Hz, 2H), 4.05 (ddpt, *J* = 11.9, 5.6, 1.3 Hz, 2H) ppm. ^13^C-NMR (CDCl_3_) δ: 151.95 (C), 133.16, 133.10 (C), 132.81, 131.42 (C), 127.11, 126.89 (C), 126.81, 125.92, 125.83, 117.76 (C), 117.17 (CH_2_), 74.46 (CH_2_) ppm. HRMS (ESI): calcd for C_26_H_20_Br_2_O_2_Na: 544.9728, found: 544.9732.

*2,2'-Bis(allyloxy)-3,3'-diphenyl-1,1'-binaphthyl* (**2d**). Yield: 90%; foam. ^1^H-NMR (CDCl_3_) δ: 7.94 (s, 2H), 7.89 (br d, *J* = 8.2 Hz, 2H), 7.73 (m, 4H), 7.33–7.46 (m, 8H), 7.24 (m, 4H), 5.27 (ddpt, *J* = 17.2, 10.4, 5.5 Hz, 2H), 4.64 (dm, *J* = 10.3 Hz, 2H), 4.54 (dpq, *J* = 17.1, 1.8 Hz, 2H), 3.94 (ddpt, *J* = 12.1, 5.4, 1.4 Hz, 2H), 3.70 (ddpt, *J* = 12.2, 5.6, 1.4 Hz, 2H) ppm. ^13^C-NMR (CDCl_3_) (One CH signal not detected) δ: 153.30 (C), 139.03 (C), 135.42 (C), 133.81, 133.67 (C), 130.77 (C), 130.25, 129.48, 128.23, 127.99, 127.25, 126.28 (C), 126.17, 125.98, 124.92, 116.17 (CH_2_), 73.73 (CH_2_) ppm. HRMS (ESI): calcd for C_38_H_30_O_2_Na: 541.2143, found: 541.2147.

#### 3.2.2. (15*E*,33*E*)-14,17,32,35-Tetrahydrotetranaphtho[2,1-b:1',2'-d:2'',1''-l:1''',2''' n][1,6,11,16]tetraoxa-cycloicosine (**3a**) and 2-Vinyl-2,3-dihydro-2'*H*-spiro[benzo[f]chromene-1,1'-naphthalen]-2'-one (**5a**)

Diallylether **2a** (37 mg, 0.1 mM) was dissolved in DCM (7 mL) and Grubbs II catalyst (8.5 mg, 10 mol%) in DCM (3 mL) was added under Ar at 40 °C during 6 h using a syringe pump. After removal of solvent the products were separated by chromatography (EtOAc/PE, 10:90 → 30:70) to give 12 mg (35%) of **5a** followed by 20 mg (59%) of **3a** (If the reaction was performed at r.t (10 h) **3a** and **5a** were isolated in 70% and 4%, respectively). Spectroscopic date are in agreement with reference [24].

*(15E,33E)-14,17,32,35-Tetrahydrotetranaphtho[2,1-b:1',2'-d:2'',1''-l:1''',2'''][1,6,11,16]tetraoxa-cycloicosine* (**3a**). White solid; ^1^H-NMR (CDCl_3_) δ: 7.92 (d, *J* = 8.2 Hz, 4H), 7.80 (d, *J* = 9.0 Hz, 4H), 7.36 (ddd, *J* = 7.8, 6.9, 0.8 Hz, 4H), 7.22 (ddd, *J* = 8.0, 6.7, 1.2 Hz, 4H), 7.12 (d, *J* = 8.8 Hz, 4H), 7.09 (d, *J* = 8.2 Hz, 4H), 5.54 (m, 4H), 4.39 (m, 8H) ppm. ^13^C-NMR (CDCl_3_) δ: 153.72 (C), 134.07 (C), 129.05, 129.02 (C), 127.87, 127.84, 126.15, 125.37, 123.37, 119.76 (C), 114.81, 68.42 (CH_2_) ppm. HRMS (ESI): calcd for C_48_H_36_O_4_Na: 699.2511, found: 699.2508.

*2-Vinyl-2,3-dihydro-2'H-spiro[benzo[f]chromene-1,1'-naphthalen]-2'-one* (**5a**). White solid; mp: 213–216 °C (EtOAc/PE). ^1^H-NMR (CDCl_3_) δ: 7.71 (d, *J* = 8.9 Hz, 1H), 7.66 (d, *J* = 8.1 Hz, 1H), 7.63 (d, *J* = 9.8 Hz, 1H), 7.41 (dd, *J* = 7.7, 1.2 Hz, 1H), 7.24 (ptd, *J* = 7.5, 1.2 Hz, 1H), 7.20 (d, *J* = 8.9 Hz, 1H), 7.13 (m, 2H), 7.01 (ddd, *J* = 8.5, 7.5, 1.4 Hz, 1H), 6.80 (d, *J* = 7.9 Hz, 1H), 6.65 (d, *J* = 8.4 Hz, 1H), 6.37 (d, *J* = 9.7 Hz, 1H), 5.52 (ddd, *J* = 17.0, 10.3, 8.8 Hz, 1H), 4.94 (dd, *J* = 10.3, 1.1 Hz, 1H), 4.66 (dpt, *J* = 17.0, 1.1 Hz, 1H), 4.56 (pt, *J* = 10.9 Hz, 1H), 4.12 (dd, *J* = 10.9, 3.4 Hz, 1H), 2.99 (m, 1H) ppm. ^13^C-NMR (CDCl_3_) δ: 201.46 (C), 154.99 (C), 147.66 (C), 145.38, 131.71 (C), 131.20 (br), 130.66, 130.41 (C), 130.24 (C), 129.89, 129.06, 128.57, 127.61, 126.95, 126.40, 126.31, 123.84, 123.00, 119.06, 118.49 (C), 115.79 (C), 63.05 (CH_2_), 56.96 (C), 54.44 ppm. HRMS (ESI): calcd for C_24_H_19_O_2_: 339.1385, found: 339.1381. (*R,R*)-**5a** was obtained similarly from (*R*)-**2a**; mp: 140–143 °C (DCM/PE), [α]_D_^20^ +457 (*c* 1.51, toluene).

#### 3.2.3. One Step Preparation of **5a** from **1a**

A degassed mixture of binaphthol **1a** (286 mg, 1 mmol) and Cs_2_CO_3_ (977 mg, 3 mmol) in dry acetone (20 mL) was heated to reflux and 1,4-dibromo-2-butene (214 mg, 1 mmol) in acetone (50 mL) was added over 30 min. After reflux overnight the solids were separated, washed with DCM and the filtrate evaporated. The residue was dissolved is DCM/water (30 mL/30 mL) and the aqueous phase separated and extracted with DCM (10 mL). The organic phases were dried and the residue subjected to chromatography (EtOAc/PE 10:90) to give 318 mg (94%) of **5a**.

#### 3.2.4. Photoisomerisation of **5a**

A solution of **5a** (252 mg, 0.746 mM) in toluene (10 mL) was irradiated (>300 nm) at 15–20 °C for 3 h. After removal of the solvent the mixture was subjected to chromatography (EtOAc/PE, 15:85) to yield 26 mg (10%) of **7a** and 168 mg (67%) of **6a**; 16 mg (6%) of starting material were recovered.

*(1R,10cR)-1,2,2a,2b,3,14b-Hexahydro-1,10c-methanobenzo[f]cyclobuta[3,4]naphtho[2,1-c]chromen-15-one* (**6a**). Mp: 164–165 °C (EtOAc/PE). ^1^H-NMR (CDCl_3_) δ: 7.79 (dm, *J* = 8.3 Hz, 1H), 7.78 (d, *J* = 8.9 Hz, 1H), 7.30 (m, 2H), 7.16-7.24 (m, 3H), 7.18 (d, *J* = 8.9 Hz, 1H), 6.97 (m, 1H), 6.51 (br d, *J* = 7.9 Hz, 1H), 4.41 (dd, *J* = 10.8, 3.5 Hz, 1H), 4.09 (br m, 1H), 4.08 (dd, *J* = 11.8, 11.1 Hz, 1H), 3.09 (dpt, *J* = 7.2, 5.0 Hz, 1H), 2.95 (dpt, *J* = 10.9, 5.7 Hz, 1H), 2.71 (m, 1H), 2.31 (m, 1H), 1.97 (d, *J* = 10.5 Hz, 1H) ppm. ^13^C-NMR (CDCl_3_) (one C(quart.) not detected) δ: 208.92 (C), 155.10 (C), 141.74 (C), 136.64 (C), 133.58 (C), 130.21, 129.96 (C), 128.83, 128.38, 126.76, 126.69, 125.81, 125.34, 124.55, 123.22, 119.41, 112.10 (C), 66.49 (CH_2_), 48.19, 45.77, 44.73, 37.44, 32.46 (CH_2_) ppm. HRMS (EI, 40 °C): calcd for C_24_H_18_O_2_: 338.1307, found: 338.1299.

*(6R,11bR)-4a,5,6,7-Tetrahydro-4H-5,7:6,11b-dimethanobenzo[3,4]cyclohepta[1,2-c]benzo[f]chromen-16-one* (**7a**). Mp: 248–249 °C (EtOAc/PE). ^1^H-NMR (CDCl_3_) δ: 7.75 (2 × d, *J* ~ 8 Hz, 2H), 7.24 (m, 1H), 7.17 (d, *J* = 8.8 Hz, 1H), 7.11–7.17 (m, 2H), 7.04–7.17 (m, 2H), 6.85 (ddd, *J* = 8.8, 6.7, 2.0 Hz, 1H), 6.32 (dd, *J* = 7.5, 1.0 Hz, 1H), 4.23 (dd, *J* = 10.8, 4.8 Hz, 1H), 3.99 (m, 1H), 3.37 (dd, *J* = 13.0, 11.0 Hz, 1H), 3.28 (ddd, *J* = 7.4, 5.8, 0.8 Hz, 1H), 3.11 (ddd, *J* = 11.0, 8.4, 7.8 Hz, 1H), 2.92 (ddd, *J* = 12.9, 4.8, 0.7 Hz, 1H), 2.49 (m, 1H), 1.78 (dm, *J* = 11.0 Hz, 1H) ppm. ^13^C-NMR (CDCl_3_) δ: 213.22 (C), 155.45 (C), 142.28 (C), 139.87 (C), 133.18 (C), 130.34 (C), 129.94, 128.85, 128.26, 127.69, 127.01, 126.77, 126.26, 124.52, 123.21, 118.89, 111.77 (C), 66.87 (CH_2_), 57.11 (C), 50.55, 44.53, 42.09, 37.17 (CH_2_), 32.37 ppm. HRMS (ESI): calcd for C_24_H_18_O_2_: 338.1307, found: 338.1301.

#### 3.2.5. Photoisomerisation of *epi*-**5a**

A solution of *epi-***5a** (18 mg in toluene-*d_8_*, 0.7 mL) was irradiated in a NMR tube for 0.5 h (300 nm). ^1^H-NMR showed complete conversion and the mixture was separated by chromatography (EtOAc/PE, 15:85) to give **11** (4 mg) and *epi*-**6a** (6 mg).

*(2aR,2bS)-1,2,2a,2b,3,14b-Hexahydro-1,10c-methanobenzo[f]cyclobuta[3,4]naphtho[2,1-c]chromen-15-one (epi-***6a**). Yield: 36%, mp: 230–234 °C (EtOAc/PE). ^1^H-NMR (CDCl_3_) δ: 7.78 (dd, *J* = 8.0, 1.3 Hz, 1H), 7.77 (d, *J* = 8.9 Hz, 1H), 7.36 (dm, *J* = 8.5 Hz, 1H), 7.31 (m, 1H), 7.28 (m, 1H), 7.25 (m, 1H), 7.23 (m, 1H), 7.10 (d, *J* = 8.8 Hz, 1H), 7.06 (ptd, *J* = 7.7, 1.6 Hz, 1H), 6.69 (dm, *J* = 7.8 Hz, 1H), 3.96 (dd, *J* = 10.4, 3.2 Hz, 1H), 3.94 (dd, *J* = 5.3, 5.0 Hz, 1H), 3.39 (dd, *J* = 12.0, 10.1 Hz, 1H), 2.99 (ddd, *J* = 7.0, 5.4, 4.7 Hz, 1H), 2.82 (ddd, *J* = 9.4, 6.9, 5.4 Hz, 1H), 2.77 (dd, *J* = 11.9, 3.0 Hz, 1H), 2.60 (pq, *J* = 5.3 Hz, 1H), 1.78 (d, *J* = 9.4 Hz, 1H) ppm. ^13^C-NMR (CDCl_3_) δ: 207.74 (C), 153.44 (C), 138.63 (C), 136.10 (C), 132.73 (C), 130.30, 129.40, 129.37 (C), 128.08, 127.90, 127.38, 127.35, 124.64, 124.56, 123.13, 119.07, 112.49 (C), 64.92 (CH_2_), 59.02 (C), 48.40, 46.01, 43.75, 40.49 (CH_2_), 38.48 ppm. HRMS (ESI): calcd for C_24_H_18_O_2_K: 377.0933, found: 377.0948.

*(8aS)-8a,9,13b,14-Tetrahydro-9,14-methanobenzo[f]cyclobuta[3,4]naphtho[2,3-c]chromen-15(8H)-one* (**11**)**. **Yield: 25%, mp: 245–248 °C, (EtOAc/PE). ^1^H-NMR (CDCl_3_) δ: 7.78 (br d, *J* = 8.0 Hz, 1H), 7.63 (br d, *J* = 8.5 Hz, 1H), 7.62 (br d, *J* = 8.8 Hz, 1H), 7.52 (ddd, *J* = 8.5, 6.9, 1.5 Hz, 1H), 7.37 (ddd, *J* = 8.0, 6.9, 1.1 Hz, 1H), 7.28 (m, 1H), 7.17–7.25 (m, 3H), 6.92 (d, *J* = 8.8 Hz, 1H), 4.69 (d, *J* = 5.7 Hz, 1H), 4.00 (dd, *J* = 10.9, 4.2 Hz, 1H), 3.40 (br dd, *J* = 8.2, 5.7 Hz, 1H), 3.31 (ptd, *J* = 4.0, 1.0 Hz, 1H), 2.91 (br dpt, *J* = 12.0, 4.0 Hz, 1H), 2.71 (dd, *J* = 12.9, 4.2 Hz, 1H), 2.59 (dd, *J* = 11.9, 10.9 Hz, 1H), 1.88 (ddd, *J* = 12.9, 8.2, 1.1 Hz, 1H) ppm. ^13^C-NMR (CDCl_3_) δ: 211.87 (C), 154.15 (C), 139.89 (C), 132.38 (C), 132.25 (C), 130.30, 130.21 (C), 129.50, 127.81, 127.37, 127.35, 126.32, 124.50, 124.11, 123.37, 119.09, 110.41 (C), 66.06 (CH_2_), 64.40 (C), 54.20, 45.26, 37.60, 36.62 (CH_2_), 35.65 ppm. HRMS (ESI): calcd for C_24_H_18_O_2_Na: 361.1204, found: 361.1194.

#### 3.2.6. 3',5-Diiodo-2-vinyl-2,3-dihydro-2'*H*-spiro[benzo[f]chromene-1,1'-naphthalen]-2'-one (**5b**)

To a degassed solution of **2b** (189 mg, 0.3 mM) in DCM (18 mL) was added Grubbs II catalyst (15 mg, 5 mol%) dissolved in DCM (2 mL) over 10 h at r.t. in the dark using a syringe pump. Removal of the solvent was followed by column chromatography (EtOAc/PE, 10:90; the column was wrapped with aluminium foil) to give 172 mg (95%) of **5b**.

*3',5-Diiodo-2-vinyl-2,3-dihydro-2'H-spiro[benzo[f]chromene-1,1'-naphthalen]-2'-one* (**5b**). Mp: 254–259 °C (EtOAc/PE). ^1^H-NMR (CDCl_3_) δ: 8.37 (s, 1H), 8.31 (s, 1H), 7.56 (dm, *J* = 8.1 Hz, 1H), 7.34 (dd, *J* = 7.7, 1.5 Hz, 1H), 7.24 (ptd, *J* = 7.5, 1.3 Hz, 1H), 7.15 (m, 1H), 7.13 (m, 1H), 7.02 (ddd, *J* = 8.5, 6.9, 1.5 Hz, 1H), 6.72 (dm, *J* = 7.9 Hz, 1H), 6.51 (dm, *J* = 8.6 Hz, 1H), 5.38 (ddd, *J* = 17.0, 10.3, 8.8 Hz, 1H), 5.01 (ddd, *J* = 10.3, 1.3, 0.5 Hz, 1H), 4.68 (dpt, *J* = 17.0, 1.1 Hz, 1H), 4.53 (dd, *J* = 11.0, 10.8 Hz, 1H), 4.20 (dd, *J* = 11.0, 3.4 Hz, 1H), 2.91 (m, 1H) ppm. ^13^C-NMR (CDCl_3_) δ: 54.43, 57.89 (C), 63.78 (CH_2_), 88.06 (C), 102.32 (C), 116.60 (C), 119.36 (CH_2_), 123.55, 123.82, 127.12, 127.40, 127.59, 127.68, 128.62, 130.23, 131.28, 131.36 (C), 131.42 (C), 131.54 (C), 140.10,147.29 (C), 152.24 (C), 154.40, 194.70 (C) ppm. HRMS (EI, 30 °C): calcd for C_24_H_16_I_2_O_2_: 589.9240, found: 589.9230.

*3',5-Dibromo-2-vinyl-2,3-dihydro-2'H-spiro[benzo[f]chromene-1,1'-naphthalen]-2'-one* (**5c**). Yield: 91%; pale yellow foam. ^1^H-NMR (CDCl_3_) δ: 8.08 (s, 1H), 8.05 (s, 1H), 7.59 (dm, *J* = 8.1 Hz, 1H), 7.37 (dd, *J* = 7.6, 1.4 Hz, 1H), 7.25 (ptd, *J* = 7.5, 1.3 Hz, 1H), 7.15 (m, 2H), 7.03 (ddd, *J* = 8.5, 6.9, 1.4 Hz, 1H), 6.72 (dm, *J* = 7.8 Hz, 1H), 6.54 (dm, *J*= 8.6 Hz, 1H), 5.42 (ddd, *J* = 17.0, 10.5, 8.9 Hz, 1H), 5.01 (dd, *J* = 10.4, 1.2 Hz, 1H), 4.69 (dpt, *J* = 17.0, 1.1 Hz, 1H), 4.56 (pt, *J* = 11.1 Hz, 1H), 4.23 (dd, *J* = 11.1, 3.4 Hz, 1H), 2.99 (ddd, *J* = 11.7, 8.9, 3.5 Hz, 1H) ppm. ^13^C-NMR (CDCl_3_) δ: 193.67 (C), 151.05 (C), 146.93, 146.67 (C), 133.15, 131.13, 130.62 (C), 130.47 (C), 130.29 (C), 129.89, 128.84, 127.85, 127.56 (2} CH), 126.97, 124.04, 123.53, 122.70 (C), 119.60 (CH_2_), 117.37 (C), 113.25 (C), 63.57 (CH_2_), 58.72 (C), 54.34 ppm. HRMS (ESI): calcd for C_24_H_16_Br_2_O_2_Na: 516.9415, found: 516.9415.

*3',5-Diphenyl-2-vinyl-2,3-dihydro-2'H-spiro[benzo[f]chromene-1,1'-naphthalen]-2'-one* (**5**). Yield: 98%; pale yellow solid. ^1^H-NMR (CDCl_3_) δ: 7.73 (2 × s, 2H), 7.69 (m, 1H), 7.68 (m, 2H), 7.54 (m, 2H), 7.44–7.50 (m, 3H), 7.31-7.41 (m, 4H), 7.27 (ptd, *J* = 7.5, 1.3 Hz, 1H), 7.15 (m, 2H), 7.02 (ddd, *J* = 8.5, 6.9, 1.5 Hz, 1H), 6.88 (dm, *J* = 7.7 Hz, 1H), 6.78 (dm, *J* = 8.6 Hz, 1H), 5.60 (ddd, *J* = 17.1, 10.4, 8.8 Hz, 1H), 4.96 (ddd, *J* = 10.3, 1.4, 0.5 Hz, 1H), 4.71 (dm, *J* = 17.1 Hz, 1H), 4,54 (dd, *J* = 10.2, 10.0 Hz, 1H), 4.13 (dd, *J* = 10.8, 3.3 Hz, 1H), 3.05 (ddd, *J* = 9.9, 8.9, 3.3 Hz, 1H) ppm. ^13^C-NMR (CDCl_3_) δ: 199.83 (C), 152.82 (C), 147.56 (C), 142.90, 138.45 (C), 136.47 (C), 135.51 (C), 131.92 (C), 131.56, 131.36 (C), 130.71 (C), 130.59, 130.33, 129.94, 129.88 (C), 129.35, 128.90, 128.73, 128.20, 128.14, 128.01, 127.51, 127.26, 127.12, 126.30, 123.82, 123.39, 118.32 (C), 116.69 (C), 63.16 (CH_2_), 57.79 (C), 54.13 ppm. HRMS (ESI): calcd for C_36_H_26_O_2_Na: 513.1830, found: 513.1847.

#### 3.2.7. Photoisomerisation of **5b** (Typical Procedure)

A sample containing ca.15 mg of **5b** in toluene-*d_8_* in a NMR tube was irradiated for a total of 10 h (60 W, light bulb) after which ^1^H-NMR indicated absence of **5b** and formation of **6b** and **7b** (ca. 9:1), separable by chromatography (EtOAc/PE, 10:90).

*(1R,10cR)-1,5-Diiodo-1,2,2a,2b,3,14b-hexahydro-1,10c-methanobenzo[f]cyclobuta[3,4]naphtho[2,1-c]chromen-15-one* (**6b**). Yield: 75%; mp: 248–249 °C (EtOAc/PE). ^1^H-NMR (CDCl_3_) δ: 8.35 (s, 1H), 7.64 (dm, *J* = 8.0 Hz, 1H), 7.32 (dd, *J* = 7.4, 1.4 Hz, 1H), 7.26 (ddd, *J* = 8.0, 6.8, 1.2 Hz, 1H), 7.21 (ptd, *J* = 7.4, 1.2 Hz, 1H), 7.17 (ddd, *J* = 8.3, 6.8, 1.5 Hz, 1H), 6.98 (m, 2H), 6.38 (br d, *J* = 7.9 Hz, 1H), 4.46 (br d, *J* = 6.8 Hz, 1H), 4.45 (dd, *J* = 11.0, 3.5 Hz, 1H), 3.97 (dd, *J* = 11.8, 11.1 Hz, 1H), 3.11 (m, 1H), 2.95 (ddd, *J* = 10.8, 7.1, 1.4 Hz, 1H), 2.46 (d, *J* = 10.7 Hz, 1H), 2.26 (m, 1H) ppm. ^13^C-NMR (CDCl_3_) δ: 201.23 (C), 152.37 (C), 140.48, 139.98 (C), 133.56 (C), 133.10 (C), 131.23 (C), 128.58, 127.79, 127.57, 127.40, 126.48, 125.88, 125.46, 124.14, 112.46 (C), 87.90 (C), 65.72 (CH_2_), 58.16 (C), 56.58, 44.10, 43.33 (CH_2_), 39.88, 35.86 (C) ppm. HRMS (EI, 30 °C): calcd for C_24_H_16_I_2_O_2_: 589.9240, found: 589.9231.

*(6R,11bR)-2,6-Diiodo-4a,5,6,7-tetrahydro-4H-5,7:6,11b-dimethanobenzo[3,4]cyclohepta[1,2-c]benzo[f]chromen-16-one* (**7b**). Yield: 12%, mp: 151–152 °C (EtOAc/PE). ^1^H-NMR (CDCl_3_) δ: 8.37 (s, 1H), 7.65 (dm, *J* = 8.1 Hz, 1H), 7.26 (ddd, *J* = 8.0, 6.8, 1.1 Hz, 1H), 7.17 (ptd, *J* = 7.5, 1.2 Hz, 1H), 7.08 (m, 1H), 7.07 (m, 1H), 6.96 (dm, *J* = 8.8 Hz, 1H), 6.87 (m, 1H), 6.29 (dd, *J* = 8.0, 1.2 Hz, 1H), 4.36 (dd, *J* = 11.2, 4.9 Hz, 1H), 4.16 (ddd, *J* = 8.5, 4.9, 0.9 Hz, 1H), 3.17 (dpt, *J* = 11.1, 8.1 Hz, 1H), 3.45 (dd, *J* = 13.0, 11.1 Hz, 1H), 3.06 (ddd, *J* = 13.0, 4.8, 0.6 Hz, 1H), 2.57 (ddm, *J* = 7.8, 4.8 Hz, 1H), 1.73 (dm, *J* = 11.1 Hz, 1H) ppm. ^13^C-NMR (CDCl_3_) δ: 205.34 (C), 152.83 (C), 140.54 (C), 140.21, 138.76 (C), 132.85 (C), 131.65 (C), 128.88, 128.28, 127.27, 127.15, 127.03, 126.37, 125.17, 124.13, 112.34 (C), 87.37 (C), 67.42 (CH_2_), 58.20, 54.34 (C), 51.19, 44.44, 36.82 (C), 35.72 (CH_2_) ppm. HRMS (ESI): calcd for C_24_H_16_I_2_O_2_Na: 612.9137, found: 612.9135.

*(1R,10cR)-1,5-Dibromo-1,2,2a,2b,3,14b-hexahydro-1,10c-methanobenzo[f]cyclobuta[3,4]naphtho[2,1-c]chromen-15-one* (**6c**). Yield: 64%, crystalline precipitate, mp: 324–328 °C (EtOAc/PE, dec.).^1^H-NMR (CDCl_3_) δ: 8.14 (s, 1H), 7.72 (dm, *J* = 8.2 Hz, 1H), 7.39 (dd, *J* = 7.4, 1.3 Hz, 1H), 7.33 (ddd, *J* = 8.0, 6.8, 1.0 Hz, 1H), 7.27 (ptd, *J* = 7.6, 1.3 Hz, 1H), 7.22 (ddd, *J* = 8.3, 6.8, 1.4 Hz, 1H), 7.07 (dm, *J* = 8.8 Hz, 1H), 7.03 (ptd, *J* = 7.5, 1.4 Hz, 1H), 6.44 (br d, *J* = 7.8 Hz, 1H), 4.55 (dd, *J* = 11.0, 3.4 Hz, 1H), 4.40 (d, *J* = 6.9 Hz, 1H), 4.03 (dd, *J* = 11.9, 11.1 Hz, 1H), 3.20 (m, 1H), 2.97 (ddd, *J* = 10.8, 7.2, 1.5 Hz, 1H), 2.49 (d, *J* = 10.8 Hz, 1H), 2.31 (dm, *J* = 12.1 Hz, 1H) ppm. ^13^C-NMR (CDCl_3_) δ: 200.42 (C), 150.97 (C), 139.77 (C), 133.58, 133.40 (C), 132.42 (C), 130.15 (C), 128.67, 127.84, 127.64, 127.54, 126.55, 125.91, 125.26, 124.39, 113.28 (C), 113.22 (C), 65.71 (CH_2_), 58.80 (C), 57.38 (C), 54.93, 44.27, 41.55 (CH_2_), 35.85 ppm. HRMS (ESI): calcd for C_24_H_16_Br_2_NaO_2_: 516.9415, found: 516.9425.

*(6R,11bR)-2,6-dibromo-4a,5,6,7-tetrahydro-4H-5,7:6,11b-dimethanobenzo[3,4]cyclohepta[1,2-c]benzo[f]chromen-16-one* (**7c**). Yield: 10%, mp: 240–246 °C (EtOAc/PE), colorless prisms. ^1^H-NMR (CDCl_3_) δ: 8.11 (s, 1H), 7.68 (br d, *J* = 8.3 Hz, 1H), 7.28 (m, 1H), 7.18 (ptd, *J* = 7.5, 1.5 Hz, 1H), 7.10 (br d, *J* = 7.4 Hz, 1H), 7.09 (m, 1H), 6.96 (d, *J* = 8.6 Hz, 1H), 6.88 (ptd, *J* = 7.6, 1.4 Hz, 1H), 6.27 (br d, *J* = 8.0 Hz, 1H), 4.39 (dd, *J* = 11.1, 4.8 Hz, 1H), 4.16 (dd, *J* = 8.1, 5.1 Hz, 1H), 3.57 (dpt, *J* = 11.2, 8.2 Hz, 1H), 3.47 (dd, *J* = 12.9, 11.1 Hz, 1H), 3.01 (dd, *J* = 13.0, 4.9 Hz, 1H), 2.58 (dd, *J* = 7.8, 5.1 Hz, 1H), 1.81 (d, *J* = 11.2 Hz, 1H) ppm. ^13^C-NMR (CDCl_3_) δ: 203.99 (C), 151.50 (C), 140.24 (C), 138.69 (C), 133.27, 132.10 (C), 130.53 (C), 128.74, 128.38, 127.42, 127.09, 127.06, 126.49, 125.04, 124.37, 113.24 (C), 112.80 (C), 67.17 (CH_2_), 58.83 (C), 56.07, 55.87 (C), 50.01, 42.10, 35.68 (CH_2_) ppm. HRMS (ESI): calcd for C_24_H_16_Br_2_NaO_2_: 518.9394, found: 518.9372.

*(1R,10cR)-1,5-Diphenyl-1,2,2a,2b,3,14b-hexahydro-1,10c-methanobenzo[f]cyclobuta[3,4]naphtho[2,1-c]chromen-15-one* (**6d**). Yield: 7%, mp: 322–327 °C (EtOAc/PE), colorless prisms. ^1^H-NMR (CDCl_3_) δ: 7.79 (s, 1H), 7.78 (d, *J* = 8.4 Hz, 1H), 7.61 (m, 2H), 7.48 (dd, *J* = 7.5, 1.2 Hz, 1H), 7.44 (m, 2H), 7.36 (m, 1H), 7.21–7.33 (m, 5H), 7.11 (m, 2H), 7.10 (m, 1H), 7.07 (ptd, *J* = 7.7, 1.4 Hz, 1H), 7.00 (d, *J* = 8.6 Hz, 1H), 6.61 (d, *J* = 7.8 Hz, 1H), 4.46 (dd, *J* = 11.1, 3.6 Hz, 1H), 4.24 (d, *J* = 6.2 Hz, 1H), 4.14 (dd, *J* = 11.6, 11.0 Hz, 1H), 3.00 (pq, *J* = 6.0 Hz, 1H), 2.81 (ddd, *J* = 10.3, 6.4, 1.7 Hz, 1H), 2.41 (m, 1H), 2.40 (d, *J* = 10.3 Hz, 1H) ppm. ^13^C-NMR (CDCl_3_) δ: 207.29 (C), 152.92 (C), 141.15 (C), 140.09 (C), 138.65 (C), 136.65 (C), 132.97 (C), 132.35 (C), 131.00, 129.86, 129.52 (C), 128.47, 128.40, 128.30, 127.96, 127.17, 127.14, 127.05, 127.02, 126.35, 126.07, 125.68, 124.69, 123.43, 112.72 (C), 65.44 (CH_2_), 58.76 (C), 57.88 (C), 49.80, 46.07, 36.89 (CH_2_), 34.08 ppm. HRMS (ESI): calcd for C_36_H_26_NaO_2_: 513.1825, found: 513.1824.

*(6R,11bR)-2,6-Diphenyl-4a,5,6,7-tetrahydro-4H-5,7:6,11b-dimethanobenzo[3,4]cyclohepta[1,2-c]benzo[f]chromen-16-one* (**7d**). Yield: 39%, oil. ^1^H-NMR (CDCl_3_) δ: 7.82 (s, 1H), 7.80 (br d, *J* = 8.3 Hz, 1H), 7.65 (m, 2H), 7.41–7.52 (m, 7H), 7.38 (m, 1H), 7.29 (m, 1H), 7.26 (m, 2H), 7.11 (m, 2H), 6.98 (m, 1H), 6.51 (d, *J* = 7.8 Hz, 1H), 4.39 (dd, *J* = 10.9, 4.9 Hz, 1H), 4.26 (dd, *J* = 8.4, 4.7 Hz, 1H), 3.54 (dd, *J* = 13.1, 10.9 Hz, 1H), 3.22 (dpt, *J* = 10.9, 8.3 Hz, 1H), 3.08 (dd, *J* = 12.9, 4.9 Hz, 1H), 2.82 (dd, *J* = 7.9, 4.7 Hz, 1H), 1.90 (d, *J* = 10.9 Hz, 1H) ppm. ^13^C-NMR (CDCl_3_) δ: 211.38 (C), 153.10 (C), 142.06 (C), 139.75 (C), 138.48 (C), 136.54 (C), 132.67 (C), 131.86 (C), 130.76, 129.94 (C), 129.84, 129.01, 128.53, 128.19, 128.00, 127.87, 127.82, 127.20 (2 × CH), 126.69, 126.47, 124.37, 123.50, 112.62 (C), 66.82 (CH_2_), 58.87 (C), 53.90 (C), 51.00, 48.95, 38.67, 35.91 (CH_2_) ppm. HRMS (ESI): calcd for C_36_H_26_NaO_2_: 513.1825, found: 513.1824.

#### 3.2.8. Synthesis of **6b**/**7b** from **2b** (One Pot Procedure)

Grubbs II catalyst (12.6 mg, 0.015 mM) dissolved in toluene (1.5 mL) was slowly added during 6 h to a stirred solution of **2b** (145 mg, 0.235 mM) in toluene (14 mL) at r.t. During this time the reaction mixture was irradiated using a desk lamp. Extractive work-up and chromatographic purification afforded 103 mg (76%) of **6b** and 17 mg (13%) of **7b**.

*(2E,2'E)-Dimethyl 4,4'-(R)-1,1'-binaphthyl-2,2'-diylbis(oxy)dibut-2-enoate* [(*R*)**-8**]. (*R*)-2,2'-Dihydroxy-1,1'-binaphthyl **1a **(859 mg, 3 mM) was dissolved in acetonitrile (25 mL). After addition of methyl 4-bromocrotonate (1.61 g, 9 mM) and K_2_CO_3_ (1.26 g, 9 mM) the mixture was stirred at r.t. under Ar for 24 h. Standard work-up with water/DCM and drying (MgSO_4_) was followed by column chromatography (EtOAc/PE, 30:70) to yield 1.07 g (74%) of (*R*)**-8**; oil; [α]_D_^20^ +30.6 (*c* 1.00, CHCl_3_). ^1^H-NMR (CDCl_3_) δ: 7.94 (d, *J* = 9.0 Hz, 2H), 7.86 (br d, *J* = 8.2 Hz, 2H), 7.33 (ddd, *J* = 8.0, 6.5, 1.4 Hz, 2H), 7.32 (d, *J* = 9.0 Hz, 2H), 7.22 (ddd, *J* = 8.2, 6.7, 1.4 Hz, 2H), 7.14 (dm, *J* = 8.6 Hz, 2H), 6.81 (dpt, *J* = 15.7, 3.9 Hz, 2H), 5.63 (dpt, *J* = 15.8, 2.2 Hz, 2H), 4.66 (m, 4H), 3.63 (s, 6H) ppm. ^13^C-NMR (CDCl_3_) δ: 166.44 (C), 153.33 (C), 143.07, 134.02 (C), 129.62, 129.53 (C), 127.99, 126.56, 125.32, 123.94, 121.09, 120.17 (C), 115.01, 67.79 (CH_2_), 51.45 (CH_3_) ppm. HRMS (ESI): calcd for C_30_H_26_O_6_Na: 505.1627, found: 505.1616.

*(2E,2'E)-4,4'-(R)-1,1'-Binaphthyl-2,2'-diylbis(oxy)dibut-2-en-1-ol* [(*R*)**-9**]. To a degassed solution of (*R*)-**8** (1.07 g, 2.22 mM) in abs. DCM (30 mL) was added at −78 °C DIBAL (1 mol solution in toluene, 8.9 mL, 8.9 mM) and the reaction was kept at −20 °C for 12 h after which time TLC (EtOAc, 100%) indicated complete conversion. A saturated solution of potassium sodium tartrate (30 mL) and glycol (0.5 mL) was added and the mixture was stirred at r.t. for 3 h. The organic phase was separated and the aqueous phase was extracted with DCM (10 mL). The combined organic extracts were washed with brine and dried (Na_2_SO_4_). After evaporation the crude product was purified by chromatography (EtOAc / PE 50:50 → 100:0) to give 672 mg (71%) of (*R*)-**9**; white solid; mp: 38–40 °C; [α]_D_^20^ +65.2 (*c* 1.00, CHCl_3_). ^1^H-NMR (CDCl_3_) δ: 7.91 (d, *J* = 9.1 Hz, 2H), 7.84 (d, *J* = 8.1 Hz, 2H), 7.37 (d, *J* = 9.1 Hz, 2H), 7.30 (ddd, *J* = 8.0, 6.7, 1.2 Hz, 2H), 7.19 (ddd, *J* = 8.4, 6.7, 1.3 Hz, 2H), 7.11 (br d, *J* = 8.4 Hz, 2H), 5.55 (m, 4H), 4.50 (m, 4H), 3.90 (m, 4H), 1.73 (br s, 2H) ppm. ^13^C-NMR (CDCl_3_) δ: 153.93 (C), 134.13 (C), 131.61, 129.39 (C), 129.20, 127.86, 126.72, 126.23, 125.48, 123.68, 120.66 (C), 115.99, 69.25 (CH_2_), 62.71 (CH_2_) ppm. HRMS (ESI) calcd for C_28_H_26_O_4_Na: 449.1729, found: 449.1727.

*(2E,2'E)-4,4'-(R)-1,1'-Binaphthyl-2,2'-diylbis(oxy)dibut-2-en-1-ylbromide* [(*R*)*-***10**]. To a solution of (*R*)-**9** (580 mg, 1.36 mM) in dry THF (15 mL) was dropwise added at −40 °C PBr_3_ (810 mg, 3.06 mM, 2.2 equiv, 284 µL) in THF (1 mL) and the mixture was slowly warmed up overnight. Sat. sodium bicarbonate solution (5 mL) was added, followed by water (10 mL) and DCM (20 mL). The aqueous layer was extracted with DCM (2 × 15 mL) and the combined extracts were washed with brine and dried (MgSO_4_). After evaporation the crude bromide was purified by chromatography (DCM/PE, 30:70) to give 544 mg (72%) of (*R*)**-10** as a colorless oil; [α]_D_^20^ +13.7 (*c* 1.00, THF). ^1^H-NMR (CDCl_3_) δ: 7.94 (d, *J* = 9.0 Hz, 2H), 7.86 (d, *J* = 8.2 Hz, 2H), 7.37 (d, *J* = 9.9 Hz, 2H), 7.32 (m, 2H), 7.22 (m, 2H), 7.14 (d, *J* = 8.4 Hz, 2H), 5.60 (m, 4H), 4.53 (m, 4H), 3.74 (m, 4H) ppm. ^13^C-NMR (CDCl_3_) δ: 153.76 (C), 133.99 (C), 130.56, 129.42 (C), 129.35, 127.92 (2 × CH), 126.33, 125.43, 123.76, 120.44 (C), 117.71, 68.57 (CH_2_), 31.96 (CH_2_) ppm. HRMS (ESI) calcd for C_28_H_24_^79^Br^81^BrO_2_Na: 575.0023, found: 575.0045.

*Macrocyclisation of (R)-***10*** with (R)-***1**. To a degassed solution of (*R*)-binaphthol **1** (57 mg, 0.2 mM) in THF (7 mL) was added KOH (22 mg, 0.4 mM, 0.4 mL of a 1 N aqueous solution) and the mixture was refluxed for 30 min. Dibromide (*R*)*-***10** (110 mg, 0.2 mM) dissolved in THF (3 mL) was added and the reaction was refluxed for 3 d. Extractive work-up with DCM/water and chromatographic purification (EtOAc / PE, 20:80) yielded 95 mg (70%) of (*R,R*)-**3a**; mp: 159–160 °C (EtOAc/PE); [α]_D_^20^ +110 (*c* 0.42, CHCl_3_). Spectroscopic data fully agreed with the racemic compound.

*Palladium mediated cleavage of* *(R,R)-***3a**. Macrocycle (*R,R*)*-***3a** (80 mg, 0.12 mmol) and Pd(PPh_3_)_4_ (14 mg, 0.012 mmol) were heated in toluene (60 °C) for 48 h. The crude mixture was separated by column chromatography (EtOAc/PE, 10:90→20:80) to give a 44 mg fraction consisting of (*R,R*)-**5a** (36 mg, 45%) and **6a** (8 mg, 10%) followed by 19 mg (24%) of (*R,S*)-*epi*-**5a**; mp: 153–155 °C (EtOAc/PE). ^1^H-NMR (CDCl_3_) δ: 7.70 (d, *J* = 9.3 Hz, 1H), 7.68 (d, *J* = 8.0 Hz, 1H), 7.61 (d, *J* = 9.9 Hz, 1H), 7.44 (dd, *J* = 7.5, 1.4 Hz, 1H), 7.29 (ptd, *J* = 7.3, 1.2 Hz, 1H), 7.18 (ptd, *J* = 7.7, 1.4 Hz, 1H), 7.17 (d, *J* = 9.1 Hz, 1H), 7.15 (ddd, *J* = 8.1, 6.9, 1.2 Hz, 1H), 7.07 (br d, *J* = 7.6 Hz, 1H), 7.07 (ddd, *J* = 8.4, 6.9, 1.4 Hz, 1H), 6.62 (dm, *J* = 8.5 Hz, 1H), 6.41 (d, *J* = 10.0 Hz, 1H), 5.00 (m, 2H), 4.94 (m, 1H), 4.24 (pt, *J* = 11.3 Hz, 1H), 4.07 (dd, *J* = 11.3, 4.0 Hz, 1H), 3.11 (m, 1H) ppm. ^13^C-NMR (CDCl_3_) δ: 203.10 (C), 154.17 (C), 145.01, 143.65 (C), 131.54 (C), 131.00, 130.70 (C), 130.25 (C), 130.01, 129.60, 129.44 (2 × CH), 128.74, 127.48, 126.51, 126.46, 123.86, 123.11, 119.75 (CH_2_), 118.86, 115.35 (C), 64.09 (CH_2_), 57.18 (C), 51.34 ppm. HRMS (ESI): calcd for C_24_H_18_O_2_Na: 361.1207, found: 361.1204.

### 3.3. Crystallographic Structure Determination

X-ray diffraction measurements were performed on an X8 APEX II CCD diffractometer at 100 or 150 K. Single crystals were positioned at 50, 35, 35, 35, 45 and 35 mm from the detector and 1270, 1831, 2488, 1406, 1467 and 1278 frames were measured, each for 60, 10, 30, 5, 10 and 10 s over 1° scan width for *epi***-5a**, **5b**, **6a**, *epi*-**6a**, **6b** and **11**, respectively. The data were processed using SAINT software [53]. Crystal data, data collection parameters, and structure refinement details are given in Table 2. The structures were solved by direct methods and refined by full-matrix least-squares techniques. Non-hydrogen atoms were refined with anisotropic displacement parameters. H atoms were placed at calculated positions and refined as riding atoms in the subsequent least squares model refinements. The isotropic thermal parameters were estimated to be 1.2 times the values of the equivalent isotropic thermal parameters of the non-hydrogen atoms to which hydrogen atoms are bonded. The following computer programs were used: structure solution, SHELXS-97 refinement, SHELXL-97 [54] molecular diagrams, ORTEP [55] computer: Pentium IV. CCDC 907285−907290 contain the supplementary crystallographic data for this paper. These data can be obtained free of charge from the Cambridge Crystallographic Data Centre via www.ccdc.cam.ac.uk/data_request/cif.

**Table 2:** Crystal data and details of data collection for *epi*-**5a, 5b, 6a,***epi*-**6a, 6b** and **11.**

Compound	*epi*-5a	5b	6a	*epi*-6a	6b	11
formula	C_24_H_18_O_2_	C_24_H_16_I_2_O_2_	C_24_H_18_O_2_	C_24_H_18_O_2_	C_73.88_H_52.25_I_6_O_7.13_	C_24_H_18_O_2_
Fw	338.38	590.17	338.38	338.38	1815.31	338.38
space group	*P*2_1_2_1_2	*P*-1	*P*2_1_2_1_2_1_	*P*2_1_	*C*2/*c*	*P*2_1_2_1_2_1_
*a* [Å]	40.275(2)	8.9274(6)	9.3461(5)	11.9996(7)	28.1310(16)	7.9726(2)
*b* [Å]	14.7521(9)	14.7039(14)	10.0950(6)	9.7117(6)	11.8338(8)	11.6491(4)
*c* [Å]	8.8731(5)	15.9680(16)	18.2172(11)	15.2652(9)	37.359(2)	17.7858(6)
*α* [°]		90.792(4)				
*β* [°]		105.289(3)		108.528(3)	95.219(6)	
*γ* [°]		90.064(3)				
*V* [Å^3^]	5271.9(5)	2021.7(3)	1718.77(17)	1686.75(17)	12385.1(13)	1651.84(9)
*Z*	12	4	4	4	8	4
*λ* [Å]	0.71073	0.70713	0.71073	0.71073	0.71073	0.71073
*ρ*_calcd _[g cm^−3^]	1.279	1.939	1.308	1.333	1.947	1.361
*T* [K]	100(2)	100(2)	100(2)	150(2)	100(2)	150(2)
*μ* [mm^−1^]	0.080	3.128	0.082	0.084	3.068	0.085
*R*_1_^a^	0.0565	0.0628	0.0294	0.0431	0.0483	0.0384
*wR*_2_^b^	0.1402	0.1592	0.0781	0.1011	0.0960	0.1032
GOF^c^	1.054	1.191	1.034	1.045	1.083	1.049

^a^ *R*_1_ = Σ||*F*_o_| − |*F*_c_||/Σ|*F*_o_|. ^b^ *wR*_2_ = {Σ[*w*(*F*_o_^2^ − *F*_c_^2^)^2^]/Σ[*w*(*F*_o_^2^)^2^]}^1/2^. ^c^ GOF = {Σ[*w*(*F*_o_^2^ − *F*_c_^2^)^2^]/(*n* − *p*)}^1/2^, where *n* is the number of reflections and *p* is the total number of parameters refined.

### 3.4. Calculations

All calculations were performed using software packages SPARTAN (B3LYP, MO6 and MP2) and the Gaussian09 on the Phoenix Linux Cluster of the Vienna University of Technology [38]. The geometry and energy of the ruthenium model compounds and the transition states were optimized at the B3LYP level [56,57,58] with the Stuttgart/Dresden ECP (SDD) basis set to describe the electrons of the ruthenium atom [59,60,61], and a standard 6-31g** basis set was employed for all other atoms [62,63,64,65,66,67,68]. All geometries were optimized without symmetry constraints. Frequency calculations were performed to confirm the nature of the stationary points, yielding one imaginary frequency for the transition states and none for the minima. Each transition state was further confirmed by following its vibrational mode downhill on both sides and obtaining the minima presented on the energy profiles. All energies reported are Gibbs free energies and thus contain zero-point, thermal, and entropy effects at 298 K and 1 atm pressure. The solvation energies were calculated on the geometries from B3LYP gas phase optimizations via the polarizable continuum model (PCM) [69,70] with the radii and nonelectrostatic terms based on Truhlar and co-workers’ solute electron density (SMD) solvation model [71] with solvation parameters corresponding to CH_2_Cl_2_.

## 4. Conclusions

Summarising, we have developed an operationally simple one-pot procedure for transforming *O,O'*-diallyl substituted axial chiral binaphthols into centro-chiral species through a RCM followed by a Claisen-type rearrangement of reactive (*E*)-dioxacyclodecine intermediates **4a**–**d**. This mechanistic assumption was supported and rationalized by DFT calculations performed on *E***-4a**, corresponding precursors and hypothetical intermediates. Under irradiation further rearrangement took place yielding strained cage compounds **6a**–**d** and **7a**–**d** through a formal [2+2] cycloaddition.

## Supplementary Materials

Supplementary File 1
