# Supplementary material for: Tandem RCM–Claisen Rearrangement–[2+2] Cycloaddition of O,O'-(But-2-en-1,4-diyl)-bridged Binaphthols
Source: Molecules. 2012 Dec 7;17(12):14531–54. doi: 10.3390/molecules171214531 (PMC6268543; doi:10.3390/molecules171214531)
Supplement: Supplementary File 1 [file molecules-17-14531-s001.pdf]

Supporting Information

Tandem RCM–Claisen Rearrangement–[2+2] Cycloaddition of *O,O'*-(but-2-en-1,4-Diyl) Bridged Binaphthols

Michael Abraham [1], Wolfgang Reischl [1], Karl A. Kirchner [2], Alexander Roller [3], Luis F. Veiros [4] and Michael Widhalm [1,*]

[1] Faculty of Chemistry, Institute of Organic Chemistry, University of Vienna, Währinger Straße 38, A-1090 Wien, Austria

[2] Institute of Applied Synthetic Chemistry, Vienna University of Technology, Getreidemarkt 9/163/AC, A-1060 Wien, Austria

[3] Faculty of Chemistry, Institute of Inorganic Chemistry, University of Vienna, Währinger Straße 42, A-1090 Wien, Austria

[4] Centro de Química Estrutural, Instituto Superior Técnico, Universidade Técnica de Lisboa, 1049-001 Lisboa, Portugal

Content

Proposed mechanism for the formation of **5a** and *epi*-**5a** from **3a** S2

Assignment of ^{1}H and ^{13}C-shifts of **5a**–**d**, **6a**–**d**, **7a**–**d**, *epi*-**5a**, *epi*-**6a** and **11** S3

^{1}H- and ^{13}C-NMR spectra.. S11

Calculations (schemes)...S56

Calculations (energies and coordinates)..S58

Suggested Mechanism for the Concomitant Formation of 5a and *epi*-5a from 3a Promoted by $Pd(PPh_3)_4$

A Pd(0) species attacks macrocycle **3a**, highly susceptible due to the presence of four Ar-O-allyl units, forming a tetracoordinated Pd(II) complex *X2* with π–allyl and enolether ligands, eventually via a Pd_2 intermediate *X1*. From Pd-σ-allyl complex *X4* **5a** will be formed by reductive elimination. The appearance of *epi*-**5a** can be rationalized through the involvement of (conformativly labile) σ-allyl complexes *X5* and *X6* with *endo* double bond. To test for reversible steps a sample of **5a** was heated in toluene-d_8 with a catalytic amount of $Pd(PPh_3)_4$ for 12 h (80 °C). The NMR spectrum obtained was completely superimposable with the original one, indicating configurational stability of **5a** and the equilibration process responsible for the formation of *epi*-**5a** can take place only at the stage of Pd intermediates like *X4*-*X7*.

Scheme 1. Proposed mechanism for the Pd-mediated cleavage of **3a**.

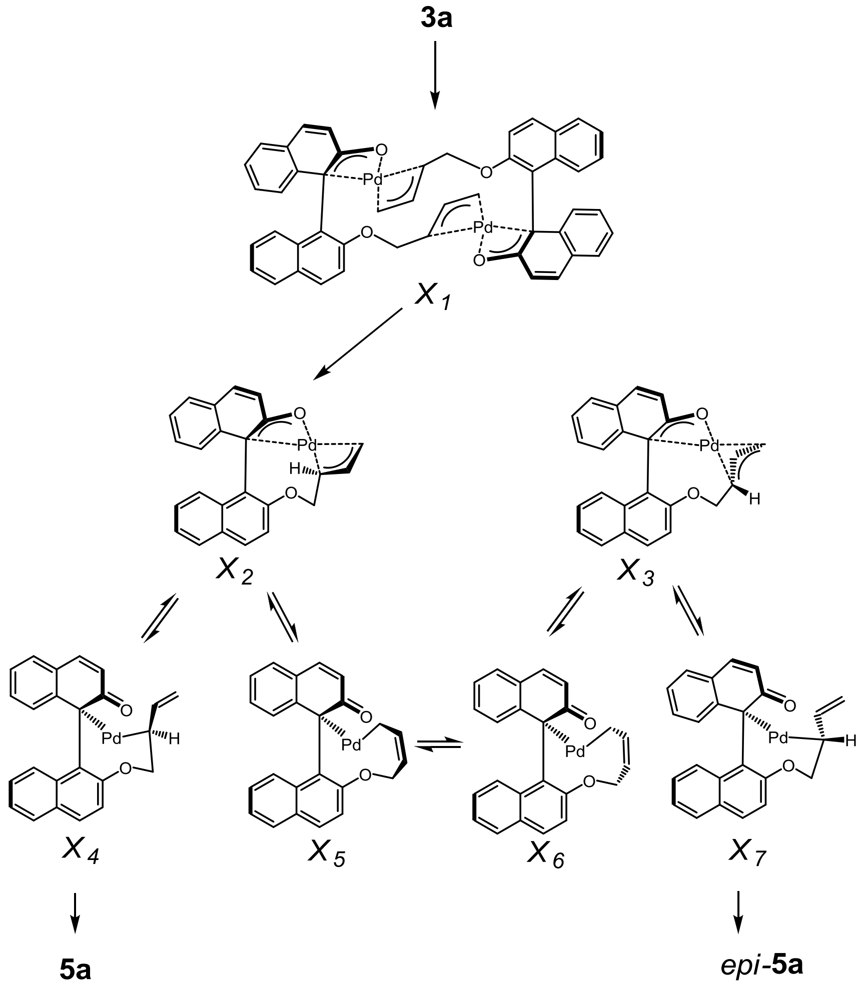

5a

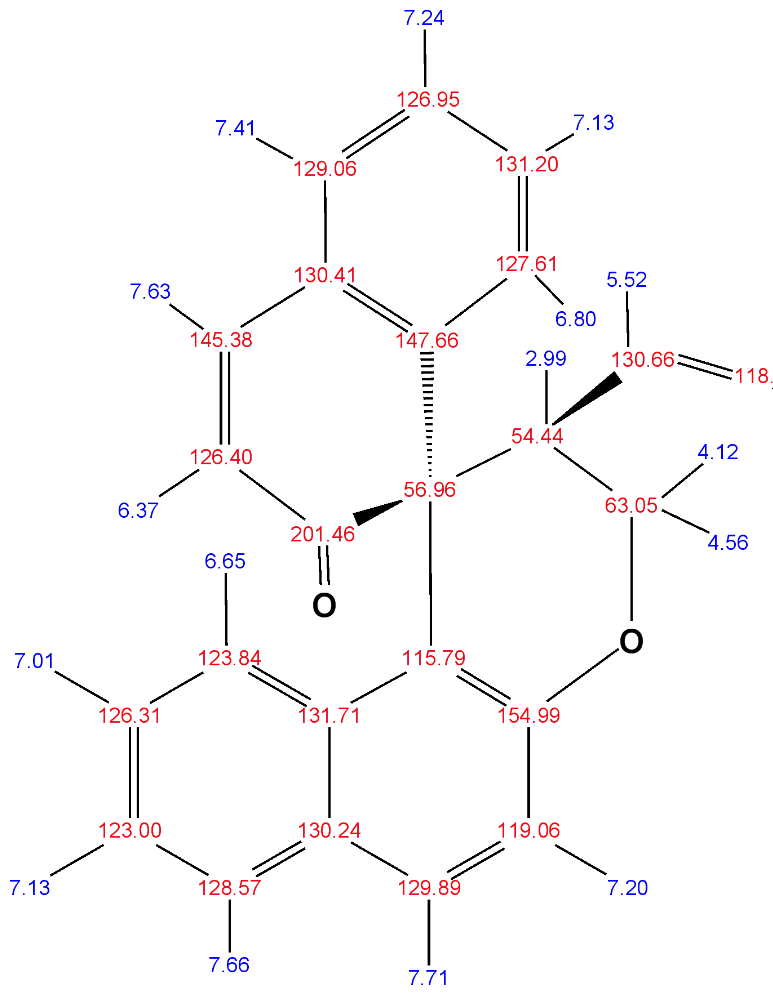
7.24
126.95
7.41
129.06
131.20
7.13
130.41
127.61
7.63
145.38
147.66
6.80
5.52
130.66
118.49
4.66
2.99
54.44
4.94
126.40
56.96
63.05
4.12
6.37
201.46
4.56
O
6.65
O
7.01
123.84
115.79
126.31
131.71
154.99
123.00
130.24
119.06
7.13
128.57
129.89
7.20
7.66
7.71

epi-**5a**

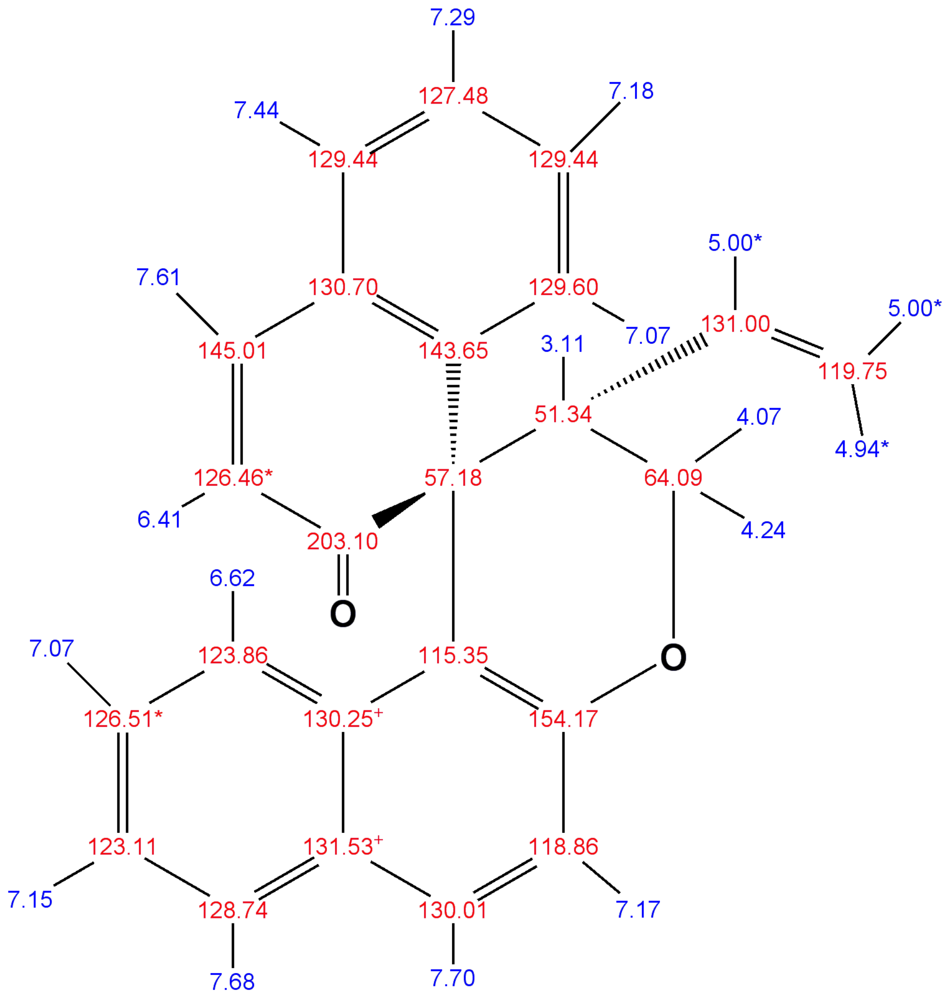
7.29
127.48
7.44
7.18
129.44
129.44
5.00*
7.61
130.70
129.60
131.00
5.00*
145.01
143.65
7.07
3.11
119.75
51.34
4.07
126.46*
57.18
64.09
4.94*
6.41
203.10
4.24
O
6.62
7.07
123.86
115.35
O
126.51*
130.25+
154.17
123.11
131.53+
118.86
7.15
128.74
130.01
7.17
7.68
7.70

5b

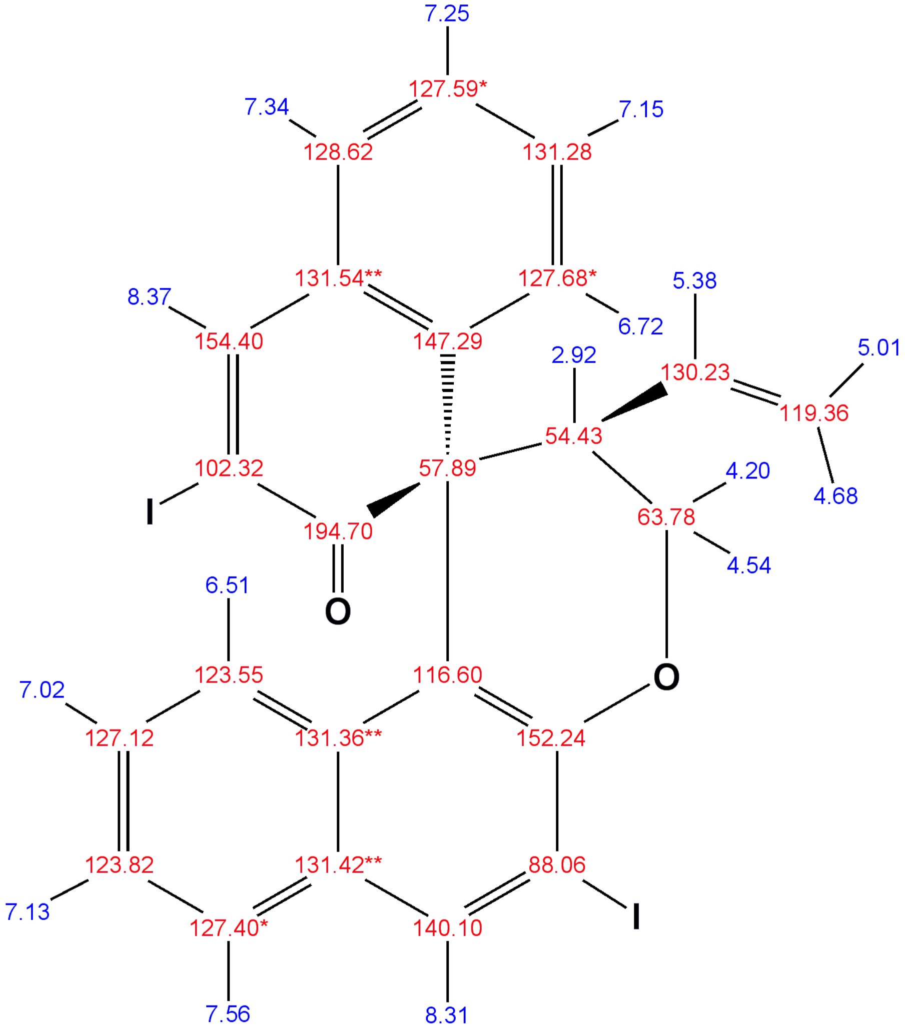
7.25
127.59*
7.34
7.15
128.62
131.28
131.54**
127.68*
8.37
5.38
154.40
147.29
6.72
2.92
130.23
5.01
54.43
119.36
102.32
57.89
4.20
I
63.78
4.68
194.70
4.54
6.51
O
7.02
123.55
116.60
O
127.12
131.36**
152.24
123.82
131.42**
88.06
7.13
127.40*
140.10
I
7.56
8.31

5c

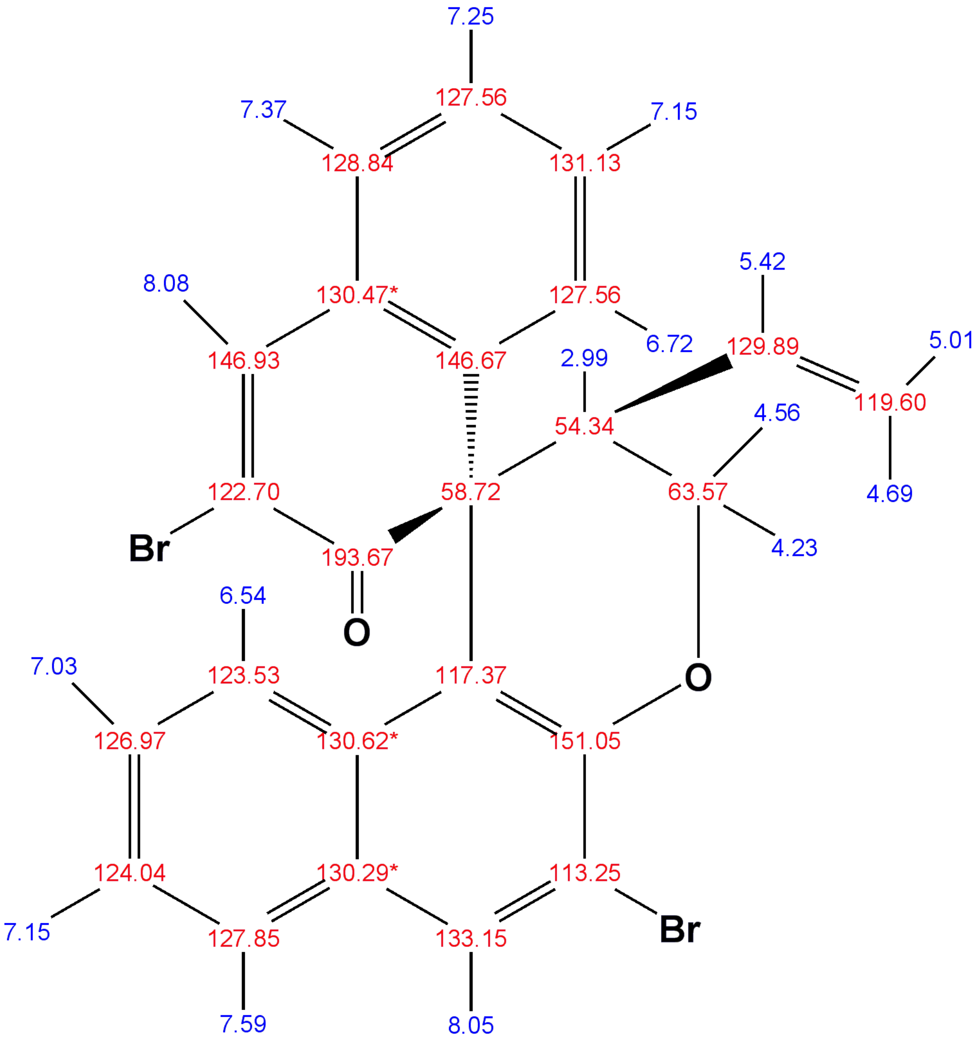
7.25
127.56
7.37
7.15
128.84
131.13
8.08
130.47*
127.56
5.42
146.93
146.67
6.72
129.89
2.99
5.01
54.34
4.56
119.60
122.70
58.72
63.57
4.69
Br
193.67
4.23
6.54
O
7.03
123.53
117.37
O
126.97
130.62*
151.05
124.04
130.29*
113.25
7.15
127.85
133.15
Br
7.59
8.05

5d

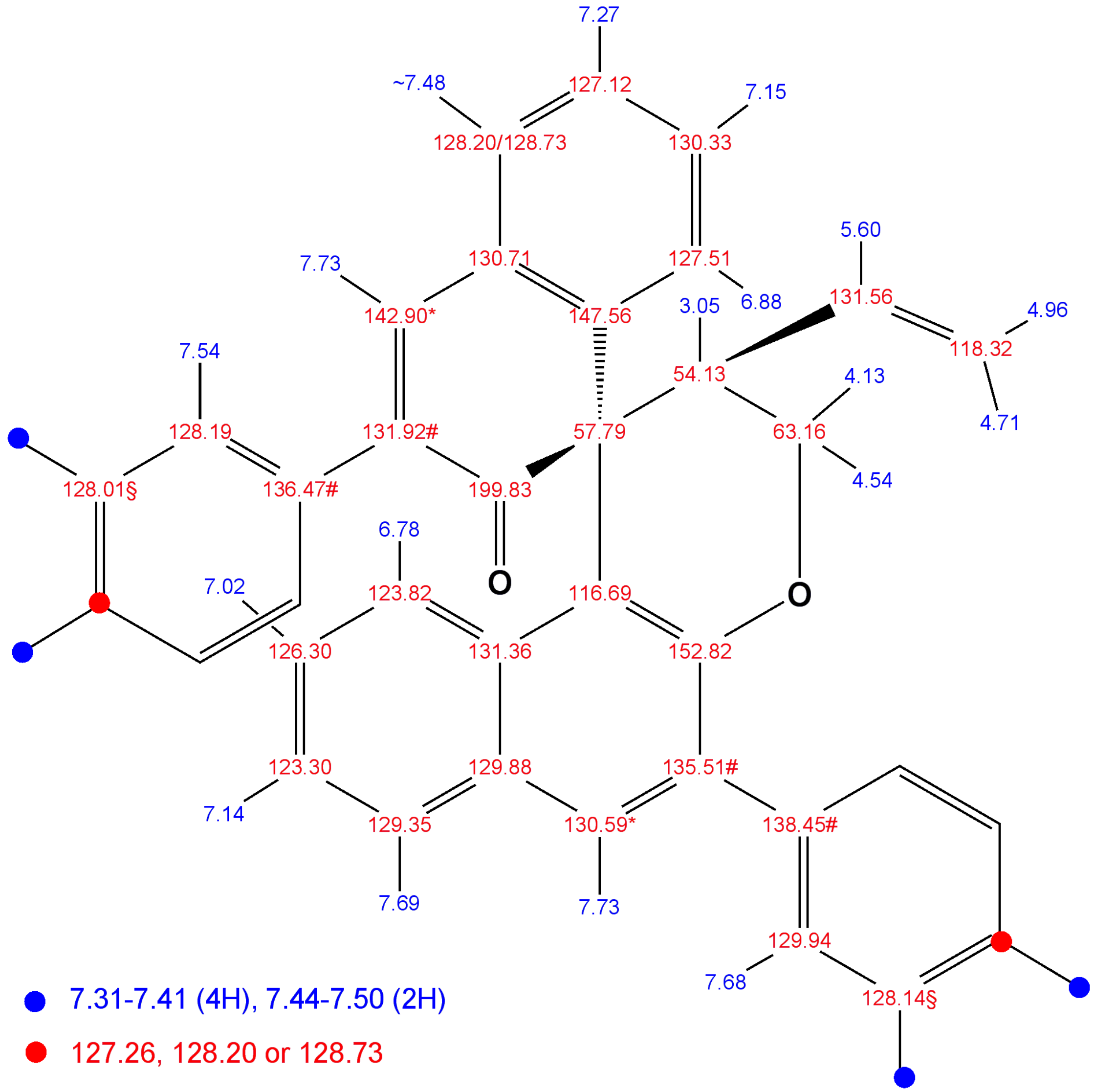
7.27
127.12
~7.48
128.20/128.73
130.33
7.15
130.71
127.51
5.60
7.73
142.90*
147.56
3.05
6.88
131.56
4.96
118.32
7.54
54.13
4.13
128.19
131.92#
57.79
63.16
4.71
128.01§
136.47#
199.83
4.54
6.78
7.02
123.82
O
116.69
O
126.30
131.36
152.82
123.30
129.88
135.51#
7.14
129.35
130.59*
138.45#
7.69
7.73
129.94
7.68
128.14§
7.31-7.41 (4H), 7.44-7.50 (2H)
127.26, 128.20 or 128.73

6a

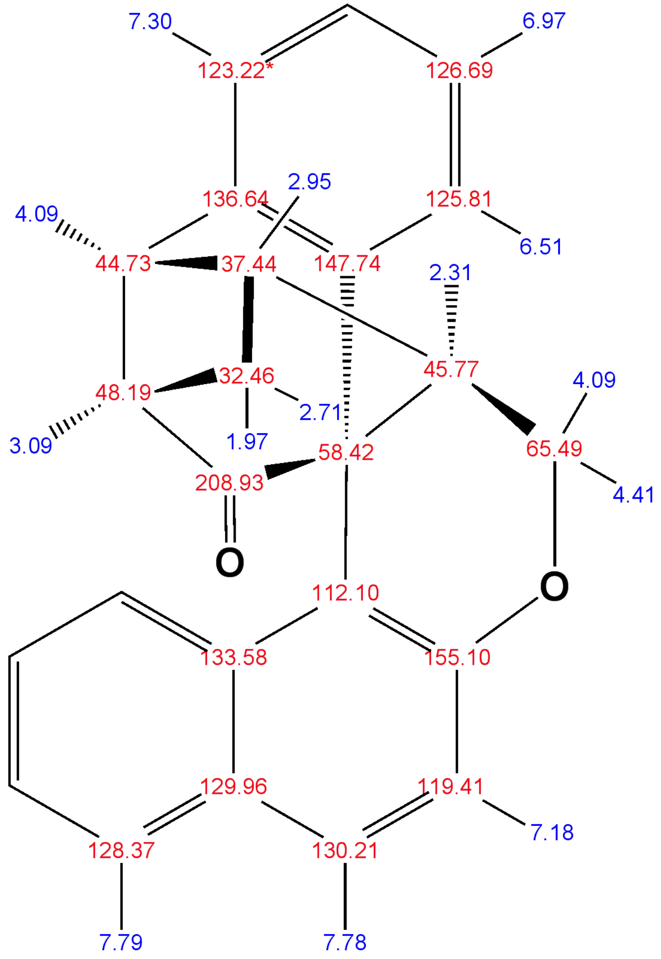

7a

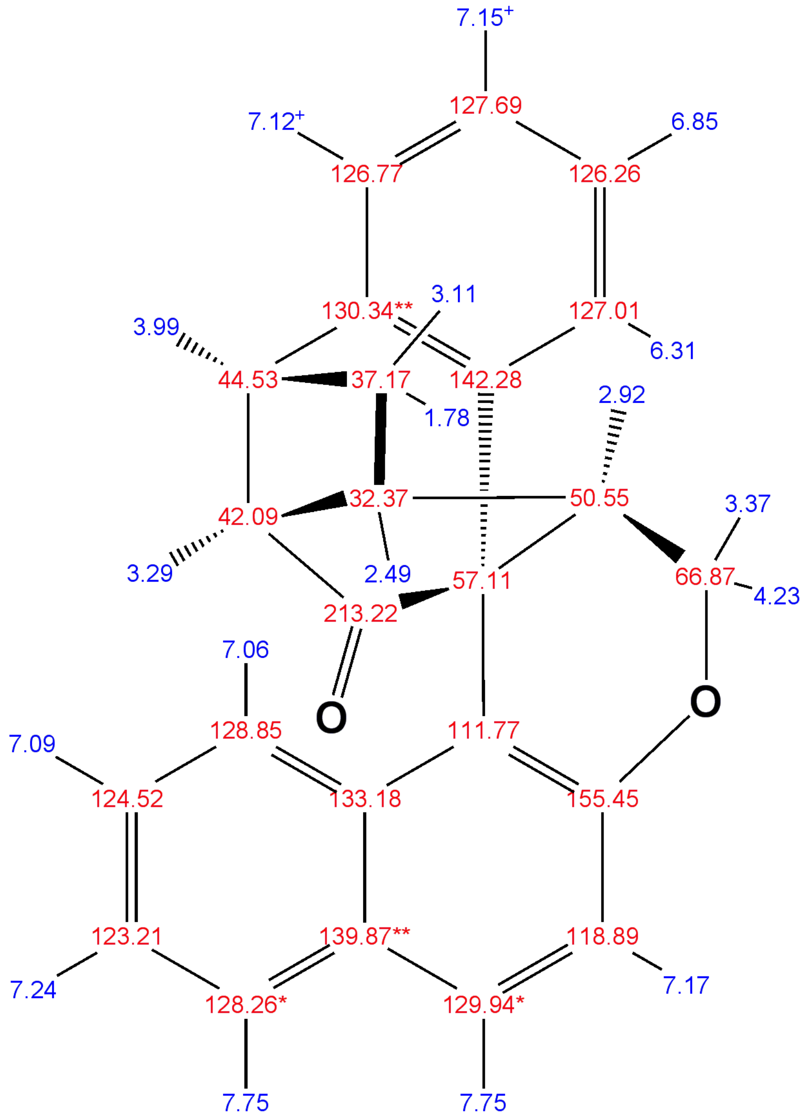

not assigned:

~7.30...........125.34*
7.18-7.21.....124.55/126.76/128.83

epi-**6a**

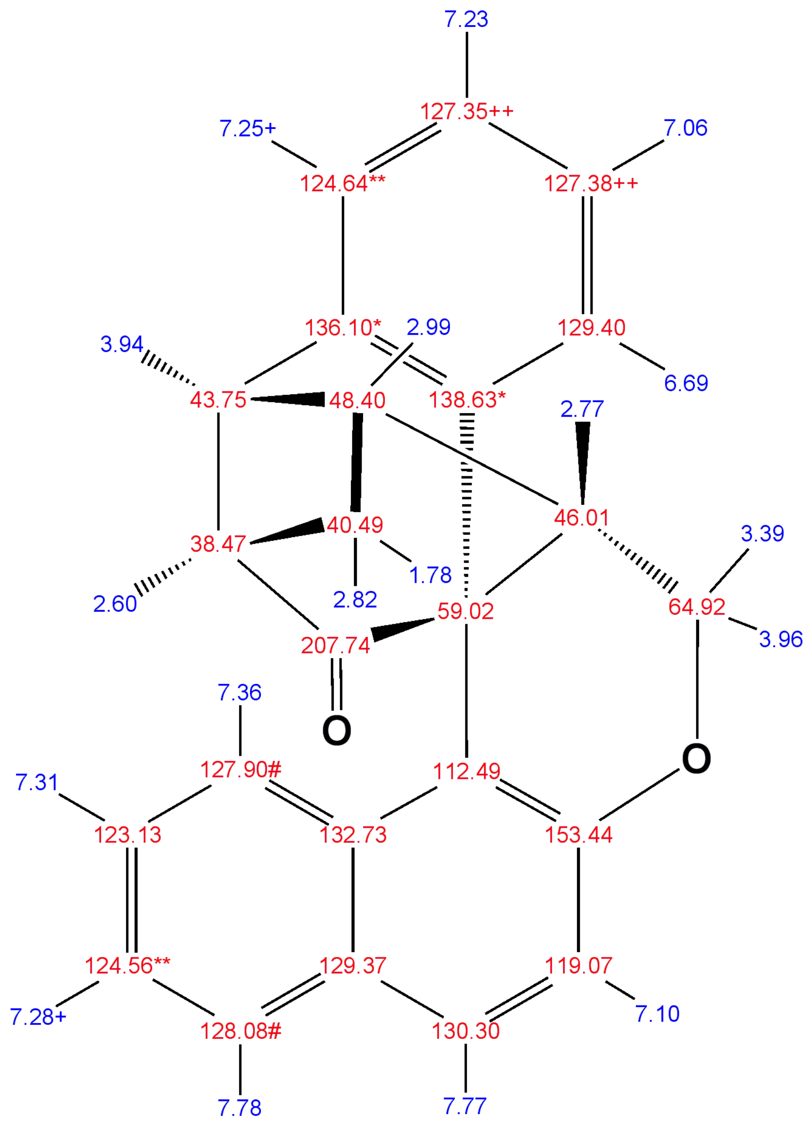
7.23
127.35++
7.25+
7.06
124.64**
127.38++
136.10*
2.99
129.40
3.94
43.75
48.40
138.63*
6.69
2.77
46.01
40.49
38.47
1.78
3.39
2.82
2.60
59.02
64.92
3.96
207.74
O
7.36
127.90#
112.49
O
7.31
123.13
132.73
153.44
124.56**
129.37
119.07
7.28+
128.08#
130.30
7.10
7.78
7.77

11

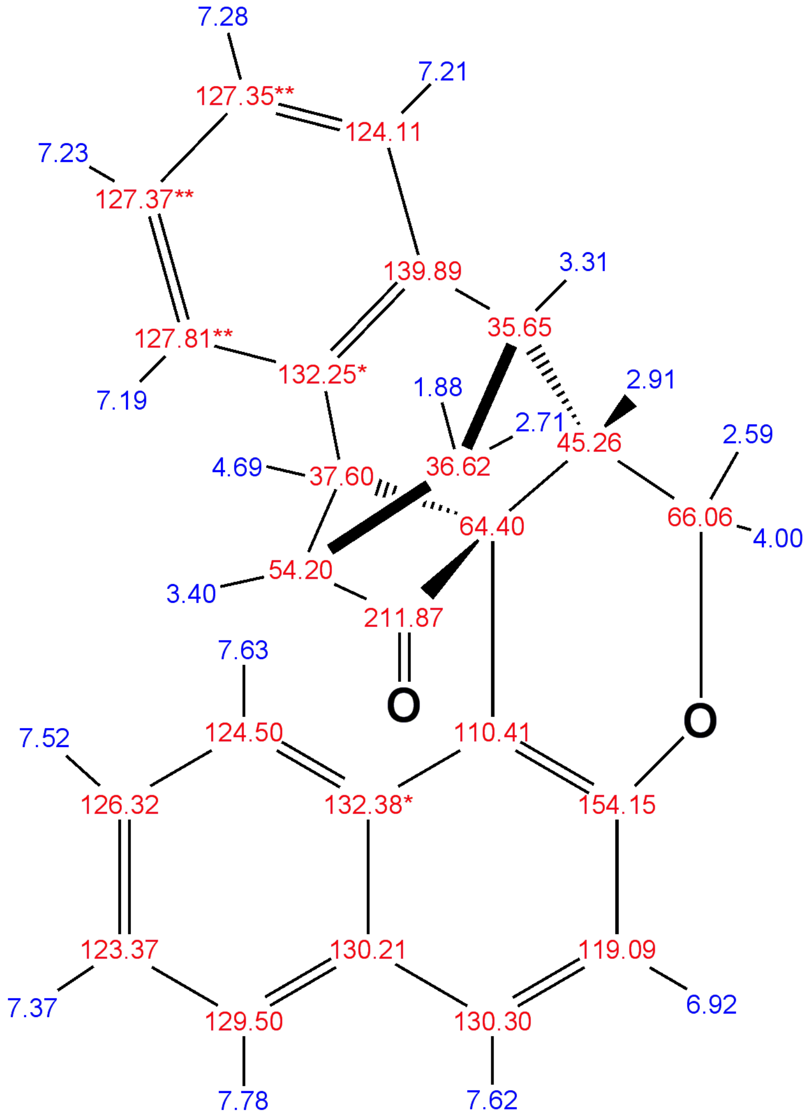
7.28
127.35**
7.21
124.11
7.23
127.37**
139.89
3.31
35.65
127.81**
132.25*
1.88
2.91
7.19
2.71
45.26
2.59
4.69
37.60
36.62
64.40
66.06
4.00
3.40
54.20
211.87
7.63
O
O
7.52
124.50
110.41
126.32
132.38*
154.15
123.37
130.21
119.09
7.37
129.50
130.30
6.92
7.78
7.62

6b

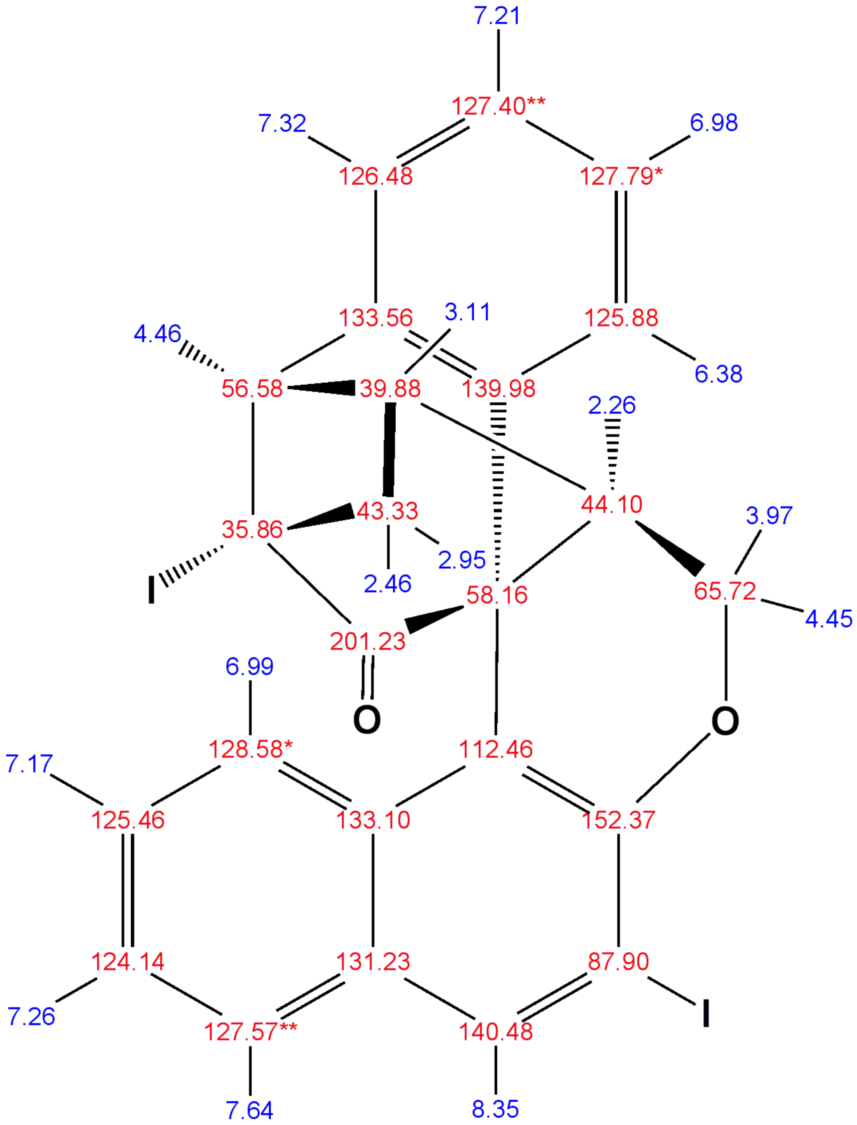
7.21
127.40**
7.32
6.98
126.48
127.79*
133.56
3.11
125.88
4.46
56.58
39.88
139.98
6.38
2.26
43.33
44.10
3.97
35.86
2.95
I
2.46
58.16
65.72
4.45
201.23
6.99
O
O
128.58*
112.46
7.17
125.46
133.10
152.37
124.14
131.23
87.90
7.26
127.57**
140.48
I
7.64
8.35

7b

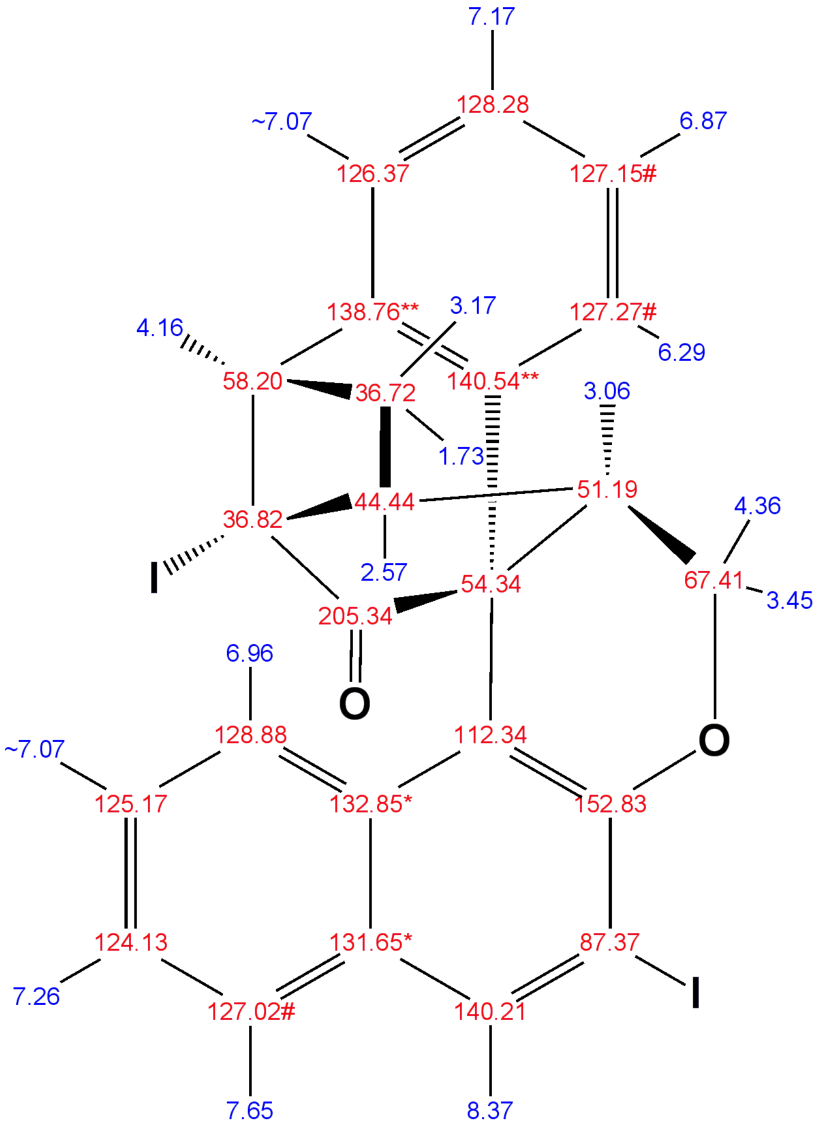
7.17
128.28
~7.07
6.87
126.37
127.15#
138.76**
3.17
127.27#
4.16
58.20
36.72
140.54**
6.29
3.06
1.73
51.19
44.44
4.36
36.82
2.57
I
54.34
67.41
3.45
205.34
6.96
O
O
128.88
112.34
~7.07
125.17
132.85*
152.83
124.13
131.65*
87.37
7.26
127.02#
140.21
I
7.65
8.37

6c

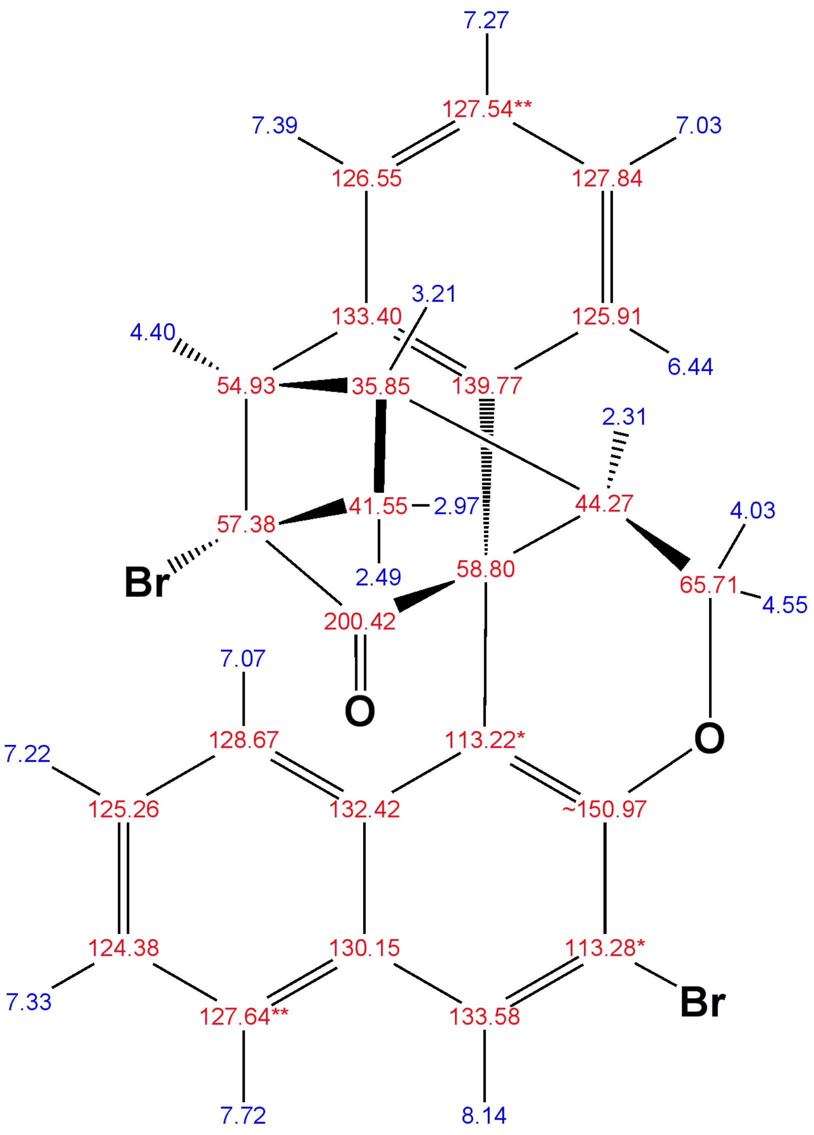
7.27
127.54**
7.39
7.03
126.55
127.84
133.40
3.21
125.91
4.40
54.93
35.85
139.77
6.44
2.31
41.55
2.97
44.27
4.03
57.38
Br
2.49
58.80
65.71
4.55
200.42
O
7.07
128.67
113.22*
O
7.22
125.26
132.42
~150.97
124.38
130.15
113.28*
7.33
127.64**
133.58
Br
7.72
8.14

7c

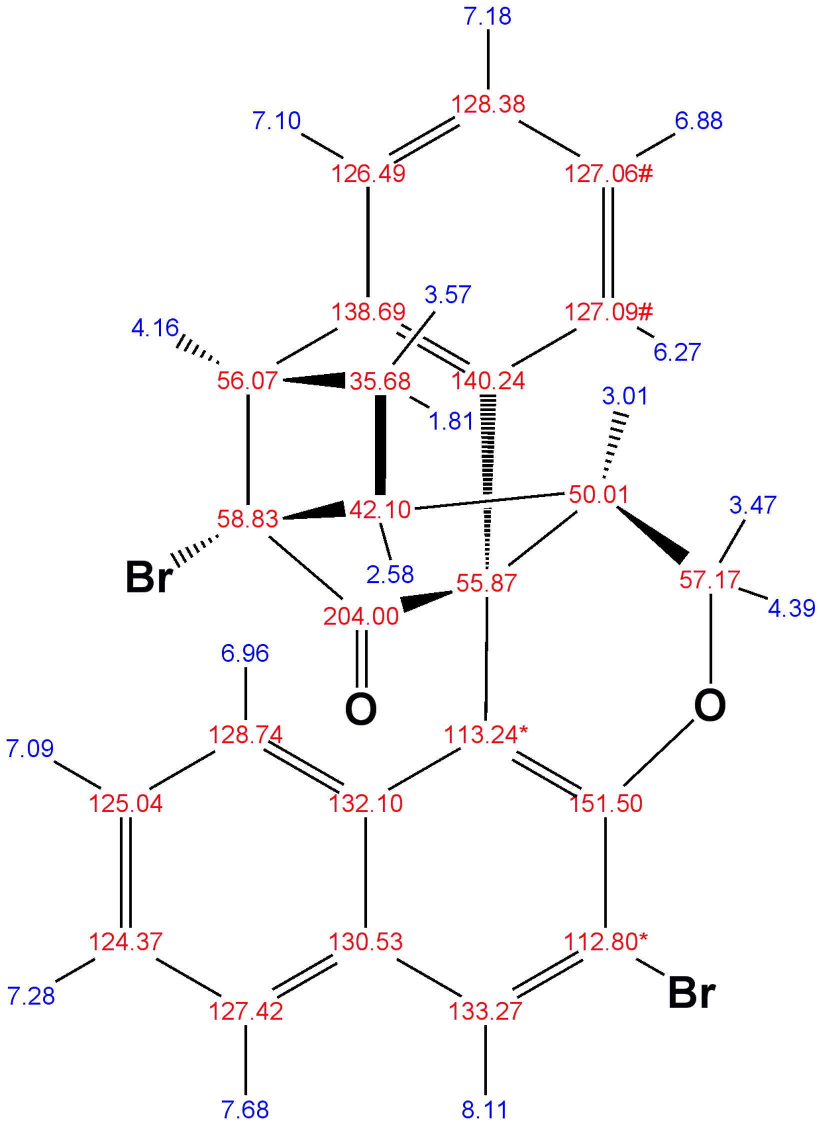
7.18
128.38
7.10
6.88
126.49
127.06#
138.69
3.57
127.09#
4.16
56.07
35.68
140.24
6.27
1.81
3.01
58.83
42.10
50.01
3.47
Br
2.58
55.87
57.17
4.39
204.00
O
6.96
128.74
113.24*
O
7.09
125.04
132.10
151.50
124.37
130.53
112.80*
7.28
127.42
133.27
Br
7.68
8.11

6d

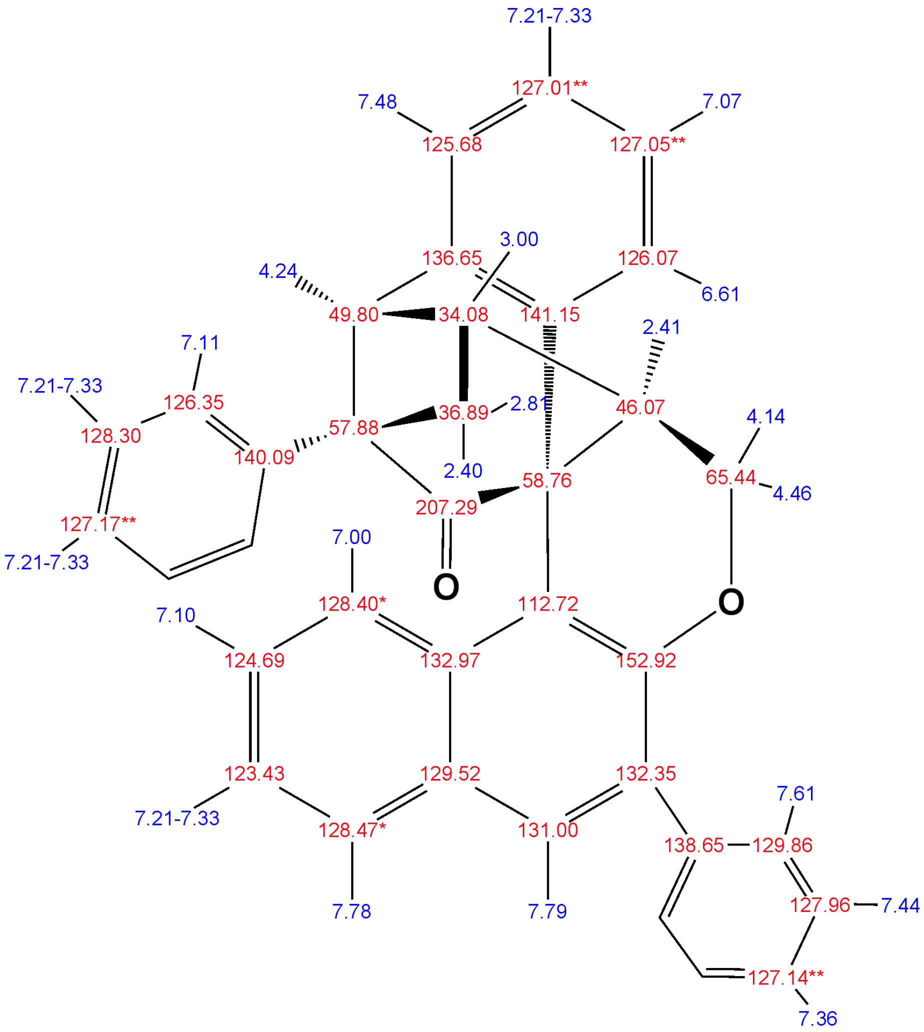
7.21-7.33
127.01**
7.48
7.07
125.68
127.05**
3.00
136.65
126.07
4.24
6.61
49.80
34.08
141.15
2.41
7.11
7.21-7.33
126.35
128.30
57.88
36.89
2.81
46.07
4.14
140.09
2.40
58.76
65.44
4.46
127.17**
207.29
7.21-7.33
7.00
O
O
7.10
128.40*
112.72
124.69
132.97
152.92
123.43
129.52
132.35
7.61
7.21-7.33
128.47*
131.00
138.65
129.86
127.96
7.44
7.78
7.79
127.14**
7.36

7d

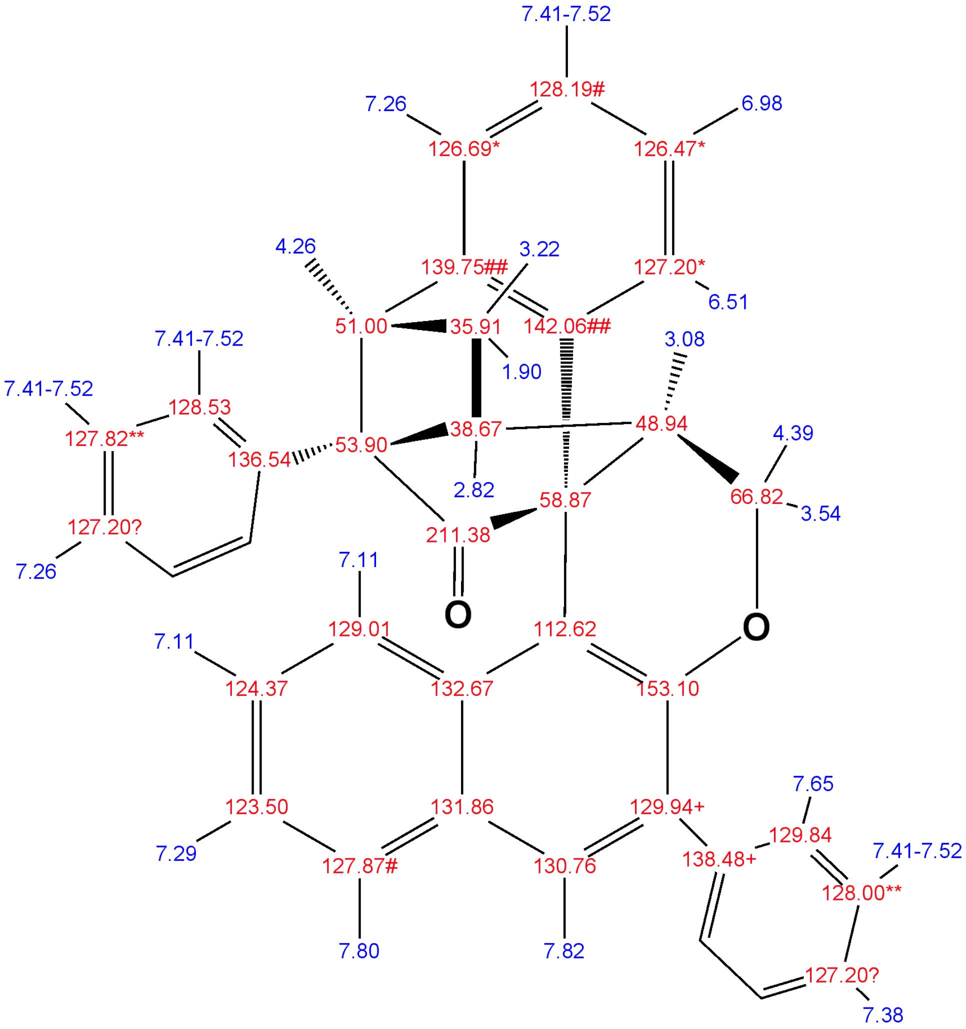
7.41-7.52
128.19#
7.26
6.98
126.69*
126.47*
4.26
3.22
139.75##
127.20*
51.00
35.91
142.06##
6.51
7.41-7.52
1.90
3.08
7.41-7.52
128.53
127.82**
136.54
53.90
38.67
48.94
4.39
2.82
58.87
66.82
3.54
127.20?
211.38
7.26
7.11
O
O
7.11
129.01
112.62
124.37
132.67
153.10
123.50
131.86
129.94+
7.65
7.29
129.84
7.41-7.52
127.87#
130.76
138.48+
128.00**
7.80
7.82
127.20?
7.38

2b

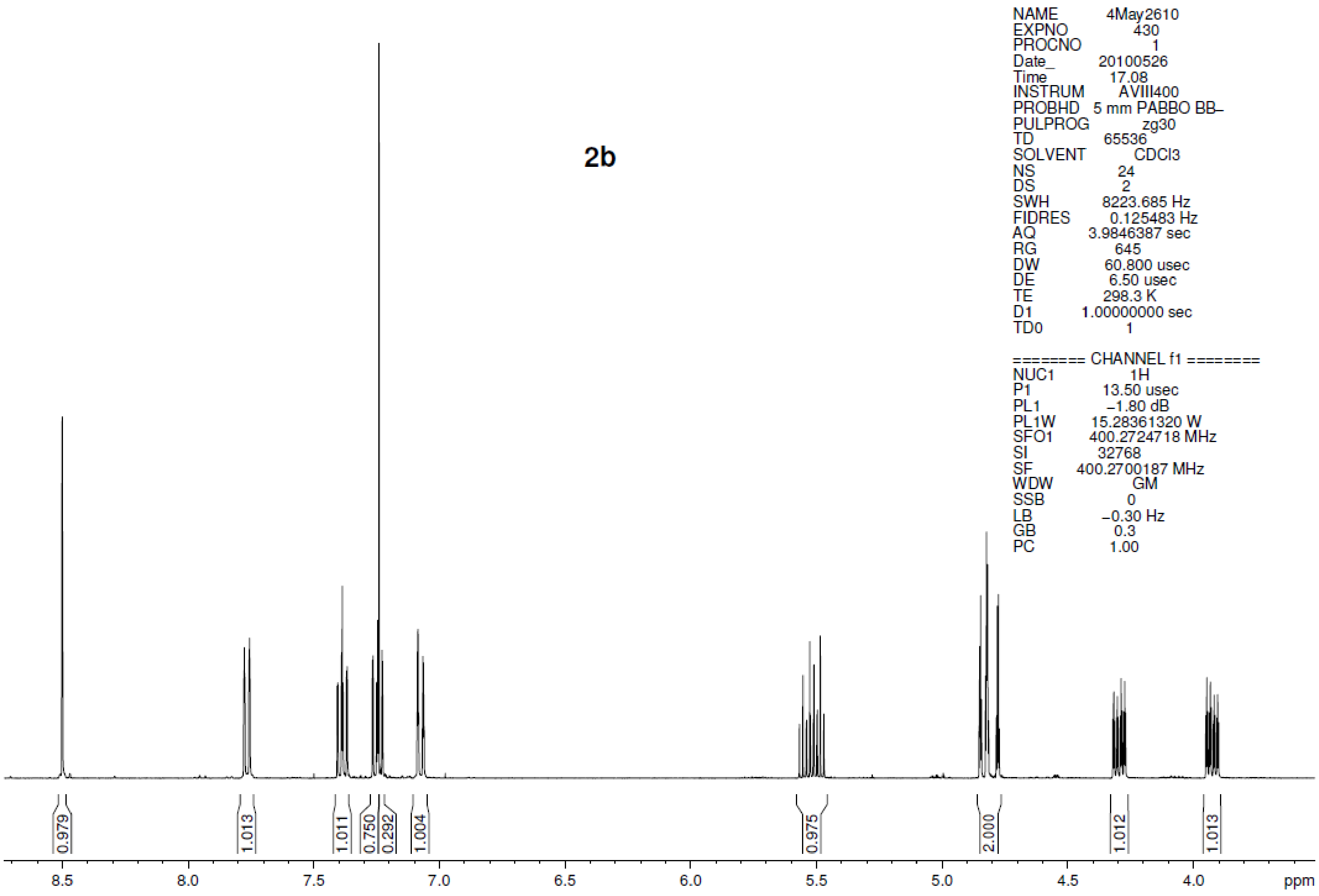
NAME 4May2610
EXPNO 430
PROCNO 1
Date_ 20100526
Time 17.08
INSTRUM AVIII400
PROBHD 5 mm PABBO BB-
PULPROG zg30
TD 65536
SOLVENT CDCl3
NS 24
DS 2
SWH 8223.685 Hz
FIDRES 0.125483 Hz
AQ 3.9846387 sec
RG 645
DW 60.800 usec
DE 6.50 usec
TE 298.3 K
D1 1.00000000 sec
TD0 1
======== CHANNEL f1 ========
NUC1 1H
P1 13.50 usec
PL1 -1.80 dB
PL1W 15.28361320 W
SFO1 400.2724718 MHz
SI 32768
SF 400.2700187 MHz
WDW GM
SSB 0
LB -0.30 Hz
GB 0.3
PC 1.00
0.979
1.013
1.011
0.750
0.292
1.004
0.975
2.000
1.012
1.013
8.5
8.0
7.5
7.0
6.5
6.0
5.5
5.0
4.5
4.0
ppm

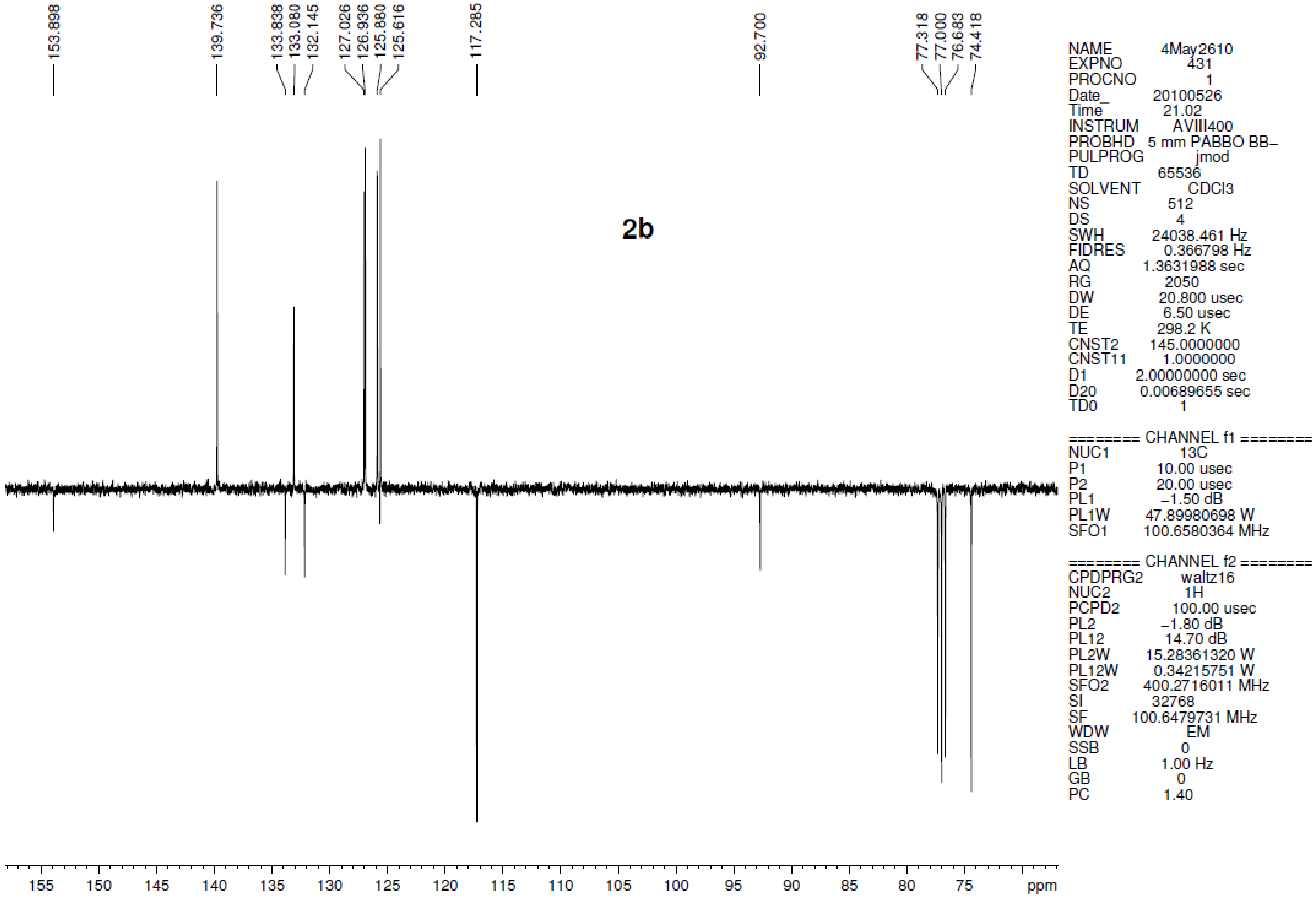

153.898
139.736
133.838
133.080
132.145
127.026
126.936
125.880
125.616
117.285
92.700
77.318
77.000
76.683
74.418
2b
155 150 145 140 135 130 125 120 115 110 105 100 95 90 85 80 75 ppm
NAME 4May2610
EXPNO 431
PROCNO 1
Date_ 20100526
Time 21.02
INSTRUM AVIII400
PROBHD 5 mm PABBO BB-
PULPROG jmod
TD 65536
SOLVENT CDCl3
NS 512
DS 4
SWH 24038.461 Hz
FIDRES 0.366798 Hz
AQ 1.3631988 sec
RG 2050
DW 20.800 usec
DE 6.50 usec
TE 298.2 K
CNST2 145.0000000
CNST11 1.0000000
D1 2.00000000 sec
D20 0.00689655 sec
TD0 1
======== CHANNEL f1 ========
NUC1 13C
P1 10.00 usec
P2 20.00 usec
PL1 -1.50 dB
PL1W 47.89980698 W
SFO1 100.6580364 MHz
======== CHANNEL f2 ========
CPDPRG2 waltz16
NUC2 1H
PCPD2 100.00 usec
PL2 -1.80 dB
PL12 14.70 dB
PL2W 15.28361320 W
PL12W 0.34215751 W
SFO2 400.2716011 MHz
SI 32768
SF 100.6479731 MHz
WDW EM
SSB 0
LB 1.00 Hz
GB 0
PC 1.40

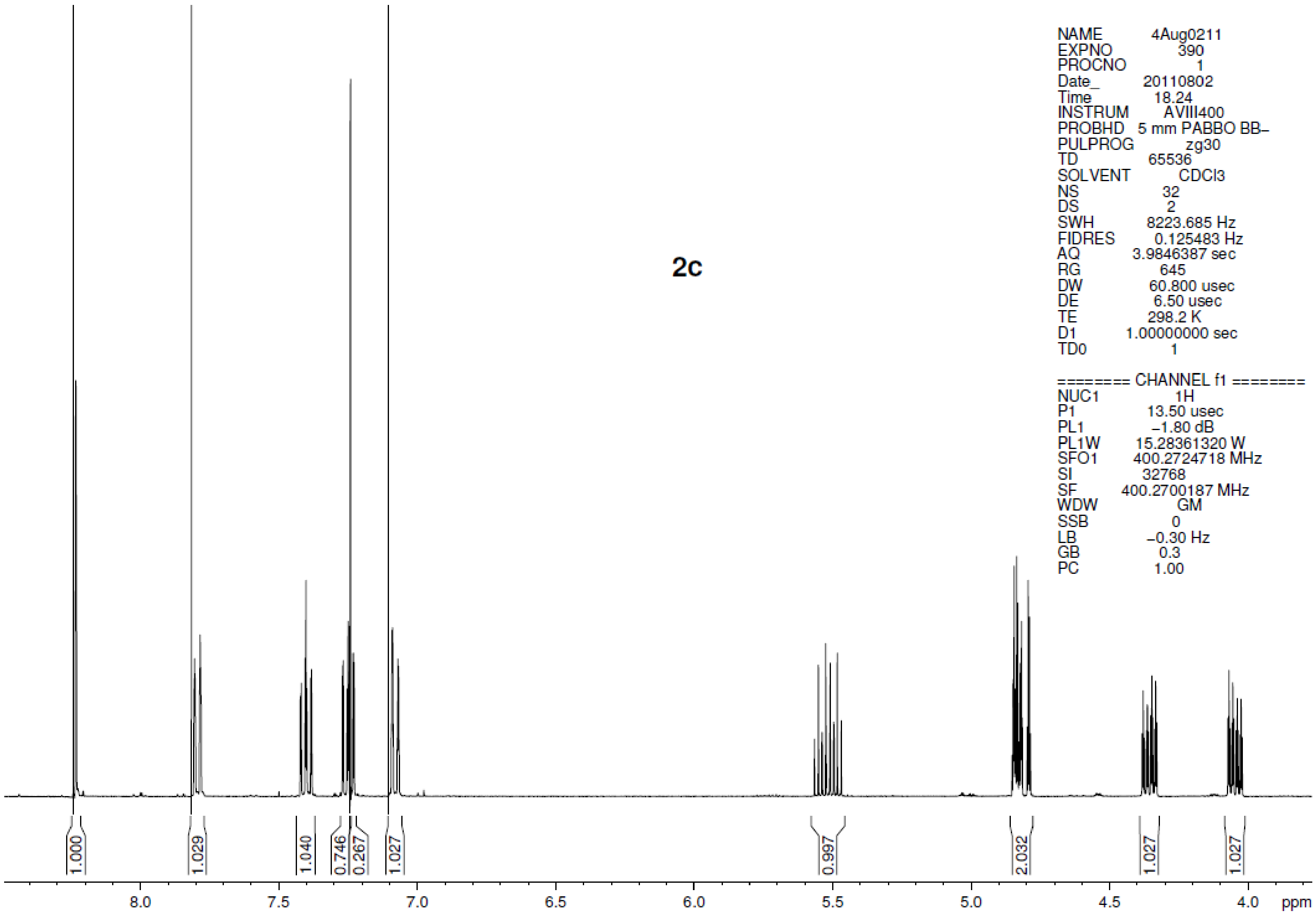
2c
NAME 4Aug0211
EXPNO 390
PROCNO 1
Date_ 20110802
Time 18.24
INSTRUM AVIII400
PROBHD 5 mm PABBO BB-
PULPROG zg30
TD 65536
SOLVENT CDCl3
NS 32
DS 2
SWH 8223.685 Hz
FIDRES 0.125483 Hz
AQ 3.9846387 sec
RG 645
DW 60.800 usec
DE 6.50 usec
TE 298.2 K
D1 1.00000000 sec
TD0 1
======== CHANNEL f1 ========
NUC1 1H
P1 13.50 usec
PL1 -1.80 dB
PL1W 15.28361320 W
SFO1 400.2724718 MHz
SI 32768
SF 400.2700187 MHz
WDW GM
SSB 0
LB -0.30 Hz
GB 0.3
PC 1.00
1.000
1.029
1.040
0.746
0.267
1.027
0.997
2.032
1.027
1.027
8.0
7.5
7.0
6.5
6.0
5.5
5.0
4.5
4.0
ppm

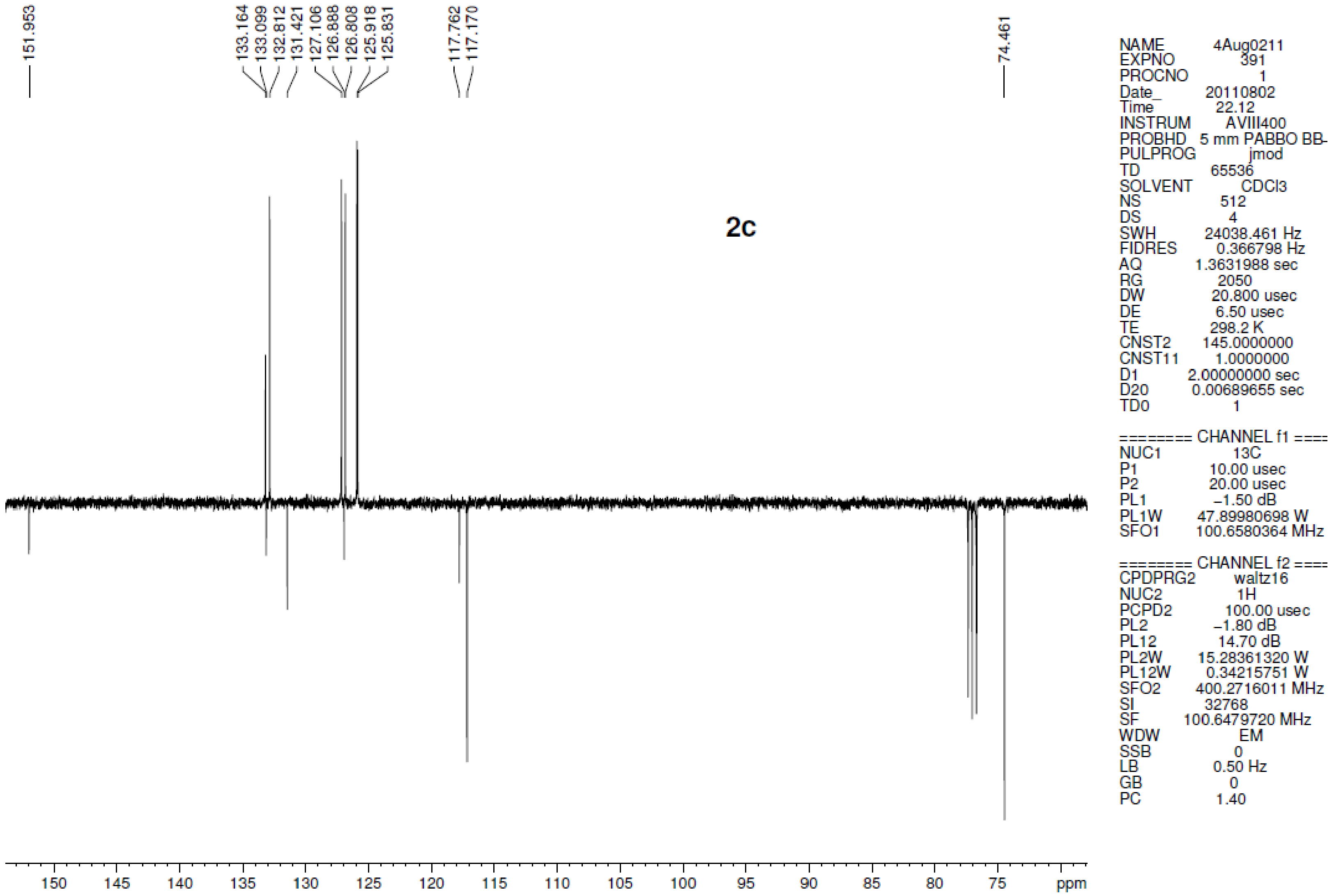
151.953
133.164
133.099
132.812
131.421
127.106
126.888
126.808
125.918
125.831
117.762
117.170
74.461
2c
150 145 140 135 130 125 120 115 110 105 100 95 90 85 80 75 ppm
NAME 4Aug0211
EXPNO 391
PROCNO 1
Date_ 20110802
Time 22.12
INSTRUM AVIII400
PROBHD 5 mm PABBO BB-
PULPROG jmod
TD 65536
SOLVENT CDCl3
NS 512
DS 4
SWH 24038.461 Hz
FIDRES 0.366798 Hz
AQ 1.3631988 sec
RG 2050
DW 20.800 usec
DE 6.50 usec
TE 298.2 K
CNST2 145.0000000
CNST11 1.0000000
D1 2.00000000 sec
D20 0.00689655 sec
TD0 1
======== CHANNEL f1 ====
NUC1 13C
P1 10.00 usec
P2 20.00 usec
PL1 -1.50 dB
PL1W 47.89980698 W
SFO1 100.6580364 MHz
======== CHANNEL f2 ====
CPDPRG2 waltz16
NUC2 1H
PCPD2 100.00 usec
PL2 -1.80 dB
PL12 14.70 dB
PL2W 15.28361320 W
PL12W 0.34215751 W
SFO2 400.2716011 MHz
SI 32768
SF 100.6479720 MHz
WDW EM
SSB 0
LB 0.50 Hz
GB 0
PC 1.40

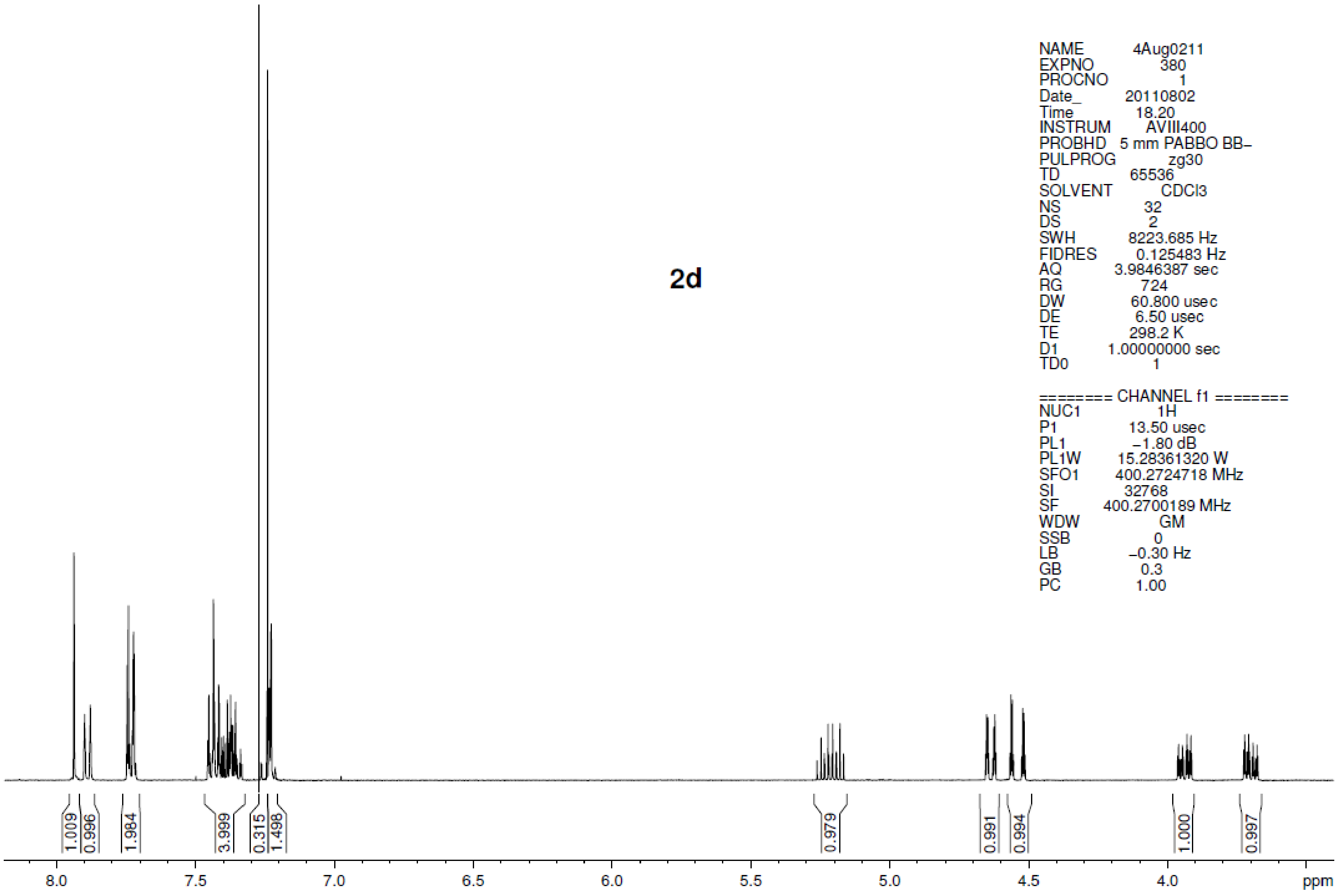
2d
NAME 4Aug0211
EXPNO 380
PROCNO 1
Date_ 20110802
Time 18.20
INSTRUM AVIII400
PROBHD 5 mm PABBO BB-
PULPROG zg30
TD 65536
SOLVENT CDCl3
NS 32
DS 2
SWH 8223.685 Hz
FIDRES 0.125483 Hz
AQ 3.9846387 sec
RG 724
DW 60.800 usec
DE 6.50 usec
TE 298.2 K
D1 1.00000000 sec
TD0 1
======== CHANNEL f1 ========
NUC1 1H
P1 13.50 usec
PL1 -1.80 dB
PL1W 15.28361320 W
SFO1 400.2724718 MHz
SI 32768
SF 400.2700189 MHz
WDW GM
SSB 0
LB -0.30 Hz
GB 0.3
PC 1.00
1.009
0.996
1.984
3.999
0.315
1.498
0.979
0.991
0.994
1.000
0.997
8.0
7.5
7.0
6.5
6.0
5.5
5.0
4.5
4.0
ppm

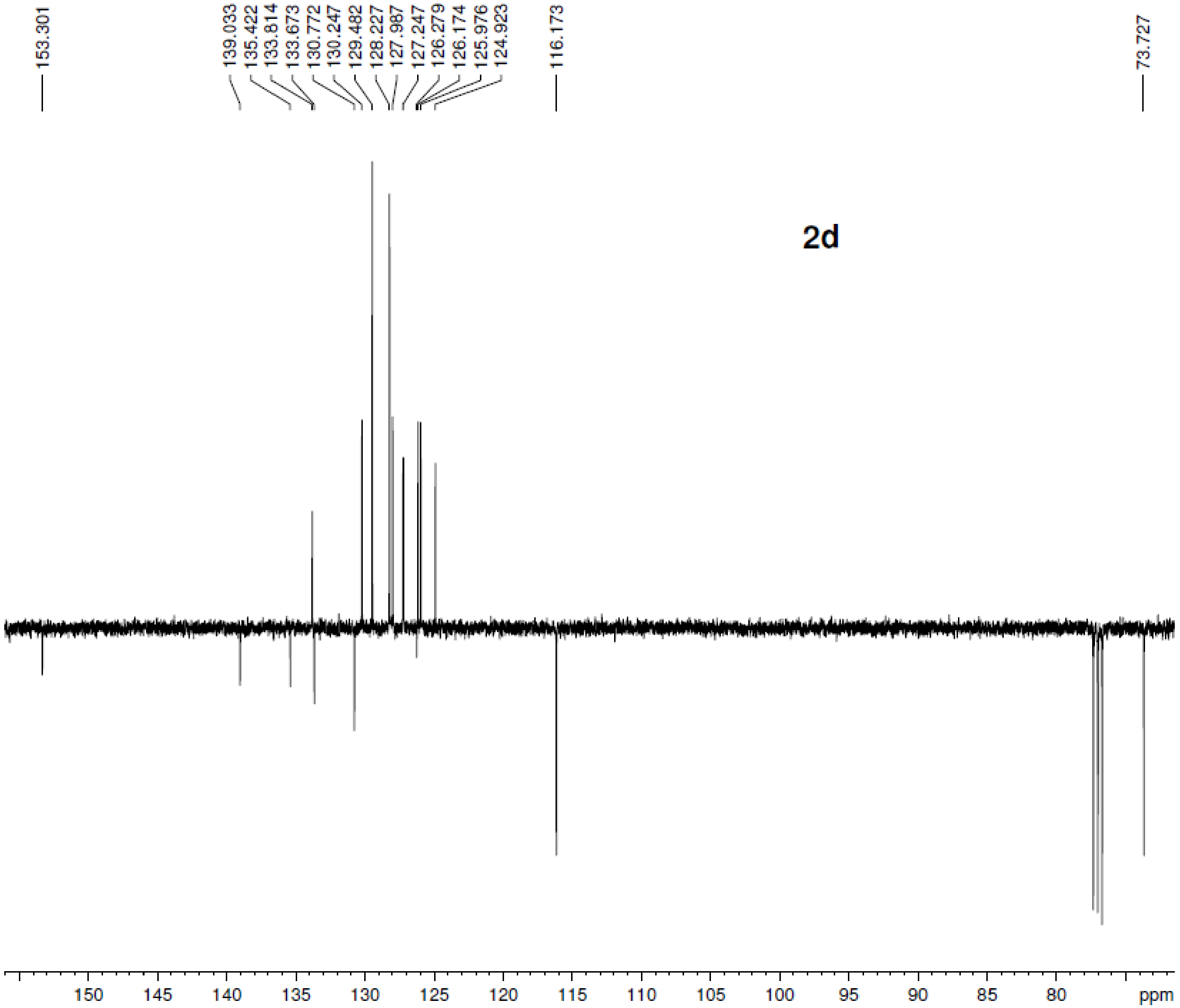

NAME	4Aug0211
EXPNO	381
PROCNO	1
Date_	20110802
Time	21.40
INSTRUM	AVIII400
PROBHD	5 mm PABBO BB-
PULPROG	jmod
TD	65536
SOLVENT	CDCl3
NS	512
DS	4
SWH	24038.461 Hz
FIDRES	0.366798 Hz
AQ	1.3631988 sec
RG	2050
DW	20.800 usec
DE	6.50 usec
TE	298.2 K
CNST2	145.0000000
CNST11	1.0000000
D1	2.00000000 sec
D20	0.00689655 sec
TD0	1
======== CHANNEL f1 ========	
NUC1	13C
P1	10.00 usec
P2	20.00 usec
PL1	-1.50 dB
PL1W	47.89980698 W
SFO1	100.6580364 MHz
======== CHANNEL f2 ========	
CPDPRG2	waltz16
NUC2	1H
PCPD2	100.00 usec
PL2	-1.80 dB
PL12	14.70 dB
PL2W	15.28361320 W
PL12W	0.34215751 W
SFO2	400.2716011 MHz
SI	32768
SF	100.6479720 MHz
WDW	EM
SSB	0
LB	0.50 Hz
GB	0
PC	1.40

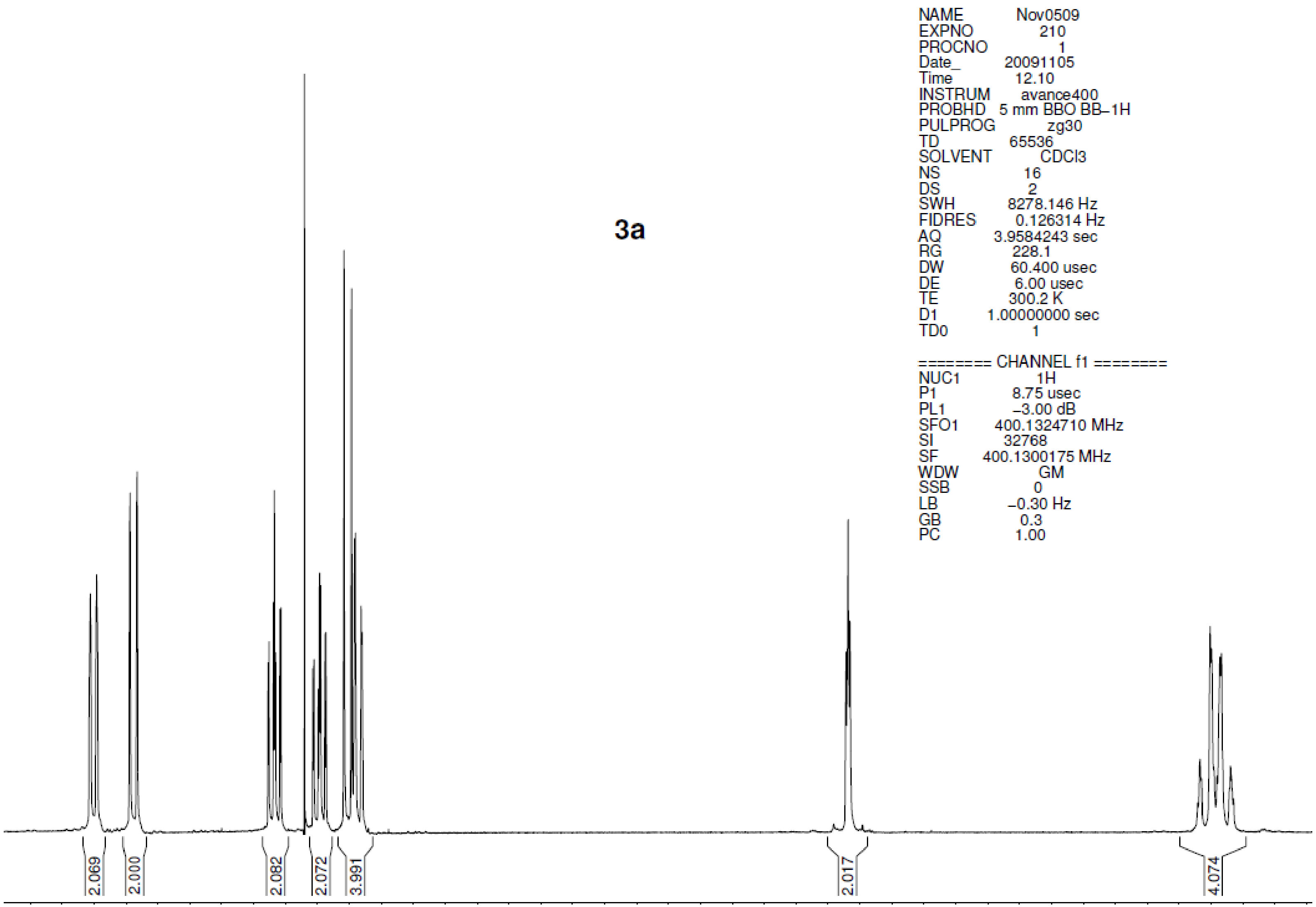
3a
NAME Nov0509
EXPNO 210
PROCNO 1
Date_ 20091105
Time 12.10
INSTRUM avance400
PROBHD 5 mm BBO BB-1H
PULPROG zg30
TD 65536
SOLVENT CDCl3
NS 16
DS 2
SWH 8278.146 Hz
FIDRES 0.126314 Hz
AQ 3.9584243 sec
RG 228.1
DW 60.400 usec
DE 6.00 usec
TE 300.2 K
D1 1.00000000 sec
TD0 1
======== CHANNEL f1 ========
NUC1 1H
P1 8.75 usec
PL1 -3.00 dB
SFO1 400.1324710 MHz
SI 32768
SF 400.1300175 MHz
WDW GM
SSB 0
LB -0.30 Hz
GB 0.3
PC 1.00
2.069
2.000
2.082
2.072
3.991
2.017
4.074

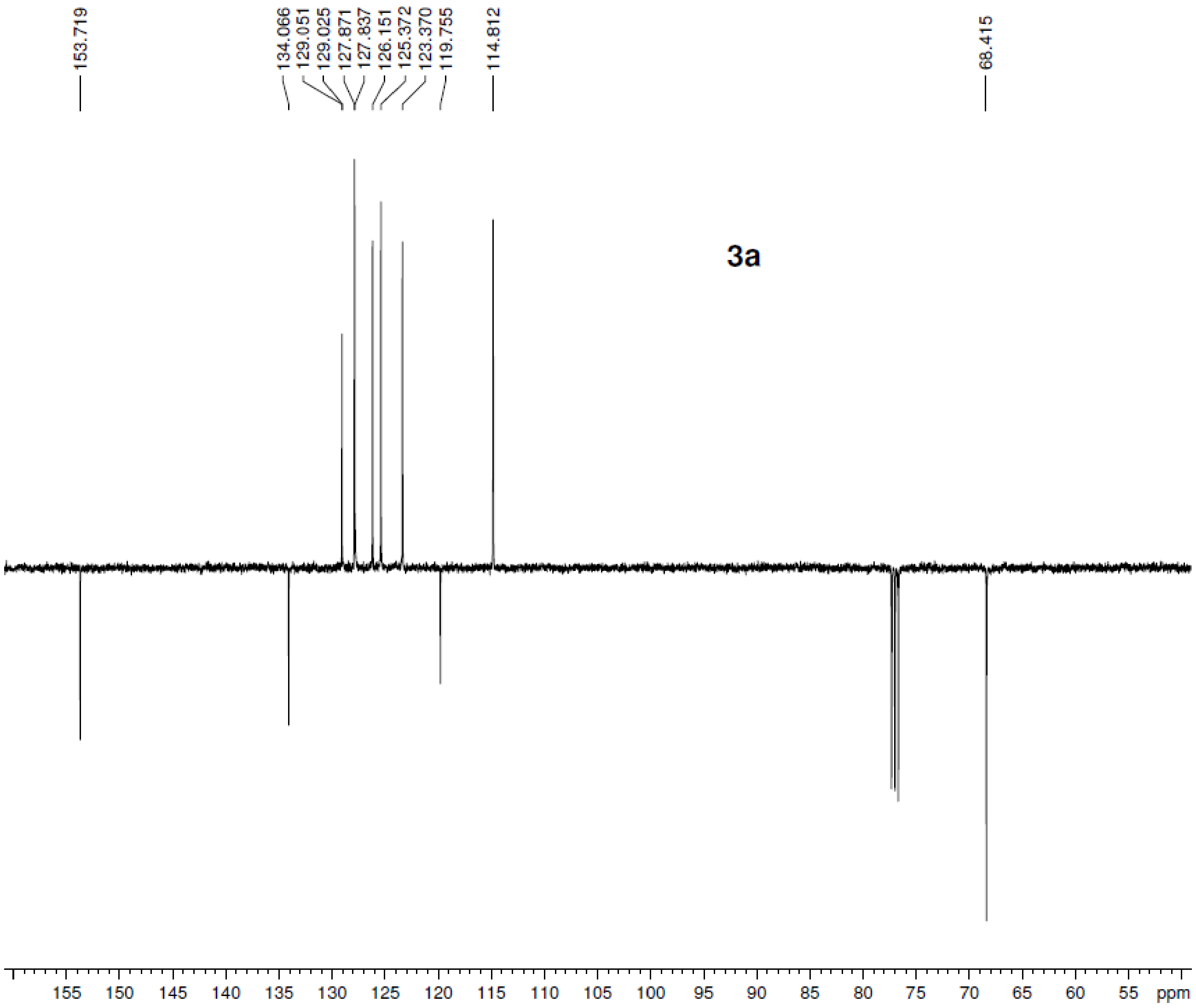

NAME Nov0509
EXPNO 211
PROCNO 1
Date_ 20091105
Time 13.07
INSTRUM avance400
PROBHD 5 mm BBO BB-1H
PULPROG jmod
TD 65536
SOLVENT CDCl3
NS 1000
DS 2
SWH 25062.656 Hz
FIDRES 0.382426 Hz
AQ 1.3074932 sec
RG 4096
DW 19.950 usec
DE 6.00 usec
TE 300.0 K
CNST2 145.0000000
CNST11 1.0000000
D1 2.00000000 sec
d20 0.00689655 sec
DELTA 0.00001311 sec
TD0 1

======== CHANNEL f1 ========
NUC1 13C
P1 10.30 usec
p2 20.60 usec
PL1 -1.00 dB
SFO1 100.6233329 MHz

======== CHANNEL f2 ========
CPDPRG2 waltz16
NUC2 1H
PCPD2 100.00 usec
PL2 -3.00 dB
PL12 16.00 dB
SFO2 400.1316005 MHz
SI 32768
SF 100.6127713 MHz
WDW EM
SSB 0
LB 1.00 Hz
GB 0
PC 1.40

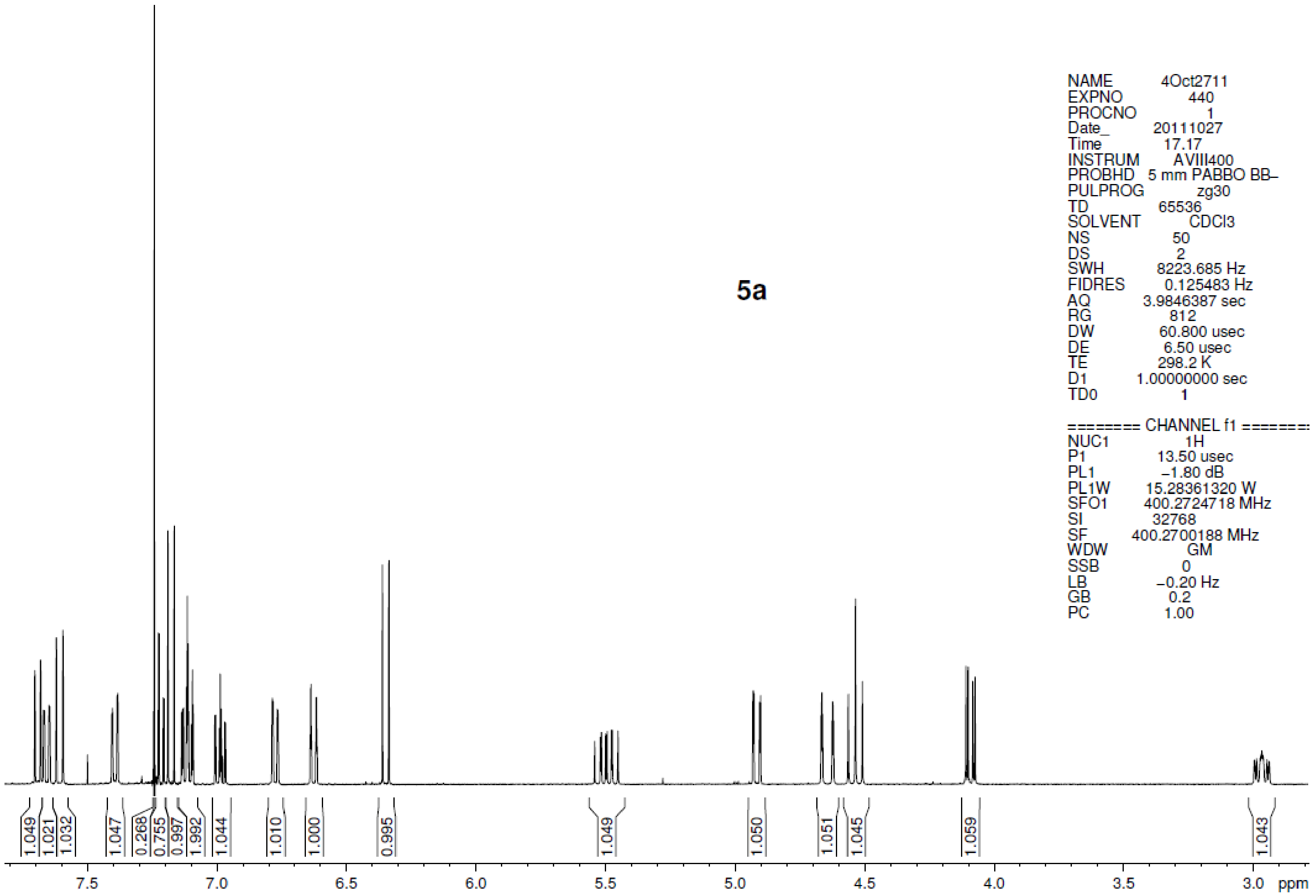

5a
NAME 4Oct2711
EXPNO 440
PROCNO 1
Date_ 20111027
Time 17.17
INSTRUM AVIII400
PROBHD 5 mm PABBO BB–
PULPROG zg30
TD 65536
SOLVENT CDCl3
NS 50
DS 2
SWH 8223.685 Hz
FIDRES 0.125483 Hz
AQ 3.9846387 sec
RG 812
DW 60.800 usec
DE 6.50 usec
TE 298.2 K
D1 1.00000000 sec
TD0 1
======== CHANNEL f1 ========
NUC1 1H
P1 13.50 usec
PL1 –1.80 dB
PL1W 15.28361320 W
SFO1 400.2724718 MHz
SI 32768
SF 400.2700188 MHz
WDW GM
SSB 0
LB –0.20 Hz
GB 0.2
PC 1.00
1.049
1.021
1.032
1.047
0.268
0.755
0.997
1.992
1.044
1.010
1.000
0.995
1.049
1.050
1.051
1.045
1.059
1.043
7.5
7.0
6.5
6.0
5.5
5.0
4.5
4.0
3.5
3.0
ppm

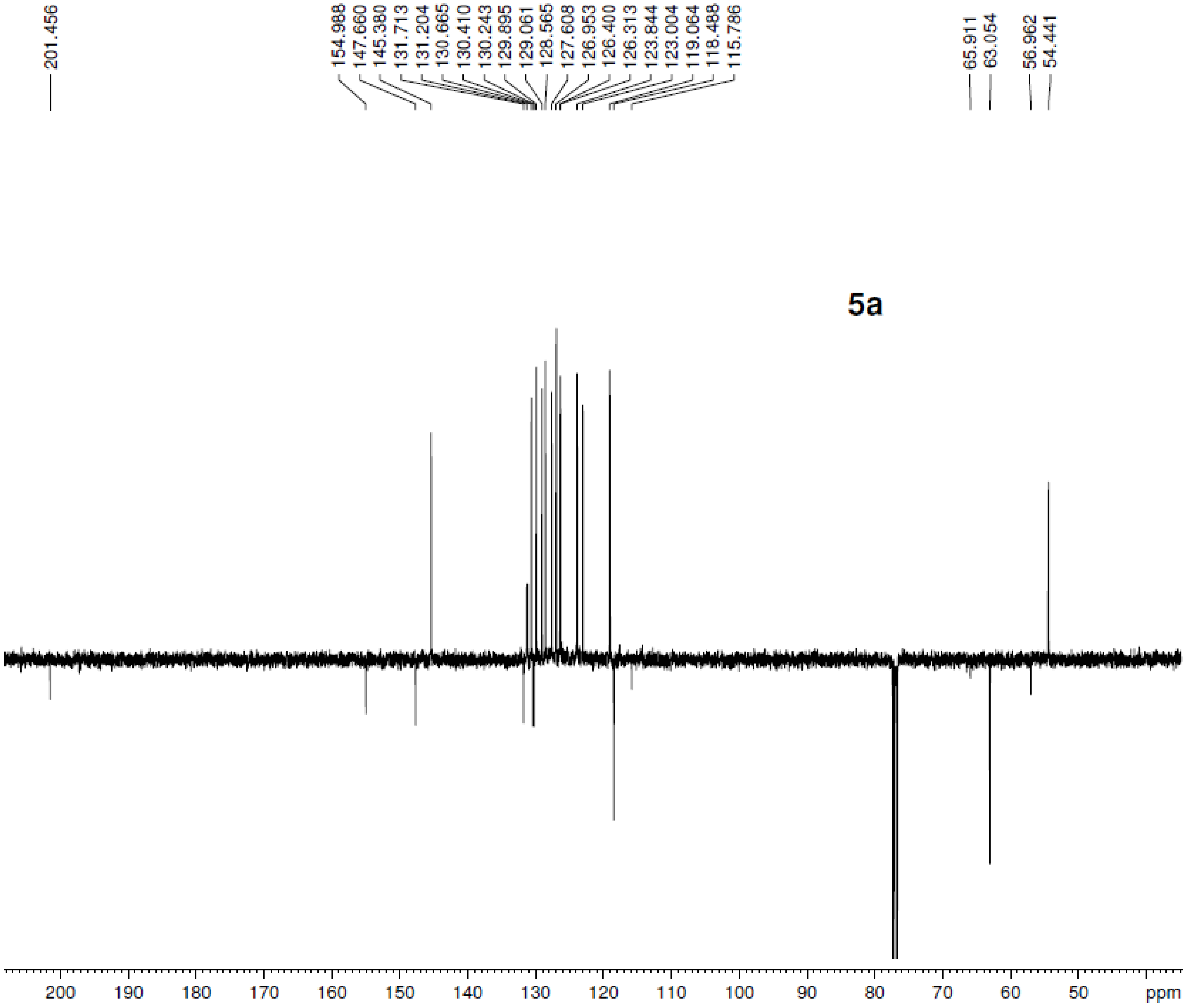

5a

NAME Apr0209
EXPNO 61
PROCNO 1
Date_ 20090403
Time 4.56
INSTRUM avance400
PROBHD 5 mm BBO BB-1H
PULPROG jmod
TD 65536
SOLVENT CDCl3
NS 1200
DS 2
SWH 25062.656 Hz
FIDRES 0.382426 Hz
AQ 1.3074932 sec
RG 3649.1
DW 19.950 usec
DE 6.00 usec
TE 300.2 K
CNST2 145.0000000
CNST11 1.0000000
D1 2.00000000 sec
d20 0.00689655 sec
DELTA 0.00001311 sec
TD0 1

======== CHANNEL f1 ========
NUC1 13C
P1 10.30 usec
p2 20.60 usec
PL1 −1.00 dB
SFO1 100.6233329 MHz

======== CHANNEL f2 ========
CPDPRG2 waltz16
NUC2 1H
PCPD2 100.00 usec
PL2 −3.00 dB
PL12 16.00 dB
SFO2 400.1316005 MHz
SI 32768
SF 100.6127706 MHz
WDW EM
SSB 0
LB 1.00 Hz
GB 0
PC 1.40

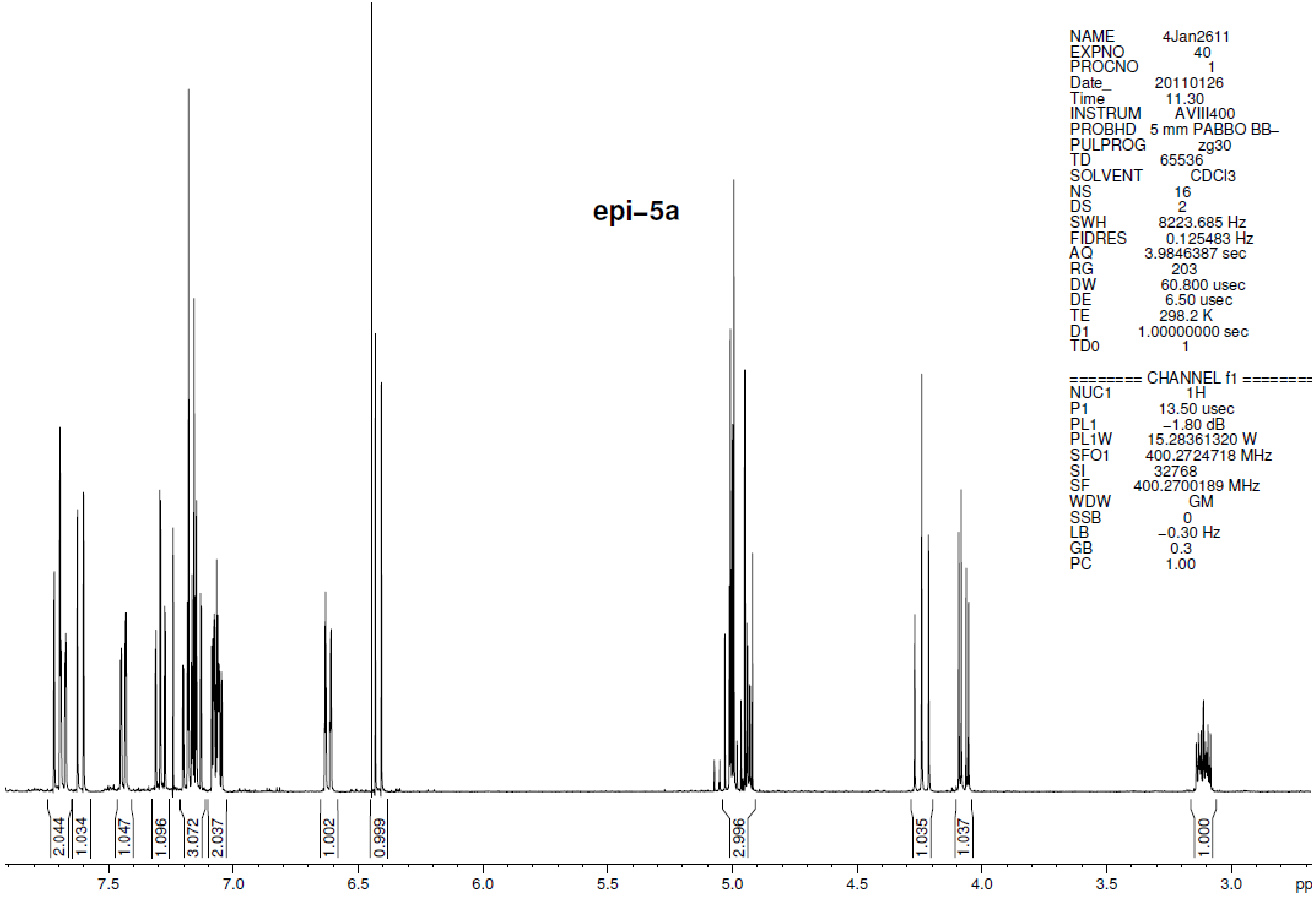
epi–5a
NAME 4Jan2611
EXPNO 40
PROCNO 1
Date_ 20110126
Time 11.30
INSTRUM AVIII400
PROBHD 5 mm PABBO BB–
PULPROG zg30
TD 65536
SOLVENT CDCl3
NS 16
DS 2
SWH 8223.685 Hz
FIDRES 0.125483 Hz
AQ 3.9846387 sec
RG 203
DW 60.800 usec
DE 6.50 usec
TE 298.2 K
D1 1.00000000 sec
TD0 1
======== CHANNEL f1 ========
NUC1 1H
P1 13.50 usec
PL1 –1.80 dB
PL1W 15.28361320 W
SFO1 400.2724718 MHz
SI 32768
SF 400.2700189 MHz
WDW GM
SSB 0
LB –0.30 Hz
GB 0.3
PC 1.00
2.044
1.034
1.047
1.096
3.072
2.037
1.002
0.999
2.996
1.035
1.037
1.000
7.5
7.0
6.5
6.0
5.5
5.0
4.5
4.0
3.5
3.0
pp

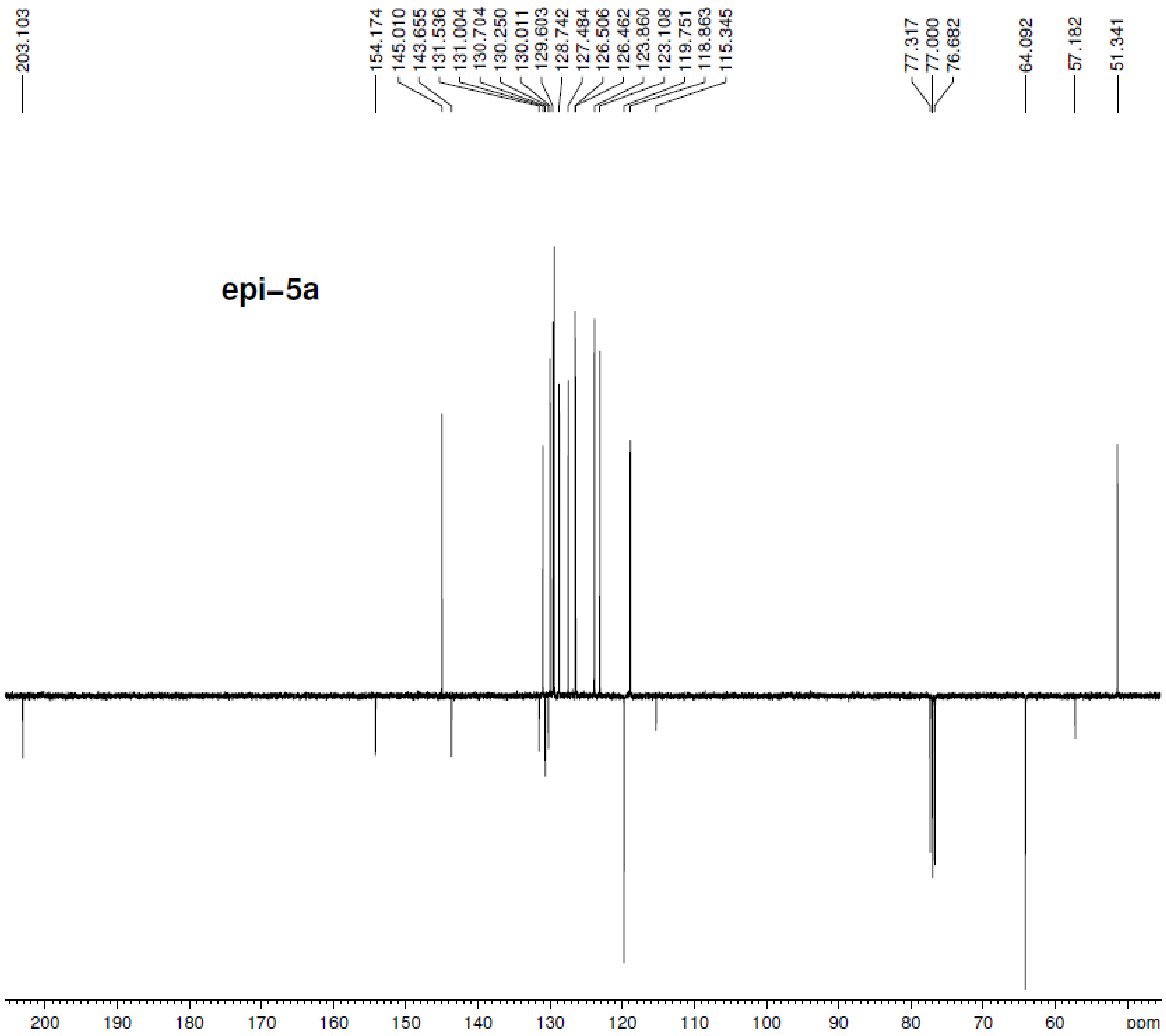

NAME	4Jan2611
EXPNO	41
PROCNO	1
Date_	20110126
Time	21.30
INSTRUM	AVIII400
PROBHD	5 mm PABBO BB-
PULPROG	jmod
TD	65536
SOLVENT	CDCl3
NS	1000
DS	4
SWH	24038.461 Hz
FIDRES	0.366798 Hz
AQ	1.3631988 sec
RG	2050
DW	20.800 usec
DE	6.50 usec
TE	298.2 K
CNST2	145.0000000
CNST11	1.0000000
D1	2.00000000 sec
D20	0.00689655 sec
TD0	1

======== CHANNEL f1 ========

NUC1	13C
P1	10.00 usec
P2	20.00 usec
PL1	-1.50 dB
PL1W	47.89980698 W
SFO1	100.6580364 MHz

======== CHANNEL f2 ========

CPDPRG2	waltz16
NUC2	1H
PCPD2	100.00 usec
PL2	-1.80 dB
PL12	14.70 dB
PL2W	15.28361320 W
PL12W	0.34215751 W
SFO2	400.2716011 MHz
SI	32768
SF	100.6479752 MHz
WDW	EM
SSB	0
LB	0.50 Hz
GB	0
PC	1.40

5b

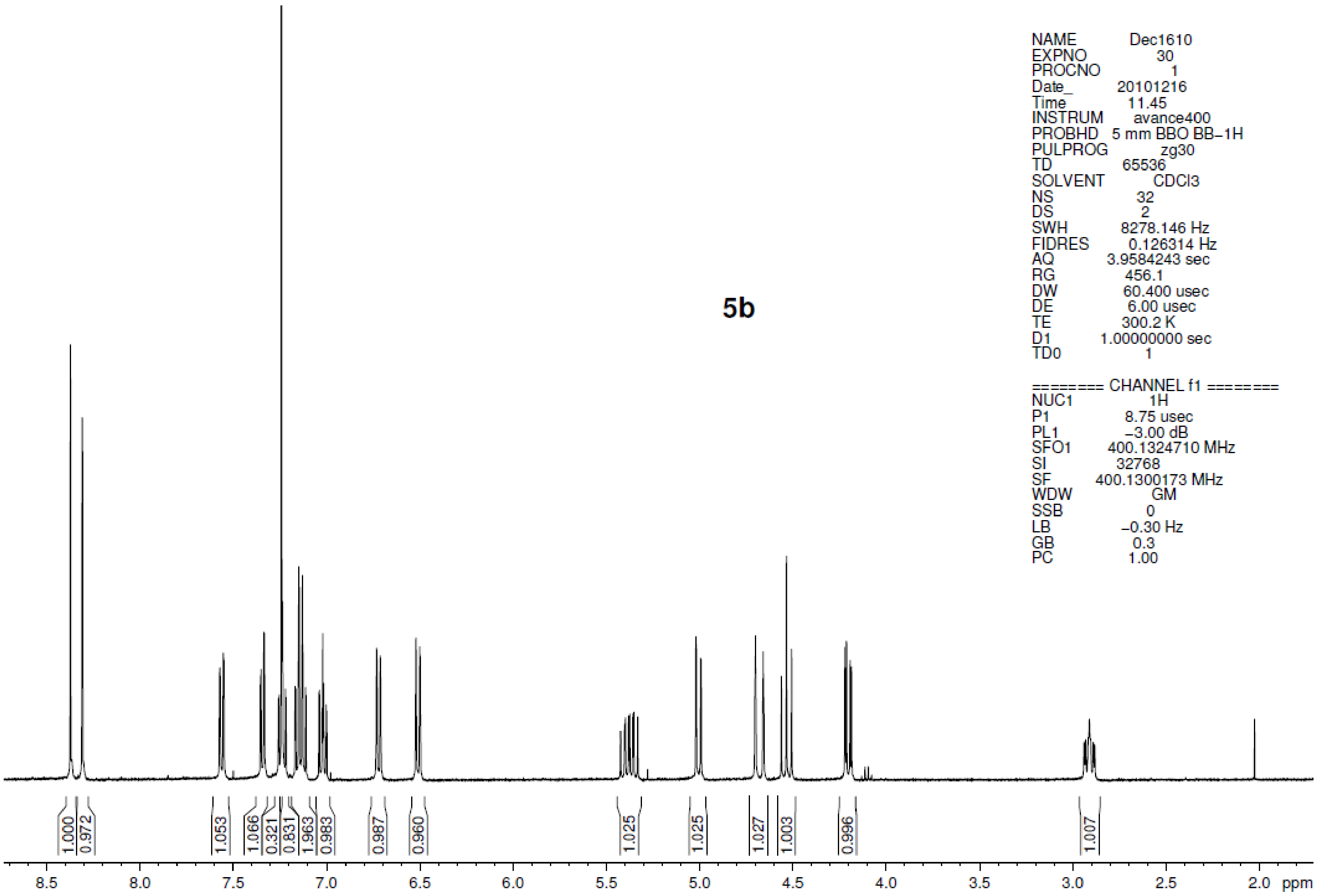
NAME Dec1610
EXPNO 30
PROCNO 1
Date_ 20101216
Time 11.45
INSTRUM avance400
PROBHD 5 mm BBO BB-1H
PULPROG zg30
TD 65536
SOLVENT CDCl3
NS 32
DS 2
SWH 8278.146 Hz
FIDRES 0.126314 Hz
AQ 3.9584243 sec
RG 456.1
DW 60.400 usec
DE 6.00 usec
TE 300.2 K
D1 1.00000000 sec
TD0 1
======== CHANNEL f1 ========
NUC1 1H
P1 8.75 usec
PL1 -3.00 dB
SFO1 400.1324710 MHz
SI 32768
SF 400.1300173 MHz
WDW GM
SSB 0
LB -0.30 Hz
GB 0.3
PC 1.00
1.000
0.972
1.053
1.066
0.321
0.831
1.963
0.983
0.987
0.960
1.025
1.025
1.027
1.003
0.996
1.007
8.5
8.0
7.5
7.0
6.5
6.0
5.5
5.0
4.5
4.0
3.5
3.0
2.5
2.0
ppm

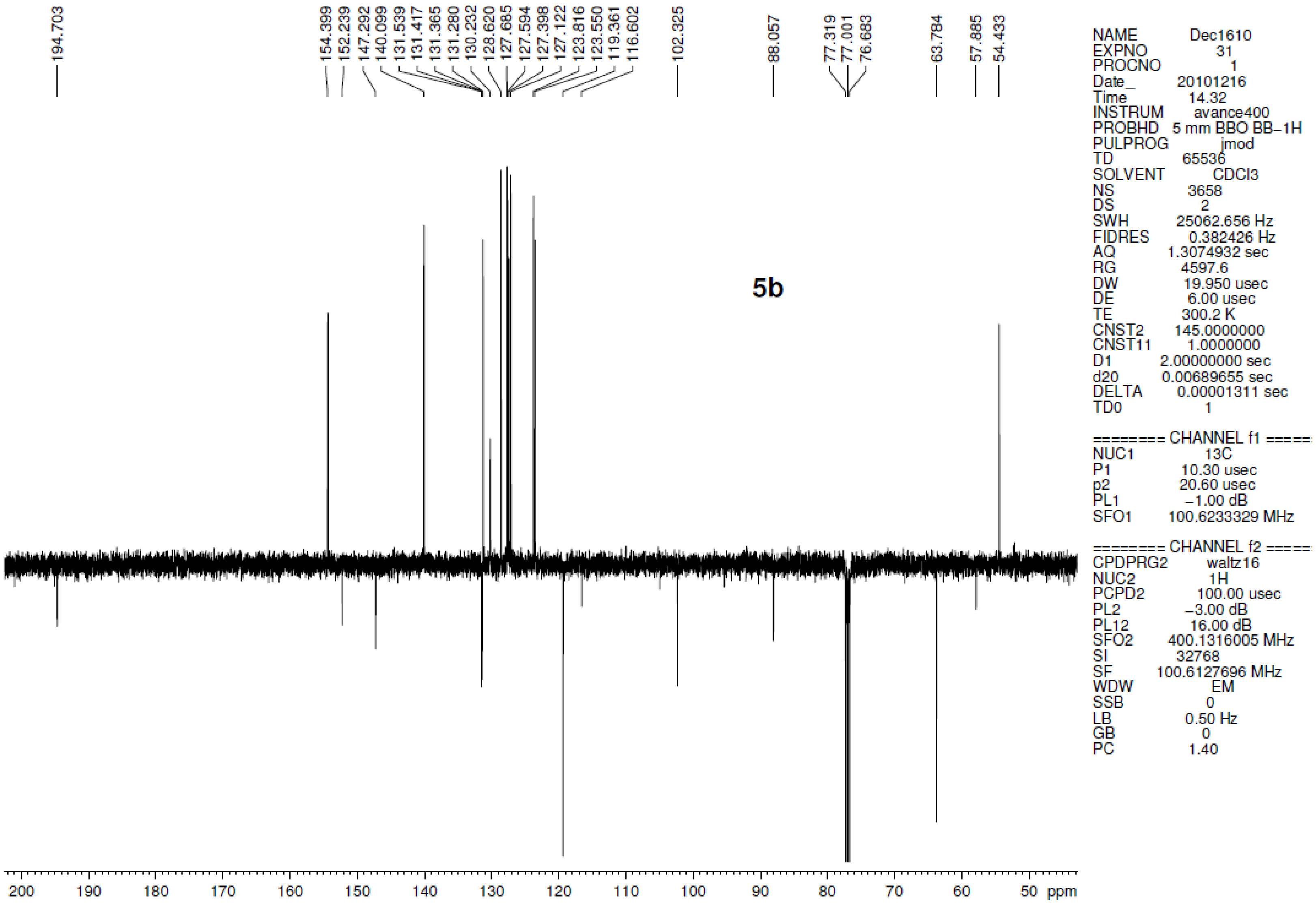
194.703
154.399
152.239
147.292
140.099
131.539
131.417
131.365
131.280
130.232
128.620
127.685
127.594
127.398
127.122
123.816
123.550
119.361
116.602
102.325
88.057
77.319
77.001
76.683
63.784
57.885
54.433
5b
NAME Dec1610
EXPNO 31
PROCNO 1
Date_ 20101216
Time 14.32
INSTRUM avance400
PROBHD 5 mm BBO BB-1H
PULPROG jmod
TD 65536
SOLVENT CDCl3
NS 3658
DS 2
SWH 25062.656 Hz
FIDRES 0.382426 Hz
AQ 1.3074932 sec
RG 4597.6
DW 19.950 usec
DE 6.00 usec
TE 300.2 K
CNST2 145.0000000
CNST11 1.0000000
D1 2.00000000 sec
d20 0.00689655 sec
DELTA 0.00001311 sec
TD0 1
======== CHANNEL f1 =====
NUC1 13C
P1 10.30 usec
p2 20.60 usec
PL1 -1.00 dB
SFO1 100.6233329 MHz
======== CHANNEL f2 =====
CPDPRG2 waltz16
NUC2 1H
PCPD2 100.00 usec
PL2 -3.00 dB
PL12 16.00 dB
SFO2 400.1316005 MHz
SI 32768
SF 100.6127696 MHz
WDW EM
SSB 0
LB 0.50 Hz
GB 0
PC 1.40
200 190 180 170 160 150 140 130 120 110 100 90 80 70 60 50 ppm

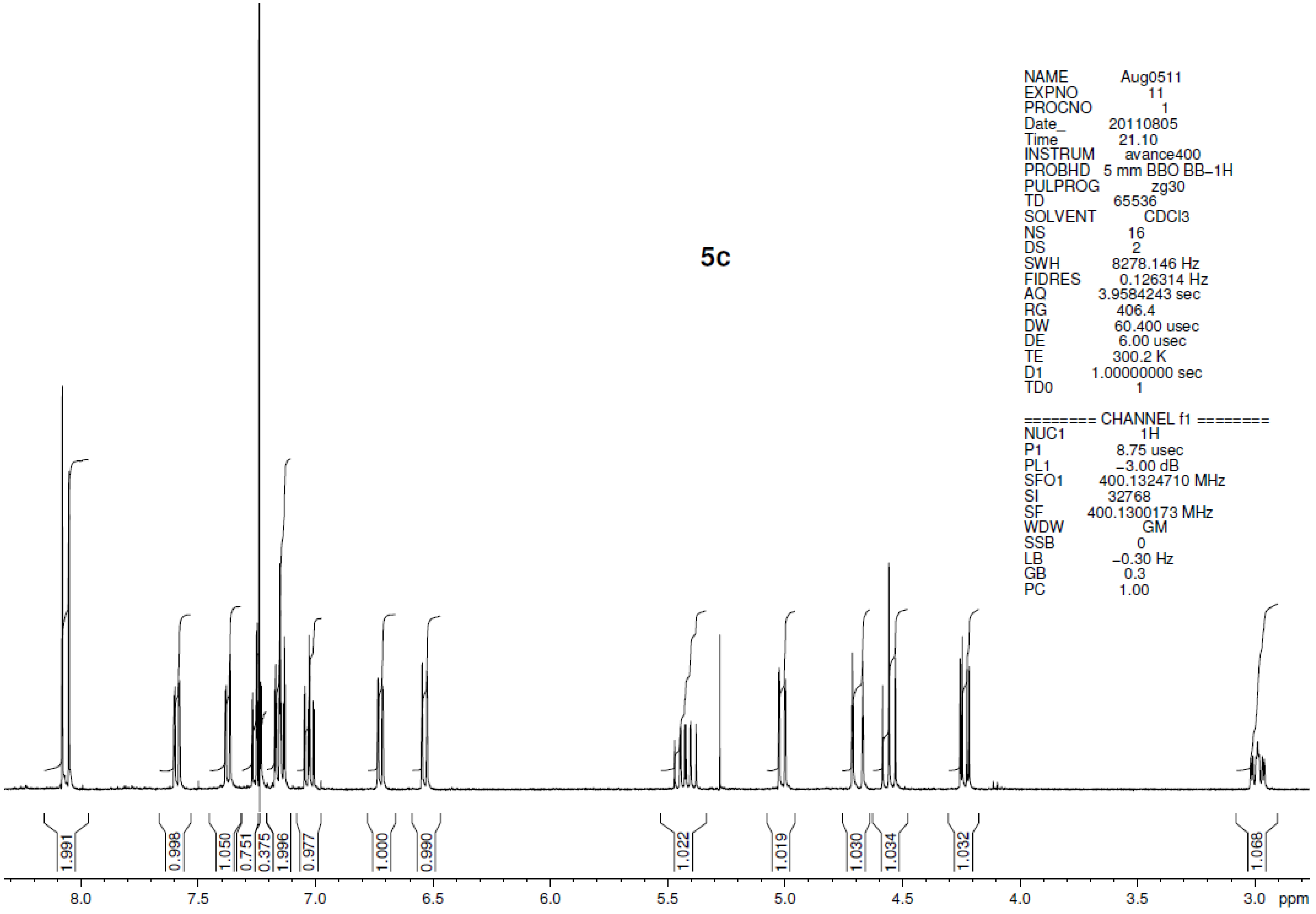
5c
NAME Aug0511
EXPNO 11
PROCNO 1
Date_ 20110805
Time 21.10
INSTRUM avance400
PROBHD 5 mm BBO BB-1H
PULPROG zg30
TD 65536
SOLVENT CDCl3
NS 16
DS 2
SWH 8278.146 Hz
FIDRES 0.126314 Hz
AQ 3.9584243 sec
RG 406.4
DW 60.400 usec
DE 6.00 usec
TE 300.2 K
D1 1.00000000 sec
TD0 1
======== CHANNEL f1 ========
NUC1 1H
P1 8.75 usec
PL1 -3.00 dB
SFO1 400.1324710 MHz
SI 32768
SF 400.1300173 MHz
WDW GM
SSB 0
LB -0.30 Hz
GB 0.3
PC 1.00
1.991
0.998
1.050
0.751
0.375
1.996
0.977
1.000
0.990
1.022
1.019
1.030
1.034
1.032
1.068
8.0
7.5
7.0
6.5
6.0
5.5
5.0
4.5
4.0
3.5
3.0
ppm

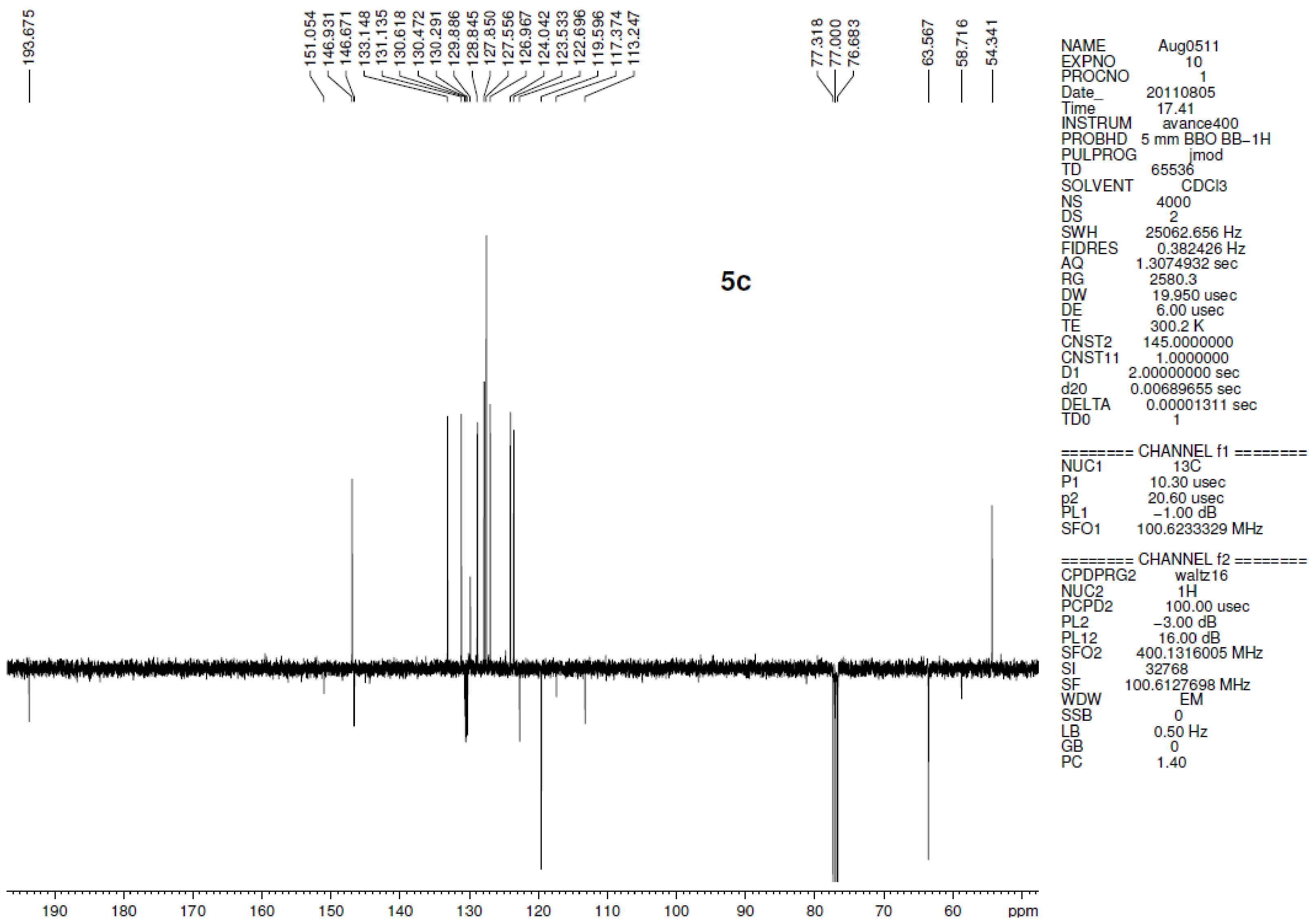
193.675
151.054
146.931
146.671
133.148
131.135
130.618
130.472
130.291
129.886
128.845
127.850
127.556
126.967
124.042
123.533
122.696
119.596
117.374
113.247
77.318
77.000
76.683
63.567
58.716
54.341
5c
190 180 170 160 150 140 130 120 110 100 90 80 70 60 ppm
NAME Aug0511
EXPNO 10
PROCNO 1
Date_ 20110805
Time 17.41
INSTRUM avance400
PROBHD 5 mm BBO BB-1H
PULPROG jmod
TD 65536
SOLVENT CDCl3
NS 4000
DS 2
SWH 25062.656 Hz
FIDRES 0.382426 Hz
AQ 1.3074932 sec
RG 2580.3
DW 19.950 usec
DE 6.00 usec
TE 300.2 K
CNST2 145.0000000
CNST11 1.0000000
D1 2.00000000 sec
d20 0.00689655 sec
DELTA 0.00001311 sec
TD0 1
======== CHANNEL f1 ========
NUC1 13C
P1 10.30 usec
p2 20.60 usec
PL1 -1.00 dB
SFO1 100.6233329 MHz
======== CHANNEL f2 ========
CPDPRG2 waltz16
NUC2 1H
PCPD2 100.00 usec
PL2 -3.00 dB
PL12 16.00 dB
SFO2 400.1316005 MHz
SI 32768
SF 100.6127698 MHz
WDW EM
SSB 0
LB 0.50 Hz
GB 0
PC 1.40

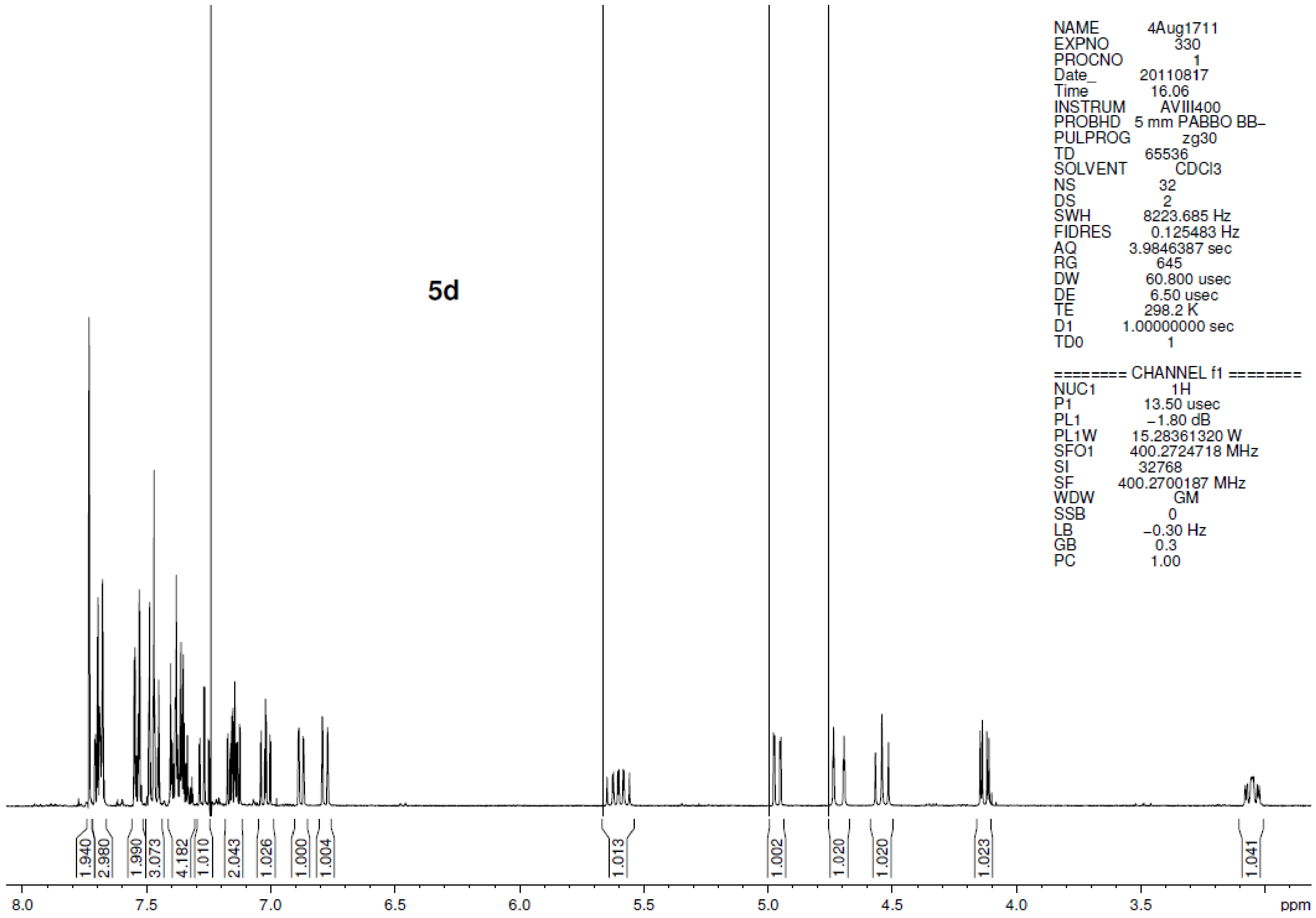
5d
NAME 4Aug1711
EXPNO 330
PROCNO 1
Date_ 20110817
Time 16.06
INSTRUM AVIII400
PROBHD 5 mm PABBO BB-
PULPROG zg30
TD 65536
SOLVENT CDCl3
NS 32
DS 2
SWH 8223.685 Hz
FIDRES 0.125483 Hz
AQ 3.9846387 sec
RG 645
DW 60.800 usec
DE 6.50 usec
TE 298.2 K
D1 1.00000000 sec
TD0 1
======== CHANNEL f1 ========
NUC1 1H
P1 13.50 usec
PL1 -1.80 dB
PL1W 15.28361320 W
SFO1 400.2724718 MHz
SI 32768
SF 400.2700187 MHz
WDW GM
SSB 0
LB -0.30 Hz
GB 0.3
PC 1.00
1.940
2.980
1.990
3.073
4.182
1.010
2.043
1.026
1.000
1.004
1.013
1.002
1.020
1.020
1.023
1.041
8.0
7.5
7.0
6.5
6.0
5.5
5.0
4.5
4.0
3.5
ppm

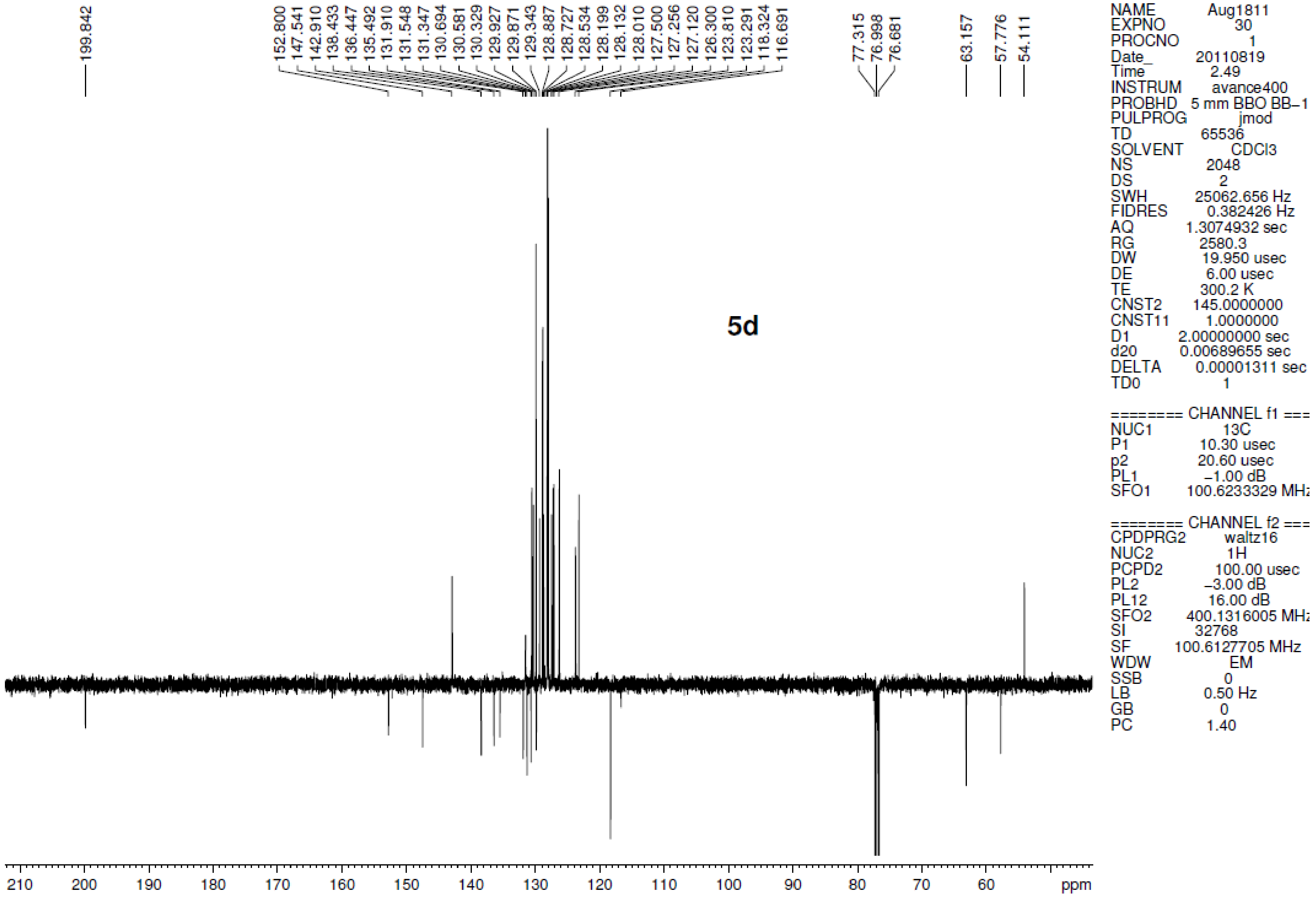
199.842
152.800
147.541
142.910
138.433
136.447
135.492
131.910
131.548
131.347
130.694
130.581
130.329
129.927
129.871
129.343
128.887
128.727
128.534
128.199
128.132
128.010
127.500
127.256
127.120
126.300
123.810
123.291
118.324
116.691
77.315
76.998
76.681
63.157
57.776
54.111
5d
210 200 190 180 170 160 150 140 130 120 110 100 90 80 70 60 ppm
NAME Aug1811
EXPNO 30
PROCNO 1
Date_ 20110819
Time 2.49
INSTRUM avance400
PROBHD 5 mm BBO BB-1
PULPROG jmod
TD 65536
SOLVENT CDCl3
NS 2048
DS 2
SWH 25062.656 Hz
FIDRES 0.382426 Hz
AQ 1.3074932 sec
RG 2580.3
DW 19.950 usec
DE 6.00 usec
TE 300.2 K
CNST2 145.0000000
CNST11 1.0000000
D1 2.00000000 sec
d20 0.00689655 sec
DELTA 0.00001311 sec
TD0 1
======== CHANNEL f1 ===
NUC1 13C
P1 10.30 usec
p2 20.60 usec
PL1 -1.00 dB
SFO1 100.6233329 MHz
======== CHANNEL f2 ===
CPDPRG2 waltz16
NUC2 1H
PCPD2 100.00 usec
PL2 -3.00 dB
PL12 16.00 dB
SFO2 400.1316005 MHz
SI 32768
SF 100.6127705 MHz
WDW EM
SSB 0
LB 0.50 Hz
GB 0
PC 1.40

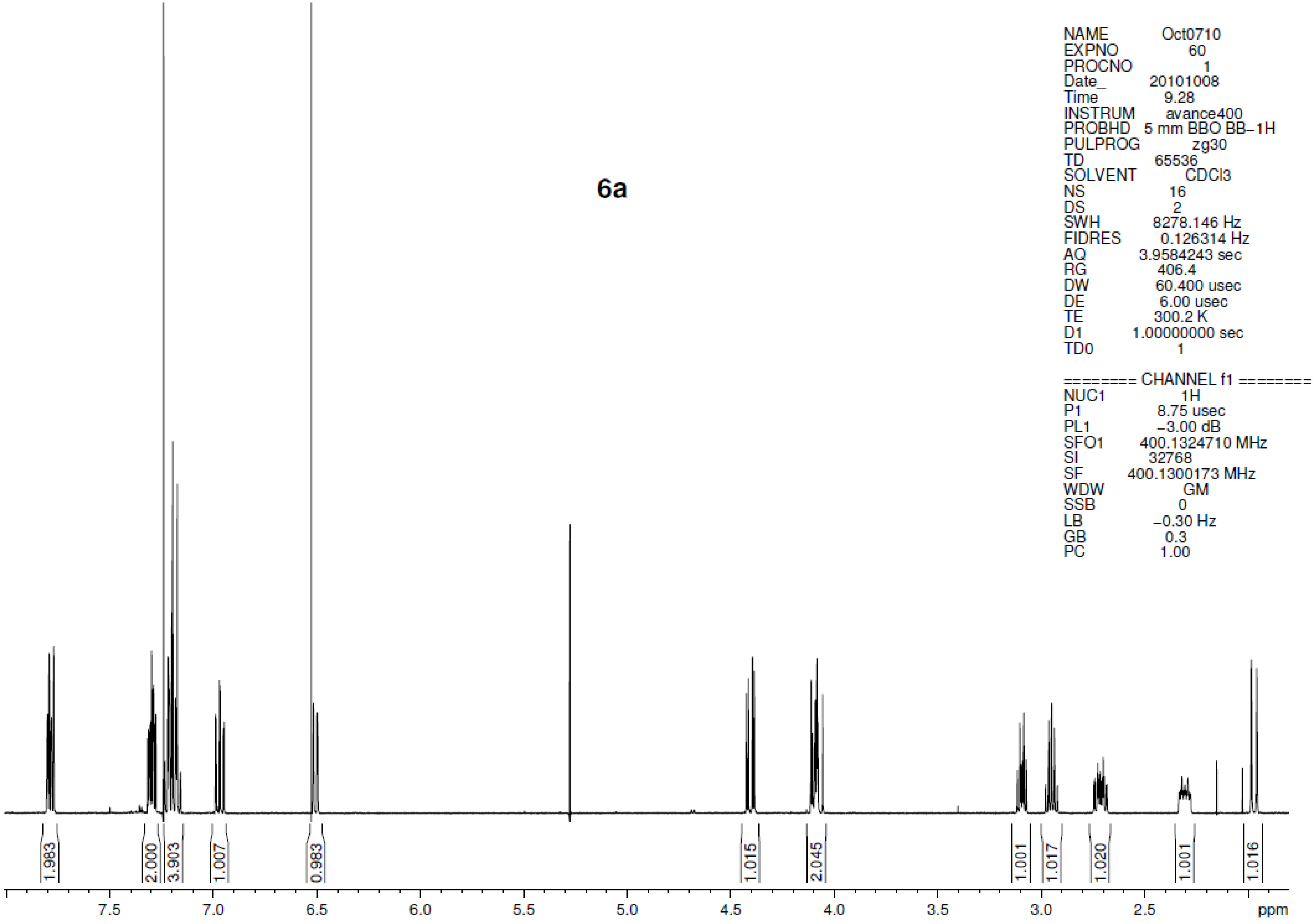
6a
NAME Oct0710
EXPNO 60
PROCNO 1
Date_ 20101008
Time 9.28
INSTRUM avance400
PROBHD 5 mm BBO BB-1H
PULPROG zg30
TD 65536
SOLVENT CDCl3
NS 16
DS 2
SWH 8278.146 Hz
FIDRES 0.126314 Hz
AQ 3.9584243 sec
RG 406.4
DW 60.400 usec
DE 6.00 usec
TE 300.2 K
D1 1.00000000 sec
TD0 1
======== CHANNEL f1 ========
NUC1 1H
P1 8.75 usec
PL1 -3.00 dB
SFO1 400.1324710 MHz
SI 32768
SF 400.1300173 MHz
WDW GM
SSB 0
LB -0.30 Hz
GB 0.3
PC 1.00
1.983
2.000
3.903
1.007
0.983
1.015
2.045
1.001
1.017
1.020
1.001
1.016
7.5
7.0
6.5
6.0
5.5
5.0
4.5
4.0
3.5
3.0
2.5
ppm

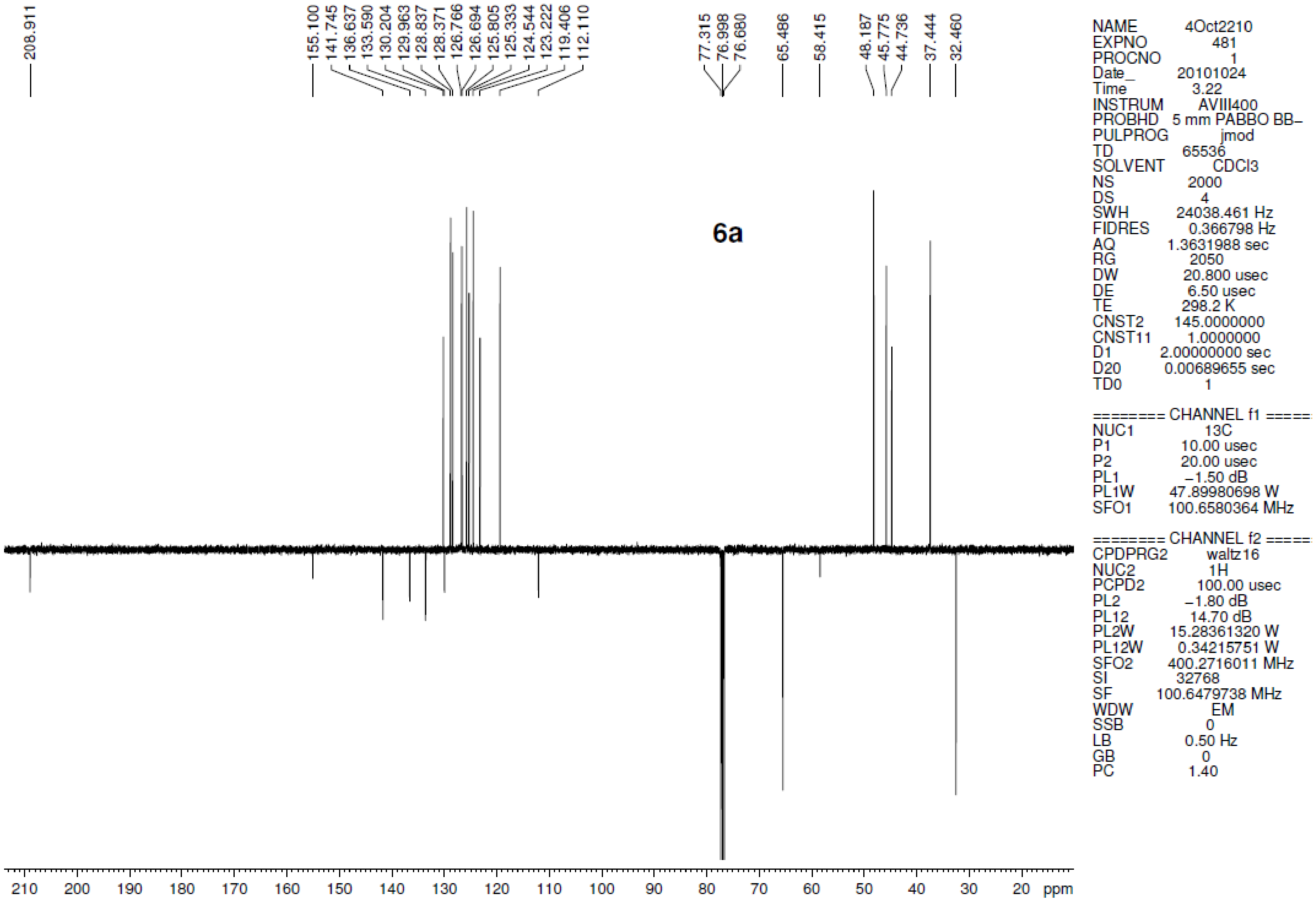
208.911
155.100
141.745
136.637
133.590
130.204
129.963
128.837
128.371
126.766
126.694
125.805
125.333
124.544
123.222
119.406
112.110
77.315
76.998
76.680
65.486
58.415
48.187
45.775
44.736
37.444
32.460
6a
210 200 190 180 170 160 150 140 130 120 110 100 90 80 70 60 50 40 30 20 ppm
NAME 4Oct2210
EXPNO 481
PROCNO 1
Date_ 20101024
Time 3.22
INSTRUM AVIII400
PROBHD 5 mm PABBO BB–
PULPROG jmod
TD 65536
SOLVENT CDCl3
NS 2000
DS 4
SWH 24038.461 Hz
FIDRES 0.366798 Hz
AQ 1.3631988 sec
RG 2050
DW 20.800 usec
DE 6.50 usec
TE 298.2 K
CNST2 145.0000000
CNST11 1.0000000
D1 2.00000000 sec
D20 0.00689655 sec
TD0 1
======== CHANNEL f1 =====
NUC1 13C
P1 10.00 usec
P2 20.00 usec
PL1 –1.50 dB
PL1W 47.89980698 W
SFO1 100.6580364 MHz
======== CHANNEL f2 =====
CPDPRG2 waltz16
NUC2 1H
PCPD2 100.00 usec
PL2 –1.80 dB
PL12 14.70 dB
PL2W 15.28361320 W
PL12W 0.34215751 W
SFO2 400.2716011 MHz
SI 32768
SF 100.6479738 MHz
WDW EM
SSB 0
LB 0.50 Hz
GB 0
PC 1.40

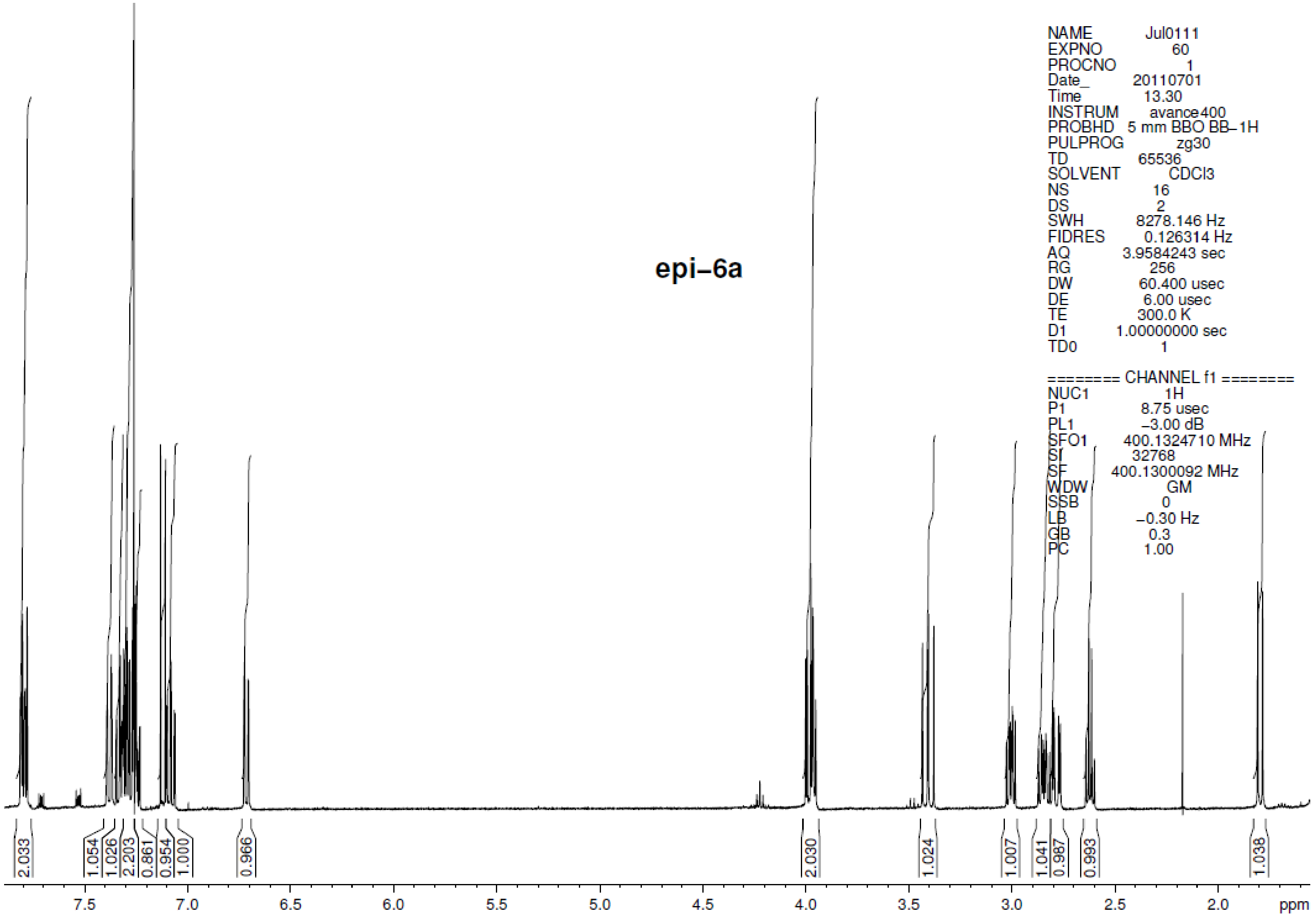
epi–6a
NAME Jul0111
EXPNO 60
PROCNO 1
Date_ 20110701
Time 13.30
INSTRUM avance400
PROBHD 5 mm BBO BB-1H
PULPROG zg30
TD 65536
SOLVENT CDCl3
NS 16
DS 2
SWH 8278.146 Hz
FIDRES 0.126314 Hz
AQ 3.9584243 sec
RG 256
DW 60.400 usec
DE 6.00 usec
TE 300.0 K
D1 1.00000000 sec
TD0 1
======== CHANNEL f1 ========
NUC1 1H
P1 8.75 usec
PL1 -3.00 dB
SFO1 400.1324710 MHz
SI 32768
SF 400.1300092 MHz
WDW GM
SSB 0
LB -0.30 Hz
GB 0.3
PC 1.00
2.033
1.054
1.026
2.203
0.861
0.954
1.000
0.966
2.030
1.024
1.007
1.041
0.987
0.993
1.038
7.5
7.0
6.5
6.0
5.5
5.0
4.5
4.0
3.5
3.0
2.5
2.0
ppm

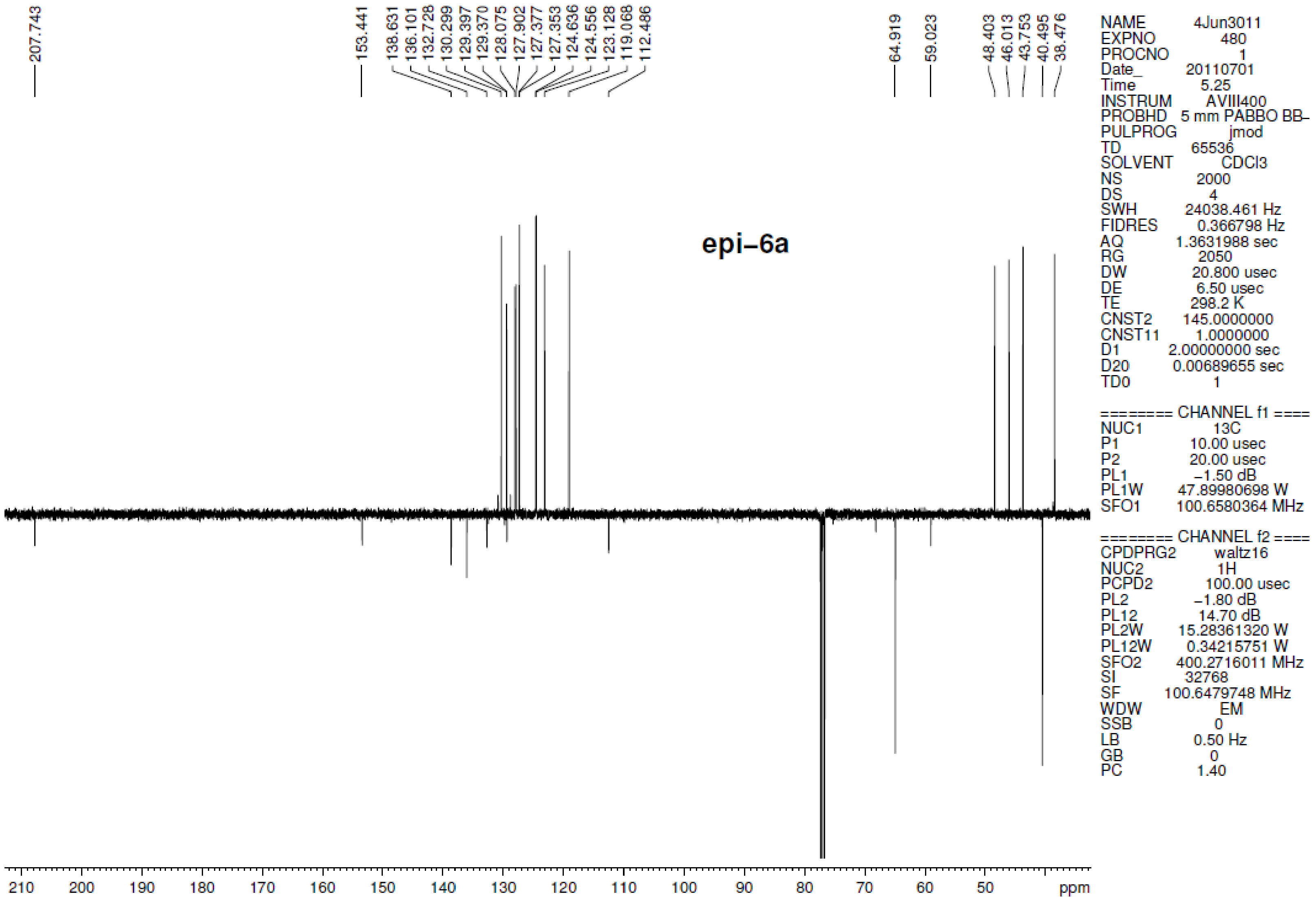

207.743
153.441
138.631
136.101
132.728
130.299
129.397
129.370
128.075
127.902
127.377
127.353
124.636
124.556
123.128
119.068
112.486
64.919
59.023
48.403
46.013
43.753
40.495
38.476
epi-6a
NAME 4Jun3011
EXPNO 480
PROCNO 1
Date_ 20110701
Time 5.25
INSTRUM AVIII400
PROBHD 5 mm PABBO BB-
PULPROG jmod
TD 65536
SOLVENT CDCl3
NS 2000
DS 4
SWH 24038.461 Hz
FIDRES 0.366798 Hz
AQ 1.3631988 sec
RG 2050
DW 20.800 usec
DE 6.50 usec
TE 298.2 K
CNST2 145.0000000
CNST11 1.0000000
D1 2.00000000 sec
D20 0.00689655 sec
TD0 1
======== CHANNEL f1 ====
NUC1 13C
P1 10.00 usec
P2 20.00 usec
PL1 -1.50 dB
PL1W 47.89980698 W
SFO1 100.6580364 MHz
======== CHANNEL f2 ====
CPDPRG2 waltz16
NUC2 1H
PCPD2 100.00 usec
PL2 -1.80 dB
PL12 14.70 dB
PL2W 15.28361320 W
PL12W 0.34215751 W
SFO2 400.2716011 MHz
SI 32768
SF 100.6479748 MHz
WDW EM
SSB 0
LB 0.50 Hz
GB 0
PC 1.40
210 200 190 180 170 160 150 140 130 120 110 100 90 80 70 60 50 ppm

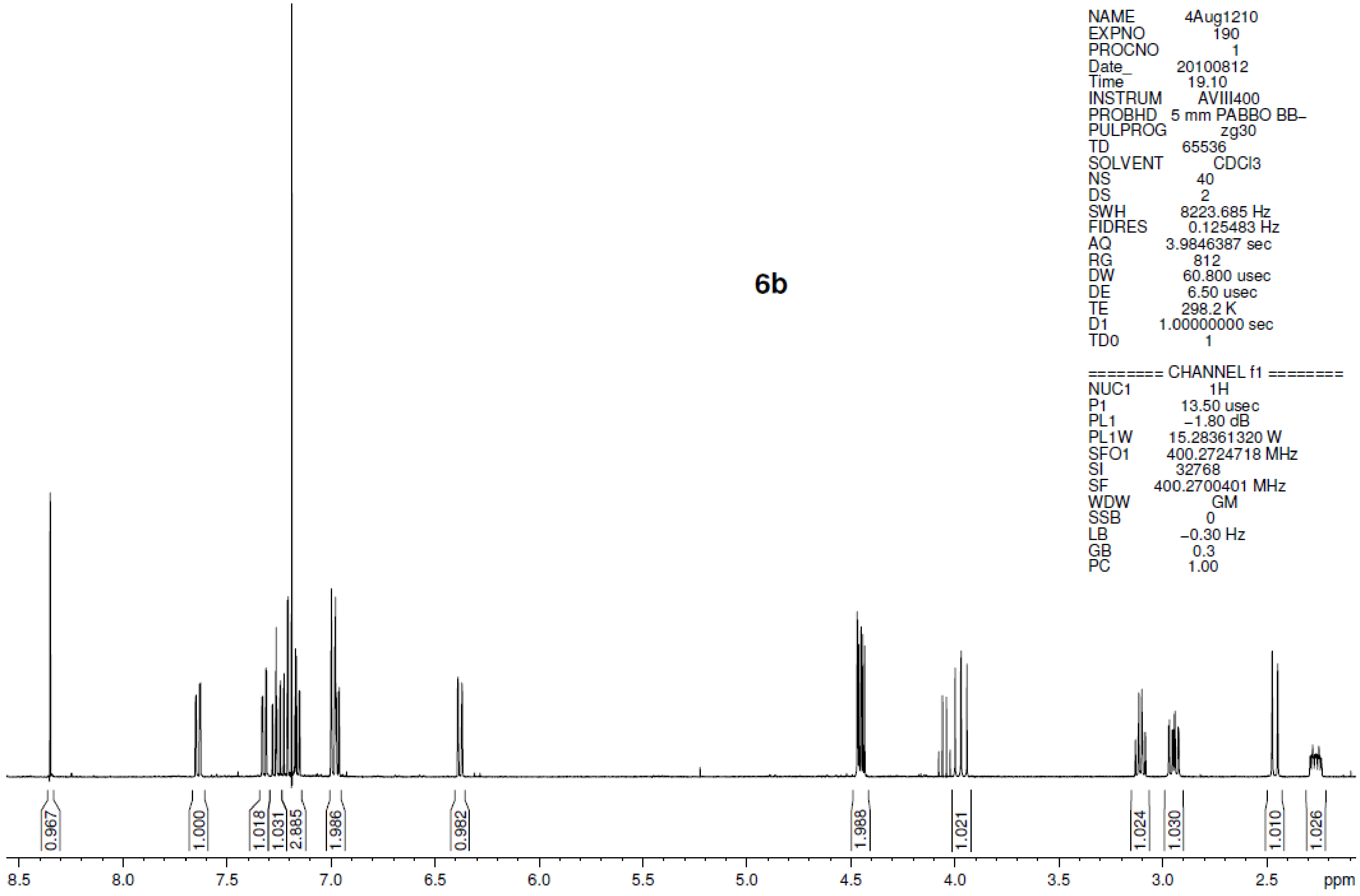

6b
NAME 4Aug1210
EXPNO 190
PROCNO 1
Date_ 20100812
Time 19.10
INSTRUM AVIII400
PROBHD 5 mm PABBO BB-
PULPROG zg30
TD 65536
SOLVENT CDCl3
NS 40
DS 2
SWH 8223.685 Hz
FIDRES 0.125483 Hz
AQ 3.9846387 sec
RG 812
DW 60.800 usec
DE 6.50 usec
TE 298.2 K
D1 1.00000000 sec
TD0 1
======== CHANNEL f1 ========
NUC1 1H
P1 13.50 usec
PL1 -1.80 dB
PL1W 15.28361320 W
SFO1 400.2724718 MHz
SI 32768
SF 400.2700401 MHz
WDW GM
SSB 0
LB -0.30 Hz
GB 0.3
PC 1.00
0.967
1.000
1.018
1.031
2.885
1.986
0.982
1.988
1.021
1.024
1.030
1.010
1.026
8.5
8.0
7.5
7.0
6.5
6.0
5.5
5.0
4.5
4.0
3.5
3.0
2.5
ppm

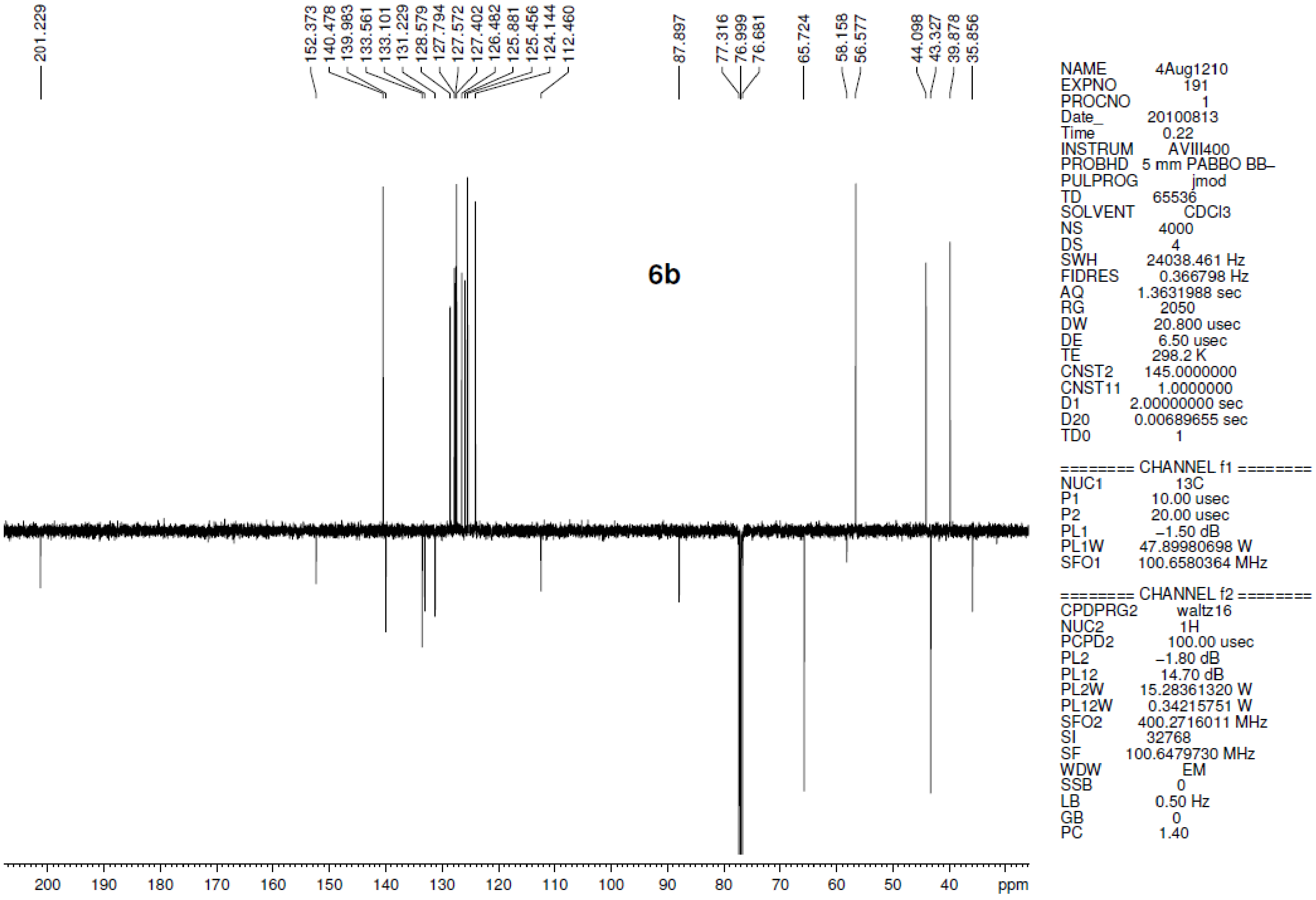
201.229
152.373
140.478
139.983
133.561
133.101
131.229
128.579
127.794
127.572
127.402
126.482
125.881
125.456
124.144
112.460
87.897
77.316
76.999
76.681
65.724
58.158
56.577
44.098
43.327
39.878
35.856
6b
200 190 180 170 160 150 140 130 120 110 100 90 80 70 60 50 40 ppm
NAME 4Aug1210
EXPNO 191
PROCNO 1
Date_ 20100813
Time 0.22
INSTRUM AVIII400
PROBHD 5 mm PABBO BB-
PULPROG jmod
TD 65536
SOLVENT CDCl3
NS 4000
DS 4
SWH 24038.461 Hz
FIDRES 0.366798 Hz
AQ 1.3631988 sec
RG 2050
DW 20.800 usec
DE 6.50 usec
TE 298.2 K
CNST2 145.0000000
CNST11 1.0000000
D1 2.00000000 sec
D20 0.00689655 sec
TD0 1
======== CHANNEL f1 ========
NUC1 13C
P1 10.00 usec
P2 20.00 usec
PL1 -1.50 dB
PL1W 47.89980698 W
SFO1 100.6580364 MHz
======== CHANNEL f2 ========
CPDPRG2 waltz16
NUC2 1H
PCPD2 100.00 usec
PL2 -1.80 dB
PL12 14.70 dB
PL2W 15.28361320 W
PL12W 0.34215751 W
SFO2 400.2716011 MHz
SI 32768
SF 100.6479730 MHz
WDW EM
SSB 0
LB 0.50 Hz
GB 0
PC 1.40

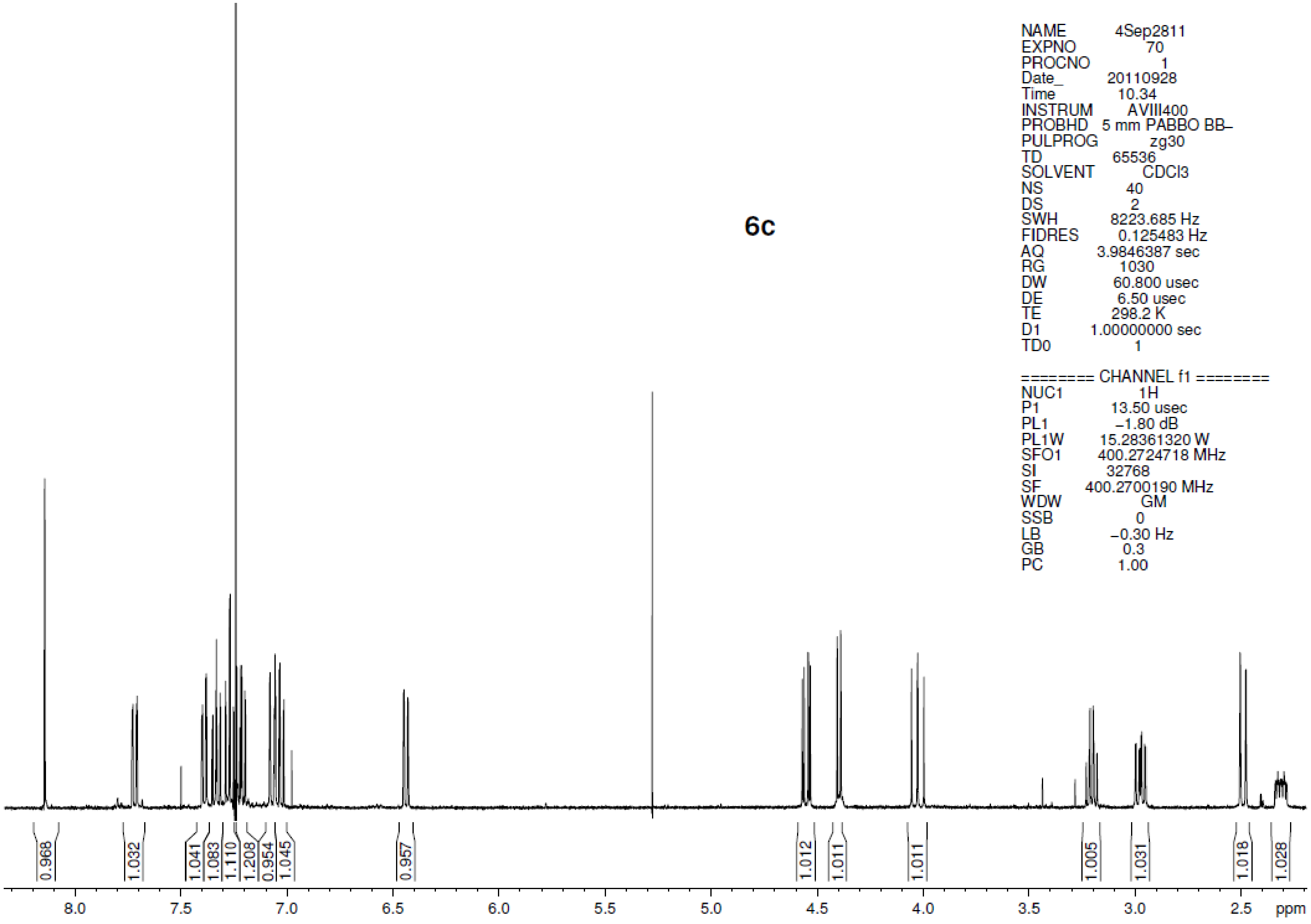
6c
NAME 4Sep2811
EXPNO 70
PROCNO 1
Date_ 20110928
Time 10.34
INSTRUM AVIII400
PROBHD 5 mm PABBO BB-
PULPROG zg30
TD 65536
SOLVENT CDCl3
NS 40
DS 2
SWH 8223.685 Hz
FIDRES 0.125483 Hz
AQ 3.9846387 sec
RG 1030
DW 60.800 usec
DE 6.50 usec
TE 298.2 K
D1 1.00000000 sec
TD0 1
======== CHANNEL f1 ========
NUC1 1H
P1 13.50 usec
PL1 -1.80 dB
PL1W 15.28361320 W
SFO1 400.2724718 MHz
SI 32768
SF 400.2700190 MHz
WDW GM
SSB 0
LB -0.30 Hz
GB 0.3
PC 1.00
0.968
1.032
1.041
1.083
1.110
1.208
0.954
1.045
0.957
1.012
1.011
1.011
1.005
1.031
1.018
1.028
8.0
7.5
7.0
6.5
6.0
5.5
5.0
4.5
4.0
3.5
3.0
2.5
ppm

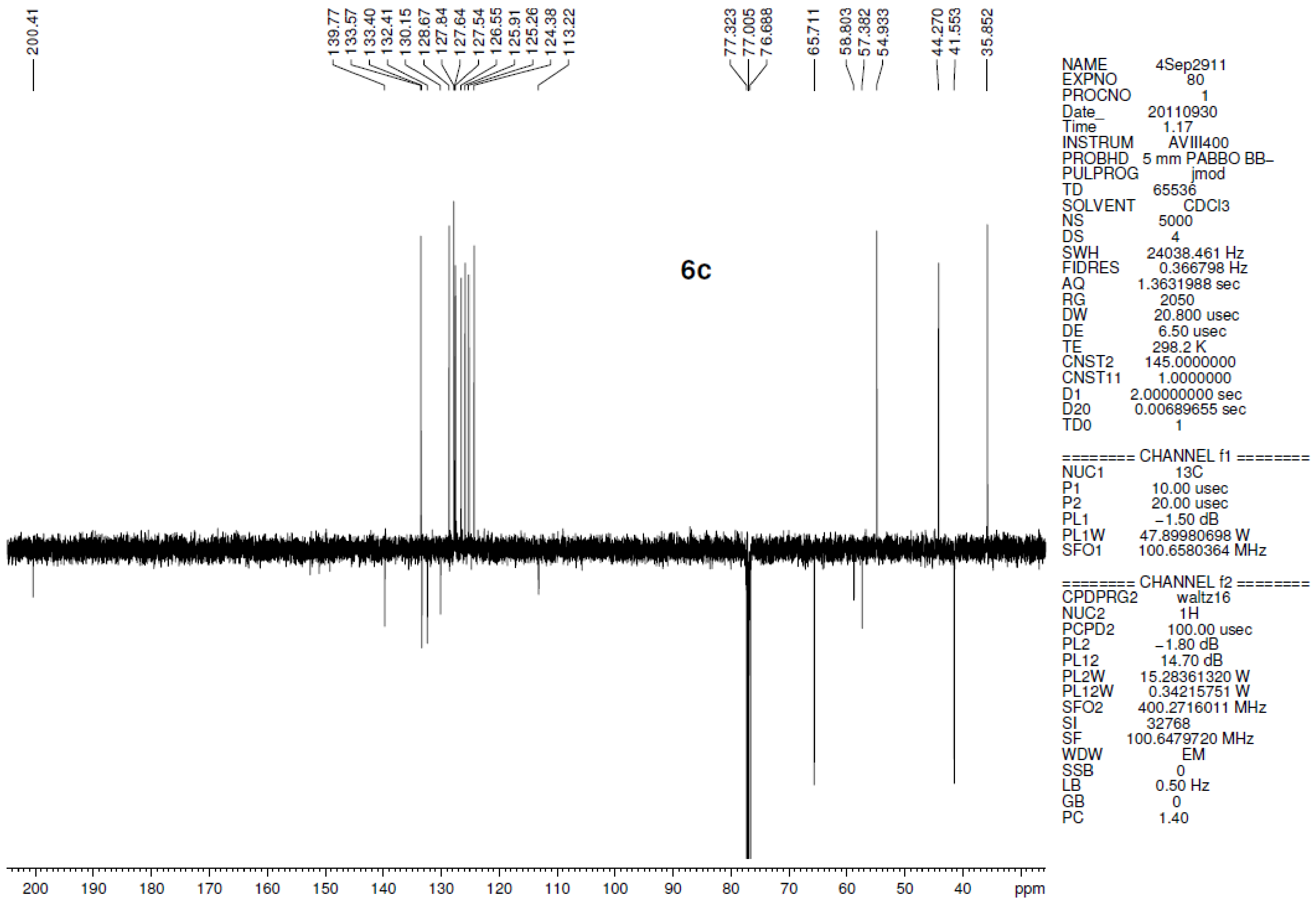
200.41
139.77
133.57
133.40
132.41
130.15
128.67
127.84
127.64
127.54
126.55
125.91
125.26
124.38
113.22
77.323
77.005
76.688
65.711
58.803
57.382
54.933
44.270
41.553
35.852
6c
200 190 180 170 160 150 140 130 120 110 100 90 80 70 60 50 40 ppm
NAME 4Sep2911
EXPNO 80
PROCNO 1
Date_ 20110930
Time 1.17
INSTRUM AVIII400
PROBHD 5 mm PABBO BB-
PULPROG jmod
TD 65536
SOLVENT CDCl3
NS 5000
DS 4
SWH 24038.461 Hz
FIDRES 0.366798 Hz
AQ 1.3631988 sec
RG 2050
DW 20.800 usec
DE 6.50 usec
TE 298.2 K
CNST2 145.0000000
CNST11 1.0000000
D1 2.00000000 sec
D20 0.00689655 sec
TD0 1
======== CHANNEL f1 ========
NUC1 13C
P1 10.00 usec
P2 20.00 usec
PL1 -1.50 dB
PL1W 47.89980698 W
SFO1 100.6580364 MHz
======== CHANNEL f2 ========
CPDPRG2 waltz16
NUC2 1H
PCPD2 100.00 usec
PL2 -1.80 dB
PL12 14.70 dB
PL2W 15.28361320 W
PL12W 0.34215751 W
SFO2 400.2716011 MHz
SI 32768
SF 100.6479720 MHz
WDW EM
SSB 0
LB 0.50 Hz
GB 0
PC 1.40

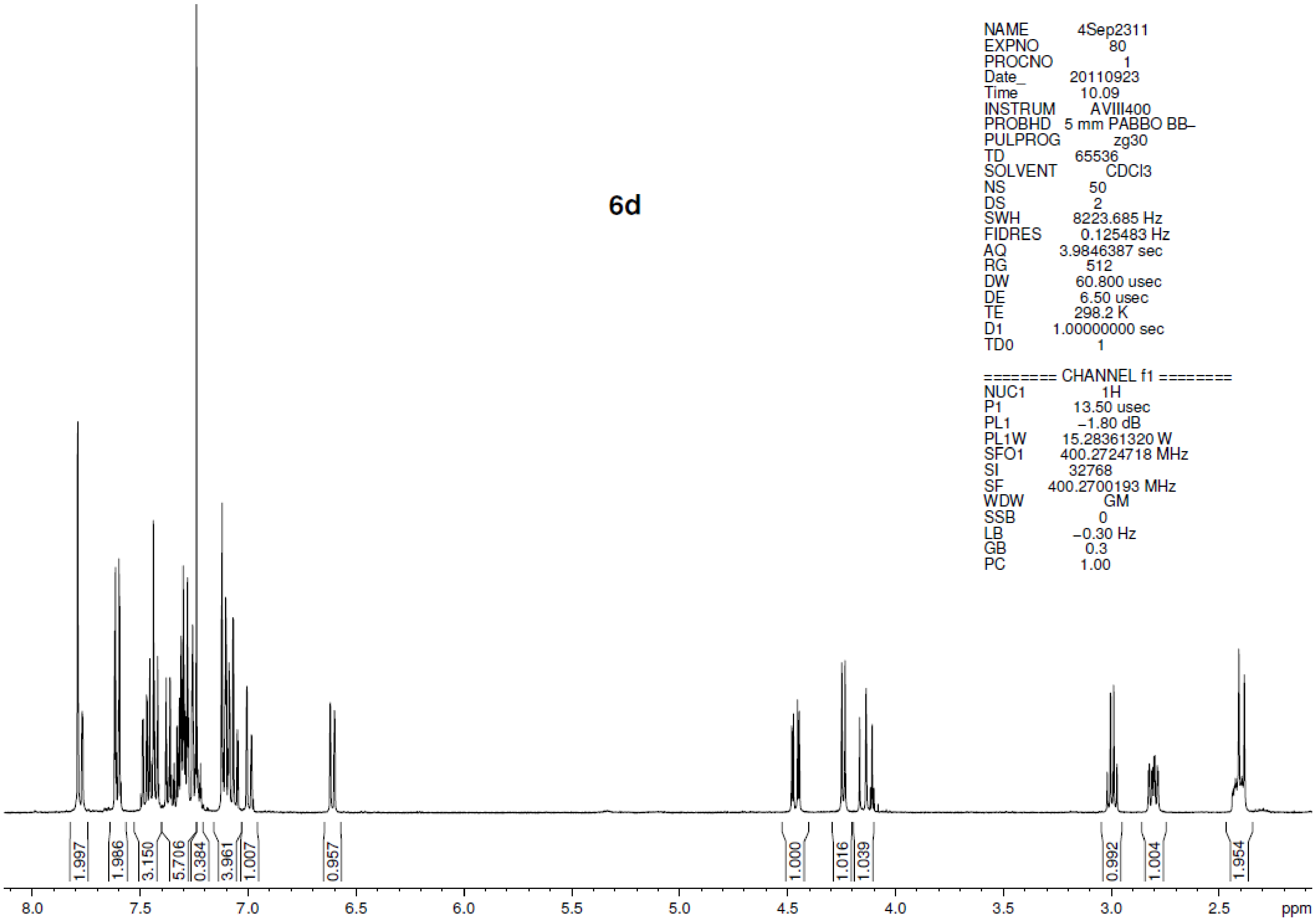
6d
NAME 4Sep2311
EXPNO 80
PROCNO 1
Date_ 20110923
Time 10.09
INSTRUM AVIII400
PROBHD 5 mm PABBO BB-
PULPROG zg30
TD 65536
SOLVENT CDCl3
NS 50
DS 2
SWH 8223.685 Hz
FIDRES 0.125483 Hz
AQ 3.9846387 sec
RG 512
DW 60.800 usec
DE 6.50 usec
TE 298.2 K
D1 1.00000000 sec
TD0 1
======== CHANNEL f1 ========
NUC1 1H
P1 13.50 usec
PL1 -1.80 dB
PL1W 15.28361320 W
SFO1 400.2724718 MHz
SI 32768
SF 400.2700193 MHz
WDW GM
SSB 0
LB -0.30 Hz
GB 0.3
PC 1.00
1.997
1.986
3.150
5.706
0.384
3.961
1.007
0.957
1.000
1.016
1.039
0.992
1.004
1.954
8.0
7.5
7.0
6.5
6.0
5.5
5.0
4.5
4.0
3.5
3.0
2.5
ppm

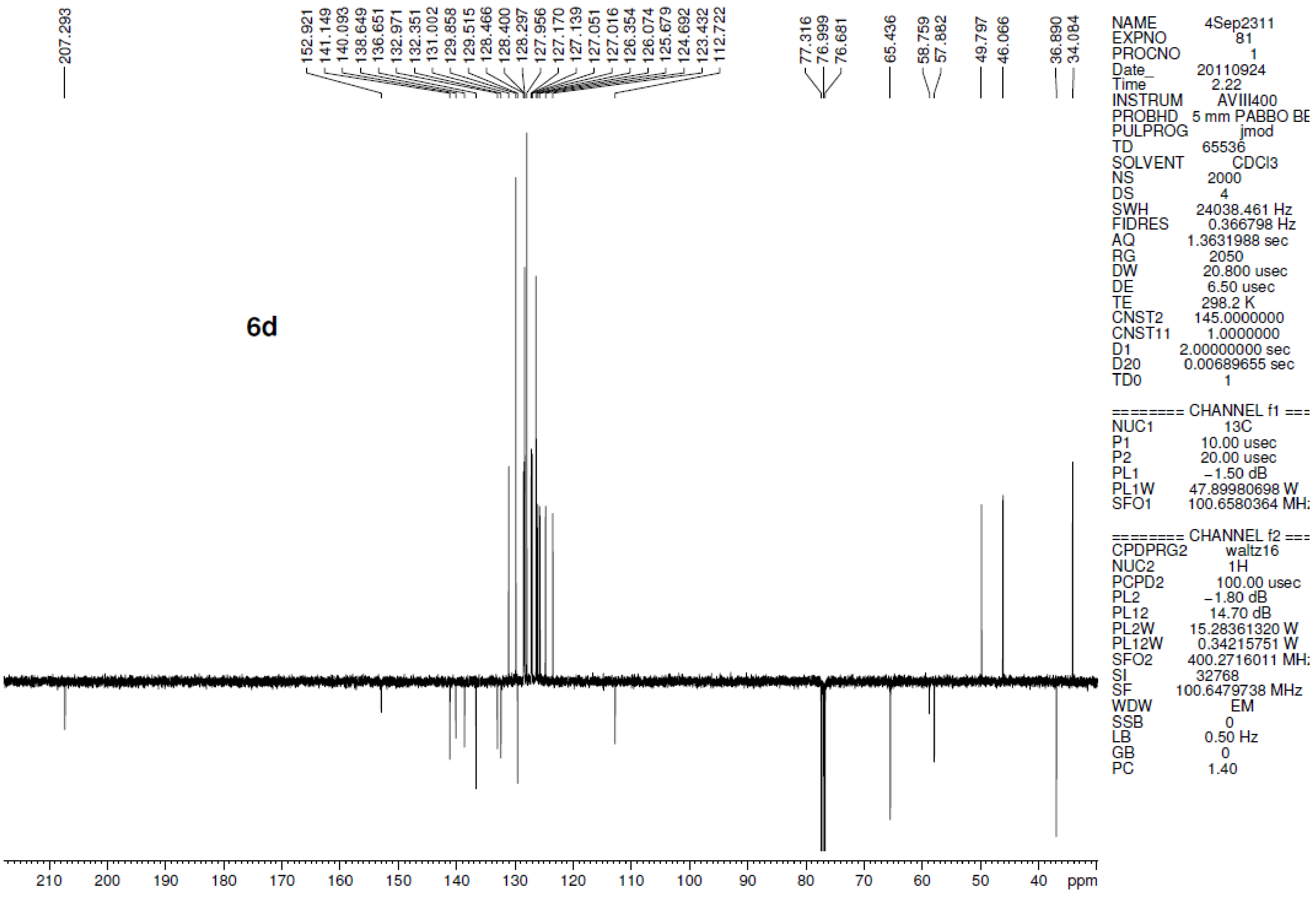
207.293
152.921
141.149
140.093
138.649
136.651
132.971
132.351
131.002
129.858
129.515
128.466
128.400
128.297
127.956
127.170
127.139
127.051
127.016
126.354
126.074
125.679
124.692
123.432
112.722
77.316
76.999
76.681
65.436
58.759
57.882
49.797
46.066
36.890
34.084
6d
210 200 190 180 170 160 150 140 130 120 110 100 90 80 70 60 50 40 ppm
NAME 4Sep2311
EXPNO 81
PROCNO 1
Date_ 20110924
Time 2.22
INSTRUM AVIII400
PROBHD 5 mm PABBO BB
PULPROG jmod
TD 65536
SOLVENT CDCl3
NS 2000
DS 4
SWH 24038.461 Hz
FIDRES 0.366798 Hz
AQ 1.3631988 sec
RG 2050
DW 20.800 usec
DE 6.50 usec
TE 298.2 K
CNST2 145.0000000
CNST11 1.0000000
D1 2.00000000 sec
D20 0.00689655 sec
TD0 1
======== CHANNEL f1 ===
NUC1 13C
P1 10.00 usec
P2 20.00 usec
PL1 -1.50 dB
PL1W 47.89980698 W
SFO1 100.6580364 MH
======== CHANNEL f2 ===
CPDPRG2 waltz16
NUC2 1H
PCPD2 100.00 usec
PL2 -1.80 dB
PL12 14.70 dB
PL2W 15.28361320 W
PL12W 0.34215751 W
SFO2 400.2716011 MH
SI 32768
SF 100.6479738 MHz
WDW EM
SSB 0
LB 0.50 Hz
GB 0
PC 1.40

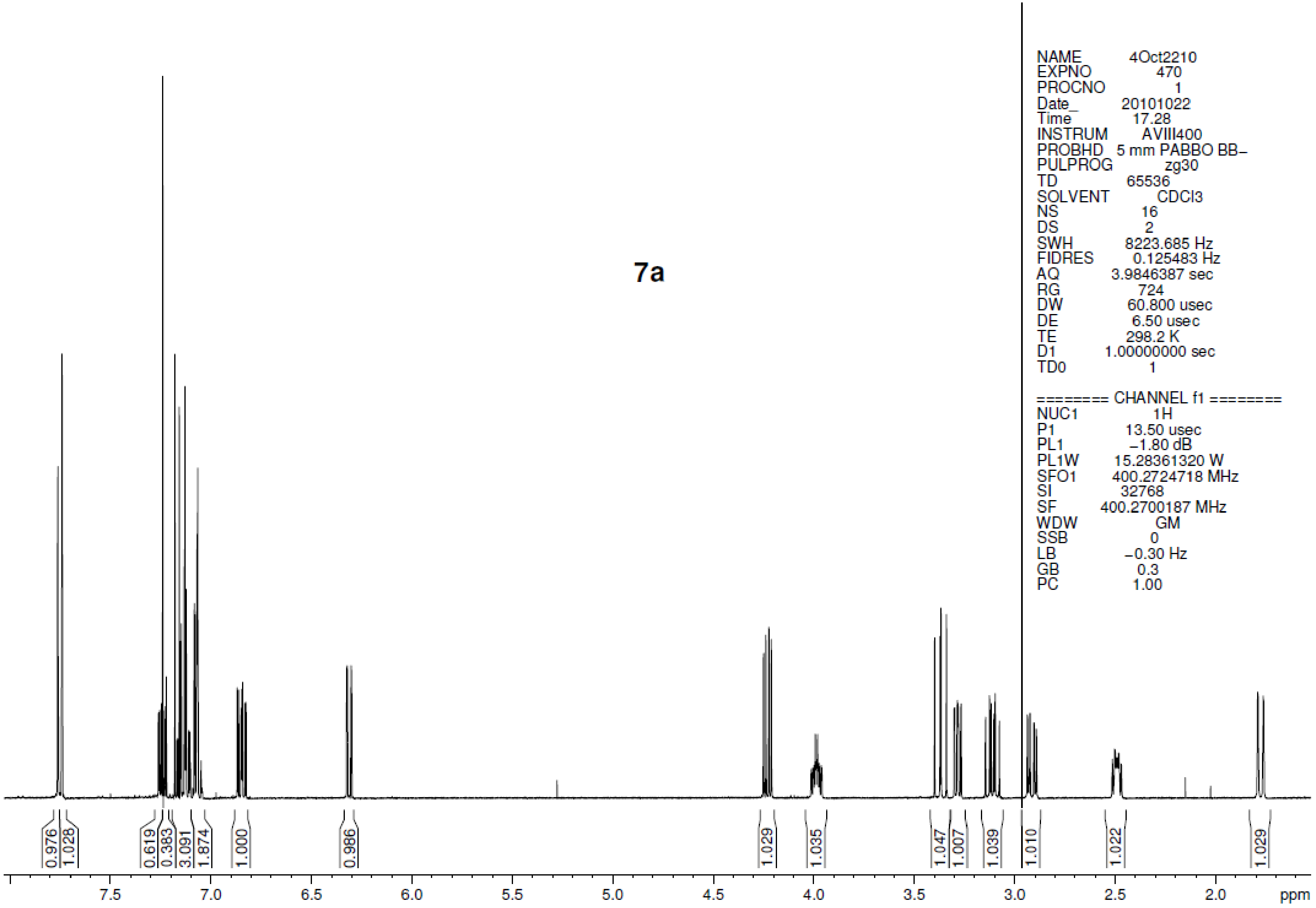
7a
NAME 4Oct2210
EXPNO 470
PROCNO 1
Date_ 20101022
Time 17.28
INSTRUM AVIII400
PROBHD 5 mm PABBO BB-
PULPROG zg30
TD 65536
SOLVENT CDCl3
NS 16
DS 2
SWH 8223.685 Hz
FIDRES 0.125483 Hz
AQ 3.9846387 sec
RG 724
DW 60.800 usec
DE 6.50 usec
TE 298.2 K
D1 1.00000000 sec
TD0 1
======== CHANNEL f1 ========
NUC1 1H
P1 13.50 usec
PL1 -1.80 dB
PL1W 15.28361320 W
SFO1 400.2724718 MHz
SI 32768
SF 400.2700187 MHz
WDW GM
SSB 0
LB -0.30 Hz
GB 0.3
PC 1.00
0.976
1.028
0.619
0.383
3.091
1.874
1.000
0.986
1.029
1.035
1.047
1.007
1.039
1.010
1.022
1.029
7.5
7.0
6.5
6.0
5.5
5.0
4.5
4.0
3.5
3.0
2.5
2.0
ppm

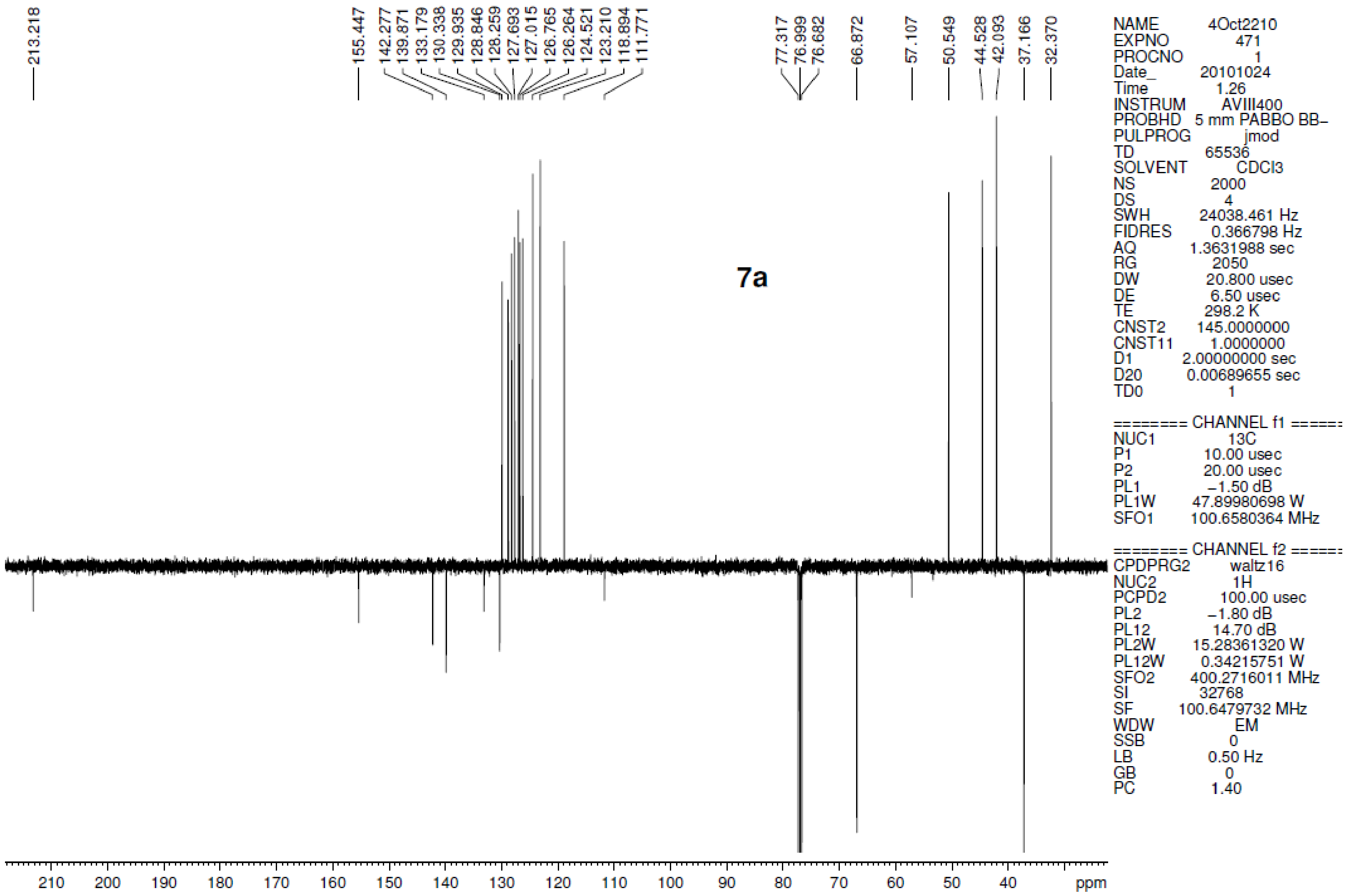
213.218
155.447
142.277
139.871
133.179
130.338
129.935
128.846
128.259
127.693
127.015
126.765
126.264
124.521
123.210
118.894
111.771
77.317
76.999
76.682
66.872
57.107
50.549
44.528
42.093
37.166
32.370
7a
210 200 190 180 170 160 150 140 130 120 110 100 90 80 70 60 50 40 ppm
NAME 4Oct2210
EXPNO 471
PROCNO 1
Date_ 20101024
Time 1.26
INSTRUM AVIII400
PROBHD 5 mm PABBO BB-
PULPROG jmod
TD 65536
SOLVENT CDCl3
NS 2000
DS 4
SWH 24038.461 Hz
FIDRES 0.366798 Hz
AQ 1.3631988 sec
RG 2050
DW 20.800 usec
DE 6.50 usec
TE 298.2 K
CNST2 145.0000000
CNST11 1.0000000
D1 2.00000000 sec
D20 0.00689655 sec
TD0 1
======== CHANNEL f1 ========
NUC1 13C
P1 10.00 usec
P2 20.00 usec
PL1 -1.50 dB
PL1W 47.89980698 W
SFO1 100.6580364 MHz
======== CHANNEL f2 ========
CPDPRG2 waltz16
NUC2 1H
PCPD2 100.00 usec
PL2 -1.80 dB
PL12 14.70 dB
PL2W 15.28361320 W
PL12W 0.34215751 W
SFO2 400.2716011 MHz
SI 32768
SF 100.6479732 MHz
WDW EM
SSB 0
LB 0.50 Hz
GB 0
PC 1.40

7b

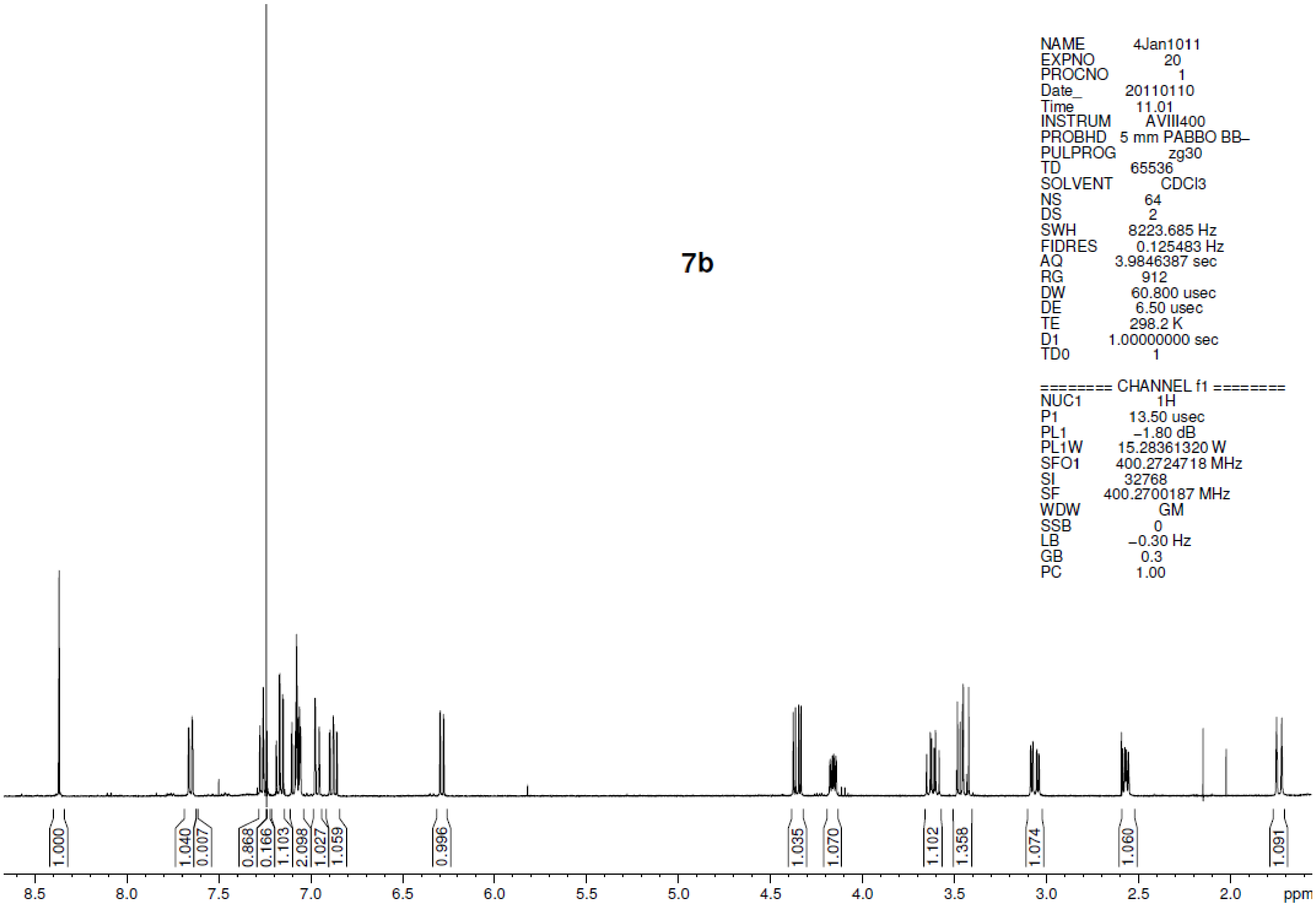

NAME 4Jan1011
EXPNO 20
PROCNO 1
Date_ 20110110
Time 11.01
INSTRUM AVIII400
PROBHD 5 mm PABBO BB–
PULPROG zg30
TD 65536
SOLVENT CDCl3
NS 64
DS 2
SWH 8223.685 Hz
FIDRES 0.125483 Hz
AQ 3.9846387 sec
RG 912
DW 60.800 usec
DE 6.50 usec
TE 298.2 K
D1 1.00000000 sec
TD0 1

======== CHANNEL f1 ========
NUC1 1H
P1 13.50 usec
PL1 –1.80 dB
PL1W 15.28361320 W
SFO1 400.2724718 MHz
SI 32768
SF 400.2700187 MHz
WDW GM
SSB 0
LB –0.30 Hz
GB 0.3
PC 1.00

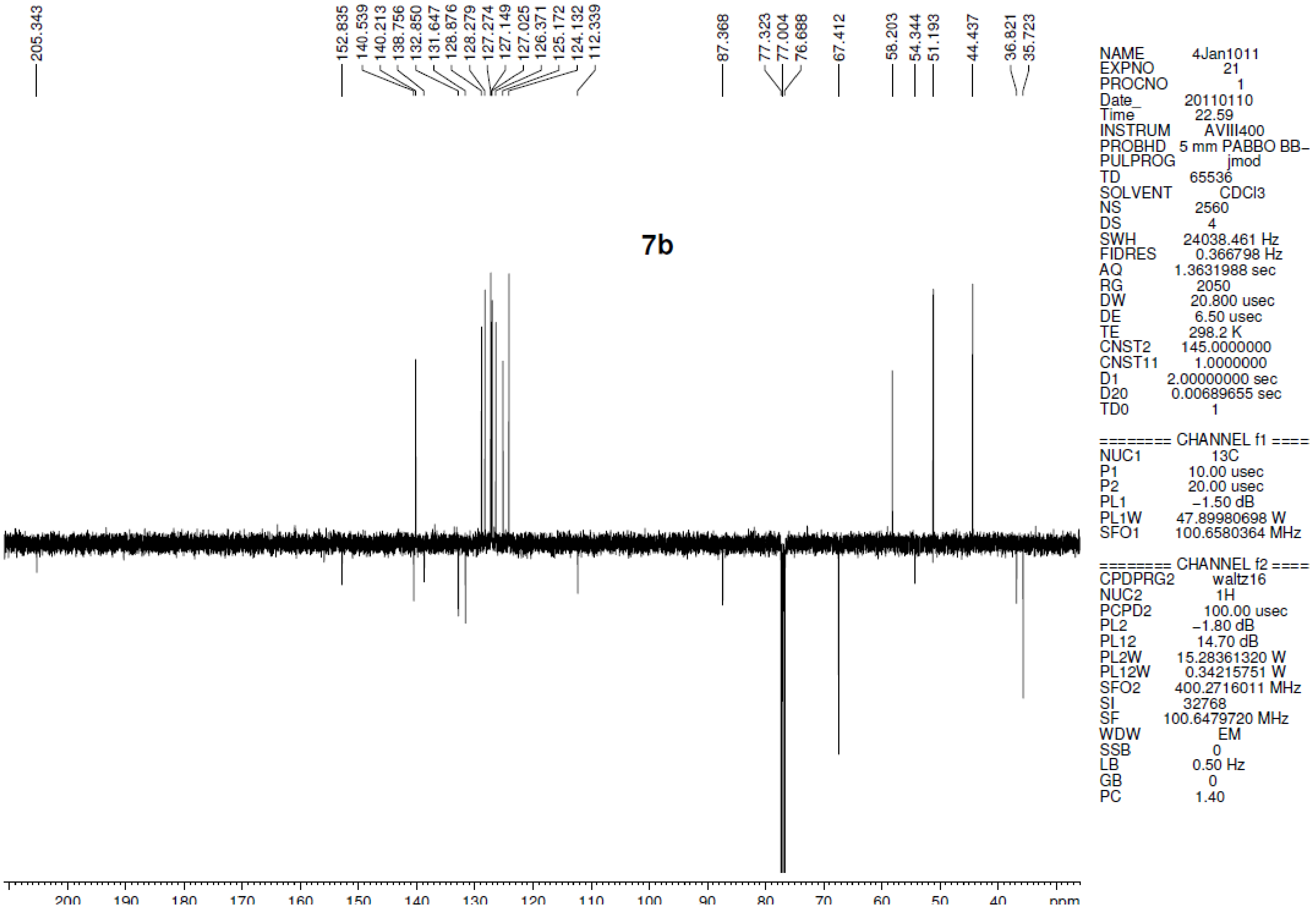
205.343
152.835
140.539
140.213
138.756
132.850
131.647
128.876
128.279
127.274
127.149
127.025
126.371
125.172
124.132
112.339
87.368
77.323
77.004
76.688
67.412
58.203
54.344
51.193
44.437
36.821
35.723
7b
200 190 180 170 160 150 140 130 120 110 100 90 80 70 60 50 40 ppm
NAME 4Jan1011
EXPNO 21
PROCNO 1
Date_ 20110110
Time 22.59
INSTRUM AVIII400
PROBHD 5 mm PABBO BB–
PULPROG jmod
TD 65536
SOLVENT CDCl3
NS 2560
DS 4
SWH 24038.461 Hz
FIDRES 0.366798 Hz
AQ 1.3631988 sec
RG 2050
DW 20.800 usec
DE 6.50 usec
TE 298.2 K
CNST2 145.0000000
CNST11 1.0000000
D1 2.00000000 sec
D20 0.00689655 sec
TD0 1
======== CHANNEL f1 ====
NUC1 13C
P1 10.00 usec
P2 20.00 usec
PL1 –1.50 dB
PL1W 47.89980698 W
SFO1 100.6580364 MHz
======== CHANNEL f2 ====
CPDPRG2 waltz16
NUC2 1H
PCPD2 100.00 usec
PL2 –1.80 dB
PL12 14.70 dB
PL2W 15.28361320 W
PL12W 0.34215751 W
SFO2 400.2716011 MHz
SI 32768
SF 100.6479720 MHz
WDW EM
SSB 0
LB 0.50 Hz
GB 0
PC 1.40

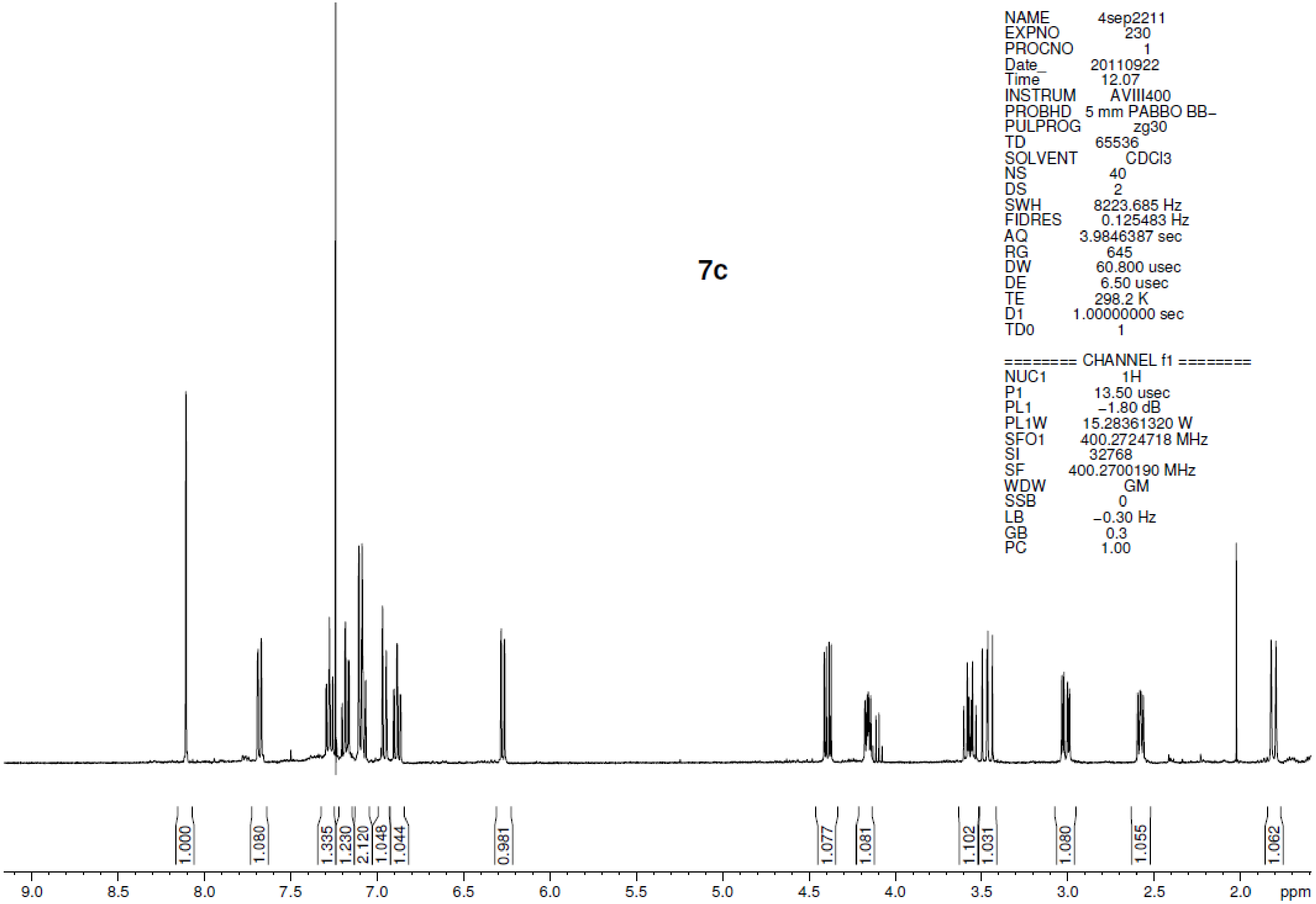
7c
NAME 4sep2211
EXPNO 230
PROCNO 1
Date_ 20110922
Time 12.07
INSTRUM AVIII400
PROBHD 5 mm PABBO BB-
PULPROG zg30
TD 65536
SOLVENT CDCl3
NS 40
DS 2
SWH 8223.685 Hz
FIDRES 0.125483 Hz
AQ 3.9846387 sec
RG 645
DW 60.800 usec
DE 6.50 usec
TE 298.2 K
D1 1.00000000 sec
TD0 1
======== CHANNEL f1 ========
NUC1 1H
P1 13.50 usec
PL1 -1.80 dB
PL1W 15.28361320 W
SFO1 400.2724718 MHz
SI 32768
SF 400.2700190 MHz
WDW GM
SSB 0
LB -0.30 Hz
GB 0.3
PC 1.00
1.000
1.080
1.335
1.230
2.120
1.048
1.044
0.981
1.077
1.081
1.102
1.031
1.080
1.055
1.062
9.0
8.5
8.0
7.5
7.0
6.5
6.0
5.5
5.0
4.5
4.0
3.5
3.0
2.5
2.0
ppm

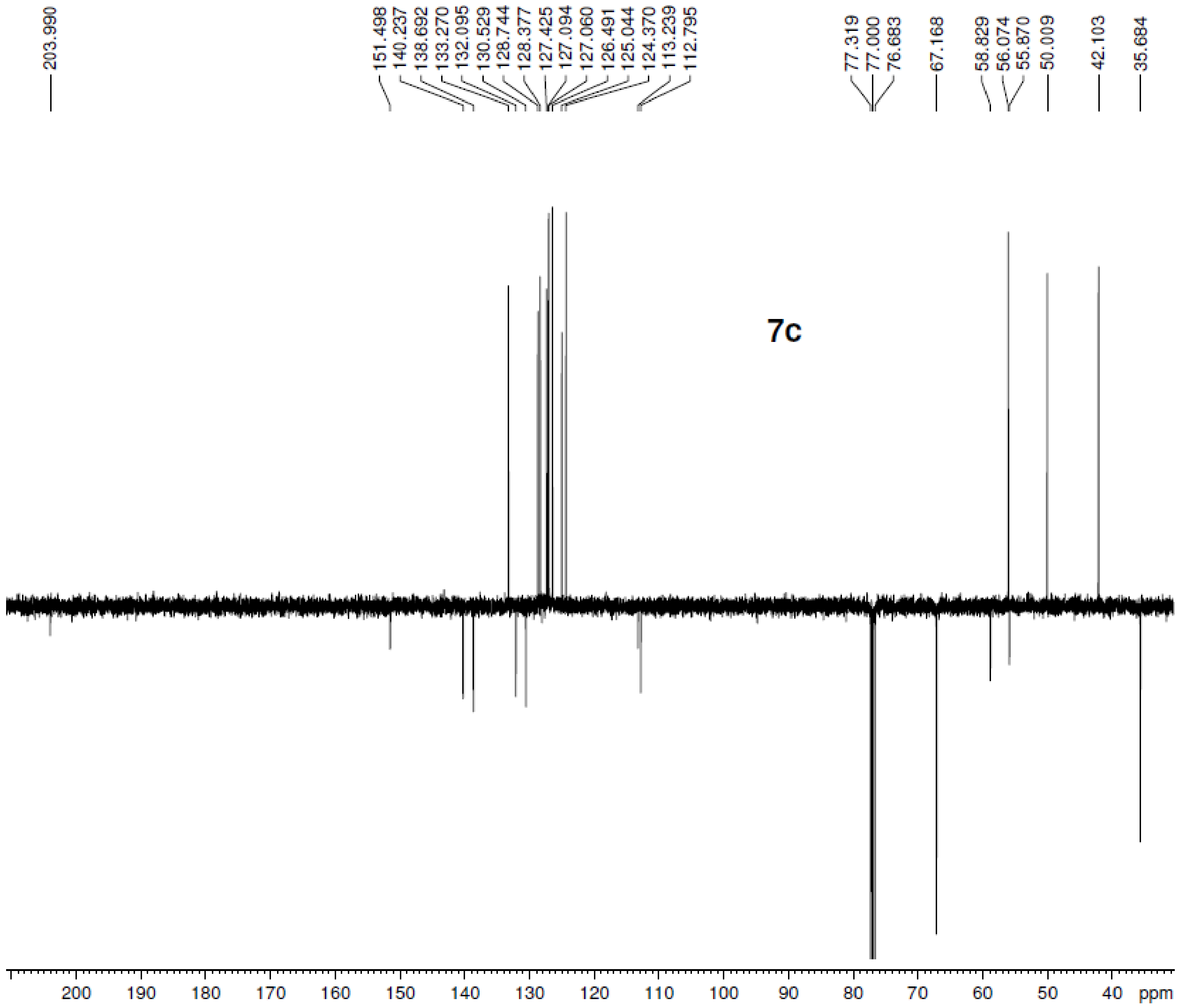

NAME 4sep2211
EXPNO 231
PROCNO 1
Date_ 20110922
Time 23.00
INSTRUM AVIII400
PROBHD 5 mm PABBO BB–
PULPROG jmod
TD 65536
SOLVENT CDCl3
NS 2000
DS 4
SWH 24038.461 Hz
FIDRES 0.366798 Hz
AQ 1.3631988 sec
RG 2050
DW 20.800 usec
DE 6.50 usec
TE 298.2 K
CNST2 145.0000000
CNST11 1.0000000
D1 2.00000000 sec
D20 0.00689655 sec
TD0 1

======== CHANNEL f1 ========
NUC1 13C
P1 10.00 usec
P2 20.00 usec
PL1 –1.50 dB
PL1W 47.89980698 W
SFO1 100.6580364 MHz

======== CHANNEL f2 ========
CPDPRG2 waltz16
NUC2 1H
PCPD2 100.00 usec
PL2 –1.80 dB
PL12 14.70 dB
PL2W 15.28361320 W
PL12W 0.34215751 W
SFO2 400.2716011 MHz
SI 32768
SF 100.6479731 MHz
WDW EM
SSB 0
LB 0.50 Hz
GB 0
PC 1.40

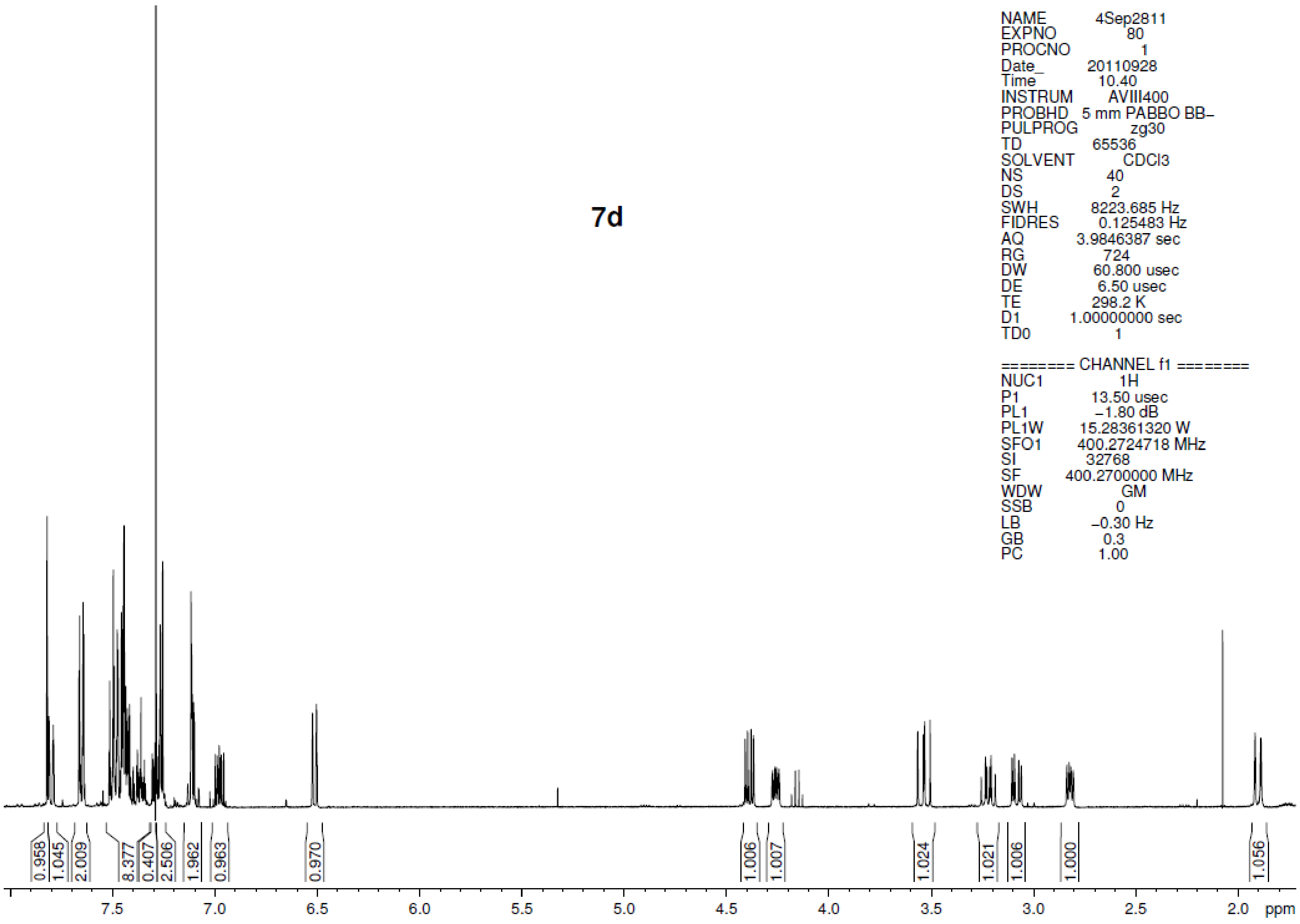
7d
NAME 4Sep2811
EXPNO 80
PROCNO 1
Date_ 20110928
Time 10.40
INSTRUM AVIII400
PROBHD 5 mm PABBO BB-
PULPROG zg30
TD 65536
SOLVENT CDCl3
NS 40
DS 2
SWH 8223.685 Hz
FIDRES 0.125483 Hz
AQ 3.9846387 sec
RG 724
DW 60.800 usec
DE 6.50 usec
TE 298.2 K
D1 1.00000000 sec
TD0 1
======== CHANNEL f1 ========
NUC1 1H
P1 13.50 usec
PL1 -1.80 dB
PL1W 15.28361320 W
SFO1 400.2724718 MHz
SI 32768
SF 400.2700000 MHz
WDW GM
SSB 0
LB -0.30 Hz
GB 0.3
PC 1.00
0.958
1.045
2.009
8.377
0.407
2.506
1.962
0.963
0.970
1.006
1.007
1.024
1.021
1.006
1.000
1.056
7.5
7.0
6.5
6.0
5.5
5.0
4.5
4.0
3.5
3.0
2.5
2.0
ppm

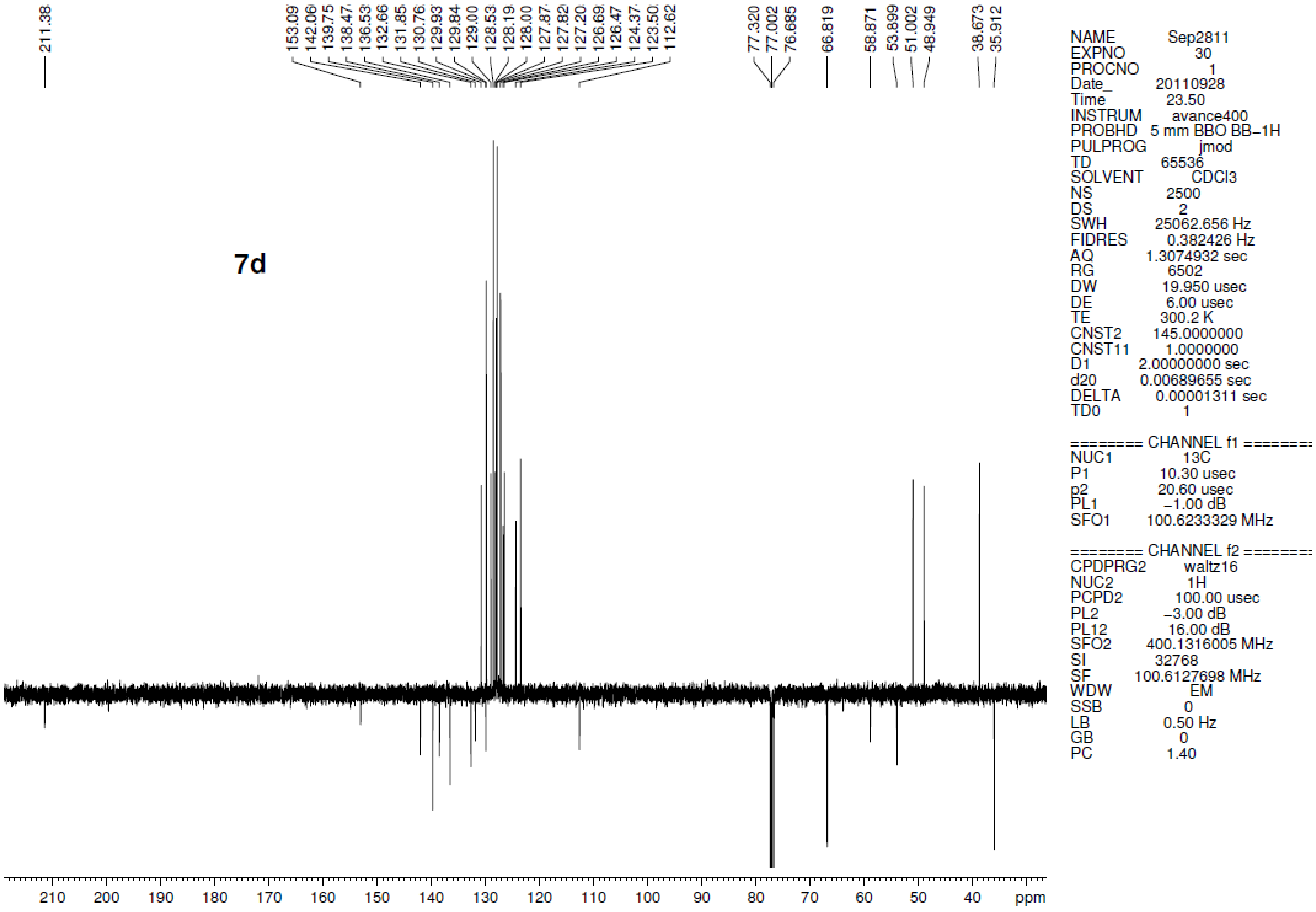
7d
211.38
153.09
142.06
139.75
138.47
136.53
132.66
131.85
130.76
129.93
129.84
129.00
128.53
128.19
128.00
127.87
127.82
127.20
126.69
126.47
124.37
123.50
112.62
77.320
77.002
76.685
66.819
58.871
53.899
51.002
48.949
38.673
35.912
210 200 190 180 170 160 150 140 130 120 110 100 90 80 70 60 50 40 ppm
NAME Sep2811
EXPNO 30
PROCNO 1
Date_ 20110928
Time 23.50
INSTRUM avance400
PROBHD 5 mm BBO BB-1H
PULPROG jmod
TD 65536
SOLVENT CDCl3
NS 2500
DS 2
SWH 25062.656 Hz
FIDRES 0.382426 Hz
AQ 1.3074932 sec
RG 6502
DW 19.950 usec
DE 6.00 usec
TE 300.2 K
CNST2 145.0000000
CNST11 1.0000000
D1 2.00000000 sec
d20 0.00689655 sec
DELTA 0.00001311 sec
TD0 1
======== CHANNEL f1 ========
NUC1 13C
P1 10.30 usec
p2 20.60 usec
PL1 -1.00 dB
SFO1 100.6233329 MHz
======== CHANNEL f2 ========
CPDPRG2 waltz16
NUC2 1H
PCPD2 100.00 usec
PL2 -3.00 dB
PL12 16.00 dB
SFO2 400.1316005 MHz
SI 32768
SF 100.6127698 MHz
WDW EM
SSB 0
LB 0.50 Hz
GB 0
PC 1.40

(R)–8

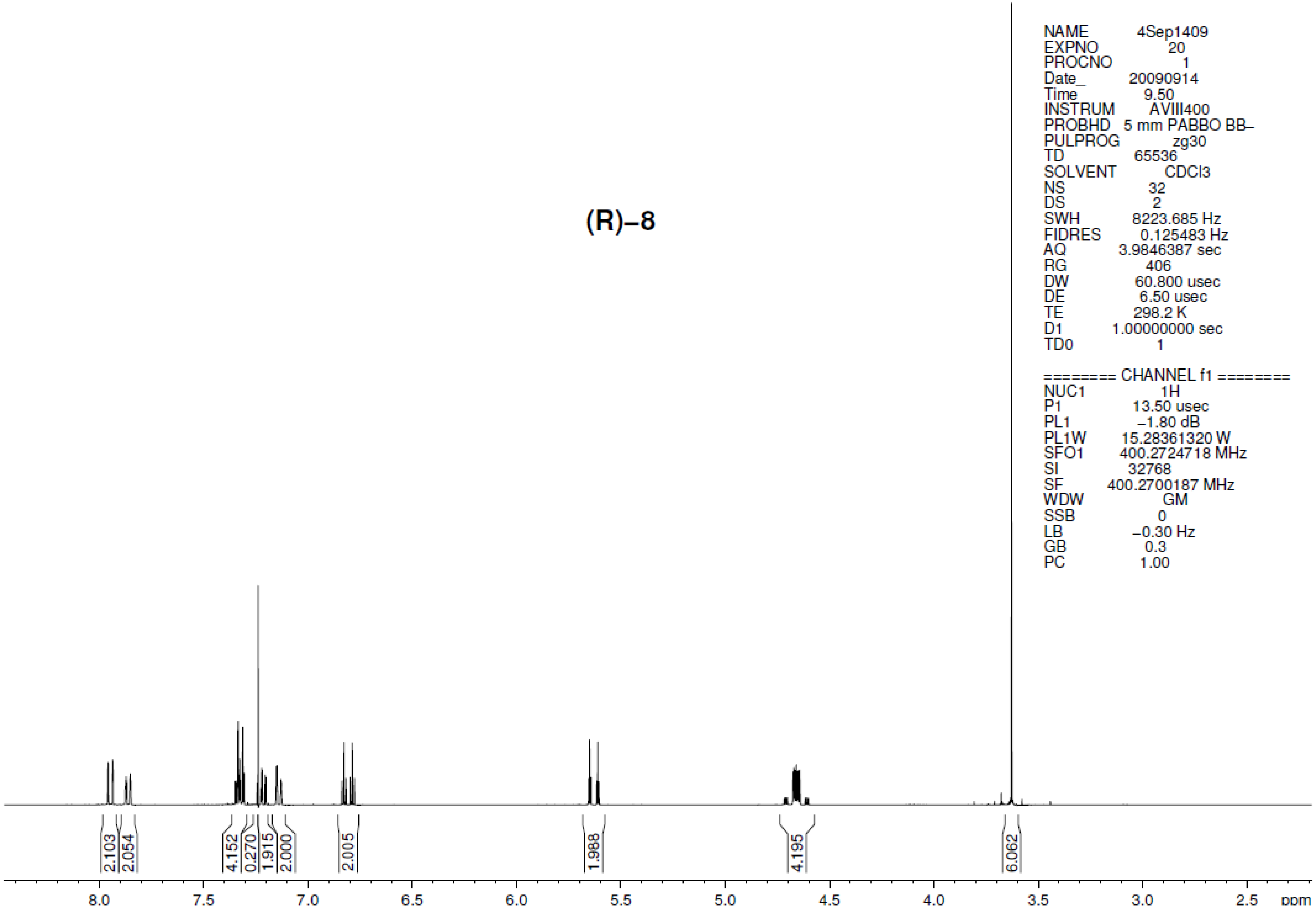
NAME 4Sep1409
EXPNO 20
PROCNO 1
Date_ 20090914
Time 9.50
INSTRUM AVIII400
PROBHD 5 mm PABBO BB–
PULPROG zg30
TD 65536
SOLVENT CDCl3
NS 32
DS 2
SWH 8223.685 Hz
FIDRES 0.125483 Hz
AQ 3.9846387 sec
RG 406
DW 60.800 usec
DE 6.50 usec
TE 298.2 K
D1 1.00000000 sec
TD0 1
======== CHANNEL f1 ========
NUC1 1H
P1 13.50 usec
PL1 −1.80 dB
PL1W 15.28361320 W
SFO1 400.2724718 MHz
SI 32768
SF 400.2700187 MHz
WDW GM
SSB 0
LB −0.30 Hz
GB 0.3
PC 1.00
2.103
2.054
4.152
0.270
1.915
2.000
2.005
1.988
4.195
6.062
8.0
7.5
7.0
6.5
6.0
5.5
5.0
4.5
4.0
3.5
3.0
2.5
ppm

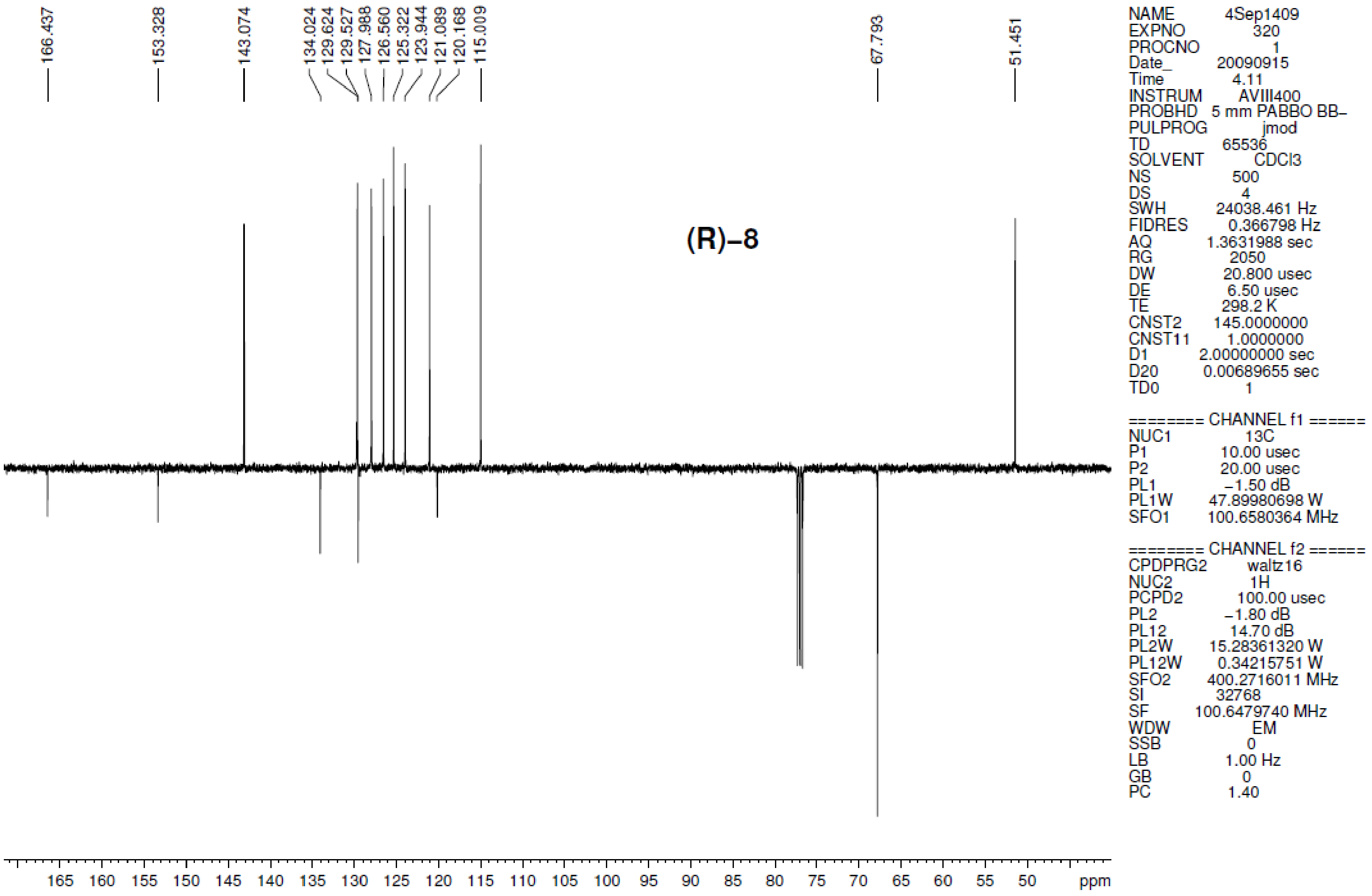
166.437
153.328
143.074
134.024
129.624
129.527
127.988
126.560
125.322
123.944
121.089
120.168
115.009
67.793
51.451
(R)–8
NAME 4Sep1409
EXPNO 320
PROCNO 1
Date_ 20090915
Time 4.11
INSTRUM AVIII400
PROBHD 5 mm PABBO BB–
PULPROG jmod
TD 65536
SOLVENT CDCl3
NS 500
DS 4
SWH 24038.461 Hz
FIDRES 0.366798 Hz
AQ 1.3631988 sec
RG 2050
DW 20.800 usec
DE 6.50 usec
TE 298.2 K
CNST2 145.0000000
CNST11 1.0000000
D1 2.00000000 sec
D20 0.00689655 sec
TD0 1
======== CHANNEL f1 ======
NUC1 13C
P1 10.00 usec
P2 20.00 usec
PL1 –1.50 dB
PL1W 47.89980698 W
SFO1 100.6580364 MHz
======== CHANNEL f2 ======
CPDPRG2 waltz16
NUC2 1H
PCPD2 100.00 usec
PL2 –1.80 dB
PL12 14.70 dB
PL2W 15.28361320 W
PL12W 0.34215751 W
SFO2 400.2716011 MHz
SI 32768
SF 100.6479740 MHz
WDW EM
SSB 0
LB 1.00 Hz
GB 0
PC 1.40
165 160 155 150 145 140 135 130 125 120 115 110 105 100 95 90 85 80 75 70 65 60 55 50 ppm

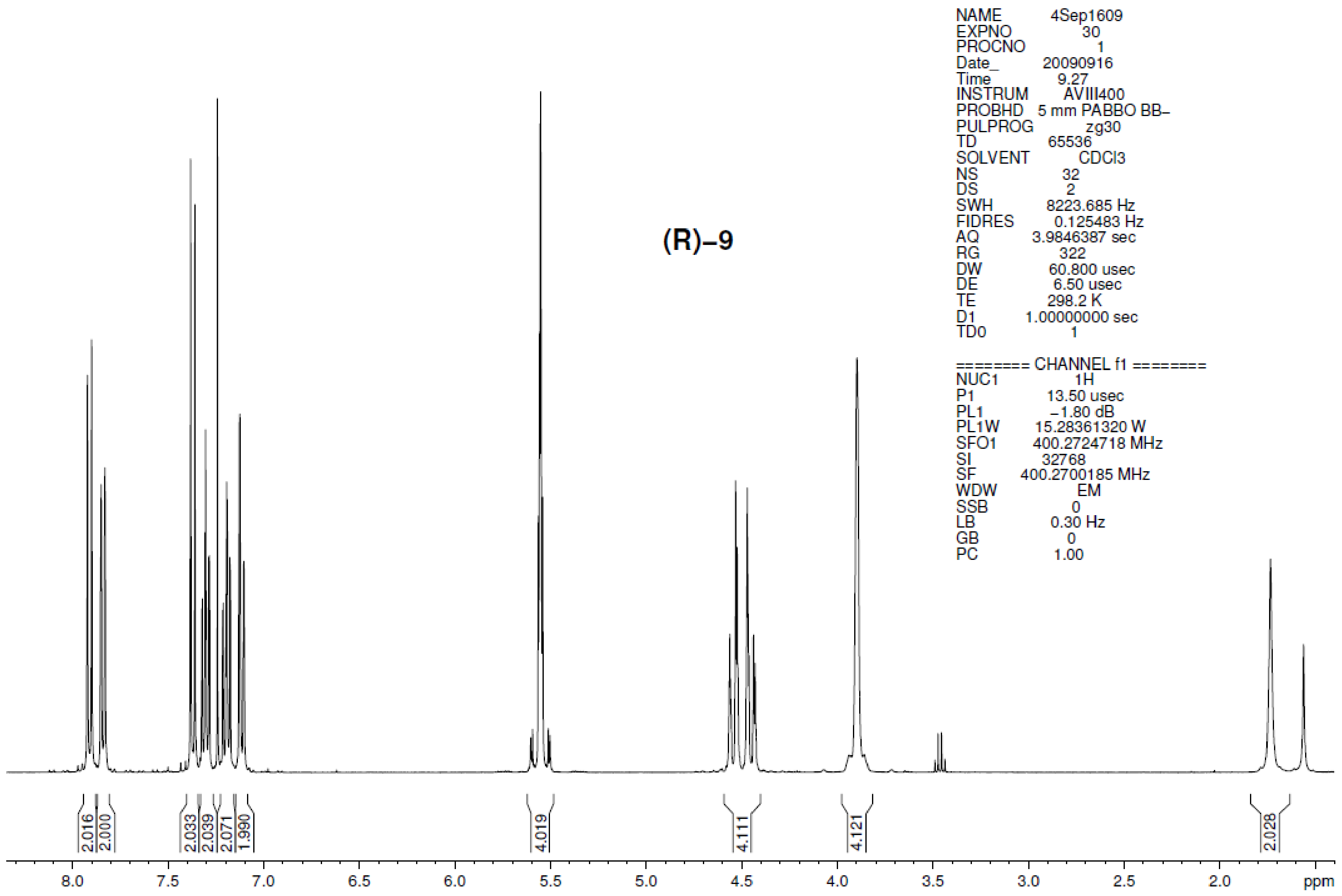

(R)–9
NAME 4Sep1609
EXPNO 30
PROCNO 1
Date_ 20090916
Time 9.27
INSTRUM AVIII400
PROBHD 5 mm PABBO BB–
PULPROG zg30
TD 65536
SOLVENT CDCl3
NS 32
DS 2
SWH 8223.685 Hz
FIDRES 0.125483 Hz
AQ 3.9846387 sec
RG 322
DW 60.800 usec
DE 6.50 usec
TE 298.2 K
D1 1.00000000 sec
TD0 1
======== CHANNEL f1 ========
NUC1 1H
P1 13.50 usec
PL1 –1.80 dB
PL1W 15.28361320 W
SFO1 400.2724718 MHz
SI 32768
SF 400.2700185 MHz
WDW EM
SSB 0
LB 0.30 Hz
GB 0
PC 1.00
2.016
2.000
2.033
2.039
2.071
1.990
4.019
4.111
4.121
2.028
8.0
7.5
7.0
6.5
6.0
5.5
5.0
4.5
4.0
3.5
3.0
2.5
2.0
ppm

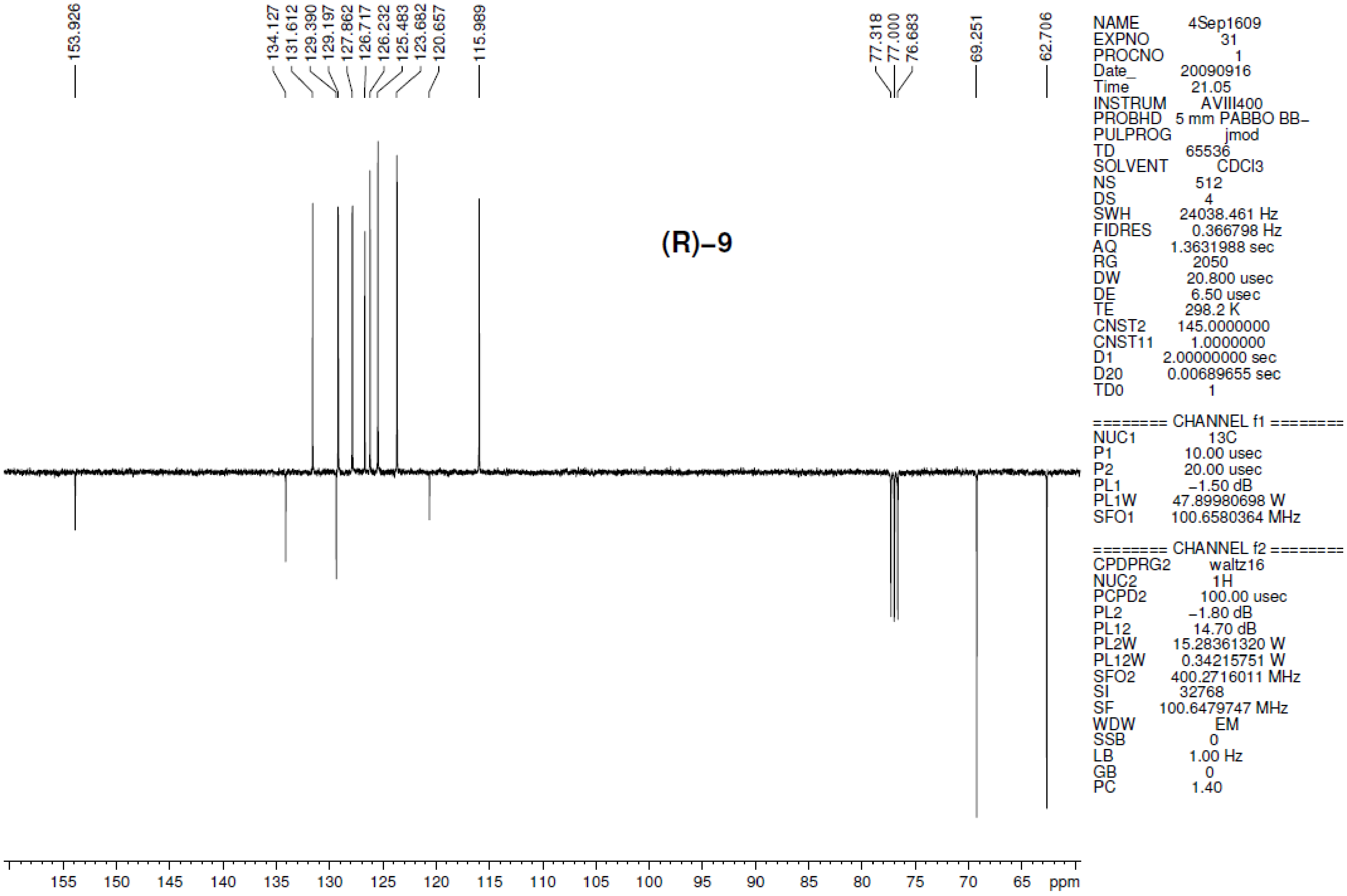
153.926
134.127
131.612
129.390
129.197
127.862
126.717
126.232
125.483
123.682
120.657
115.989
77.318
77.000
76.683
69.251
62.706
(R)–9
NAME 4Sep1609
EXPNO 31
PROCNO 1
Date_ 20090916
Time 21.05
INSTRUM AVIII400
PROBHD 5 mm PABBO BB-
PULPROG jmod
TD 65536
SOLVENT CDCl3
NS 512
DS 4
SWH 24038.461 Hz
FIDRES 0.366798 Hz
AQ 1.3631988 sec
RG 2050
DW 20.800 usec
DE 6.50 usec
TE 298.2 K
CNST2 145.0000000
CNST11 1.0000000
D1 2.00000000 sec
D20 0.00689655 sec
TD0 1
======== CHANNEL f1 ========
NUC1 13C
P1 10.00 usec
P2 20.00 usec
PL1 -1.50 dB
PL1W 47.89980698 W
SFO1 100.6580364 MHz
======== CHANNEL f2 ========
CPDPRG2 waltz16
NUC2 1H
PCPD2 100.00 usec
PL2 -1.80 dB
PL12 14.70 dB
PL2W 15.28361320 W
PL12W 0.34215751 W
SFO2 400.2716011 MHz
SI 32768
SF 100.6479747 MHz
WDW EM
SSB 0
LB 1.00 Hz
GB 0
PC 1.40
155 150 145 140 135 130 125 120 115 110 105 100 95 90 85 80 75 70 65 ppm

(R)–10

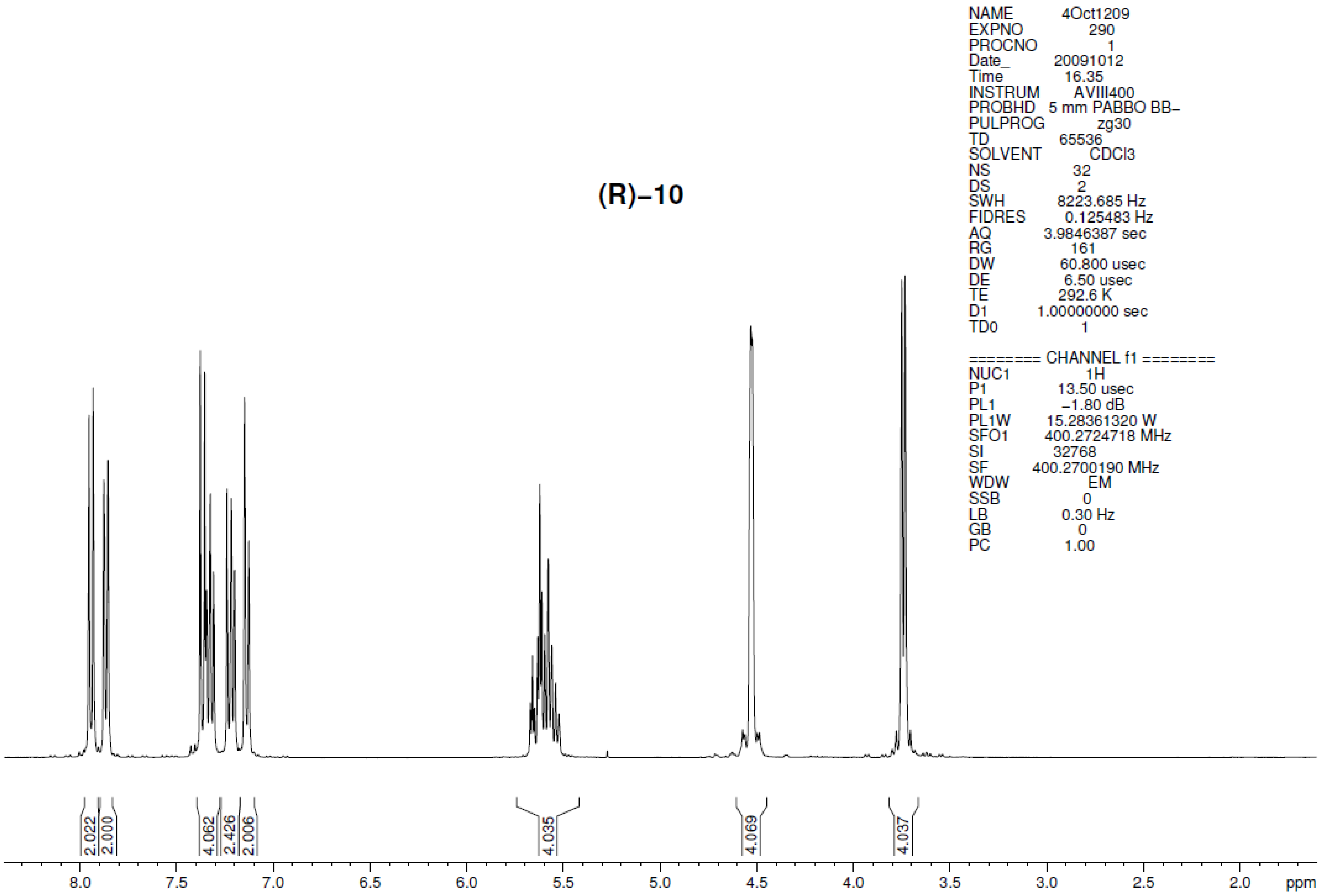
NAME 4Oct1209
EXPNO 290
PROCNO 1
Date_ 20091012
Time 16.35
INSTRUM AVIII400
PROBHD 5 mm PABBO BB–
PULPROG zg30
TD 65536
SOLVENT CDCl3
NS 32
DS 2
SWH 8223.685 Hz
FIDRES 0.125483 Hz
AQ 3.9846387 sec
RG 161
DW 60.800 usec
DE 6.50 usec
TE 292.6 K
D1 1.00000000 sec
TD0 1
======== CHANNEL f1 ========
NUC1 1H
P1 13.50 usec
PL1 –1.80 dB
PL1W 15.28361320 W
SFO1 400.2724718 MHz
SI 32768
SF 400.2700190 MHz
WDW EM
SSB 0
LB 0.30 Hz
GB 0
PC 1.00
2.022
2.000
4.062
2.426
2.006
4.035
4.069
4.037
8.0
7.5
7.0
6.5
6.0
5.5
5.0
4.5
4.0
3.5
3.0
2.5
2.0
ppm

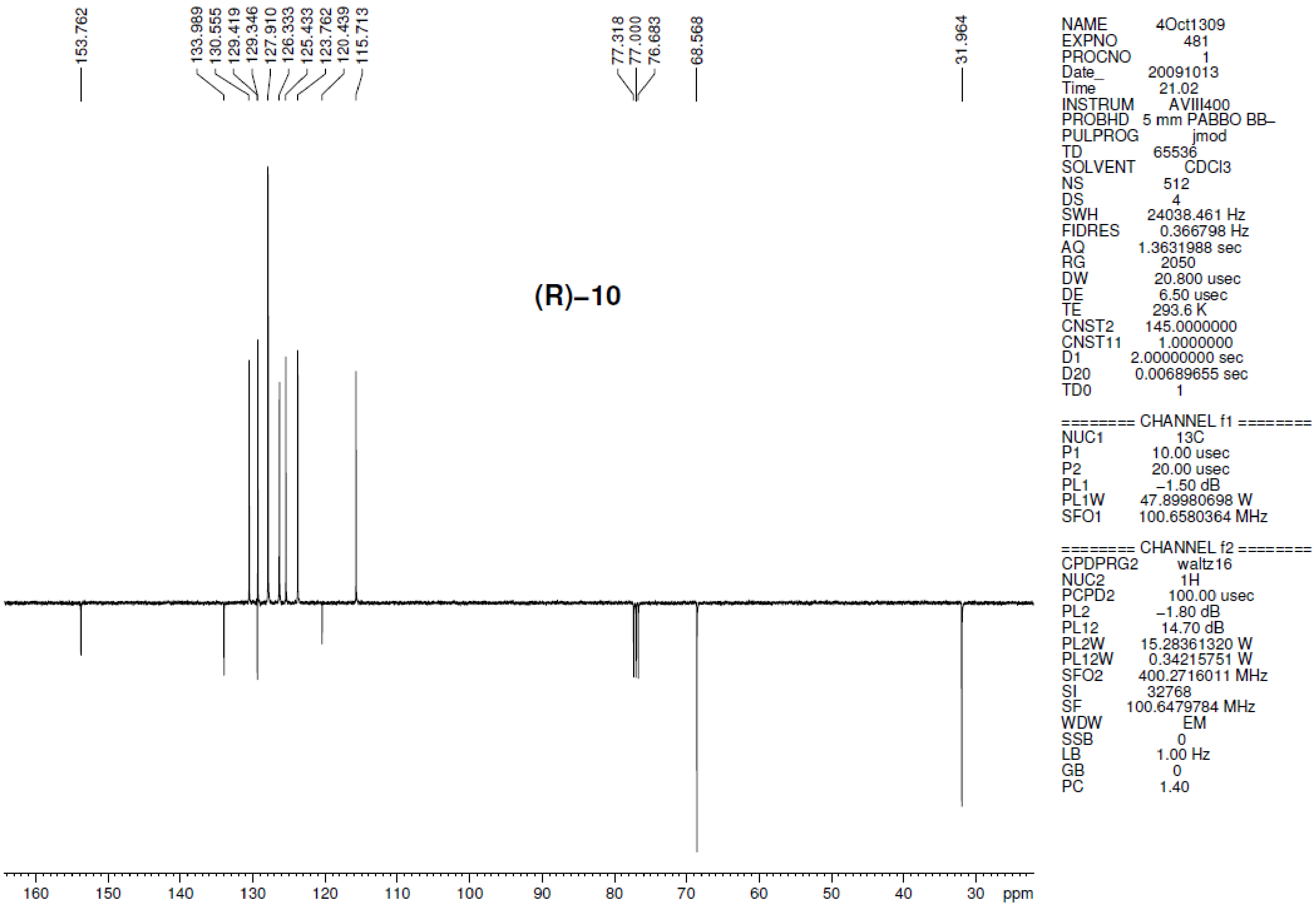
153.762
133.989
130.555
129.419
129.346
127.910
126.333
125.433
123.762
120.439
115.713
77.318
77.000
76.683
68.568
31.964
(R)–10
160 150 140 130 120 110 100 90 80 70 60 50 40 30 ppm
NAME 4Oct1309
EXPNO 481
PROCNO 1
Date_ 20091013
Time 21.02
INSTRUM AVIII400
PROBHD 5 mm PABBO BB–
PULPROG jmod
TD 65536
SOLVENT CDCl3
NS 512
DS 4
SWH 24038.461 Hz
FIDRES 0.366798 Hz
AQ 1.3631988 sec
RG 2050
DW 20.800 usec
DE 6.50 usec
TE 293.6 K
CNST2 145.0000000
CNST11 1.0000000
D1 2.00000000 sec
D20 0.00689655 sec
TD0 1
======== CHANNEL f1 ========
NUC1 13C
P1 10.00 usec
P2 20.00 usec
PL1 –1.50 dB
PL1W 47.89980698 W
SFO1 100.6580364 MHz
======== CHANNEL f2 ========
CPDPRG2 waltz16
NUC2 1H
PCPD2 100.00 usec
PL2 –1.80 dB
PL12 14.70 dB
PL2W 15.28361320 W
PL12W 0.34215751 W
SFO2 400.2716011 MHz
SI 32768
SF 100.6479784 MHz
WDW EM
SSB 0
LB 1.00 Hz
GB 0
PC 1.40

11

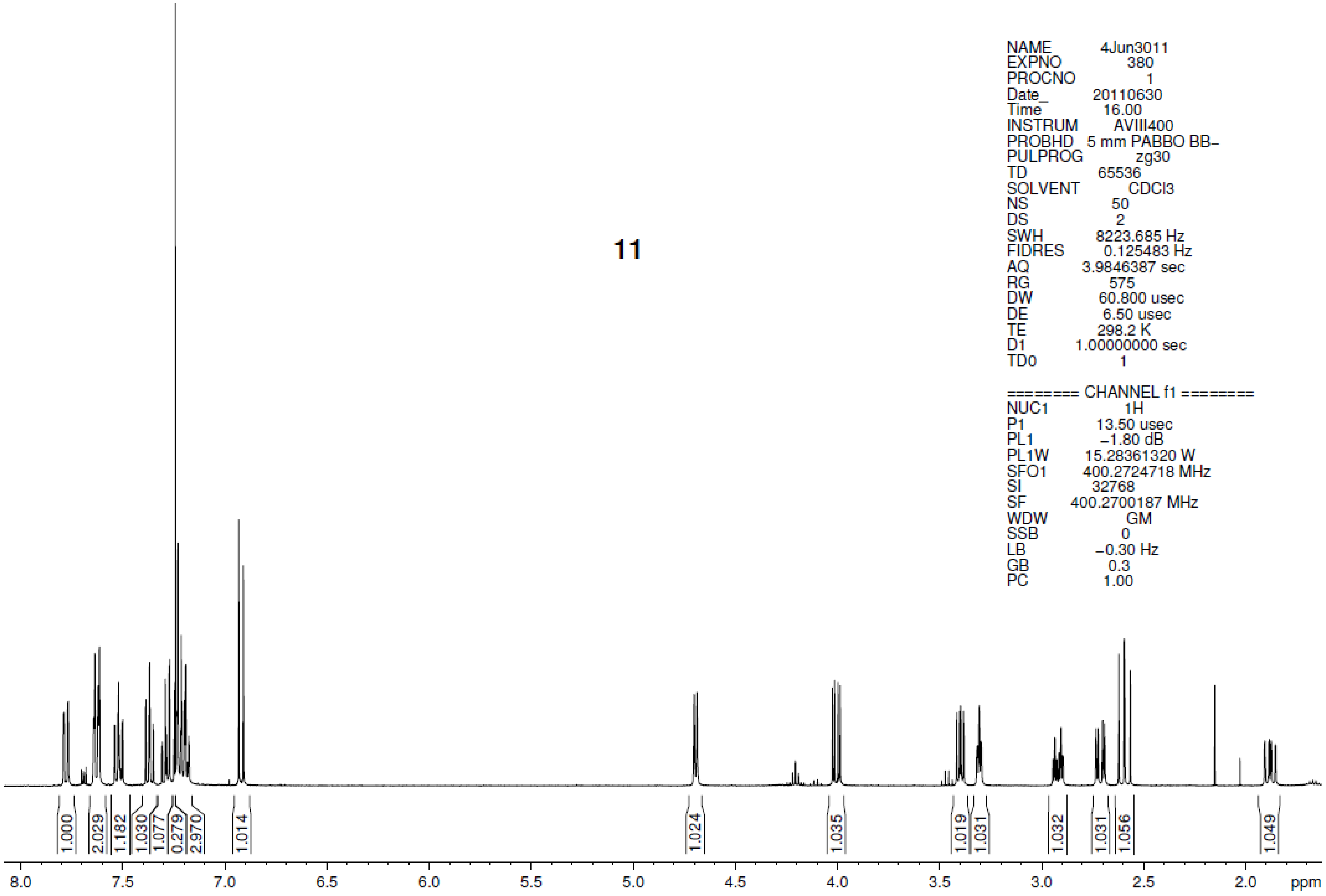
NAME 4Jun3011
EXPNO 380
PROCNO 1
Date_ 20110630
Time 16.00
INSTRUM AVIII400
PROBHD 5 mm PABBO BB-
PULPROG zg30
TD 65536
SOLVENT CDCl3
NS 50
DS 2
SWH 8223.685 Hz
FIDRES 0.125483 Hz
AQ 3.9846387 sec
RG 575
DW 60.800 usec
DE 6.50 usec
TE 298.2 K
D1 1.00000000 sec
TD0 1
======== CHANNEL f1 ========
NUC1 1H
P1 13.50 usec
PL1 -1.80 dB
PL1W 15.28361320 W
SFO1 400.2724718 MHz
SI 32768
SF 400.2700187 MHz
WDW GM
SSB 0
LB -0.30 Hz
GB 0.3
PC 1.00
1.000
2.029
1.182
1.030
1.077
0.279
2.970
1.014
1.024
1.035
1.019
1.031
1.032
1.031
1.056
1.049
8.0
7.5
7.0
6.5
6.0
5.5
5.0
4.5
4.0
3.5
3.0
2.5
2.0
ppm

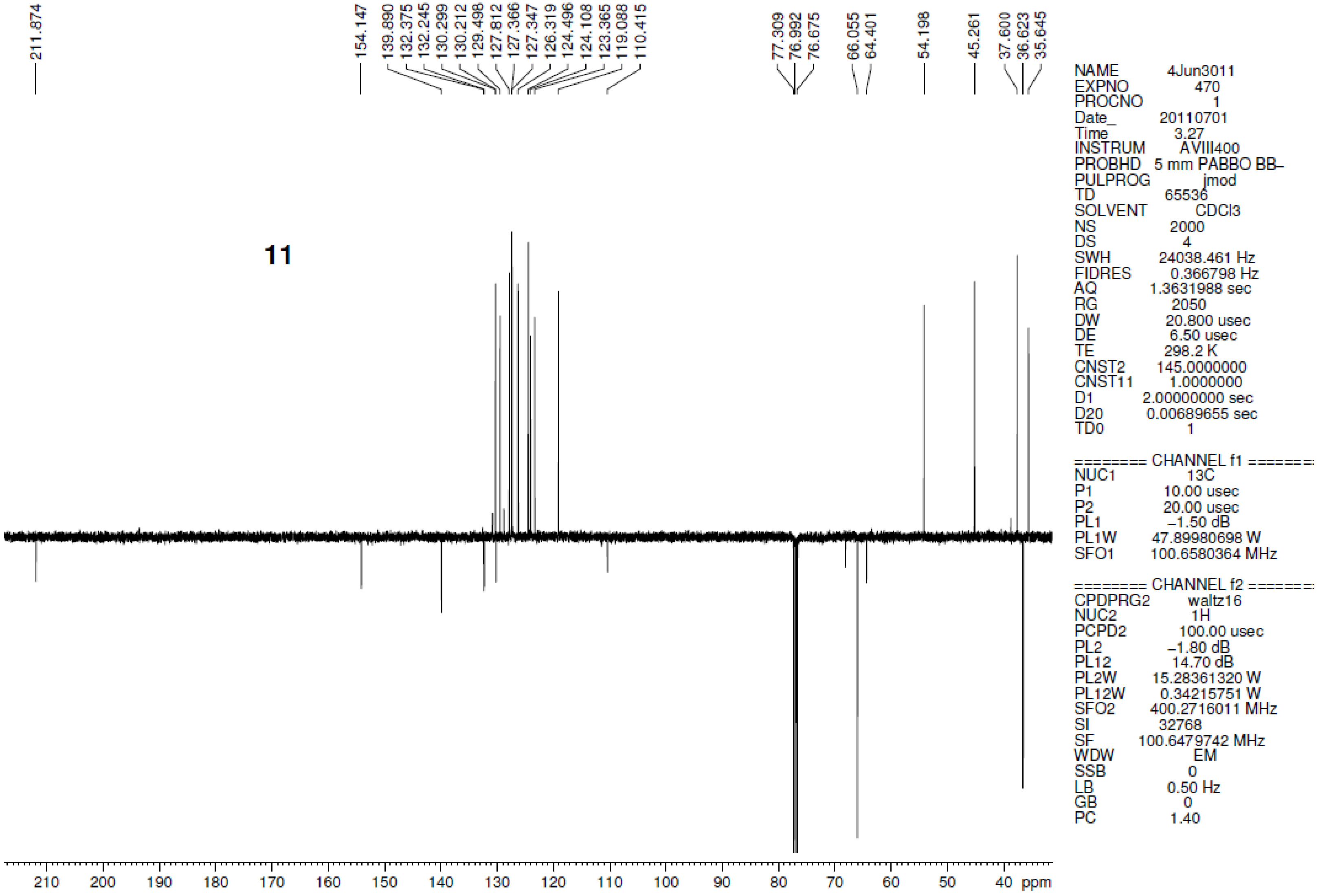
211.874
154.147
139.890
132.375
132.245
130.299
130.212
129.498
127.812
127.366
127.347
126.319
124.496
124.108
123.365
119.088
110.415
77.309
76.992
76.675
66.055
64.401
54.198
45.261
37.600
36.623
35.645
11
210 200 190 180 170 160 150 140 130 120 110 100 90 80 70 60 50 40 ppm
NAME 4Jun3011
EXPNO 470
PROCNO 1
Date_ 20110701
Time 3.27
INSTRUM AVIII400
PROBHD 5 mm PABBO BB-
PULPROG jmod
TD 65536
SOLVENT CDCl3
NS 2000
DS 4
SWH 24038.461 Hz
FIDRES 0.366798 Hz
AQ 1.3631988 sec
RG 2050
DW 20.800 usec
DE 6.50 usec
TE 298.2 K
CNST2 145.0000000
CNST11 1.0000000
D1 2.00000000 sec
D20 0.00689655 sec
TD0 1
======== CHANNEL f1 ========
NUC1 13C
P1 10.00 usec
P2 20.00 usec
PL1 -1.50 dB
PL1W 47.89980698 W
SFO1 100.6580364 MHz
======== CHANNEL f2 ========
CPDPRG2 waltz16
NUC2 1H
PCPD2 100.00 usec
PL2 -1.80 dB
PL12 14.70 dB
PL2W 15.28361320 W
PL12W 0.34215751 W
SFO2 400.2716011 MHz
SI 32768
SF 100.6479742 MHz
WDW EM
SSB 0
LB 0.50 Hz
GB 0
PC 1.40

Formation of the spiro compound **5a**

via SN' reaction or Claisen rearrangement path
(ground state and transition state energies in kcal)

Numerical values from calculations
B3LYP (first line, green)
MO6 (second line, blue)
MP2 (third line, brown)

vacuum

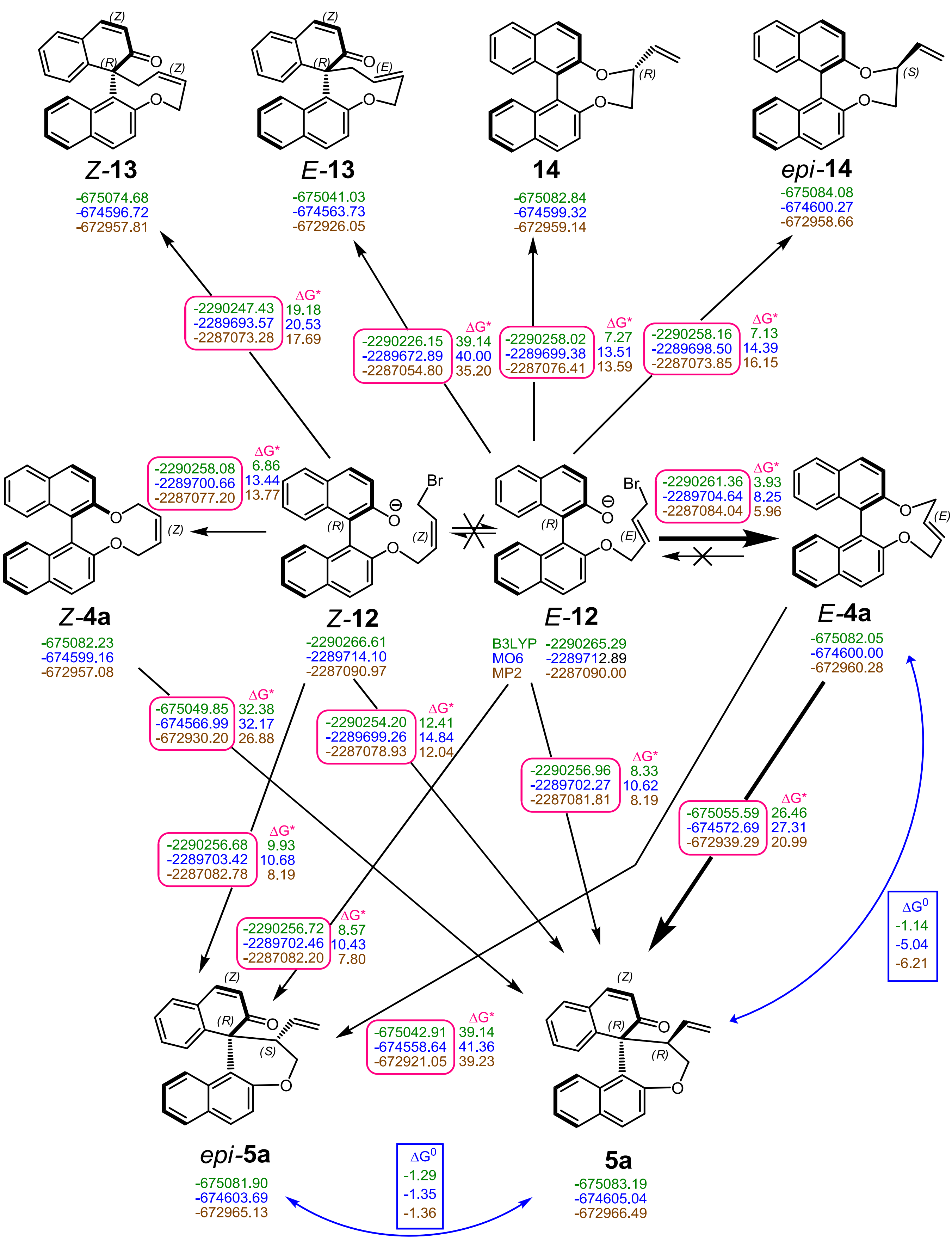

Formation of the spiro compound **5a**
via S_N' reaction or Claisen rearrangement path
(ground state and transition state energies in kcal with B3LYP)

in DCM

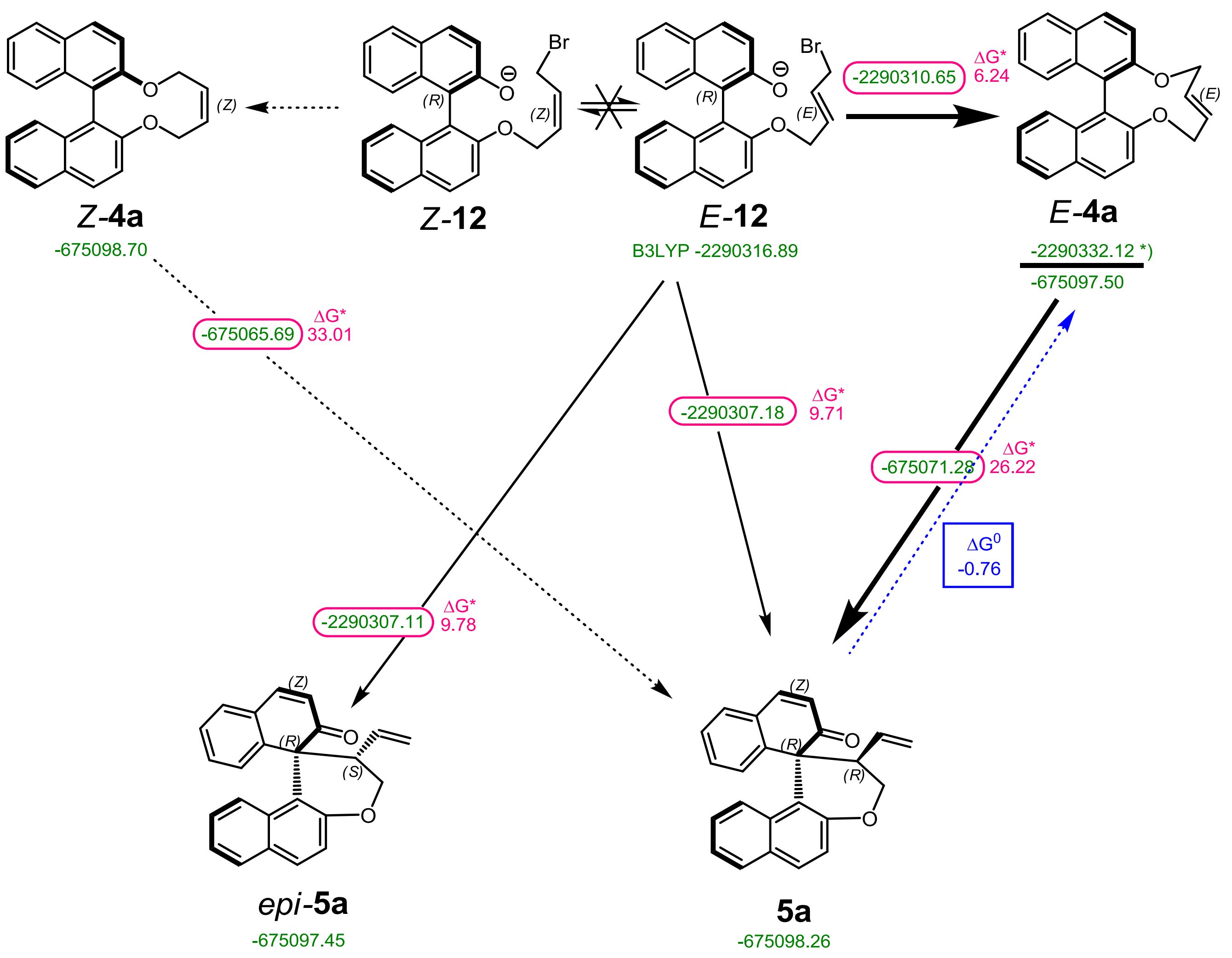

*) bromide included

Atomic coordinates and energies

2a-*pro*-*cis*-**Ru***

Sum of electronic and zero-point Energies= -2668.207250

Sum of electronic and thermal Energies= -2668.165652

Sum of electronic and thermal Enthalpies= -2668.164707

Sum of electronic and thermal Free Energies= -2668.281489

6	4.913491000	0.185625000	-0.522900000
6	6.095333000	0.513460000	-1.205844000
6	3.825928000	1.076217000	-0.626162000
6	6.194718000	1.663042000	-1.988773000
6	3.917147000	2.225611000	-1.417651000
6	5.097147000	2.516614000	-2.101242000
1	6.937353000	-0.169582000	-1.139223000
1	7.118207000	1.882035000	-2.516360000
1	3.067788000	2.899261000	-1.472612000
1	5.158595000	3.412770000	-2.712296000
6	4.853305000	-1.060223000	0.289517000
6	3.853158000	-2.041852000	0.089720000
6	5.848300000	-1.321037000	1.238723000
6	3.871362000	-3.229178000	0.829417000
6	5.869961000	-2.502437000	1.982618000
6	4.877154000	-3.454277000	1.773291000
1	6.612622000	-0.566421000	1.401052000
1	3.113613000	-3.988553000	0.677456000
1	6.649662000	-2.669222000	2.719234000
1	4.872444000	-4.379766000	2.341884000
8	2.705417000	0.816053000	0.122025000
8	2.927596000	-1.759590000	-0.878635000
6	1.455949000	0.784364000	-0.586594000
1	1.578970000	0.349398000	-1.580513000
1	1.054898000	1.800426000	-0.712613000
6	0.487396000	0.017409000	0.254411000
1	0.778519000	0.005027000	1.314171000
6	1.872079000	-2.664346000	-1.164787000
1	1.452713000	-2.330816000	-2.117459000
1	2.271081000	-3.680669000	-1.306691000
6	0.780349000	-2.722028000	-0.124645000
1	1.064376000	-2.653498000	0.921718000
6	-0.484757000	-3.149513000	-0.444549000
1	-0.733947000	-3.380104000	-1.478037000
1	-1.147723000	-3.514362000	0.333996000

44	-1.033520000	-0.923492000	-0.189671000
17	-0.723749000	-0.657852000	-2.592274000
17	-1.311845000	-1.459626000	2.171631000
6	-2.547837000	0.465948000	-0.141677000
6	-3.897726000	2.293134000	0.005823000
1	-4.120497000	3.340543000	0.128340000
6	-4.698224000	1.221549000	-0.184236000
1	-5.769576000	1.134179000	-0.266255000
7	-3.866384000	0.108066000	-0.272422000
7	-2.584078000	1.821284000	0.027063000
6	-1.468335000	2.724198000	0.173567000
6	-0.961032000	2.970515000	1.461862000
6	-0.992808000	3.384418000	-0.974040000
6	0.090823000	3.888995000	1.575997000
6	0.047861000	4.307042000	-0.804209000
6	0.591044000	4.551079000	0.456902000
1	0.509067000	4.089749000	2.557930000
1	0.431882000	4.833004000	-1.673785000
1	1.401999000	5.264951000	0.568480000
6	-4.367764000	-1.233826000	-0.484129000
6	-4.507106000	-1.700983000	-1.806970000
6	-4.799738000	-1.979241000	0.631893000
6	-4.976257000	-3.009148000	-1.983567000
6	-5.264439000	-3.279346000	0.398725000
6	-5.330412000	-3.799025000	-0.892356000
1	-5.081260000	-3.396274000	-2.992998000
1	-5.589406000	-3.878688000	1.244168000
1	-5.692505000	-4.810939000	-1.051044000
6	-1.553111000	3.096363000	-2.345539000
1	-1.106545000	3.762186000	-3.088087000
1	-1.346585000	2.061496000	-2.642939000
1	-2.639176000	3.233560000	-2.379725000
6	-1.533256000	2.293849000	2.682926000
1	-1.420645000	1.205317000	2.638785000
1	-1.034634000	2.653309000	3.586261000
1	-2.604687000	2.501523000	2.785295000
6	-4.223080000	-0.826153000	-3.002180000
1	-4.736839000	0.137987000	-2.919674000
1	-3.152883000	-0.623407000	-3.106688000
1	-4.569398000	-1.314269000	-3.916607000
6	-4.810938000	-1.403914000	2.025659000
1	-5.299623000	-2.096001000	2.715834000
1	-3.793471000	-1.222949000	2.384498000

1	-5.354312000	-0.453192000	2.061680000

2a-*pro-cis*-**Ru*** → *cis*-**Ru-cycl***

Sum of electronic and zero-point Energies= -2668.203168
Sum of electronic and thermal Energies= -2668.162705
Sum of electronic and thermal Enthalpies= -2668.161761
Sum of electronic and thermal Free Energies= -2668.276303

6	0.262070000	-1.203377000	-3.625792000
6	1.152756000	-1.303394000	-4.704912000
6	0.266218000	-0.009425000	-2.876216000
6	1.997144000	-0.249657000	-5.054802000
6	1.102203000	1.053629000	-3.228492000
6	1.963174000	0.935559000	-4.319803000
1	1.153441000	-2.216178000	-5.293932000
1	2.666531000	-0.350312000	-5.903899000
1	1.090014000	1.958632000	-2.628841000
1	2.611326000	1.765551000	-4.586682000
6	-0.641121000	-2.336100000	-3.288442000
6	-2.038592000	-2.153688000	-3.164672000
6	-0.128529000	-3.627949000	-3.126640000
6	-2.869968000	-3.237160000	-2.864526000
6	-0.953750000	-4.715540000	-2.832950000
6	-2.324237000	-4.513061000	-2.698210000
1	0.944930000	-3.770329000	-3.213200000
1	-3.941192000	-3.102255000	-2.769814000
1	-0.526168000	-5.704435000	-2.700093000
1	-2.982362000	-5.344627000	-2.463016000
8	-0.521947000	0.045617000	-1.753690000
8	-2.491370000	-0.885036000	-3.409132000
6	-1.450560000	1.136071000	-1.660480000
1	-1.820403000	1.430237000	-2.644180000
1	-0.964821000	2.012880000	-1.207298000
6	-2.541561000	0.714756000	-0.719841000
1	-2.209069000	-0.055043000	-0.015383000
6	-3.820272000	-0.506649000	-3.087685000
1	-3.962274000	0.475836000	-3.543495000
1	-4.536579000	-1.198573000	-3.553983000
6	-4.144170000	-0.433914000	-1.604344000
1	-3.821770000	-1.272201000	-0.992276000
6	-5.412790000	0.136494000	-1.251429000
1	-5.981609000	0.621534000	-2.044245000

1	-5.986993000	-0.332210000	-0.457568000
44	-4.163966000	1.569096000	-0.346767000
17	-4.172616000	2.912473000	-2.370076000
17	-4.310206000	0.170230000	1.642877000
6	-4.209271000	3.294383000	0.832451000
6	-3.657901000	5.132559000	2.046539000
1	-2.988929000	5.849245000	2.494671000
6	-5.005967000	5.030999000	2.061897000
1	-5.765083000	5.641219000	2.523876000
7	-5.334070000	3.900423000	1.315737000
7	-3.179330000	4.064429000	1.287423000
6	-1.768142000	3.868721000	1.039530000
6	-1.037934000	3.040011000	1.911316000
6	-1.170008000	4.581142000	-0.016384000
6	0.332294000	2.889893000	1.661715000
6	0.205781000	4.405108000	-0.216028000
6	0.948579000	3.560020000	0.606992000
1	0.917364000	2.247994000	2.313744000
1	0.691956000	4.944184000	-1.024204000
1	2.013580000	3.434026000	0.434500000
6	-6.700515000	3.467377000	1.104357000
6	-7.395741000	3.952752000	-0.020516000
6	-7.308071000	2.654443000	2.081387000
6	-8.712289000	3.511135000	-0.204786000
6	-8.626526000	2.244361000	1.849301000
6	-9.316476000	2.651449000	0.709540000
1	-9.267240000	3.864241000	-1.069238000
1	-9.113701000	1.607647000	2.582029000
1	-10.338687000	2.320884000	0.548371000
6	-1.962858000	5.504893000	-0.908071000
1	-1.301652000	6.005273000	-1.619691000
1	-2.720747000	4.952635000	-1.474073000
1	-2.481299000	6.278425000	-0.330758000
6	-1.685347000	2.352356000	3.087430000
1	-0.930843000	1.842399000	3.691234000
1	-2.203139000	3.071076000	3.732793000
1	-2.426856000	1.612034000	2.768472000
6	-6.783273000	4.943531000	-0.979267000
1	-6.363845000	5.805513000	-0.448695000
1	-5.974967000	4.492635000	-1.563553000
1	-7.541590000	5.313823000	-1.673558000
6	-6.595990000	2.255585000	3.349175000
1	-7.284376000	1.740427000	4.023607000

1	-5.759474000	1.584012000	3.132638000
1	-6.196916000	3.128158000	3.878132000

cis-**Ru-cycl***

Sum of electronic and zero-point Energies= -2668.208429

Sum of electronic and thermal Energies= -2668.167959

Sum of electronic and thermal Enthalpies= -2668.167015

Sum of electronic and thermal Free Energies= -2668.281773

6	5.319998000	0.040316000	-0.372446000
6	6.513256000	0.377749000	-1.026908000
6	4.285667000	0.998761000	-0.345933000
6	6.671662000	1.608648000	-1.664782000
6	4.434895000	2.228242000	-0.992930000
6	5.624729000	2.530598000	-1.656183000
1	7.314074000	-0.355788000	-1.058214000
1	7.601857000	1.839036000	-2.175589000
1	3.625232000	2.950450000	-0.951658000
1	5.734398000	3.489432000	-2.155094000
6	5.161999000	-1.285834000	0.282259000
6	4.064866000	-2.123987000	-0.021103000
6	6.113691000	-1.751211000	1.196590000
6	3.924595000	-3.364809000	0.606753000
6	5.985495000	-2.994129000	1.820025000
6	4.884030000	-3.794342000	1.527273000
1	6.954944000	-1.107585000	1.437773000
1	3.083262000	-4.008006000	0.373456000
1	6.731659000	-3.324175000	2.536167000
1	4.763131000	-4.760308000	2.009295000
8	3.167948000	0.712382000	0.395058000
8	3.219958000	-1.662728000	-0.996459000
6	1.890608000	0.766515000	-0.271225000
1	1.995843000	0.540809000	-1.332193000
1	1.462732000	1.771709000	-0.171145000
6	1.000343000	-0.211127000	0.470040000
1	1.175845000	-0.134153000	1.543938000
6	1.892814000	-2.152334000	-1.108835000
1	1.506169000	-1.716287000	-2.031711000
1	1.888503000	-3.243803000	-1.221677000
6	0.964444000	-1.778903000	0.063215000
1	1.236937000	-2.265368000	1.001313000
6	-0.475111000	-2.353989000	-0.275843000

1	-0.575998000	-2.681890000	-1.311688000
1	-0.838867000	-3.060985000	0.467317000
44	-0.910503000	-0.460116000	0.039621000
17	-0.617929000	0.020534000	-2.313987000
17	-1.211567000	-1.049314000	2.374122000
6	-2.619681000	0.688066000	0.117357000
6	-4.016344000	2.452884000	0.365044000
1	-4.275935000	3.481950000	0.553613000
6	-4.777834000	1.369746000	0.089025000
1	-5.844087000	1.252742000	-0.017512000
7	-3.909531000	0.288734000	-0.061694000
7	-2.689644000	2.023660000	0.377176000
6	-1.570722000	2.914303000	0.610768000
6	-1.124629000	3.106766000	1.932360000
6	-1.032583000	3.615056000	-0.485966000
6	-0.040275000	3.972001000	2.125876000
6	0.045944000	4.472916000	-0.235521000
6	0.548686000	4.638141000	1.053647000
1	0.331577000	4.129006000	3.134068000
1	0.483246000	5.021116000	-1.065017000
1	1.387740000	5.305862000	1.226719000
6	-4.365099000	-1.049006000	-0.383981000
6	-4.471696000	-1.412972000	-1.739860000
6	-4.767839000	-1.894872000	0.666789000
6	-4.915118000	-2.710347000	-2.025245000
6	-5.205566000	-3.180453000	0.325750000
6	-5.263211000	-3.591501000	-1.004150000
1	-4.998431000	-3.019481000	-3.063175000
1	-5.512753000	-3.856720000	1.118247000
1	-5.605376000	-4.593594000	-1.246803000
6	-1.591522000	3.477302000	-1.880291000
1	-1.128259000	4.208259000	-2.547454000
1	-1.403310000	2.477270000	-2.284113000
1	-2.674054000	3.643786000	-1.899309000
6	-1.793526000	2.443914000	3.110559000
1	-1.644743000	1.359194000	3.101830000
1	-1.387798000	2.837542000	4.045638000
1	-2.874098000	2.625584000	3.110352000
6	-4.158732000	-0.450120000	-2.858204000
1	-4.681472000	0.503403000	-2.724474000
1	-3.087427000	-0.231489000	-2.912601000
1	-4.470322000	-0.871369000	-3.817183000
6	-4.761126000	-1.445924000	2.106407000

1	-5.226773000	-2.203758000	2.741176000
1	-3.739741000	-1.278052000	2.462332000
1	-5.315838000	-0.510336000	2.238607000

cis-**Ru-cycl*** → Z-**4a-Ru***

Sum of electronic and zero-point Energies= -2668.194876

Sum of electronic and thermal Energies= -2668.154158

Sum of electronic and thermal Enthalpies= -2668.153214

Sum of electronic and thermal Free Energies= -2668.269382

44	-0.004842000	1.634565000	-0.314757000
6	-2.647609000	-4.156915000	-0.159988000
6	-3.343943000	-5.162769000	0.524706000
6	-3.220617000	-2.869621000	-0.207088000
6	-4.551630000	-4.901162000	1.172871000
6	-4.424649000	-2.599960000	0.448436000
6	-5.086555000	-3.613273000	1.142683000
6	-1.352483000	-4.453352000	-0.830836000
6	-0.183136000	-3.726747000	-0.516426000
6	-1.256569000	-5.479965000	-1.777214000
6	1.022193000	-4.005152000	-1.165813000
6	-0.053161000	-5.770822000	-2.422678000
6	1.082780000	-5.023815000	-2.119822000
8	-2.600720000	-1.914686000	-0.976120000
8	-0.304194000	-2.806695000	0.496740000
6	-2.202734000	-0.692112000	-0.320936000
6	-0.922417000	-0.275783000	-1.019327000
6	0.557441000	-1.668608000	0.544434000
6	0.347302000	-0.754447000	-0.656472000
6	1.658831000	1.039295000	0.170373000
17	-0.805308000	1.270281000	1.953538000
17	0.755477000	1.897812000	-2.611197000
6	-0.100847000	3.683739000	-0.067627000
6	-0.932001000	5.798586000	-0.048312000
6	0.365696000	5.870317000	0.325578000
7	0.868177000	4.568763000	0.309882000
7	-1.208105000	4.453328000	-0.285352000
6	-2.506468000	3.960619000	-0.695165000
6	-2.791141000	3.866127000	-2.071950000
6	-3.461682000	3.673056000	0.300443000
6	-4.046256000	3.362435000	-2.437448000

6	-4.701847000	3.177221000	-0.120095000
6	-4.985486000	3.003857000	-1.473380000
6	2.237526000	4.264620000	0.661112000
6	2.535719000	3.967043000	2.003829000
6	3.224182000	4.349044000	-0.338630000
6	3.870394000	3.685630000	2.321544000
6	4.543840000	4.061461000	0.032139000
6	4.863749000	3.721655000	1.345010000
6	-3.191116000	3.911191000	1.764962000
6	-1.815746000	4.323168000	-3.127750000
6	1.472973000	3.963282000	3.074507000
6	2.892536000	4.739411000	-1.757399000
1	-2.908624000	-6.157170000	0.569322000
1	-5.063831000	-5.695902000	1.706964000
1	-4.847957000	-1.601777000	0.390654000
1	-6.023242000	-3.395995000	1.648213000
1	-2.154824000	-6.038252000	-2.025341000
1	1.919871000	-3.447630000	-0.921128000
1	-0.009930000	-6.563347000	-3.163374000
1	2.025421000	-5.230804000	-2.618385000
1	-2.060216000	-0.855712000	0.746278000
1	-2.976777000	0.072291000	-0.458984000
1	-1.046224000	-0.029486000	-2.072366000
1	0.279171000	-1.160011000	1.468705000
1	1.609478000	-1.968854000	0.617706000
1	1.083430000	-0.791080000	-1.454121000
1	1.875292000	0.724477000	1.196216000
1	2.489809000	1.014180000	-0.541556000
1	-1.685026000	6.560281000	-0.170389000
1	0.984379000	6.708720000	0.601533000
1	-4.286541000	3.268984000	-3.492495000
1	-5.452013000	2.938433000	0.628229000
1	-5.953050000	2.615605000	-1.778601000
1	4.126547000	3.447608000	3.350034000
1	5.323916000	4.113518000	-0.721969000
1	5.893685000	3.502134000	1.611970000
1	-4.104232000	3.763200000	2.346719000
1	-2.429147000	3.222233000	2.142095000
1	-2.837757000	4.931944000	1.947349000
1	-0.940556000	3.667558000	-3.178517000
1	-2.297107000	4.326889000	-4.108776000
1	-1.455422000	5.337773000	-2.925517000
1	0.926747000	4.913003000	3.098965000

1	0.736544000	3.169229000	2.909705000
1	1.924973000	3.812255000	4.057949000
1	3.807502000	4.852231000	-2.344243000
1	2.265264000	3.981855000	-2.239731000
1	2.348895000	5.689802000	-1.797319000

Z-**4a**-Ru*

Sum of electronic and zero-point Energies= -2668.196308

Sum of electronic and thermal Energies= -2668.154304

Sum of electronic and thermal Enthalpies= -2668.153360

Sum of electronic and thermal Free Energies= -2668.273423

6	5.529038000	0.096558000	-0.542510000
6	6.628092000	0.292016000	-1.390882000
6	4.535290000	1.095786000	-0.506938000
6	6.728367000	1.420251000	-2.205624000
6	4.625971000	2.222643000	-1.327665000
6	5.718526000	2.382205000	-2.180609000
1	7.397978000	-0.473629000	-1.426813000
1	7.584287000	1.540055000	-2.863079000
1	3.849868000	2.980114000	-1.275341000
1	5.782753000	3.262142000	-2.814406000
6	5.435840000	-1.120350000	0.309385000
6	4.320074000	-1.982970000	0.236815000
6	6.469227000	-1.451144000	1.193647000
6	4.238834000	-3.108403000	1.061007000
6	6.400895000	-2.581261000	2.010303000
6	5.277723000	-3.402557000	1.947219000
1	7.327375000	-0.787891000	1.255581000
1	3.381073000	-3.769797000	1.005267000
1	7.210526000	-2.806797000	2.697485000
1	5.202858000	-4.280775000	2.582166000
8	3.517558000	0.964439000	0.407932000
8	3.387553000	-1.680047000	-0.726151000
6	2.170998000	0.943125000	-0.109378000
1	2.164083000	0.606125000	-1.145489000
1	1.738035000	1.949578000	-0.064054000
6	1.411077000	-0.001956000	0.798628000
1	1.166657000	0.379066000	1.788798000
6	2.023836000	-2.069894000	-0.548551000
1	1.536044000	-1.770329000	-1.478290000

1	1.927786000	-3.157236000	-0.445436000
6	1.383557000	-1.364630000	0.634628000
1	1.061856000	-1.963114000	1.481414000
6	-1.237470000	-2.033265000	-0.113861000
1	-1.129545000	-2.457868000	-1.118257000
1	-1.529719000	-2.726036000	0.683248000
44	-0.899539000	-0.279938000	0.226458000
17	-0.368663000	0.075774000	-2.120566000
17	-1.238616000	-0.646019000	2.605589000
6	-2.771319000	0.500662000	-0.015774000
6	-4.423774000	2.062961000	-0.090825000
1	-4.845995000	3.051758000	-0.012936000
6	-4.981674000	0.870241000	-0.396788000
1	-5.995273000	0.597718000	-0.641877000
7	-3.962278000	-0.082130000	-0.349851000
7	-3.070515000	1.827128000	0.140855000
6	-2.127036000	2.867091000	0.493197000
6	-1.953717000	3.190021000	1.854114000
6	-1.486429000	3.575546000	-0.543480000
6	-1.017278000	4.183603000	2.166673000
6	-0.561285000	4.560560000	-0.175229000
6	-0.313016000	4.848449000	1.165380000
1	-0.855712000	4.442240000	3.209020000
1	-0.047246000	5.114775000	-0.955260000
1	0.409656000	5.615183000	1.429753000
6	-4.207985000	-1.478380000	-0.623966000
6	-4.146319000	-1.923721000	-1.958056000
6	-4.581415000	-2.317115000	0.443326000
6	-4.422993000	-3.274903000	-2.200777000
6	-4.848666000	-3.659365000	0.146893000
6	-4.762948000	-4.136591000	-1.159612000
1	-4.376284000	-3.645667000	-3.220709000
1	-5.133672000	-4.328951000	0.953357000
1	-4.976149000	-5.180981000	-1.369276000
6	-1.803787000	3.330119000	-1.997432000
1	-1.296752000	4.067858000	-2.624192000
1	-1.482631000	2.333288000	-2.314475000
1	-2.879739000	3.409680000	-2.188784000
6	-2.763678000	2.534067000	2.943872000
1	-2.499516000	1.477721000	3.053888000
1	-2.583833000	3.032504000	3.899534000
1	-3.837144000	2.589557000	2.731883000
6	-3.801399000	-0.991668000	-3.092603000

1	-4.472832000	-0.126165000	-3.120260000
1	-2.779893000	-0.607558000	-2.996905000
1	-3.884642000	-1.510923000	-4.050469000
6	-4.695639000	-1.800758000	1.855649000
1	-5.090153000	-2.577988000	2.514705000
1	-3.722030000	-1.481295000	2.244142000
1	-5.367083000	-0.936880000	1.914192000

2a-*pro*-*trans*-**Ru***

Sum of electronic and zero-point Energies= -2668.206402

Sum of electronic and thermal Energies= -2668.164861

Sum of electronic and thermal Enthalpies= -2668.163917

Sum of electronic and thermal Free Energies= -2668.281060

6	4.320072000	0.029487000	0.231410000
6	5.333329000	0.669047000	-0.490158000
6	3.189046000	0.785376000	0.614327000
6	5.231134000	2.012074000	-0.858910000
6	3.073502000	2.125951000	0.236768000
6	4.093869000	2.731923000	-0.500502000
1	6.202765000	0.088432000	-0.785484000
1	6.025011000	2.482730000	-1.430692000
1	2.204678000	2.706781000	0.521852000
1	3.993259000	3.775162000	-0.787348000
6	4.443267000	-1.403213000	0.614169000
6	3.477699000	-2.351297000	0.204305000
6	5.524753000	-1.846526000	1.380560000
6	3.583522000	-3.685378000	0.608642000
6	5.650453000	-3.183258000	1.767215000
6	4.669952000	-4.094661000	1.388064000
1	6.265237000	-1.117138000	1.696528000
1	2.837343000	-4.416458000	0.320707000
1	6.494865000	-3.499852000	2.371520000
1	4.739742000	-5.134956000	1.693091000
8	2.292907000	0.137368000	1.422817000
8	2.500781000	-1.869500000	-0.627911000
6	0.916692000	0.525474000	1.489334000
1	0.529001000	0.066381000	2.396858000
1	0.801025000	1.614078000	1.586959000
6	0.171515000	0.066931000	0.264000000
1	0.725772000	0.248852000	-0.666201000

6	1.364955000	-2.658892000	-0.943659000
1	0.935964000	-2.222690000	-1.849220000
1	1.658764000	-3.690747000	-1.182064000
6	0.331348000	-2.681414000	0.155403000
1	0.688459000	-2.593917000	1.178546000
6	-0.966316000	-3.070803000	-0.074146000
1	-1.285871000	-3.333918000	-1.079469000
1	-1.588677000	-3.386732000	0.757994000
44	-1.41000	-0.828998000	0.109972000
17	-1.212689000	-0.719081000	-2.313813000
17	-1.672700000	-1.219282000	2.507414000
6	-2.914128000	0.601425000	0.112547000
6	-4.218022000	2.466846000	0.150778000
1	-4.416026000	3.523054000	0.233507000
6	-5.038622000	1.412300000	-0.046954000
1	-6.107343000	1.350676000	-0.174155000
7	-4.235130000	0.275210000	-0.068057000
7	-2.920278000	1.960099000	0.247824000
6	-1.802095000	2.838047000	0.494116000
6	-1.503537000	3.175716000	1.826854000
6	-1.133163000	3.402822000	-0.606298000
6	-0.478909000	4.105724000	2.043854000
6	-0.114285000	4.325997000	-0.336537000
6	0.208894000	4.676766000	0.973370000
1	-0.227679000	4.385050000	3.063047000
1	0.418985000	4.777474000	-1.168078000
1	0.995589000	5.401954000	1.161439000
6	-4.768239000	-1.059181000	-0.250558000
6	-4.949679000	-1.541776000	-1.562789000
6	-5.190994000	-1.778609000	0.885845000
6	-5.447075000	-2.842761000	-1.708671000
6	-5.683137000	-3.074282000	0.683269000
6	-5.787845000	-3.611329000	-0.597748000
1	-5.582100000	-3.242632000	-2.709450000
1	-6.002108000	-3.653832000	1.544696000
1	-6.170841000	-4.618926000	-0.733059000
6	-1.503055000	3.048804000	-2.025174000
1	-1.359028000	1.981354000	-2.225206000
1	-2.555478000	3.275727000	-2.230794000
1	-0.893117000	3.616540000	-2.731898000
6	-2.249402000	2.560095000	2.985350000
1	-2.121510000	1.471844000	3.008719000
1	-1.889227000	2.969183000	3.932398000

1	-3.325448000	2.757276000	2.922133000
6	-4.680269000	-0.687901000	-2.776051000
1	-5.233329000	0.256972000	-2.726677000
1	-3.616777000	-0.449861000	-2.867760000
1	-4.992896000	-1.211481000	-3.682827000
6	-5.174832000	-1.178352000	2.269371000
1	-5.700030000	-1.832576000	2.969661000
1	-4.152003000	-1.039769000	2.631959000
1	-5.670215000	-0.201273000	2.285287000

2a-*pro-trans*-**Ru*** → *trans*-**Ru-cycl***

Sum of electronic and zero-point Energies= -2668.204309

Sum of electronic and thermal Energies= -2668.163783

Sum of electronic and thermal Enthalpies= -2668.162838

Sum of electronic and thermal Free Energies= -2668.277728

6	4.420712000	-0.051650000	0.231802000
6	5.411843000	0.618403000	-0.492716000
6	3.306941000	0.687484000	0.687704000
6	5.300666000	1.977527000	-0.793873000
6	3.182135000	2.044395000	0.379186000
6	4.178408000	2.682302000	-0.364161000
1	6.268667000	0.050880000	-0.844873000
1	6.076323000	2.473639000	-1.369175000
1	2.325318000	2.611809000	0.723452000
1	4.072651000	3.738140000	-0.598021000
6	4.540977000	-1.501989000	0.541428000
6	3.549516000	-2.415267000	0.116733000
6	5.636141000	-1.994121000	1.257420000
6	3.645180000	-3.767411000	0.456777000
6	5.748980000	-3.348115000	1.581814000
6	4.743699000	-4.227013000	1.189153000
1	6.396061000	-1.290634000	1.585783000
1	2.884486000	-4.474857000	0.147946000
1	6.603441000	-3.704559000	2.148685000
1	4.805912000	-5.281149000	1.444311000
8	2.436971000	0.007103000	1.501305000
8	2.566575000	-1.884684000	-0.681168000
6	1.047170000	0.336305000	1.553915000
1	0.667268000	-0.131371000	2.460342000
1	0.881733000	1.418862000	1.648974000

6	0.312578000	-0.134317000	0.325015000
1	0.861662000	0.045118000	-0.605527000
6	1.330100000	-2.556785000	-0.865325000
1	0.900594000	-2.160104000	-1.787722000
1	1.483227000	-3.633495000	-1.008979000
6	0.355071000	-2.359409000	0.281891000
1	0.779177000	-2.358518000	1.283421000
6	-0.969638000	-2.854945000	0.143718000
1	-1.269838000	-3.254837000	-0.823445000
1	-1.462360000	-3.264821000	1.020539000
44	-1.436919000	-0.771546000	0.173722000
17	-1.226185000	-0.703659000	-2.246318000
17	-1.734507000	-1.021049000	2.582796000
6	-3.002306000	0.606544000	0.096335000
6	-4.285669000	2.481168000	0.090954000
1	-4.476461000	3.540093000	0.155106000
6	-5.112175000	1.429817000	-0.105275000
1	-6.179428000	1.374560000	-0.246776000
7	-4.317023000	0.285540000	-0.100802000
7	-2.994878000	1.965159000	0.215133000
6	-1.855860000	2.822523000	0.453749000
6	-1.550027000	3.173407000	1.781692000
6	-1.164097000	3.345805000	-0.653710000
6	-0.480559000	4.054972000	1.986303000
6	-0.102375000	4.222343000	-0.395305000
6	0.238732000	4.572552000	0.909902000
1	-0.222962000	4.342652000	3.001564000
1	0.450149000	4.638597000	-1.232556000
1	1.059062000	5.261968000	1.089097000
6	-4.855413000	-1.047891000	-0.279505000
6	-5.022090000	-1.538227000	-1.590045000
6	-5.285238000	-1.760219000	0.857694000
6	-5.530145000	-2.835189000	-1.733339000
6	-5.786785000	-3.052712000	0.657703000
6	-5.888823000	-3.594181000	-0.621509000
1	-5.655888000	-3.240981000	-2.732948000
1	-6.113684000	-3.626723000	1.519892000
1	-6.279703000	-4.598980000	-0.754856000
6	-1.551338000	3.000848000	-2.070161000
1	-1.409678000	1.934660000	-2.277790000
1	-2.605194000	3.231042000	-2.263414000
1	-0.947385000	3.571636000	-2.779621000
6	-2.340641000	2.637537000	2.949683000

1	-2.243377000	1.549411000	3.030580000
1	-1.990641000	3.084457000	3.883409000
1	-3.408369000	2.862408000	2.849639000
6	-4.714722000	-0.700934000	-2.806088000
1	-5.240894000	0.259711000	-2.772738000
1	-3.643699000	-0.492463000	-2.883992000
1	-5.028897000	-1.223146000	-3.713109000
6	-5.260557000	-1.158605000	2.240611000
1	-5.793119000	-1.805288000	2.942328000
1	-4.236050000	-1.030005000	2.603001000
1	-5.743283000	-0.175203000	2.256789000

trans-**Ru-cycl***

Sum of electronic and zero-point Energies= -2668.211650

Sum of electronic and thermal Energies= -2668.171017

Sum of electronic and thermal Enthalpies= -2668.170073

Sum of electronic and thermal Free Energies= -2668.285752

6	5.107697000	0.260163000	-0.272784000
6	6.048934000	0.902030000	-1.084507000
6	4.019188000	1.013036000	0.218533000
6	5.911449000	2.246521000	-1.435624000
6	3.870465000	2.356850000	-0.136531000
6	4.814254000	2.966131000	-0.966704000
1	6.886343000	0.323158000	-1.463900000
1	6.647767000	2.720406000	-2.077521000
1	3.035503000	2.935801000	0.242644000
1	4.690271000	4.011561000	-1.235671000
6	5.257685000	-1.177767000	0.084140000
6	4.271630000	-2.121091000	-0.279406000
6	6.387367000	-1.630042000	0.773821000
6	4.415342000	-3.465105000	0.074701000
6	6.539458000	-2.972618000	1.126499000
6	5.545136000	-3.884616000	0.781681000
1	7.144535000	-0.904046000	1.056281000
1	3.669033000	-4.196395000	-0.214878000
1	7.420087000	-3.297131000	1.672246000
1	5.644675000	-4.932664000	1.049855000
8	3.203602000	0.370612000	1.114772000
8	3.248647000	-1.642141000	-1.062603000
6	1.796439000	0.647476000	1.169918000

1	1.469810000	0.282966000	2.141796000
1	1.591860000	1.721777000	1.120544000
6	1.082328000	-0.064632000	0.035059000
1	1.438689000	0.262624000	-0.943421000
6	1.955332000	-2.226482000	-1.031531000
1	1.487814000	-1.970913000	-1.984345000
1	2.005262000	-3.316676000	-0.950672000
6	1.085110000	-1.671711000	0.117141000
1	1.481908000	-1.905375000	1.107559000
6	-0.332975000	-2.359961000	0.026997000
1	-0.507269000	-2.878363000	-0.916656000
1	-0.591122000	-2.927617000	0.919288000
44	-0.857179000	-0.456940000	0.055675000
17	-0.783784000	-0.429915000	-2.370622000
17	-0.970220000	-0.612370000	2.471032000
6	-2.609432000	0.626594000	-0.000448000
6	-4.070080000	2.357097000	-0.017055000
1	-4.366743000	3.393085000	0.008768000
6	-4.791788000	1.217285000	-0.111362000
1	-5.853513000	1.046221000	-0.184523000
7	-3.884637000	0.157945000	-0.101115000
7	-2.728306000	1.982861000	0.052275000
6	-1.647441000	2.939261000	0.181451000
6	-1.275299000	3.359255000	1.473190000
6	-1.076682000	3.474351000	-0.989118000
6	-0.235384000	4.292216000	1.572615000
6	-0.040548000	4.403991000	-0.833169000
6	0.385417000	4.800427000	0.432912000
1	0.075433000	4.628427000	2.557509000
1	0.422575000	4.825989000	-1.720228000
1	1.186633000	5.527468000	0.531771000
6	-4.295317000	-1.230343000	-0.180357000
6	-4.479735000	-1.806190000	-1.451843000
6	-4.581360000	-1.915785000	1.015640000
6	-4.875062000	-3.148658000	-1.498938000
6	-4.974108000	-3.255977000	0.911475000
6	-5.103270000	-3.872557000	-0.330798000
1	-5.014407000	-3.620058000	-2.467475000
1	-5.191501000	-3.810446000	1.819773000
1	-5.408811000	-4.913364000	-0.389701000
6	-1.564863000	3.096603000	-2.365027000
1	-1.347951000	2.047612000	-2.591758000
1	-2.647833000	3.235097000	-2.457831000

1	-1.081010000	3.718269000	-3.122169000
6	-1.972624000	2.855922000	2.712278000
1	-1.782487000	1.789232000	2.869779000
1	-1.620839000	3.400017000	3.592140000
1	-3.057788000	2.991147000	2.645363000
6	-4.294603000	-1.019194000	-2.724904000
1	-4.892829000	-0.101064000	-2.719955000
1	-3.247954000	-0.731310000	-2.864104000
1	-4.604556000	-1.615480000	-3.586538000
6	-4.506863000	-1.244868000	2.364423000
1	-4.913110000	-1.902858000	3.136497000
1	-3.474304000	-0.999353000	2.632120000
1	-5.082412000	-0.312640000	2.381058000

trans-**Ru-cycl*** → E-**4a**-Ru*

Sum of electronic and zero-point Energies= -2668.184764

Sum of electronic and thermal Energies= -2668.143604

Sum of electronic and thermal Enthalpies= -2668.142660

Sum of electronic and thermal Free Energies= -2668.259566

44	-0.107420000	1.700765000	0.935636000
6	-1.471750000	-3.980439000	-0.812248000
6	-0.826078000	-5.179283000	-1.138761000
6	-1.420915000	-3.540014000	0.525264000
6	-0.125468000	-5.914141000	-0.180995000
6	-0.724253000	-4.273556000	1.490475000
6	-0.071815000	-5.454406000	1.134557000
1	-0.864450000	-5.524802000	-2.167940000
1	0.374655000	-6.836091000	-0.461869000
1	-0.714281000	-3.933871000	2.521422000
1	0.464876000	-6.019223000	1.891704000
6	-2.216238000	-3.209186000	-1.855264000
6	-1.665506000	-2.050754000	-2.439889000
6	-3.475046000	-3.638735000	-2.292429000
6	-2.375142000	-1.342302000	-3.415087000
6	-4.189181000	-2.933950000	-3.262670000
6	-3.636891000	-1.780543000	-3.818761000
1	-3.901829000	-4.532262000	-1.845650000
1	-1.930569000	-0.465269000	-3.874617000
1	-5.167906000	-3.282585000	-3.578236000
1	-4.178510000	-1.224939000	-4.579344000

8	-2.151937000	-2.417881000	0.850246000
8	-0.384136000	-1.707969000	-2.073490000
6	-1.530406000	-1.357087000	1.603582000
1	-2.335619000	-0.663931000	1.831848000
1	-1.113487000	-1.722365000	2.547120000
6	-0.474014000	-0.734078000	0.719715000
1	0.492156000	-1.235997000	0.722254000
6	-0.086246000	-0.341150000	-1.721934000
1	0.995137000	-0.314427000	-1.608332000
1	-0.359758000	0.352566000	-2.520829000
6	-0.795147000	-0.022480000	-0.420265000
1	-1.836827000	0.303309000	-0.511601000
6	1.319512000	1.133611000	1.902548000
1	1.203313000	0.822406000	2.946670000
1	2.321433000	1.056706000	1.468164000
17	-1.597592000	1.507285000	2.850505000
17	1.435276000	2.182750000	-0.910617000
6	-0.065199000	3.750897000	1.325063000
6	-0.668501000	5.941956000	1.240739000
1	-1.269447000	6.785358000	0.941464000
6	0.434713000	5.856383000	2.017527000
1	0.997980000	6.609948000	2.543435000
7	0.799156000	4.511403000	2.063064000
7	-0.968445000	4.647810000	0.824445000
6	-2.106716000	4.338846000	-0.018566000
6	-1.940718000	4.364503000	-1.418042000
6	-3.361037000	4.132886000	0.591100000
6	-3.052855000	4.039945000	-2.205749000
6	-4.439679000	3.814325000	-0.243142000
6	-4.284107000	3.744421000	-1.625629000
1	-2.946910000	4.041628000	-3.286774000
1	-5.412739000	3.634963000	0.204543000
1	-5.133436000	3.492918000	-2.254404000
6	1.949176000	4.069287000	2.822084000
6	1.767193000	3.704999000	4.170098000
6	3.219174000	4.126338000	2.216130000
6	2.899005000	3.300824000	4.889296000
6	4.317934000	3.713095000	2.979766000
6	4.160154000	3.289790000	4.297695000
1	2.782321000	3.005804000	5.928182000
1	5.306664000	3.737019000	2.530652000
1	5.025804000	2.972727000	4.872360000
6	-3.569782000	4.293386000	2.075880000

1	-4.631202000	4.204228000	2.319876000
1	-3.022209000	3.531663000	2.637776000
1	-3.227275000	5.274940000	2.422366000
6	-0.644842000	4.780775000	-2.067194000
1	0.156477000	4.063142000	-1.868399000
1	-0.774545000	4.862485000	-3.149261000
1	-0.310762000	5.756038000	-1.695280000
6	0.418291000	3.773987000	4.840624000
1	-0.008746000	4.781119000	4.770215000
1	-0.298577000	3.085656000	4.382016000
1	0.507926000	3.522870000	5.900371000
6	3.413197000	4.636761000	0.810643000
1	4.475181000	4.645492000	0.553494000
1	2.885519000	4.011398000	0.084108000
1	3.034122000	5.659504000	0.701650000

E-**4a**-Ru*

Sum of electronic and zero-point Energies= -2668.192437

Sum of electronic and thermal Energies= -2668.150817

Sum of electronic and thermal Enthalpies= -2668.149873

Sum of electronic and thermal Free Energies= -2668.268397

6	5.052949000	-0.666496000	-0.732219000
6	5.988845000	-1.497316000	-1.364470000
6	4.240565000	0.151679000	-1.540805000
6	6.111890000	-1.535949000	-2.753010000
6	4.366858000	0.123306000	-2.934162000
6	5.295353000	-0.722108000	-3.538935000
1	6.620194000	-2.128722000	-0.745884000
1	6.840703000	-2.194492000	-3.215951000
1	3.745714000	0.776968000	-3.538569000
1	5.385201000	-0.734989000	-4.621502000
6	4.976741000	-0.640427000	0.763077000
6	4.090006000	-1.467580000	1.478895000
6	5.838593000	0.189993000	1.493053000
6	4.070684000	-1.449470000	2.877636000
6	5.817005000	0.218668000	2.887402000
6	4.928417000	-0.604434000	3.580251000
1	6.528123000	0.828274000	0.947862000
1	3.390373000	-2.108576000	3.407925000
1	6.491771000	0.876227000	3.427444000

1	4.906817000	-0.598633000	4.666372000
8	3.363747000	1.027144000	-0.929001000
8	3.279816000	-2.334797000	0.772207000
6	1.970068000	0.913490000	-1.286620000
1	1.484935000	1.727781000	-0.741751000
1	1.811943000	1.077381000	-2.355193000
6	1.456725000	-0.448630000	-0.862814000
1	1.513793000	-1.234470000	-1.610710000
6	1.853235000	-2.178582000	0.936744000
1	1.418361000	-2.951108000	0.299252000
1	1.548147000	-2.356180000	1.971347000
6	1.466645000	-0.792373000	0.477642000
1	1.679528000	-0.004551000	1.198226000
6	-1.290569000	-2.071521000	-0.413288000
1	-1.459287000	-2.444493000	-1.428121000
1	-1.488762000	-2.755645000	0.417801000
44	-0.691756000	-0.393240000	-0.118884000
17	-0.912054000	0.031934000	-2.534848000
17	-0.852102000	-0.776110000	2.305256000
6	-2.546386000	0.599166000	0.083850000
6	-3.984393000	2.334841000	0.383332000
1	-4.264675000	3.365336000	0.530179000
6	-4.725447000	1.208136000	0.281314000
1	-5.790681000	1.048119000	0.319459000
7	-3.837056000	0.150187000	0.098313000
7	-2.652932000	1.951018000	0.260335000
6	-1.556801000	2.899006000	0.293573000
6	-0.958715000	3.202704000	1.533236000
6	-1.204476000	3.560950000	-0.900590000
6	0.116363000	4.101504000	1.527183000
6	-0.127707000	4.454657000	-0.848717000
6	0.543646000	4.704451000	0.346643000
1	0.605439000	4.339013000	2.467297000
1	0.173120000	4.966026000	-1.758430000
1	1.381165000	5.395799000	0.363593000
6	-4.311174000	-1.209273000	-0.052205000
6	-4.592479000	-1.684935000	-1.347149000
6	-4.572542000	-1.963187000	1.107669000
6	-5.064443000	-2.998528000	-1.464262000
6	-5.045626000	-3.269770000	0.934894000
6	-5.274152000	-3.789807000	-0.337248000
1	-5.278900000	-3.392151000	-2.453651000
1	-5.243930000	-3.876001000	1.814066000

1	-5.639551000	-4.806657000	-0.449284000
6	-1.975637000	3.371030000	-2.182998000
1	-1.586517000	4.035047000	-2.958683000
1	-1.903803000	2.341558000	-2.544724000
1	-3.037640000	3.604289000	-2.045430000
6	-1.478641000	2.647920000	2.835590000
1	-1.344501000	1.564320000	2.899191000
1	-0.955665000	3.109297000	3.676782000
1	-2.548864000	2.853566000	2.952076000
6	-4.431411000	-0.817533000	-2.570397000
1	-5.010563000	0.108677000	-2.478883000
1	-3.387002000	-0.532101000	-2.731320000
1	-4.784982000	-1.345824000	-3.459231000
6	-4.387537000	-1.392577000	2.491419000
1	-4.677524000	-2.126904000	3.246822000
1	-3.346466000	-1.106666000	2.668514000
1	-5.005397000	-0.499213000	2.639800000

E-**4a**

E = -2290332.12 kcal (+ Br^-)

REMARK Spartan '10 exported M0001

HETATM	1	H	UNK	0001	-1.821	2.381	-4.349
HETATM	2	C	UNK	0001	-1.689	2.329	-3.270
HETATM	3	C	UNK	0001	-1.324	2.176	-0.499
HETATM	4	C	UNK	0001	-0.999	1.214	-2.726
HETATM	5	C	UNK	0001	-2.175	3.327	-2.457
HETATM	6	C	UNK	0001	-1.987	3.246	-1.058
HETATM	7	C	UNK	0001	-0.811	1.123	-1.306
HETATM	8	H	UNK	0001	-2.699	4.177	-2.886
HETATM	9	H	UNK	0001	-2.368	4.037	-0.417
HETATM	10	C	UNK	0001	-0.477	0.190	-3.559
HETATM	11	C	UNK	0001	0.202	-0.871	-3.016
HETATM	12	C	UNK	0001	-0.114	-0.003	-0.747
HETATM	13	C	UNK	0001	0.390	-0.967	-1.613
HETATM	14	C	UNK	0001	0.104	-0.098	0.731
HETATM	15	C	UNK	0001	0.494	-0.235	3.542
HETATM	16	C	UNK	0001	-0.937	-0.422	1.595
HETATM	17	C	UNK	0001	1.395	0.194	1.293
HETATM	18	C	UNK	0001	1.590	0.115	2.711

```
HETATM   19  C   UNK  0001      -0.738  -0.495   2.997
HETATM   20  H   UNK  0001      -1.591  -0.742   3.623
HETATM   21  H   UNK  0001       0.641  -0.281   4.619
HETATM   22  C   UNK  0001       2.506   0.570   0.488
HETATM   23  C   UNK  0001       3.734   0.844   1.049
HETATM   24  C   UNK  0001       2.869   0.401   3.258
HETATM   25  H   UNK  0001       2.998   0.336   4.336
HETATM   26  C   UNK  0001       3.923   0.757   2.448
HETATM   27  H   UNK  0001       4.897   0.974   2.878
HETATM   28  O   UNK  0001       1.121  -2.039  -1.142
HETATM   29  O   UNK  0001      -2.213  -0.658   1.122
HETATM   30  C   UNK  0001       0.386  -3.293  -1.061
HETATM   31  H   UNK  0001      -0.113  -3.491  -2.018
HETATM   32  H   UNK  0001       1.155  -4.051  -0.896
HETATM   33  C   UNK  0001      -0.596  -3.193   0.066
HETATM   34  H   UNK  0001      -0.198  -3.336   1.070
HETATM   35  H   UNK  0001       4.565   1.129   0.411
HETATM   36  H   UNK  0001       2.376   0.643  -0.586
HETATM   37  H   UNK  0001       0.627  -1.649  -3.643
HETATM   38  H   UNK  0001      -0.611   0.266  -4.635
HETATM   39  H   UNK  0001      -1.186   2.127   0.575
HETATM   40  C   UNK  0001      -1.821  -2.681  -0.098
HETATM   41  H   UNK  0001      -2.203  -2.494  -1.100
HETATM   42  C   UNK  0001      -2.582  -2.062   1.035
HETATM   43  H   UNK  0001      -2.367  -2.562   1.988
HETATM   44  H   UNK  0001      -3.663  -2.052   0.874
HETATM   45 Br   UNK  0001       1.308   5.119   0.372
CONECT    1    2
CONECT    2    1    5    4
CONECT    3    6    7   39
CONECT    4    7    2   10
CONECT    5    2    6    8
CONECT    6    5    3    9
CONECT    7    3    4   12
CONECT    8    5
CONECT    9    6
CONECT   10   11    4   38
CONECT   11   10   13   37
CONECT   12    7   13   14
CONECT   13   12   11   28
CONECT   14   17   16   12
CONECT   15   18   19   21
CONECT   16   19   14   29
```

CONECT 17 14 18 22
CONECT 18 17 15 24
CONECT 19 15 16 20
CONECT 20 19
CONECT 21 15
CONECT 22 23 17 36
CONECT 23 22 26 35
CONECT 24 25 18 26
CONECT 25 24
CONECT 26 27 24 23
CONECT 27 26
CONECT 28 30 13
CONECT 29 16 42
CONECT 30 31 32 28 33
CONECT 31 30
CONECT 32 30
CONECT 33 34 30 40
CONECT 34 33
CONECT 35 23
CONECT 36 22
CONECT 37 11
CONECT 38 10
CONECT 39 3
CONECT 40 41 33 42
CONECT 41 40
CONECT 42 43 44 40 29
CONECT 43 42
CONECT 44 42
END

Z-**4a**

E = -675098.70 kcal

REMARK Spartan '10 exported M0001
HETATM 1 H UNK 0001 3.910 -1.847 3.273
HETATM 2 C UNK 0001 2.875 -1.836 2.937
HETATM 3 C UNK 0001 0.217 -1.785 2.059
HETATM 4 C UNK 0001 2.521 -0.979 1.861
HETATM 5 C UNK 0001 1.934 -2.633 3.548
HETATM 6 C UNK 0001 0.591 -2.599 3.105
HETATM 7 C UNK 0001 1.164 -0.957 1.394

```
HETATM    8  H   UNK  0001       2.217  -3.283   4.372
HETATM    9  H   UNK  0001      -0.153  -3.221   3.596
HETATM   10  C   UNK  0001       3.479  -0.137   1.240
HETATM   11  C   UNK  0001       3.115   0.699   0.213
HETATM   12  C   UNK  0001       0.800  -0.092   0.309
HETATM   13  C   UNK  0001       1.775   0.724  -0.247
HETATM   14  C   UNK  0001      -0.596  -0.048  -0.224
HETATM   15  C   UNK  0001      -3.240   0.105  -1.217
HETATM   16  C   UNK  0001      -1.374   1.087  -0.030
HETATM   17  C   UNK  0001      -1.143  -1.145  -0.967
HETATM   18  C   UNK  0001      -2.486  -1.069  -1.465
HETATM   19  C   UNK  0001      -2.700   1.160  -0.524
HETATM   20  H   UNK  0001      -3.298   2.047  -0.345
HETATM   21  H   UNK  0001      -4.261   0.164  -1.588
HETATM   22  C   UNK  0001      -0.390  -2.316  -1.266
HETATM   23  C   UNK  0001      -0.939  -3.351  -1.990
HETATM   24  C   UNK  0001      -3.024  -2.157  -2.203
HETATM   25  H   UNK  0001      -4.047  -2.082  -2.565
HETATM   26  C   UNK  0001      -2.272  -3.279  -2.460
HETATM   27  H   UNK  0001      -2.693  -4.106  -3.026
HETATM   28  O   UNK  0001       1.437   1.552  -1.294
HETATM   29  O   UNK  0001      -0.819   2.110   0.705
HETATM   30  C   UNK  0001      -1.193   3.459   0.396
HETATM   31  H   UNK  0001      -2.258   3.625   0.599
HETATM   32  C   UNK  0001       1.497   2.968  -1.007
HETATM   33  H   UNK  0001       2.430   3.375  -1.418
HETATM   34  H   UNK  0001       1.504   3.117   0.076
HETATM   35  H   UNK  0001      -0.340  -4.232  -2.206
HETATM   36  H   UNK  0001       0.635  -2.386  -0.920
HETATM   37  H   UNK  0001       3.846   1.337  -0.276
HETATM   38  H   UNK  0001       4.509  -0.164   1.588
HETATM   39  H   UNK  0001      -0.817  -1.765   1.732
HETATM   40  H   UNK  0001      -0.637   4.053   1.130
HETATM   41  C   UNK  0001       0.311   3.656  -1.634
HETATM   42  H   UNK  0001       0.422   3.967  -2.671
HETATM   43  C   UNK  0001      -0.856   3.878  -1.019
HETATM   44  H   UNK  0001      -1.647   4.386  -1.569
CONECT    1    2
CONECT    2    1    5    4
CONECT    3    6    7   39
CONECT    4    7    2   10
CONECT    5    2    6    8
CONECT    6    5    3    9
```

```
CONECT    7    3    4   12
CONECT    8    5
CONECT    9    6
CONECT   10   11    4   38
CONECT   11   10   13   37
CONECT   12    7   13   14
CONECT   13   12   11   28
CONECT   14   17   16   12
CONECT   15   18   19   21
CONECT   16   19   14   29
CONECT   17   14   18   22
CONECT   18   17   15   24
CONECT   19   15   16   20
CONECT   20   19
CONECT   21   15
CONECT   22   23   17   36
CONECT   23   22   26   35
CONECT   24   25   18   26
CONECT   25   24
CONECT   26   27   24   23
CONECT   27   26
CONECT   28   32   13
CONECT   29   16   30
CONECT   30   31   29   40   43
CONECT   31   30
CONECT   32   33   34   28   41
CONECT   33   32
CONECT   34   32
CONECT   35   23
CONECT   36   22
CONECT   37   11
CONECT   38   10
CONECT   39    3
CONECT   40   30
CONECT   41   42   43   32
CONECT   42   41
CONECT   43   44   41   30
CONECT   44   43
END
```

5a

E = -675098.26 kcal

```
REMARK Spartan '10 exported M0001
HETATM    1  H   UNK  0001      4.263  -2.043   1.764
HETATM    2  C   UNK  0001      3.411  -1.483   1.388
HETATM    3  C   UNK  0001      1.198  -0.029   0.411
HETATM    4  C   UNK  0001      2.189  -1.515   2.064
HETATM    5  C   UNK  0001      3.526  -0.734   0.223
HETATM    6  C   UNK  0001      2.431  -0.006  -0.277
HETATM    7  C   UNK  0001      1.099  -0.793   1.577
HETATM    8  H   UNK  0001      4.467  -0.709  -0.322
HETATM    9  C   UNK  0001      2.550   0.724  -1.528
HETATM   10  H   UNK  0001      3.519   0.704  -2.025
HETATM   11  C   UNK  0001      1.516   1.379  -2.099
HETATM   12  H   UNK  0001      1.611   1.886  -3.055
HETATM   13  C   UNK  0001      0.192   1.438  -1.471
HETATM   14  C   UNK  0001      0.013   0.826  -0.055
HETATM   15  O   UNK  0001     -0.740   2.019  -2.014
HETATM   16  O   UNK  0001     -2.416   1.749   1.365
HETATM   17  C   UNK  0001     -1.437   2.687   0.926
HETATM   18  H   UNK  0001     -1.494   3.521   1.629
HETATM   19  C   UNK  0001     -0.045   2.061   0.958
HETATM   20  C   UNK  0001     -2.394   0.538   0.736
HETATM   21  C   UNK  0001     -2.691  -1.954  -0.517
HETATM   22  C   UNK  0001     -3.590  -0.211   0.903
HETATM   23  C   UNK  0001     -1.315   0.051   0.010
HETATM   24  C   UNK  0001     -1.477  -1.205  -0.678
HETATM   25  C   UNK  0001     -3.727  -1.430   0.304
HETATM   26  H   UNK  0001     -4.377   0.229   1.506
HETATM   27  H   UNK  0001     -4.642  -2.005   0.427
HETATM   28  H   UNK  0001      0.095   1.644   1.961
HETATM   29  C   UNK  0001      1.016   3.106   0.727
HETATM   30  H   UNK  0001      0.914   3.707  -0.177
HETATM   31  C   UNK  0001      2.023   3.353   1.565
HETATM   32  H   UNK  0001      2.749   4.137   1.365
HETATM   33  H   UNK  0001      2.165   2.782   2.480
HETATM   34  H   UNK  0001     -1.686   3.036  -0.082
HETATM   35  C   UNK  0001     -2.851  -3.194  -1.186
HETATM   36  H   UNK  0001     -3.781  -3.741  -1.044
HETATM   37  C   UNK  0001     -1.867  -3.693  -2.009
HETATM   38  H   UNK  0001     -2.005  -4.641  -2.522
HETATM   39  C   UNK  0001     -0.679  -2.954  -2.191
HETATM   40  H   UNK  0001      0.097  -3.333  -2.851
```

```
HETATM   41  C   UNK  0001      -0.489  -1.752  -1.542
HETATM   42  H   UNK  0001       0.433  -1.213  -1.715
HETATM   43  H   UNK  0001       0.149  -0.833   2.102
HETATM   44  H   UNK  0001       2.080  -2.105   2.969
CONECT    1    2
CONECT    2    1    5    4
CONECT    3    6    7   14
CONECT    4    7    2   44
CONECT    5    2    6    8
CONECT    6    5    3    9
CONECT    7    3    4   43
CONECT    8    5
CONECT    9   10    6   11
CONECT   10    9
CONECT   11   12    9   13
CONECT   12   11
CONECT   13   11   14   15
CONECT   14    3   13   19   23
CONECT   15   13
CONECT   16   17   20
CONECT   17   18   16   34   19
CONECT   18   17
CONECT   19   14   28   29   17
CONECT   20   23   22   16
CONECT   21   24   25   35
CONECT   22   25   20   26
CONECT   23   20   24   14
CONECT   24   23   21   41
CONECT   25   21   22   27
CONECT   26   22
CONECT   27   25
CONECT   28   19
CONECT   29   30   19   31
CONECT   30   29
CONECT   31   32   33   29
CONECT   32   31
CONECT   33   31
CONECT   34   17
CONECT   35   36   21   37
CONECT   36   35
CONECT   37   38   35   39
CONECT   38   37
CONECT   39   40   37   41
```

CONECT 40 39
CONECT 41 42 39 24
CONECT 42 41
CONECT 43 7
CONECT 44 4
END

Epi-**5a**

E = -675097.45 kcal

REMARK Spartan '10 exported M0001
HETATM 1 H UNK 0001 -3.909 -2.851 -1.721
HETATM 2 C UNK 0001 -3.153 -2.149 -1.381
HETATM 3 C UNK 0001 -1.187 -0.313 -0.504
HETATM 4 C UNK 0001 -1.872 -2.163 -1.936
HETATM 5 C UNK 0001 -3.443 -1.241 -0.369
HETATM 6 C UNK 0001 -2.475 -0.328 0.083
HETATM 7 C UNK 0001 -0.904 -1.258 -1.496
HETATM 8 H UNK 0001 -4.427 -1.236 0.096
HETATM 9 C UNK 0001 -2.778 0.549 1.204
HETATM 10 H UNK 0001 -3.785 0.499 1.614
HETATM 11 C UNK 0001 -1.862 1.364 1.766
HETATM 12 H UNK 0001 -2.087 1.976 2.634
HETATM 13 C UNK 0001 -0.492 1.461 1.251
HETATM 14 C UNK 0001 -0.149 0.737 -0.088
HETATM 15 O UNK 0001 0.342 2.170 1.796
HETATM 16 O UNK 0001 2.019 1.307 -2.036
HETATM 17 C UNK 0001 0.666 1.606 -2.359
HETATM 18 H UNK 0001 0.708 2.463 -3.035
HETATM 19 H UNK 0001 0.219 0.762 -2.902
HETATM 20 C UNK 0001 -0.142 1.960 -1.113
HETATM 21 H UNK 0001 0.408 2.759 -0.602
HETATM 22 C UNK 0001 -1.502 2.473 -1.507
HETATM 23 H UNK 0001 -2.142 1.784 -2.058
HETATM 24 C UNK 0001 -1.932 3.712 -1.264
HETATM 25 H UNK 0001 -2.910 4.051 -1.597
HETATM 26 H UNK 0001 -1.327 4.432 -0.717
HETATM 27 C UNK 0001 2.231 0.493 -0.958
HETATM 28 C UNK 0001 2.986 -1.255 1.103
HETATM 29 C UNK 0001 3.568 0.020 -0.871
HETATM 30 C UNK 0001 1.267 0.147 -0.023

```
HETATM  31  C  UNK  0001     1.638  -0.774   1.021
HETATM  32  C  UNK  0001     3.937  -0.823   0.138
HETATM  33  H  UNK  0001     4.269   0.352  -1.631
HETATM  34  H  UNK  0001     4.960  -1.184   0.208
HETATM  35  C  UNK  0001     0.718  -1.252   1.994
HETATM  36  H  UNK  0001    -0.317  -0.933   1.962
HETATM  37  C  UNK  0001     1.104  -2.131   2.984
HETATM  38  H  UNK  0001     0.371  -2.475   3.709
HETATM  39  C  UNK  0001     2.437  -2.589   3.064
HETATM  40  H  UNK  0001     2.730  -3.278   3.851
HETATM  41  C  UNK  0001     3.355  -2.156   2.134
HETATM  42  H  UNK  0001     4.386  -2.500   2.174
HETATM  43  H  UNK  0001    -1.619  -2.885  -2.708
HETATM  44  H  UNK  0001     0.095  -1.306  -1.915
CONECT    1    2
CONECT    2    1    5    4
CONECT    3    6    7   14
CONECT    4    7    2   43
CONECT    5    2    6    8
CONECT    6    5    3    9
CONECT    7    3    4   44
CONECT    8    5
CONECT    9   10    6   11
CONECT   10    9
CONECT   11   12    9   13
CONECT   12   11
CONECT   13   11   14   15
CONECT   14    3   13   20   30
CONECT   15   13
CONECT   16   17   27
CONECT   17   18   19   16   20
CONECT   18   17
CONECT   19   17
CONECT   20   21   17   14   22
CONECT   21   20
CONECT   22   23   20   24
CONECT   23   22
CONECT   24   25   26   22
CONECT   25   24
CONECT   26   24
CONECT   27   30   29   16
CONECT   28   31   32   41
CONECT   29   32   27   33
```

CONECT 30 27 31 14
CONECT 31 30 28 35
CONECT 32 28 29 34
CONECT 33 29
CONECT 34 32
CONECT 35 36 31 37
CONECT 36 35
CONECT 37 38 35 39
CONECT 38 37
CONECT 39 40 37 41
CONECT 40 39
CONECT 41 42 39 28
CONECT 42 41
CONECT 43 4
CONECT 44 7
END

E-**12**

E = -2290316.89 kcal

REMARK Spartan '10 exported M0001
HETATM 1 H UNK 0001 -1.184 1.793 -4.298
HETATM 2 C UNK 0001 -1.176 1.845 -3.209
HETATM 3 C UNK 0001 -1.127 1.953 -0.420
HETATM 4 C UNK 0001 -0.352 0.937 -2.505
HETATM 5 C UNK 0001 -1.954 2.779 -2.552
HETATM 6 C UNK 0001 -1.920 2.823 -1.139
HETATM 7 C UNK 0001 -0.309 0.971 -1.063
HETATM 8 H UNK 0001 -2.584 3.470 -3.109
HETATM 9 H UNK 0001 -2.530 3.552 -0.608
HETATM 10 C UNK 0001 0.462 -0.021 -3.180
HETATM 11 C UNK 0001 1.243 -0.903 -2.489
HETATM 12 C UNK 0001 0.511 0.052 -0.353
HETATM 13 C UNK 0001 1.298 -0.943 -1.030
HETATM 14 C UNK 0001 0.625 0.135 1.137
HETATM 15 C UNK 0001 0.793 0.424 3.977
HETATM 16 C UNK 0001 -0.230 -0.544 1.998
HETATM 17 C UNK 0001 1.639 0.964 1.735
HETATM 18 C UNK 0001 1.725 1.114 3.160
HETATM 19 C UNK 0001 -0.153 -0.390 3.407
HETATM 20 H UNK 0001 -0.870 -0.938 4.011

```
HETATM   21  H   UNK  0001       0.846   0.539   5.058
HETATM   22  C   UNK  0001       2.582   1.669   0.936
HETATM   23  C   UNK  0001       3.549   2.469   1.505
HETATM   24  C   UNK  0001       2.731   1.949   3.716
HETATM   25  H   UNK  0001       2.779   2.050   4.799
HETATM   26  C   UNK  0001       3.627   2.615   2.911
HETATM   27  H   UNK  0001       4.394   3.250   3.351
HETATM   28  O   UNK  0001       1.972  -1.831  -0.426
HETATM   29  O   UNK  0001      -1.250  -1.366   1.567
HETATM   30  C   UNK  0001      -2.914  -3.602  -2.355
HETATM   31  H   UNK  0001      -2.555  -4.368  -3.043
HETATM   32  C   UNK  0001      -2.251  -3.615  -1.039
HETATM   33  H   UNK  0001      -2.156  -4.581  -0.542
HETATM   34  H   UNK  0001       4.261   2.991   0.869
HETATM   35  H   UNK  0001       2.524   1.552  -0.140
HETATM   36  H   UNK  0001       1.858  -1.639  -3.005
HETATM   37  H   UNK  0001       0.444  -0.036  -4.271
HETATM   38  H   UNK  0001      -1.121   2.002   0.665
HETATM   39  C   UNK  0001      -1.723  -2.512  -0.490
HETATM   40  H   UNK  0001      -1.828  -1.548  -0.983
HETATM   41  C   UNK  0001      -0.892  -2.500   0.756
HETATM   42  H   UNK  0001       0.179  -2.466   0.498
HETATM   43  H   UNK  0001      -1.077  -3.409   1.350
HETATM   44  H   UNK  0001      -2.906  -2.622  -2.830
HETATM   45 Br   UNK  0001      -4.914  -4.077  -2.241
CONECT    1    2
CONECT    2    1    5    4
CONECT    3    6    7   38
CONECT    4    7    2   10
CONECT    5    2    6    8
CONECT    6    5    3    9
CONECT    7    3    4   12
CONECT    8    5
CONECT    9    6
CONECT   10   11    4   37
CONECT   11   10   13   36
CONECT   12    7   13   14
CONECT   13   12   11   28
CONECT   14   17   16   12
CONECT   15   18   19   21
CONECT   16   19   14   29
CONECT   17   14   18   22
CONECT   18   17   15   24
```

CONECT 19 15 16 20
CONECT 20 19
CONECT 21 15
CONECT 22 23 17 35
CONECT 23 22 26 34
CONECT 24 25 18 26
CONECT 25 24
CONECT 26 27 24 23
CONECT 27 26
CONECT 28 13
CONECT 29 16 41
CONECT 30 31 32 44 45
CONECT 31 30
CONECT 32 33 30 39
CONECT 33 32
CONECT 34 23
CONECT 35 22
CONECT 36 11
CONECT 37 10
CONECT 38 3
CONECT 39 40 32 41
CONECT 40 39
CONECT 41 42 43 39 29
CONECT 42 41
CONECT 43 41
CONECT 44 30
CONECT 45 30
END

E-**4a** → **5a**

E = -675071.28 kcal

REMARK Spartan '10 exported M0001
HETATM 1 H UNK 0001 0.436 -4.983 0.477
HETATM 2 C UNK 0001 -0.154 -4.087 0.301
HETATM 3 C UNK 0001 -1.636 -1.773 -0.178
HETATM 4 C UNK 0001 0.472 -2.829 0.429
HETATM 5 C UNK 0001 -1.487 -4.188 -0.056
HETATM 6 C UNK 0001 -2.228 -3.020 -0.304
HETATM 7 C UNK 0001 -0.280 -1.635 0.205
HETATM 8 H UNK 0001 -1.956 -5.163 -0.155

```
HETATM    9  H   UNK  0001      -3.270  -3.092  -0.604
HETATM   10  C   UNK  0001       1.870  -2.729   0.741
HETATM   11  C   UNK  0001       2.491  -1.523   0.810
HETATM   12  C   UNK  0001       0.349  -0.329   0.384
HETATM   13  C   UNK  0001       1.783  -0.278   0.575
HETATM   14  C   UNK  0001      -0.361   0.879  -0.164
HETATM   15  C   UNK  0001      -1.684   3.128  -1.308
HETATM   16  C   UNK  0001      -1.369   1.557   0.513
HETATM   17  C   UNK  0001      -0.027   1.323  -1.499
HETATM   18  C   UNK  0001      -0.687   2.461  -2.069
HETATM   19  C   UNK  0001      -2.017   2.687  -0.060
HETATM   20  H   UNK  0001      -2.782   3.176   0.535
HETATM   21  H   UNK  0001      -2.186   3.992  -1.737
HETATM   22  C   UNK  0001       0.934   0.656  -2.309
HETATM   23  C   UNK  0001       1.234   1.095  -3.581
HETATM   24  C   UNK  0001      -0.353   2.893  -3.378
HETATM   25  H   UNK  0001      -0.868   3.762  -3.781
HETATM   26  C   UNK  0001       0.592   2.228  -4.126
HETATM   27  H   UNK  0001       0.839   2.566  -5.129
HETATM   28  O   UNK  0001       2.410   0.828   0.683
HETATM   29  O   UNK  0001      -1.862   1.295   1.769
HETATM   30  C   UNK  0001       2.228   1.311   2.716
HETATM   31  H   UNK  0001       2.789   0.418   2.971
HETATM   32  H   UNK  0001       2.794   2.236   2.703
HETATM   33  C   UNK  0001       0.847   1.324   2.763
HETATM   34  H   UNK  0001       0.335   2.270   2.612
HETATM   35  H   UNK  0001       1.973   0.559  -4.170
HETATM   36  H   UNK  0001       1.442  -0.218  -1.921
HETATM   37  H   UNK  0001       3.555  -1.439   1.014
HETATM   38  H   UNK  0001       2.428  -3.650   0.899
HETATM   39  H   UNK  0001      -2.217  -0.881  -0.389
HETATM   40  C   UNK  0001       0.104   0.149   2.631
HETATM   41  H   UNK  0001       0.539  -0.803   2.924
HETATM   42  C   UNK  0001      -1.391   0.199   2.551
HETATM   43  H   UNK  0001      -1.829   0.372   3.543
HETATM   44  H   UNK  0001      -1.798  -0.745   2.170
CONECT    1    2
CONECT    2    1    5    4
CONECT    3    6    7   39
CONECT    4    7    2   10
CONECT    5    2    6    8
CONECT    6    5    3    9
CONECT    7    3    4   12
```

```
CONECT    8    5
CONECT    9    6
CONECT   10   11    4   38
CONECT   11   10   13   37
CONECT   12    7   13   14   40
CONECT   13   12   11   28
CONECT   14   17   16   12
CONECT   15   18   19   21
CONECT   16   19   14   29
CONECT   17   14   18   22
CONECT   18   17   15   24
CONECT   19   15   16   20
CONECT   20   19
CONECT   21   15
CONECT   22   23   17   36
CONECT   23   22   26   35
CONECT   24   25   18   26
CONECT   25   24
CONECT   26   27   24   23
CONECT   27   26
CONECT   28   30   13
CONECT   29   16   42
CONECT   30   31   32   28   33
CONECT   31   30
CONECT   32   30
CONECT   33   34   30   40
CONECT   34   33
CONECT   35   23
CONECT   36   22
CONECT   37   11
CONECT   38   10
CONECT   39    3
CONECT   40   41   33   42   12
CONECT   41   40
CONECT   42   43   44   40   29
CONECT   43   42
CONECT   44   42
END
```

Z-**4a** → **5a**

E = -675065.69 kcal

REMARK Spartan '10 exported M0001
HETATM 1 H UNK 0001 -3.338 -2.238 2.872
HETATM 2 C UNK 0001 -3.055 -1.519 2.106
HETATM 3 C UNK 0001 -2.306 0.305 0.128
HETATM 4 C UNK 0001 -1.684 -1.331 1.830
HETATM 5 C UNK 0001 -4.027 -0.814 1.418
HETATM 6 C UNK 0001 -3.645 0.099 0.418
HETATM 7 C UNK 0001 -1.289 -0.388 0.830
HETATM 8 H UNK 0001 -5.078 -0.972 1.640
HETATM 9 H UNK 0001 -4.405 0.643 -0.137
HETATM 10 C UNK 0001 -0.673 -2.082 2.518
HETATM 11 C UNK 0001 0.649 -1.909 2.248
HETATM 12 C UNK 0001 0.120 -0.158 0.577
HETATM 13 C UNK 0001 1.108 -0.973 1.237
HETATM 14 C UNK 0001 0.550 0.513 -0.703
HETATM 15 C UNK 0001 1.476 1.587 -3.178
HETATM 16 C UNK 0001 1.046 1.803 -0.804
HETATM 17 C UNK 0001 0.510 -0.286 -1.909
HETATM 18 C UNK 0001 0.972 0.260 -3.152
HETATM 19 C UNK 0001 1.511 2.336 -2.036
HETATM 20 H UNK 0001 1.885 3.355 -2.028
HETATM 21 H UNK 0001 1.829 2.001 -4.119
HETATM 22 C UNK 0001 0.039 -1.629 -1.920
HETATM 23 C UNK 0001 0.015 -2.374 -3.080
HETATM 24 C UNK 0001 0.931 -0.530 -4.329
HETATM 25 H UNK 0001 1.286 -0.090 -5.259
HETATM 26 C UNK 0001 0.461 -1.823 -4.302
HETATM 27 H UNK 0001 0.436 -2.420 -5.210
HETATM 28 O UNK 0001 2.354 -0.801 1.040
HETATM 29 O UNK 0001 1.183 2.695 0.233
HETATM 30 C UNK 0001 0.268 2.615 1.314
HETATM 31 H UNK 0001 0.347 3.590 1.815
HETATM 32 C UNK 0001 2.931 1.024 2.095
HETATM 33 H UNK 0001 3.054 1.521 1.144
HETATM 34 H UNK 0001 3.822 0.594 2.539
HETATM 35 H UNK 0001 -0.349 -3.397 -3.051
HETATM 36 H UNK 0001 -0.304 -2.080 -0.997
HETATM 37 H UNK 0001 1.417 -2.480 2.761
HETATM 38 H UNK 0001 -0.992 -2.803 3.269
HETATM 39 H UNK 0001 -2.022 0.995 -0.660
HETATM 40 H UNK 0001 -0.760 2.526 0.937
HETATM 41 C UNK 0001 1.776 1.149 2.842

HETATM 42 H UNK 0001 1.743 0.609 3.787

HETATM 43 C UNK 0001 0.538 1.539 2.324

HETATM 44 H UNK 0001 -0.330 1.338 2.950

CONECT 1 2

CONECT 2 1 5 4

CONECT 3 6 7 39

CONECT 4 7 2 10

CONECT 5 2 6 8

CONECT 6 5 3 9

CONECT 7 3 4 12

CONECT 8 5

CONECT 9 6

CONECT 10 11 4 38

CONECT 11 10 13 37

CONECT 12 7 13 14

CONECT 13 12 11 28

CONECT 14 17 16 12

CONECT 15 18 19 21

CONECT 16 19 14 29

CONECT 17 14 18 22

CONECT 18 17 15 24

CONECT 19 15 16 20

CONECT 20 19

CONECT 21 15

CONECT 22 23 17 36

CONECT 23 22 26 35

CONECT 24 25 18 26

CONECT 25 24

CONECT 26 27 24 23

CONECT 27 26

CONECT 28 32 13

CONECT 29 16 30

CONECT 30 31 29 40 43

CONECT 31 30

CONECT 32 33 34 28 41

CONECT 33 32

CONECT 34 32

CONECT 35 23

CONECT 36 22

CONECT 37 11

CONECT 38 10

CONECT 39 3

CONECT 40 30

CONECT 41 42 43 32
CONECT 42 41
CONECT 43 44 41 30
CONECT 44 43
END

E-**12**→*E*-**4a**

E = -2290310.65 kcal

REMARK Spartan '10 exported M0005
HETATM 1 H UNK 0001 2.534 1.598 -4.507
HETATM 2 C UNK 0001 2.516 1.475 -3.425
HETATM 3 C UNK 0001 2.443 1.142 -0.649
HETATM 4 C UNK 0001 1.407 0.807 -2.839
HETATM 5 C UNK 0001 3.546 1.955 -2.650
HETATM 6 C UNK 0001 3.506 1.782 -1.246
HETATM 7 C UNK 0001 1.358 0.635 -1.416
HETATM 8 H UNK 0001 4.389 2.465 -3.111
HETATM 9 H UNK 0001 4.320 2.161 -0.633
HETATM 10 C UNK 0001 0.349 0.292 -3.634
HETATM 11 C UNK 0001 -0.703 -0.361 -3.043
HETATM 12 C UNK 0001 0.246 -0.044 -0.802
HETATM 13 C UNK 0001 -0.754 -0.532 -1.635
HETATM 14 C UNK 0001 0.190 -0.237 0.677
HETATM 15 C UNK 0001 0.153 -0.555 3.495
HETATM 16 C UNK 0001 0.001 0.900 1.511
HETATM 17 C UNK 0001 0.395 -1.533 1.250
HETATM 18 C UNK 0001 0.372 -1.701 2.677
HETATM 19 C UNK 0001 -0.025 0.680 2.940
HETATM 20 H UNK 0001 -0.177 1.560 3.561
HETATM 21 H UNK 0001 0.136 -0.684 4.577
HETATM 22 C UNK 0001 0.644 -2.697 0.464
HETATM 23 C UNK 0001 0.846 -3.933 1.043
HETATM 24 C UNK 0001 0.581 -2.984 3.240
HETATM 25 H UNK 0001 0.555 -3.080 4.325
HETATM 26 C UNK 0001 0.813 -4.088 2.448
HETATM 27 H UNK 0001 0.972 -5.067 2.895
HETATM 28 O UNK 0001 -1.828 -1.238 -1.130
HETATM 29 O UNK 0001 -0.164 2.097 1.043
HETATM 30 C UNK 0001 -3.025 -0.444 -0.900
HETATM 31 H UNK 0001 -3.287 0.090 -1.825

```
HETATM   32  H   UNK  0001      -3.798  -1.192  -0.698
HETATM   33  C   UNK  0001      -2.859   0.516   0.234
HETATM   34  H   UNK  0001      -2.877   0.103   1.240
HETATM   35  H   UNK  0001       1.033  -4.798   0.409
HETATM   36  H   UNK  0001       0.675  -2.600  -0.616
HETATM   37  H   UNK  0001      -1.515  -0.775  -3.635
HETATM   38  H   UNK  0001       0.390   0.417  -4.714
HETATM   39  H   UNK  0001       2.416   1.016   0.427
HETATM   40  C   UNK  0001      -2.580   1.820   0.045
HETATM   41  H   UNK  0001      -2.551   2.222  -0.966
HETATM   42  C   UNK  0001      -2.191   2.717   1.107
HETATM   43  H   UNK  0001      -2.255   2.408   2.138
HETATM   44  H   UNK  0001      -1.785   3.689   0.895
HETATM   45 Br   UNK  0001      -4.411   3.996   1.429
CONECT    1    2
CONECT    2    1    5    4
CONECT    3    6    7   39
CONECT    4    7    2   10
CONECT    5    2    6    8
CONECT    6    5    3    9
CONECT    7    3    4   12
CONECT    8    5
CONECT    9    6
CONECT   10   11    4   38
CONECT   11   10   13   37
CONECT   12    7   13   14
CONECT   13   12   11   28
CONECT   14   17   16   12
CONECT   15   18   19   21
CONECT   16   19   14   29
CONECT   17   14   18   22
CONECT   18   17   15   24
CONECT   19   15   16   20
CONECT   20   19
CONECT   21   15
CONECT   22   23   17   36
CONECT   23   22   26   35
CONECT   24   25   18   26
CONECT   25   24
CONECT   26   27   24   23
CONECT   27   26
CONECT   28   30   13
CONECT   29   16
```

CONECT 30 31 32 28 33
CONECT 31 30
CONECT 32 30
CONECT 33 34 30 40
CONECT 34 33
CONECT 35 23
CONECT 36 22
CONECT 37 11
CONECT 38 10
CONECT 39 3
CONECT 40 41 33 42
CONECT 41 40
CONECT 42 43 44 40 45
CONECT 43 42
CONECT 44 42
CONECT 45 42
END

E-**12** → **5a**

E = -2290307.18 kcal

REMARK Spartan '10 exported M015
HETATM 1 H UNK 0001 -0.868 1.273 -4.295
HETATM 2 C UNK 0001 -0.961 1.348 -3.213
HETATM 3 C UNK 0001 -1.170 1.514 -0.438
HETATM 4 C UNK 0001 -0.261 0.420 -2.412
HETATM 5 C UNK 0001 -1.749 2.334 -2.647
HETATM 6 C UNK 0001 -1.851 2.410 -1.245
HETATM 7 C UNK 0001 -0.355 0.493 -0.989
HETATM 8 H UNK 0001 -2.287 3.039 -3.276
HETATM 9 H UNK 0001 -2.471 3.178 -0.788
HETATM 10 C UNK 0001 0.609 -0.567 -2.999
HETATM 11 C UNK 0001 1.342 -1.424 -2.244
HETATM 12 C UNK 0001 0.312 -0.502 -0.174
HETATM 13 C UNK 0001 1.257 -1.442 -0.787
HETATM 14 C UNK 0001 0.522 -0.230 1.291
HETATM 15 C UNK 0001 0.930 0.416 4.044
HETATM 16 C UNK 0001 -0.062 -0.972 2.312
HETATM 17 C UNK 0001 1.418 0.836 1.673
HETATM 18 C UNK 0001 1.613 1.172 3.055

HETATM	19	C	UNK	0001	0.129	-0.630	3.681
HETATM	20	H	UNK	0001	-0.387	-1.239	4.418
HETATM	21	H	UNK	0001	1.066	0.669	5.093
HETATM	22	C	UNK	0001	2.147	1.592	0.712
HETATM	23	C	UNK	0001	2.995	2.613	1.087
HETATM	24	C	UNK	0001	2.484	2.234	3.409
HETATM	25	H	UNK	0001	2.609	2.469	4.465
HETATM	26	C	UNK	0001	3.165	2.949	2.449
HETATM	27	H	UNK	0001	3.832	3.759	2.733
HETATM	28	O	UNK	0001	1.923	-2.259	-0.112
HETATM	29	O	UNK	0001	-0.890	-2.047	2.166
HETATM	30	C	UNK	0001	-2.553	-1.927	-2.381
HETATM	31	H	UNK	0001	-2.577	-2.255	-3.411
HETATM	32	C	UNK	0001	-1.689	-2.498	-1.467
HETATM	33	H	UNK	0001	-1.144	-3.399	-1.747
HETATM	34	H	UNK	0001	3.541	3.163	0.323
HETATM	35	H	UNK	0001	2.039	1.348	-0.338
HETATM	36	H	UNK	0001	2.022	-2.142	-2.693
HETATM	37	H	UNK	0001	0.687	-0.587	-4.086
HETATM	38	H	UNK	0001	-1.262	1.584	0.642
HETATM	39	C	UNK	0001	-1.490	-1.968	-0.188
HETATM	40	H	UNK	0001	-2.148	-1.159	0.121
HETATM	41	C	UNK	0001	-0.918	-2.772	0.933
HETATM	42	H	UNK	0001	0.090	-3.141	0.693
HETATM	43	H	UNK	0001	-1.569	-3.629	1.146
HETATM	44	H	UNK	0001	-3.038	-0.984	-2.165
HETATM	45	Br	UNK	0001	-5.032	-3.037	-2.352

CONECT	1	2		
CONECT	2	1	5	4
CONECT	3	6	7	38
CONECT	4	7	2	10
CONECT	5	2	6	8
CONECT	6	5	3	9
CONECT	7	3	4	12
CONECT	8	5		
CONECT	9	6		
CONECT	10	11	4	37
CONECT	11	10	13	36
CONECT	12	7	13	14
CONECT	13	12	11	28
CONECT	14	17	16	12
CONECT	15	18	19	21
CONECT	16	19	14	29

CONECT 17 14 18 22
CONECT 18 17 15 24
CONECT 19 15 16 20
CONECT 20 19
CONECT 21 15
CONECT 22 23 17 35
CONECT 23 22 26 34
CONECT 24 25 18 26
CONECT 25 24
CONECT 26 27 24 23
CONECT 27 26
CONECT 28 13
CONECT 29 16 41
CONECT 30 31 32 44 45
CONECT 31 30
CONECT 32 33 30 39
CONECT 33 32
CONECT 34 23
CONECT 35 22
CONECT 36 11
CONECT 37 10
CONECT 38 3
CONECT 39 40 32 41
CONECT 40 39
CONECT 41 42 43 39 29
CONECT 42 41
CONECT 43 41
CONECT 44 30
CONECT 45 30
END

E-**12** → *epi*-**5a**

E = -2290307.11 kcal

REMARK Spartan '10 exported M0003
HETATM 1 H UNK 0001 -1.059 4.495 -0.213
HETATM 2 C UNK 0001 -0.700 3.583 0.261
HETATM 3 C UNK 0001 0.208 1.227 1.434
HETATM 4 C UNK 0001 -0.242 2.536 -0.561
HETATM 5 C UNK 0001 -0.698 3.468 1.643
HETATM 6 C UNK 0001 -0.245 2.275 2.225

```
HETATM    7  C   UNK  0001       0.246   1.326   0.024
HETATM    8  H   UNK  0001      -1.056   4.284   2.265
HETATM    9  H   UNK  0001      -0.254   2.162   3.307
HETATM   10  C   UNK  0001      -0.235   2.668  -1.995
HETATM   11  C   UNK  0001       0.232   1.682  -2.800
HETATM   12  C   UNK  0001       0.679   0.223  -0.816
HETATM   13  C   UNK  0001       0.782   0.431  -2.268
HETATM   14  C   UNK  0001       1.619  -0.799  -0.223
HETATM   15  C   UNK  0001       3.432  -2.608   1.053
HETATM   16  C   UNK  0001       1.317  -2.147  -0.052
HETATM   17  C   UNK  0001       2.933  -0.361   0.192
HETATM   18  C   UNK  0001       3.838  -1.263   0.845
HETATM   19  C   UNK  0001       2.215  -3.035   0.606
HETATM   20  H   UNK  0001       1.889  -4.064   0.728
HETATM   21  H   UNK  0001       4.110  -3.295   1.556
HETATM   22  C   UNK  0001       3.395   0.968  -0.031
HETATM   23  C   UNK  0001       4.647   1.378   0.374
HETATM   24  C   UNK  0001       5.116  -0.810   1.260
HETATM   25  H   UNK  0001       5.777  -1.517   1.759
HETATM   26  C   UNK  0001       5.521   0.486   1.035
HETATM   27  H   UNK  0001       6.505   0.821   1.355
HETATM   28  O   UNK  0001       1.272  -0.418  -3.043
HETATM   29  O   UNK  0001       0.172  -2.783  -0.436
HETATM   30  C   UNK  0001      -2.779   0.029   0.845
HETATM   31  H   UNK  0001      -3.008   0.899   0.242
HETATM   32  C   UNK  0001      -1.886  -0.924   0.407
HETATM   33  H   UNK  0001      -1.603  -1.731   1.080
HETATM   34  H   UNK  0001       4.965   2.400   0.180
HETATM   35  H   UNK  0001       2.745   1.671  -0.539
HETATM   36  H   UNK  0001       0.261   1.790  -3.881
HETATM   37  H   UNK  0001      -0.612   3.598  -2.422
HETATM   38  H   UNK  0001       0.551   0.310   1.901
HETATM   39  C   UNK  0001      -1.317  -0.919  -0.876
HETATM   40  H   UNK  0001      -1.739  -0.242  -1.614
HETATM   41  C   UNK  0001      -0.658  -2.148  -1.418
HETATM   42  H   UNK  0001      -0.063  -1.933  -2.312
HETATM   43  H   UNK  0001      -1.416  -2.904  -1.662
HETATM   44  H   UNK  0001      -3.095   0.071   1.878
HETATM   45 Br   UNK  0001      -5.399  -0.708   0.492
CONECT    1    2
CONECT    2    1    5    4
CONECT    3    6    7   38
CONECT    4    7    2   10
```

```
CONECT    5    2    6    8
CONECT    6    5    3    9
CONECT    7    3    4   12
CONECT    8    5
CONECT    9    6
CONECT   10   11    4   37
CONECT   11   10   13   36
CONECT   12    7   13   14
CONECT   13   12   11   28
CONECT   14   17   16   12
CONECT   15   18   19   21
CONECT   16   19   14   29
CONECT   17   14   18   22
CONECT   18   17   15   24
CONECT   19   15   16   20
CONECT   20   19
CONECT   21   15
CONECT   22   23   17   35
CONECT   23   22   26   34
CONECT   24   25   18   26
CONECT   25   24
CONECT   26   27   24   23
CONECT   27   26
CONECT   28   13
CONECT   29   16   41
CONECT   30   31   32   44   45
CONECT   31   30
CONECT   32   33   30   39
CONECT   33   32
CONECT   34   23
CONECT   35   22
CONECT   36   11
CONECT   37   10
CONECT   38    3
CONECT   39   40   32   41
CONECT   40   39
CONECT   41   42   43   39   29
CONECT   42   41
CONECT   43   41
CONECT   44   30
CONECT   45   30
END
```
